# Stereocontrolled Construction of Multi-Chiral [2.2]Paracyclophanes via Cobaltaphotoredox Dual Catalysis

**DOI:** 10.1021/acscatal.5c03002

**Published:** 2025-06-23

**Authors:** Yang Xu, Neeraj Kumar Pandit, Silvia Meraviglia, Philipp Boos, Paula Anna Maria Stark, Max Surke, Regine Herbst-Irmer, Dietmar Stalke, Lutz Ackermann

**Affiliations:** † Wöhler Research Institute for Sustainable Chemistry (WISCh), 9375 Georg-August-Universität Göttingen Tammannstraße 2, Göttingen 37077, Germany; ‡ Institute of Inorganic Chemistry, Georg-August-Universität Göttingen, Tammannstraße 4, Göttingen 37077, Germany

**Keywords:** photoredox catalysis, C–H activation, enantioselective cobalt catalysis, flow chemistry, [2.2]paracyclophanes

## Abstract

*Ortho*-/*pseudo*-disubstituted multichiral [2.2]­paracyclophanes (PCPs) represent privileged scaffolds for asymmetric catalysis, finding extensive applications as chiral ligands in organic synthesis and functional materials. However, enantioselective C–H activation strategies for accessing these structurally demanding molecules remain largely underexplored. We report a synergistic strategy combining photoredox catalysis with enantioselective cobalt-catalyzed C–H activation that enables efficient construction of central chiral and planar chiral PCP derivatives through kinetic resolution. This method provides access to diverse disubstituted multichiral PCPs in good yields with exceptional levels of enantioselectivity (>20:1 dr, >99% ee) while simultaneously recovering the unreacted enantiomer in a high optical purity (up to 50% yield, >99% ee). Computational studies reveal the favorable pathway for a single enantiomer of the racemic PCP, rationalizing the observed enantioselectivity in terms of attractive dispersion interactions emerging as key contributors during the enantiodetermining step. The synthetic utility is demonstrated through: (1) gram-scale continuous photoflow synthesis with maintained efficiency and (2) versatile downstream functionalization of the products into valuable PCP-based ligands. Our findings represent a paradigm shift for the synthesis of sterically congested chiral PCP architectures, significantly expanding the toolbox for asymmetric synthesis and chiral material design.

## Graphical abstract

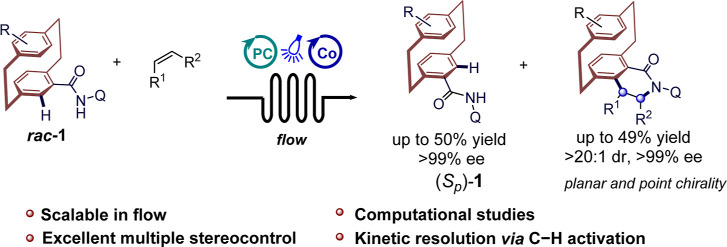

## Introduction

Planar chiral derivatives of [2.2]­paracyclophane (PCP) are a type of structurally interesting and practically useful chiral compounds, characterized by their unique photophysical^1,2^ and optoelectronic properties.^3,4^ As a result, they have found extensive applications in π-stacked polymers,^5,6^ organic luminescent materials,^7–10^ and as a valuable platform for developing chiral ligands or organocatalysts^11–23^ (Figure 1A). Nonetheless, the synthesis of optically pure planar chiral PCPs continues to pose a considerable challenge in organic synthesis, with current approaches primarily relying on several established strategies (Figure 1B, left). The first approach involves the use of chiral high-performance liquid chromatography (HPLC) to separate racemic functionalized PCPs or chemical resolution with stoichiometric amounts of chiral reagents.^24–29^ The second strategy has been well developed by Bräse,^30^ Micouin,^31,32^ Yang,^33^ Jin,^34^ Veselý,^35^ and Wang,^36^ among others, focusing on the construction of monosubstituted chiral PCPs via asymmetric functionalization. Lately, the Xu group elegantly devised asymmetric copper-catalyzed alkynylation reactions of strained dehydro[2.2]­paracyclophane to access monosubstituted planar chiral alkynyl PCPs.^37^ These studies have primarily focused on the preparation of chiral PCPs containing a single stereogenic element and are often constrained by the challenge of accessing symmetrical precursors. Multichiral *ortho*-disubstituted PCP derivatives represent one of the most extensively investigated substitution patterns in chiral ligand design, owing to their unique structural, electronic properties and multiple stereogenic environments. However, synthesizing multichiral PCPs with high diastereo- and enantioselectivities remains a formidable challenge.^38^ As a result, the development of novel approaches to accessing such structures continues to be in high demand.

**Figure 1:**
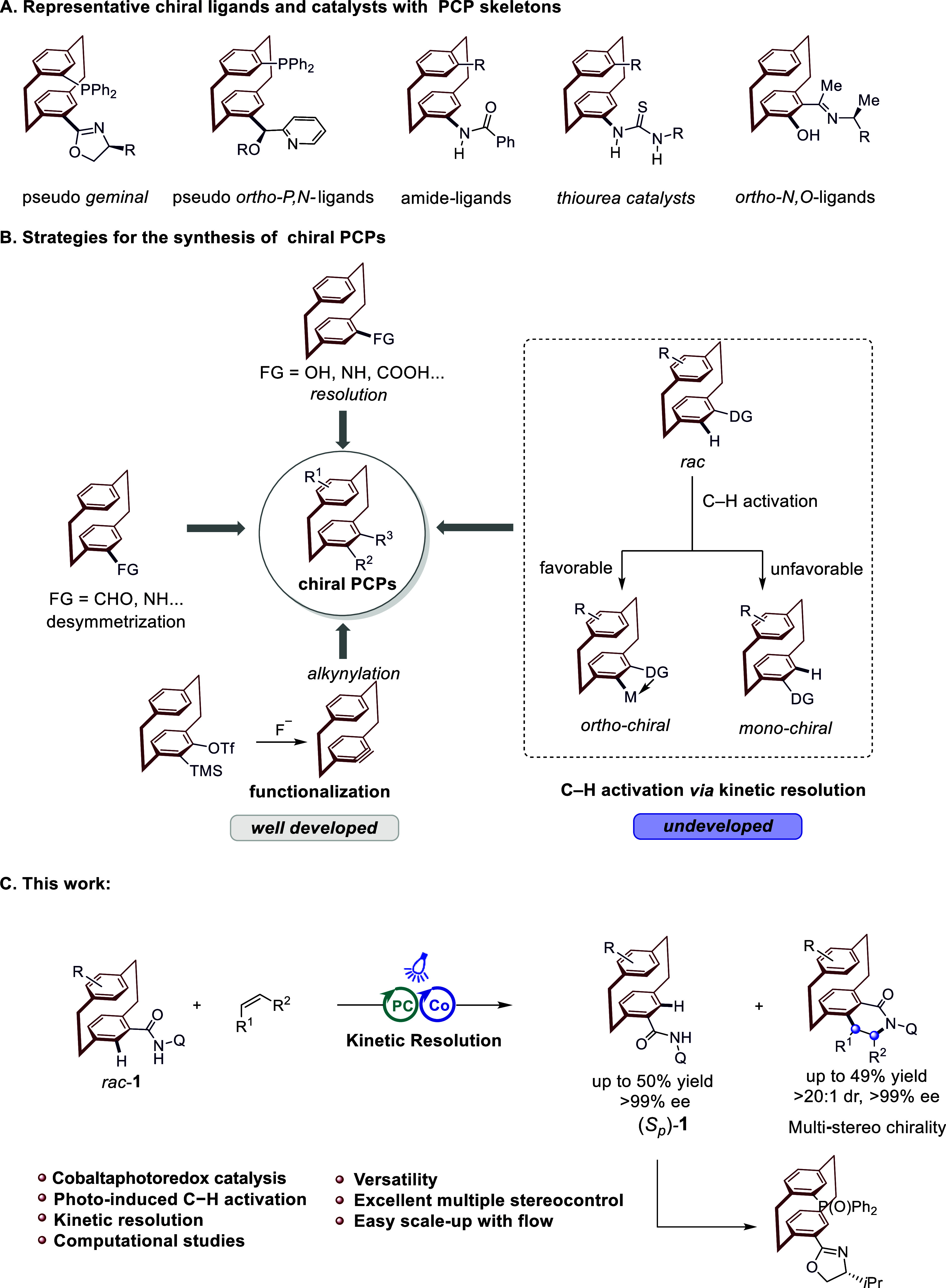
Design blueprint for the assembly of multichiral PCPs via enantioselective C–H activation. (A) Representative chiral ligands and catalysts with PCP skeletons. (B) Strategies for the synthesis of chiral PCPs. (C) Our findings.

Transition-metal-catalyzed enantioselective C–H activation has emerged as an efficient platform for constructing disubstituted chiral molecules including central, axial, inherent, and planar chirality in last decades.^39–51^ Despite recent advancements, the synthesis of multichiral PCPs with high enantioselectivities remains elusive via transition-metal-catalyzed enantioselective C–H activation. The merger of cobalt-catalyzed C–H activation with photoredox catalysis has emerged as an efficient strategy for constructing C–C and C–heteroatom bonds.^52,53^ In 2024, we have developed an enantioselective cobaltaphotoredox dual-catalytic system for controlling central or axial chirality via C–H activation under continuous photoflow conditions.^54^ Experimental results highlighted that the addition of the organic base diisopropylamine (DIPA) effectively facilitated the reaction and gave chiral indolines with excellent yields and enantioselectivities. Based on these findings, we wondered whether a significant challenge of simultaneously controlling the planar chirality of PCPs and generating new contiguous stereocenters via C–H/N–H annulation with alkenes would be viable by innovative catalytic strategies (Figure 1B, right). To this end, we report herein the unprecedented enantioselective C–H/N–H annulation of PCPs through kinetic resolution under cobaltaphotoredox conditions (Figure 1C). This strategy provides access to a series of PCPs with various *ortho*-substitutions, incorporating both planar and central chirality along with valuable chiral amide substrates. The chiral amides can be readily converted to a range of mono- or bidentate planar chiral PCP ligands in a few synthetic steps.

## Results and Discussion

To address the synthetically challenging construction of planar chiral PCPs with multiple stereogenic centers, we conceived an enantioselective cobaltaphotoredox-catalyzed C–H/N–H annulation via a kinetic resolution of [2.2]­paracyclophanecarboxamide. Initial attempts employed Co­(OAc)_2_·4H_2_O and chiral salicyloxazoline ligand **L** for the C–H activation of *rac*-**1a** with 2,5-dihydrofuran **2a**. Under blue-light irradiation (450 nm, ambient air), using Rhodamine 6G as the photocatalyst and NaOTf as the base (24 h), no annulation product was observed. Instead, a dimer byproduct was formed in 16% yield, while 68% of **1a** was recovered with a minimal enantiomeric enrichment (−3% *ee*; Figure 2, entry 5). Replacing the base with NaOPiv significantly improved enantiocontrol: (*S*_*p*_)-**1a** was recovered in 42% yield with 97% *ee*, while the desired annulation product (**3**) was obtained in 28% yield (>20:1 *dr*, 90% *ee*). The moderate yield of **3** was attributed to a competing acyloxylation side reaction of NaOPiv (19% yield; Figure 2, entry 6). Building on prior findings that demonstrated the efficacy of diisopropylamine (DIPA) in cobalt/photoredox systems,^54^ we found that DIPA afforded **3** in 31% yield (>20:1 *dr*, 99% *ee*) and recovered (*S*_*p*_)-**1a** in 69% yield (45% *ee*; Figure 2, entry 3). Extending the reaction time to 48 h further improved the outcome, delivering **3** in 46% isolated yield (>20:1 *dr*, 99% *ee*) and (*S*_*p*_)-**1a** in 49% isolated yield (99% *ee*), corresponding to a selectivity factor (*s*) of 1057 (Figure 2, entry 1). Extending the reaction time to 96 h showed no significant changes in either the yield or enantioselectivities of both the product **3** and (*S*_*p*_)-**1a** (Figure 2, entry 4). Substituting the photoredox system with stoichiometric Ag_2_CO_3_ led to inferior results (lower yield and dr of **3**, 16% dimer byproduct; Figure 2, entry 7). Similarly, affordable oxidants like Mn­(OAc)_2_·4H_2_O and Mn­(OAc)_3_·2H_2_O proved ineffective, yielding poor kinetic resolution (Figure 2, entries 8–9). Furthermore, control experiments confirmed the crucial roles of Co­(OAc)_2_·4H_2_O, ligand **L**, rhodamine 6G, blue light, and DIPA for the enantioselective photocatalysis (Figure 2, entries 10–12).

**Figure 2:**
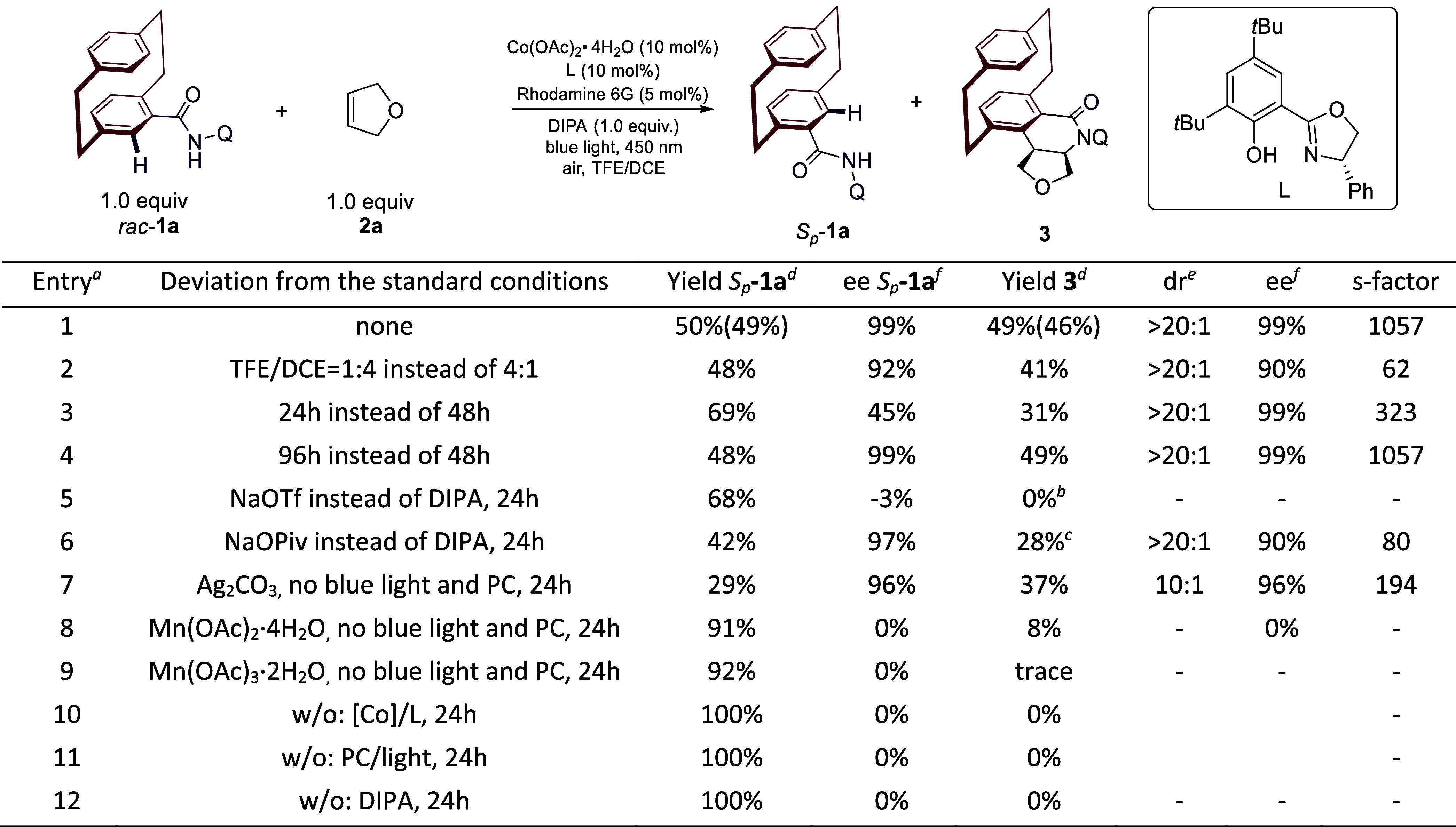
Optimization of the reaction parameters: ^a^Reaction conditions: **1a** (0.15 mmol, 1.0 equiv),**2a** (0.15 mmol, 1.0 equiv), PC (0.0075 mmol, 5 mol %), Co­(OAc)_2_·4H_2_O (0.015 mmol, 10 mol %), **L** (0.015 mmol, 10 mol %), base (0.15 mmol, 1.0 equiv), TFE (1.0 mL), DCE (0.25 mL), 32–35 °C, 48 h. ^b^Dimer byproduct: 16% yield. ^c^Acyloxylation byproduct: 19% yield. ^d^Yields were determined by ^1^H-NMR using 1,3,5-trimethoxybenzene as the internal standard; isolated yields after column chromatography are shown in parentheses. ^e^The dr value was determined by ^1^H-NMR analysis. ^f^The ee value was determined by chiral high-performance liquid chromatography (HPLC) analysis. *Q* = 8-quinolinyl. PC = photocatalyst. TFE = 2,2,2-trifluoroethanol. DCE = 1,2-dichloroethane. DIPA = diisopropylamine. Calculated conversion, *C* = ee_SM_/(ee_SM_ + ee_PR_); selectivity (*S*) = ln­[(1 – *C*)­(1 – ee_SM_)]/ln­[(1 – *C*)­(1 + ee_SM_)].

With the optimized reaction conditions in hand, we explored the versatility of the PCP kinetic resolution via cobaltaphotoredox catalysis. As shown in Figure 3, this strategy enabled the synthesis of chiral *N*-heterobicyclic PCP derivatives (**4**–**5**) in excellent yields and enantioselectivities (43–46% yield, >20:1 dr, 99% ee) while simultaneously recovering the enantiopure starting material (*S*_*p*_)-**1a** in 46–48% yield with 97–99% *ee*. Bridged heterobicyclic alkenes exhibit diverse reactivity and serve as valuable intermediates for the stereoselective synthesis of chiral bicyclic frameworks.^55–59^ To explore their utility, we evaluated the compatibility of bridged heterobicyclic alkenes, affording a variety of chiral PCPs bearing multiple stereocenters. Our strategy showed ample scope, delivering a wide range of chiral bridged dihydroisoquinolinones (**6**–**14**) with high enantioselectivity and tolerance for diverse functional groups. For instance, methyl-, heterocyclic-, and bromide-substituted 7-oxabenzonorbornadienes underwent efficient conversion to the desired products (**6**–**9**) with excellent diastereo- and enantioselectivity, alongside efficient kinetic resolution of the starting materials ((*S*_*p*_)-**1a**, 45–49% yield, 99% ee). Both *exo*- and *endo*-oxonorbornene imides reacted efficiently with *rac*-**1a**, yielding PCP-fused polycycles with four contiguous stereocenters (**10**–**11**) in good yields (46%) and high stereoselectivity (>20:1 dr, 91–99% ee). Moreover, the devised photocatalysis was not limited to 7-oxabenzonorbornadienes; with 7-azabenzonorbornadienes (Figure 3, **12**–**14**), the desired product formation with satisfying enantiocontrol was also achieved. Terminal alkenes proved to be also amenable under the cobaltaphotoredox conditions (**15**–**17**), furnishing chiral PCPs with an outstanding stereoselectivity. Allenes were likewise well tolerated, affording the corresponding annulation product in good yields (40%) and excellent enantioselectivities (96% ee) (**18**). Furthermore, the method accommodated structurally complex substrates, including drug-derived motifs such as estrone, *S*-*i*buprofen, and Boc-*D*-alanine (**19**–**21**). To further demonstrate the synthetic utility, we explored the scope of PCP derivatives (*rac*-**1**). Arenes with pseudopara substitutions (**23**–**26**) and halogenated variants (**22**) proved viable, as did diphenylphosphine oxide-functionalized PCPs (**27**–**28**), which afforded products in good yields with an excellent stereocontrol (32–48% yield, >20:1 dr, 99% ee) while recovering (S_*p*_)-**1g** in 49–57%, with 78–99% ee.

**Figure 3:**
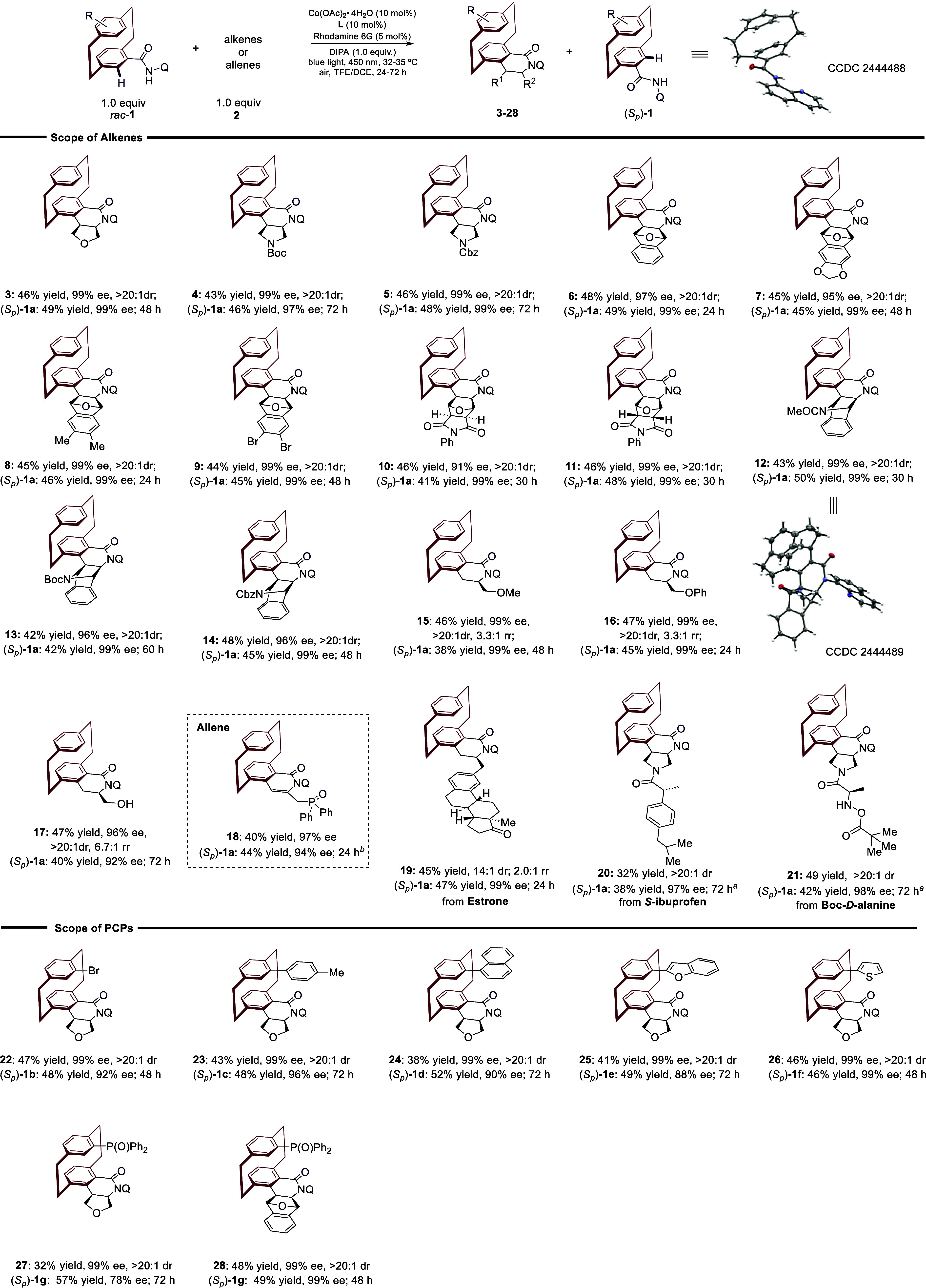
Scope of amides with alkenes. Reaction conditions: **1** (0.15 mmol, 1.0 equiv),**2** (0.15 mmol, 1.0 equiv), Rhodamine 6G (0.0075 mmol, 5 mol %), Co­(OAc)_2_·4H_2_O (0.015 mmol, 10 mol %), **L** (0.015 mmol, 10 mol %), DIPA (0.15 mmol, 1.0 equiv), TFE (1.0 mL), DCE (0.25 mL), 32–35 °C, 24–72 h. ^a^Co­(OAc)_2_·4H_2_O (0.030 mmol, 20 mol %), **L** (0.030 mmol, 20 mol %).^b^ **2** (0.075 mmol, 0.5 equiv).

### Practicality Studies

To demonstrate the synthetic utility and address the challenges often met during scale-up, we carried out a gram-scale reaction under continuous flow conditions with a reduced loading of rhodamine 6G (3 mol %), Co­(OAc)_2_·4H_2_O (5 mol %), and **L** (6 mol %), resulting in the formation of **6** with a yield of 45%, >20:1 dr, and 99% ee, while recovering (S_*p*_)-**1a** in 49% yield with 99% ee (Figure 4A). However, under this flow conditions, the reaction of **1a** with 1.0 equiv of allene **2t** afforded the C–H/N–H annulation product **18** in >50% yield with 83% ee. We hypothesized that remaining substrate (*S*_*p*_)-**1a** might slowly react with excess allene. Consequently, reducing the loading of allene to 0.5 equiv delivered both **18** and (S_*p*_)-**1a** in a good yield with an excellent enantioselectivity. The directing group (Q) of (S_*p*_)-**1g** could be readily removed to afford phosphorus-substituted paracyclophanecarboxylic acid (*S*_*p*_)-**29** in 68% yield with 99% ee. The carboxylic acid (*S*_*p*_)-**29** served as a versatile intermediate for the synthesis of various chiral derivatives. For instance, coupling with (*R*)-(+)-1-(1-naphthyl) ethylamine provided the chiral phosphorus-containing amide ligand (*S*_*p*_)-**31** in a good yield, while reaction with *l* -valinol followed by cyclization furnished precursors to chiral pseudogeminal ligands (*S*_*p*_)-**32**.

**Figure 4:**
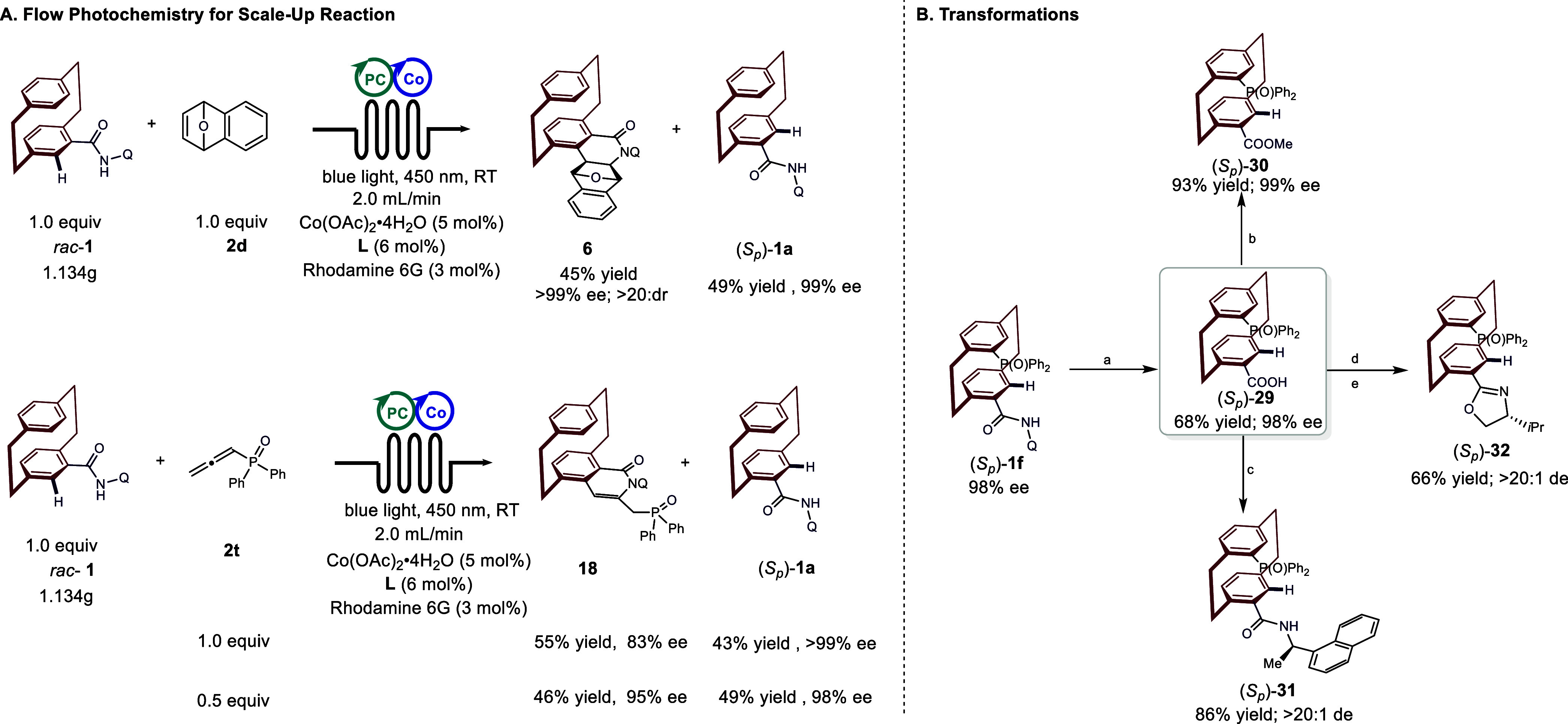
(A) Flow photochemistry for the scale-up reaction. (B) Transformations; a: 50% H_2_SO_4_ (aq.), dioxane at 120 °C for 24 h. b: K_2_CO_3_ and MeI in MeCN at RT for 16 h. c: DMAP, EDCI, and *R*-1-(2-naphthyl)­ethylamine in DCM at RT for 24 h. d: DMAP, EDCI, and *l* -valinol in DCM at RT for 24 h. e: MsCl, DMAP, and NEt_3_ in DCM at RT for 16 h.

### Density Functional Theory (DFT) Calculations

To rationalize the origin of the observed enantioselectivity with the racemic PCP substrate, we performed density functional theory (DFT) calculations on both enantiomers for the enantio-determining step. Substrate *rac*-**1a** and alkene **2d** (7-oxabenzonorbornadiene) were considered to model the cobalt-catalyzed migratory insertion (see Supporting Information for more details). Although the experimental reaction starts from a racemic mixture, only one enantiomer undergoes the desired transformation.

The calculations revealed an exergonic nature and a distinct difference in the activation barriers for the alkene migratory insertion step (Figure 5A). The *R*_*p*_ enantiomer (*ent1*) proceeds through an energy barrier of 21.3 kcal mol^–1^ (**TS1^1^**-*ent1*), kinetically favoring the insertion step. Interestingly, while the competing *S*_*p*_ enantiomer (*ent2*) pathway leads to a more stable postinsertion intermediate **Int3^1^**-*ent2* (−32.8 kcal mol^–1^), the corresponding transition state is 5.2 kcal mol^–1^ higher in energy than *ent1*. These results were suggestive of the enantioselectivity arising from the kinetic control during the insertion step rather than thermodynamic stabilization in the later intermediate **Int3**. To better understand the nature of the migratory insertion transition state (**TS1**), we performed a distortion–interaction analysis, for both PCP enantiomers.^60^ The computed relationship between distortion (Δ*E*_distort_), interaction (Δ*E*_interact_), and activation (Δ*E*_activate_) energies is shown in Figure 5B. While the alkene distortion energies were higher for *ent2*, a notably larger distortion energy was required for **Int1** ^**3**^-*ent2* to achieve its corresponding **TS1** geometry. This greater distortion requirement accounts for the higher activation energy observed for the *ent2* enantiomer.

**Figure 5:**
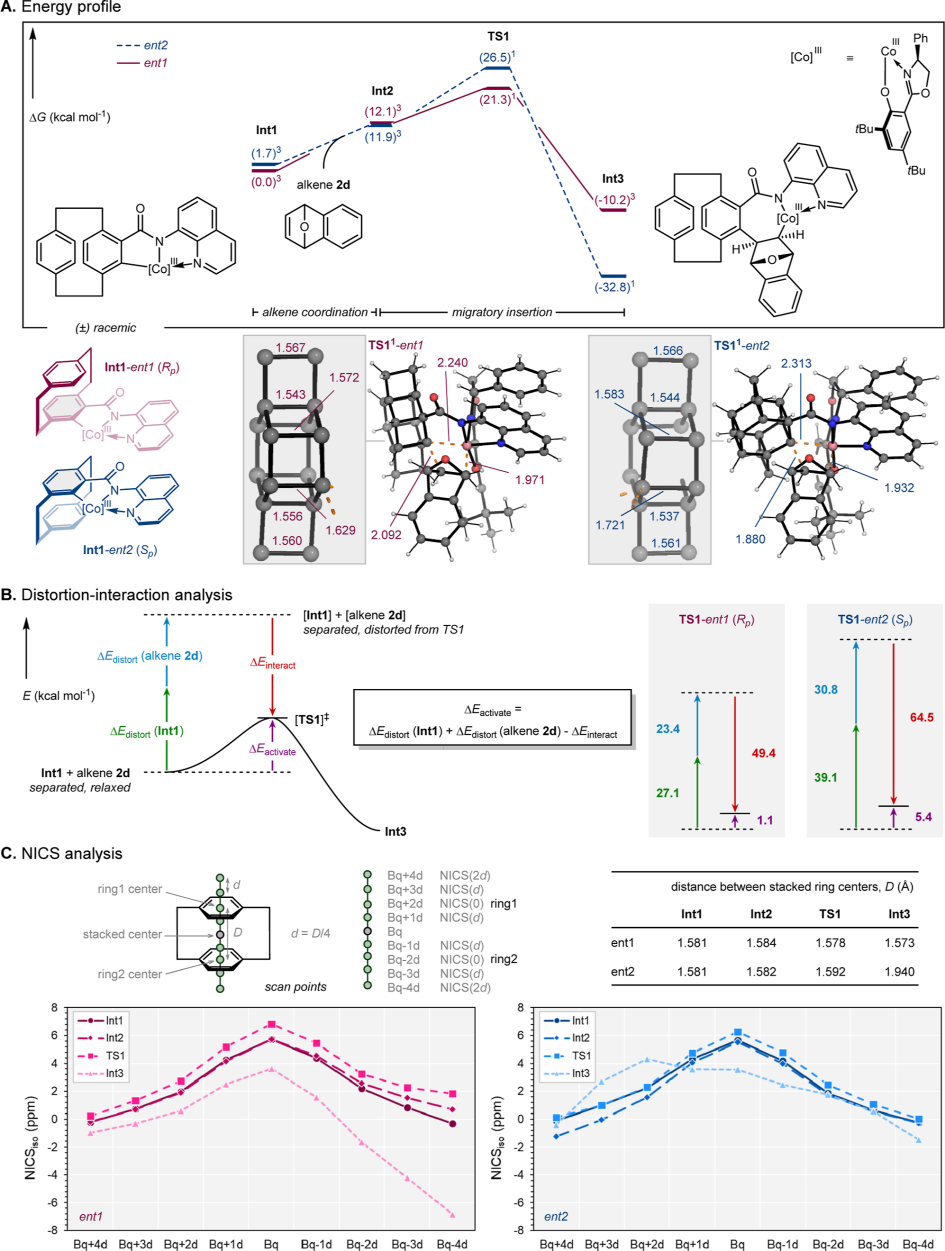
Computational studies. (A) Computed Gibbs free energies (Δ*G*_308.15_) for the elementary steps. (B) Distortion analysis of both enantiomers’ **TS1**. (C) NICS analysis of the PCP fragment for both enantiomers. Energies reported are in kcal mol^–1^, hydrogen atoms from focused PCP fragments were removed for clarity, and the key interatomic distances are labeled in Å.

To further explore structural influences on this reactivity, notably given the rigid and aromatic nature of the PCP scaffold, we carried out isotropic Nucleus-Independent Chemical Shift (NICS)^61,62^ analyses along the computed ground-state structures (Figure 5C). Specifically, we monitored changes in the aromatic character and π–π stacking interactions within the cyclophane unit (see Supporting Information for more details). For the reactive enantiomer (*ent1*), we observed a progressive disorder of aromatic stacking as the PCP fragment advanced toward the enantio-determining transition state and beyond. This loss of aromatic interaction appears to reduce steric repulsion, which allowed for a more favorable geometric arrangement during migratory insertion. In contrast, the nonreactive enantiomer (*ent2*) retained stronger π–π interactions throughout, which imposed a more rigid geometry and higher distortion in the ring2 in **TS1** and **Int3**.

Calculations without dispersion corrections revealed a consistent energetic trend with the same enantiomeric pathway favored as in the dispersion-corrected model. However, all transition states exhibited significantly higher energy barriers, emphasizing the influence of dispersion forces on accurate modeling of this sterically and electronically complex system.

## Conclusion

We have developed an efficient strategy for the enantioselective synthesis of multichiral, *ortho*-disubstituted PCPs via cobaltaphotoredox-catalyzed C–H/N–H annulation. This approach enabled the efficient construction of both planar and central chirality, delivering a range of chiral PCP derivatives with excellent enantioselectivity. DFT calculations provided key mechanistic insights into the origin of the enantioselectivity, revealing a 5.2 kcal mol^–1^ difference in the activation barrier of the diastereomeric transition states during alkene migratory insertion. The more reactive enantiomer (*R_p_*) undergoes a progressive disruption of π–π stacking interactions within the PCP scaffold, reducing steric hindrance and facilitating a more favorable geometry at the transition state. In contrast, the nonreactive enantiomer (*S*_*p*_) retains rigid π–π interactions, resulting in greater distortion and a higher activation barrier. The synthetic utility was highlighted by gram-scale synthesis with continued flow without erosion of enantioselectivity and late-stage functionalization of bioactive molecule hybrids. We anticipate that our findings will pave the way for the development of more robust and versatile strategies toward diversely substituted chiral PCPs.

## Materials and Methods

### General Procedure for the Synthesis of Products **3**–**28**

A 10 mL vial was charged with the amide *rac*-**1** (0.15 mmol, 1.0 equiv), alkenes or allenes **2** (0.15 mmol, 1.0 equiv), Co­(OAc)_2_·4H_2_O (3.7 mg, 10 mol %), **L** (5.3 mg, 10 mol %), Rhodamine 6G (3.6 mg, 5 mol %), DIPA (21 μL, 0.15 mmol, 1.0 equiv), and a Teflon-coated magnetic stirring bar. Then, DCE (0.25 mL) and TFE (1 mL) were added. The vial was stirred at room temperature under blue LEDs (450 nm) for 24h–72 h. After completion of the reaction, the solvent was then removed under vacuum and the residue was purified by column chromatography on silica gel with *n*-hexane/ethyl acetate (5:1–1:2) as an eluent to the give corresponding products **3**–**28** and (*S*_*p*_)-**1**. Racemic samples of compounds **3**–**28** were prepared with racemic **L**.

### DFT Methods for Computational Studies

Gibbs free energies for the elementary steps were computed at the PW6B95-D4/def2-TZVPP + SMD­(TFE)//TPSS-D3­(BJ)/def2-SVP level of theory. Distortion–interaction analysis of **TS1** for both enantiomers was performed at the PW6B95-D3­(BJ)/def2-TZVPP + SMD­(TFE)//TPSS-D3­(BJ)/def2-SVP level of theory. The NICS analysis of the PCP fragment for both enantiomers was performed at the PBE0-GIAO/6-311++G­(d,p)//TPSS-D3­(BJ)/def2-SVP level of theory.

All information pertaining to the materials and methods used in this study is provided in the Supporting Information.

## Supplementary Material



## Data Availability

Crystallographic data^63–68^ for the structures reported in this article have been deposited at the Cambridge Crystallographic Data Centre, under deposition numbers CCDC 2444488 ((S_*p*_)-**1a**) and 2444489 (**12**). Copies of the data can be obtained free of charge via https://www.ccdc.cam.ac.uk/structures/.

## Notes

General remarks, synthesis of substrates, optimization of reaction conditions, experimental, transformations, mechanistic studies, computational studies, X-ray crystallographic data, references, Cartesian coordinates of the optimized structures, and NMR spectra (PDF)

## Notes

The authors declare that the data supporting the findings of this study are available within the paper and its Supporting Information files. Source data is provided with this paper. All other requests for materials and information should be addressed to the corresponding authors.

## Abbreviations

PCP: [2,2]­paracyclophane
Q: 8-quinolinyl
PC: photocatalyst
TFE: 2,2,2-trifluoroethanol
DCE: 1,2-dichloroethane
DIPA: diisopropylamine
NMR: nuclear magnetic resonance.
