# Supplementary material for: Stereocontrolled Construction of Multi-Chiral [2.2]Paracyclophanes via Cobaltaphotoredox Dual Catalysis
Source: ACS Catal. 2025 Jun 23;15(13):11716–25. doi: 10.1021/acscatal.5c03002 (PMC12235589; doi:10.1021/acscatal.5c03002)
Supplement: Supplementary file 1 [file cs5c03002_si_001.pdf]

Electronic Supporting Information

Stereocontrolled Construction of Multi-Chiral [2.2]Paracyclophanes via Cobaltaphotoredox Dual Catalysis

Yang Xu,[a] Neeraj Kumar Pandit,[a] Silvia Meraviglia,[a] Philipp Boos,[a] Paula Anna Maria Stark,[b] Max Surke,[a] Regine Herbst-Irmer,[b] Dietmar Stalke[b] and Lutz Ackermann[a]*

[a]Wöhler Research Institute for Sustainable Chemistry (WISCh), Georg-August-Universität Göttingen Tammannstraße 2, 37077, Göttingen, Germany

[b]Institute of Inorganic Chemistry, Georg-August-Universität Göttingen Tammannstraße 4, 37077, Göttingen, Germany

*Corresponding author: Lutz.Ackermann@chemie.uni-goettingen.de

Contents

1 General Remarks **S1**

2 Synthesis of Substrates **S2**

3 Optimization of Reaction Conditions **S9**

4 Experimental **S12**

5 Transformations **S53**

6 Mechanistic Studies **S58**

7 Computational Studies **S60**

8 X-Ray Crystallographic Data **S71**

References **S79**

Cartesian coordinates of the optimized structures **S81**

NMR Spectra **S220**

1 General Remarks

Catalytic reactions were performed in 10 mL vial using a Kessil PR160L photoreactor with 450 nm irradiation. The reaction temperature was measured by digital thermometer PCE-T 390, which was in the range of 32 to 35 °C. Solvents for column chromatography and extraction (EtOAc, *n*-hexane, DCM) were distilled prior to their use. Routine TLC analysis was carried out on aluminium sheets coated with silica gel 60 F254, 0.2 mm thickness. Plates were analyzed using a 254 nm UV lamp. Chromatography was carried out on Merck silica gel 60 (40–63 µm). The substrates were either purchased directly from commercial suppliers or prepared according to previously reported procedures, if not noted otherwise. All other reagents and solvents used in this study were purchased from commercial sources and used as received. NMR spectra were recorded on a Varian Mercury VX 300 or Bruker Avance III 400 in the solvent indicated; chemical shifts (δ) are given in ppm relative to the residual solvent peak. All IR spectra were recorded on a Bruker FT-IR Alpha-P device. EI-MS spectra were recorded on Jeol AccuTOF at 70eV, ESI-MS spectra on Bruker MicrOTOF and maXis. HPLC chromatograms were recorded on an Agilent 1290 Infinity using CHIRALPAK® IA-3, IB-3, IC-3, ID-3, IE-3, IF-3, AD-3, and CHIRALCEL® OD-3, OJ-3 columns (3.0 µm particle size; Ø: 4.6 mm and 250 mm length). Optical rotations were measured with Anton Paar MCP 150 at 20 °C under a Na/Hg lamp, λ = 589 nm (c in g/100 mL). Values were denoted as specific rotations: $[\alpha]_D^{20}$. X-ray diffraction experiments for the compounds analyzed were carried out at 100(2) K on a Bruker D8 Venture four-circle-diffractometer from Bruker AXS GmbH. M. p.: Stuart melting point apparatus SMP3, Barloworld Scientific, values are uncorrected. Absorption spectra were measured on a Jasco V-770 spectrophotometer.

2 Synthesis of Substrates

The amides *rac*-**1a**-**1b** were synthesized according to literature procedure[1].

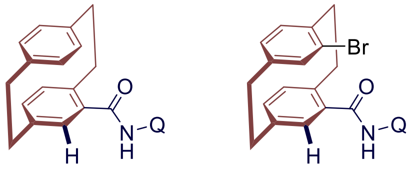

The amides *rac*-**1c**-**1g** were synthesized according to the following procedure.

General Procedure 1:

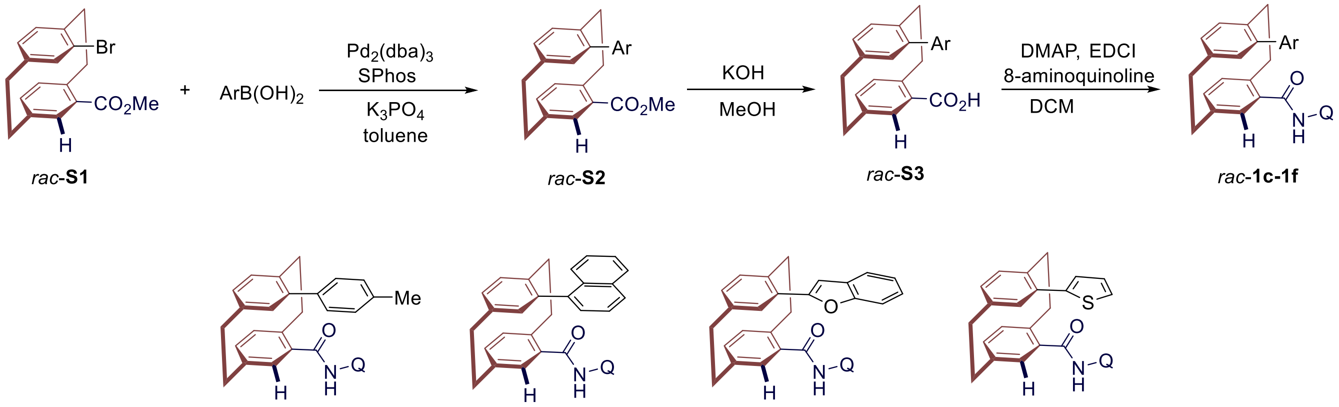

Step 1: Synthesis of *rac*-**S2**

A mixture of *rac*-**S1** (1.0 mmol), $ArB(OH)_2$ (1.5 mmol), $Pd_2(dba)_3$ (0.025 mmol), SPhos (0.1 mmol), and K_3PO_4 (3.0 mmol) in toluene (10 mL) was stirred under a nitrogen atmosphere at 110 °C (oil bath temperature) for 24 h. After cooling to room temperature, the reaction mixture was diluted with ethyl acetate (10 mL) and 1 M HCl (10 mL). The organic layer was separated, and the aqueous phase was extracted with ethyl acetate (3 × 10 mL). The combined organic extracts were dried over anhydrous Na_2SO_4, filtered, and concentrated under reduced pressure. The crude product was purified by column chromatography on silica gel using *n*-hexane /ethyl acetate (50:1 to 20:1) as the eluent to afford *rac*-**S2**.

Step 2: Synthesis of *rac*-**S3**

rac-**S2** (1.0 mmol) was dissolved in 25% KOH in methanol (10 mL), and the mixture was stirred at 80 °C (oil bath temperature) for 24 h. After cooling to room temperature, the reaction mixture was diluted with ethyl acetate (10 mL), and the pH was adjusted to 2–3 by adding 2 M HCl. The organic layer was

separated, and the aqueous phase was extracted with ethyl acetate (3 × 10 mL). The combined organic extracts were dried over anhydrous Na_2SO_4, filtered, and concentrated under reduced pressure to give crude *rac*-**S3**, which was used directly in the next step without further purification.

Step 3: Synthesis of *rac*-**1c-1f**

To a solution of *rac*-**S3** (1.0 mmol) in dry dichloromethane (5 mL) under a nitrogen atmosphere, DMAP (1.0 equiv.) and EDCI (1.2 equiv.) were added. The reaction mixture was stirred at room temperature for 16 h. After completion of the reaction (monitored by TLC), the solvent was removed under reduced pressure, and the residue was purified by column chromatography on silica gel using *n*-hexane /ethyl acetate (20:1 to 5:1) as the eluent to afford the desired product *rac*-**1c-1f**.

***rac*-N-(quinolin-8-yl)-4^2-(p-tolyl)-1,4(1,4)-dibenzenacyclohexaphane-1^2-carboxamide (*rac*-1c)**

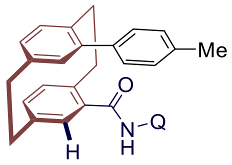

rac-**1c**

Prepared according to general procedure **1** on a 1.0 mmol scale, column chromatography (ethyl acetate/*n*-hexane) afforded the compound *rac*-**1c** as a white solid.

^{1}H NMR (400 MHz, $CDCl_3$) δ 10.12 (s, 1H), 8.79 (dd, J = 4.2, 1.6 Hz, 1H), 8.47 (dd, J = 7.2, 1.9 Hz, 1H), 8.18 (dt, J = 8.3, 1.5 Hz, 1H), 7.58 – 7.49 (m, 2H), 7.45 (ddd, J = 8.3, 4.2, 1.1 Hz, 1H), 7.36 (d, J = 1.8 Hz, 1H), 7.32 – 7.25 (m, 2H), 6.87 (d, J = 7.8 Hz, 3H), 6.81 (d, J = 7.7 Hz, 1H), 6.76 (d, J = 7.7 Hz, 1H), 6.70 (d, J = 1.9 Hz, 1H), 6.60 (dd, J = 7.7, 1.9 Hz, 1H), 4.00 – 3.84 (m, 2H), 3.39 – 2.90 (m, 6H), 2.97 (s, 3H) ppm;

^{13}C NMR (101 MHz, $CDCl_3$) δ 165.0 (C_q), 148.0 (CH), 142.5 (C_q), 142.1 (C_q), 140.0 (C_q), 138.9 (C_q), 138.8 (C_q), 137.8 (C_q), 137.7 (CH), 136.7 (CH), 136.2 (CH), 135.8 (C_q), 135.5 (CH), 135.4 (CH), 135.1 (C_q), 132.5 (C_q), 132.1 (CH), 131.8 (CH), 130.6 (CH), 129.4 (CH), 128.9 (CH), 127.9 (C_q), 127.2 (CH), 121.4 (CH), 120.9 (CH), 116.7 (CH), 37.1 (CH_2), 35.3 (CH_2), 35.1 (CH_2), 33.8 (CH_2), 21.0 (CH_3) ppm;

HRMS (ESI): *m/z* $[M+H]^+$ calcd for $C_{33}H_{28}N_2O$: 469.2274; found: 469.2273.

***rac*-4^2-(naphthalen-1-yl)-N-(quinolin-8-yl)-1,4(1,4)-dibenzenacyclohexaphane-12-carboxamide (*rac*-1d)**

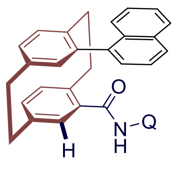

rac-**1d**

Prepared according to general procedure **1** on a 1.0 mmol scale, column chromatography (ethyl acetate/*n*-hexane) afforded the compound *rac*-**1d** as a white solid.

^{1}H NMR (300 MHz, $CDCl_3$) δ 10.13 (s, 1H), 8.68 – 8.53 (m, 1H), 8.15 (dd, *J* = 8.3, 1.7 Hz, 2H), 7.84 (d, *J* = 8.2 Hz, 1H), 7.74 (t, *J* = 7.3 Hz, 2H), 7.62 – 7.28 (m, 7H), 7.13 – 7.01 (m, 1H), 6.97 – 6.84 (m, 2H), 6.84 – 6.68 (m, 3H), 3.80 (ddd, *J* = 12.9, 8.1, 3.0 Hz, 1H), 3.43 – 2.77 (m, 7H) ppm;

^{13}C NMR (75 MHz, $CDCl_3$) δ 165.3 (C_q), 148.0 (CH), 141.8 (C_q), 140.3 (C_q), 140.1 (C_q), 139.3 (C_q), 139.2 (C_q), 138.8 (C_q), 138.8 (C_q), 136.8 (CH), 136.2 (CH), 135.7 (CH), 135.1 (C_q), 134.2 (CH), 133.5 (C_q), 132.9 (CH), 132.2 (CH), 132.2 (C_q), 131.6 (CH), 131.3 (C_q), 128.3 (CH), 128.2 (CH), 127.9 (C_q), 127.4 (CH), 127.2 (CH), 125.8 (CH), 125.6 (CH), 125.2 (CH), 121.5 (CH), 121.1 (CH), 116.6 (CH), 36.9 (CH_2), 35.4 (CH_2), 35.2 (CH_2), 34.6 (CH_2) ppm;

HRMS (ESI): *m/z* $[M+H]^+$ calcd for $C_{36}H_{28}N_2O$: 505.2274; found: 505.2274.

***rac*-4^2-(benzofuran-2-yl)-N-(quinolin-8-yl)-1,4(1,4)-dibenzenacyclohexaphane-1^2-carboxamide (*rac*-1e)**

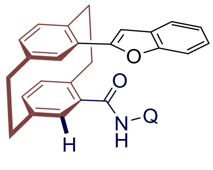

rac-**1e**

Prepared according to general procedure **1** on a 1.0 mmol scale, column chromatography (ethyl acetate/*n*-hexane) afforded the compound *rac*-**1e** as a white solid

^{1}H NMR (300 MHz, $CDCl_3$) δ 10.01 (s, 1H), 8.82 (dd, *J* = 4.2, 1.7 Hz, 1H), 8.11 (dd, *J* = 8.3, 1.7 Hz, 1H), 7.90 (dd, *J* = 7.8, 1.3 Hz, 1H), 7.44 (dd, *J* = 8.3, 4.2 Hz, 1H), 7.35 (d, *J* = 1.3 Hz, 0H), 7.32 (d, *J* = 1.3 Hz, 1H), 7.28 (d, *J* = 1.6 Hz, 1H), 7.24 – 7.09 (m, 3H), 7.03 – 6.86 (m, 3H), 6.82 – 6.72 (m, 4H), 6.64 (dd, *J* = 7.8, 1.9 Hz, 1H), 4.32 – 3.89 (m, 2H), 3.38 – 2.97 (m, 6H) ppm;

^{13}C NMR (75 MHz, CDCl3) δ 165.1 (C_q), 155.8 (C_q), 154.4 (C_q), 147.9 (CH), 140.9 (C_q), 139.9 (C_q), 139.6 (C_q), 138.6 (C_q), 137.8 (C_q), 136.4 (CH), 136.1 (CH), 136.0 (CH), 135.6 (CH), 134.8 (C_q), 134.5 (C_q), 132.8 (CH), 131.5 (CH), 131.1 (C_q), 130.9 (CH), 129.2 (C_q), 127.8 (C_q), 127.4 (CH), 123.5 (CH), 122.2 (CH), 121.3 (CH), 120.7 (CH), 120.5 (CH), 116.4 (CH), 110.8 (CH), 104.8 (CH), 35.2 (CH_2), 35.1 (CH_2), 34.8 (CH_2) ppm;

HRMS (ESI): *m/z* $[M+H]^+$ calcd for $C_{34}H_{26}N_2O_2$: 495.2067; found: 495.2069.

***rac*-*N*-(quinolin-8-yl)-4^2-(thiophen-2-yl)-1,4(1,4)-dibenzenacyclohexaphane-1^2-carboxamide (*rac*-1f)**

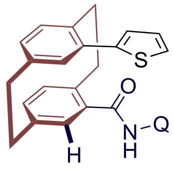

rac-**1f**

Prepared according to general procedure **1** on a 1.0 mmol scale, column chromatography (ethyl acetate/*n*-hexane) afforded the compound *rac*-**1f** as a white solid.

^{1}H NMR (400 MHz, $CDCl_3$) δ 10.08 (s, 1H), 8.78 (dd, *J* = 4.2, 1.7 Hz, 1H), 8.53 (dd, *J* = 7.5, 1.5 Hz, 1H), 8.14 (dd, *J* = 8.3, 1.7 Hz, 1H), 7.58 – 7.46 (m, 2H), 7.42 (dd, *J* = 8.2, 4.2 Hz, 1H), 7.30 (d, *J* = 1.9 Hz, 1H), 7.09 (dd, *J* = 3.5, 1.2 Hz, 1H), 7.03 (dd, *J* = 5.1, 1.1 Hz, 1H), 6.86 – 6.80 (m, 2H), 6.79 – 6.66 (m, 3H), 6.58 (dd, *J* = 7.8, 1.9 Hz, 1H), 4.21 (ddd, *J* = 13.2, 9.3, 5.9 Hz, 1H), 4.05 (ddd, *J* = 12.5, 9.3, 2.9 Hz, 1H), 3.34 – 2.93 (m, 6H) ppm;

^{13}C NMR (101 MHz, $CDCl_3$) δ 165.3 (C_q), 148.0 (CH), 143.7 (C_q), 141.7 (C_q), 139.9 (C_q), 139.5 (C_q), 138.8 (C_q), 137.8 (C_q), 136.6 (CH), 136.2 (CH), 135.6 (CH), 135.5 (CH), 135.2 (C_q), 135.1 (C_q), 133.1 (C_q), 132.3 (CH), 131.7 (CH), 131.6 (CH), 127.9 (C_q), 127.3 (CH), 127.3 (CH), 126.2 (CH), 125.0 (CH), 121.4 (CH), 120.9 (CH), 116.5 (CH), 36.4 (CH_2), 35.2 (CH_2), 35.0 (CH_2), 34.1 (CH_2) ppm;

HRMS (ESI): *m/z* $[M+H]^+$ calcd for $C_{30}H_{24}N_2OS$: 461.1682; found: 461.1685.

General Procedure 2 for the Synthesis of *rac*-1g

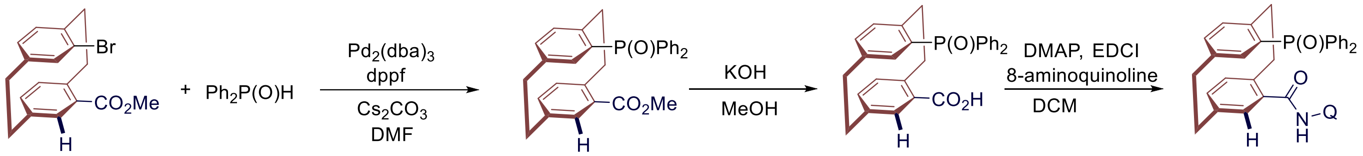

Step 1: Synthesis of *rac*-**S2g**

A mixture of *rac*-**S1** (1.0 mmol), $Ph_2P(O)H$ (2.0 mmol), $Pd_2(dba)_3$ (0.05 mmol), dppf (0.1 mmol), and Cs_2CO_3 (2.0 mmol) in DMF (10 mL) was stirred under a nitrogen atmosphere at 120 °C (oil bath temperature) for 24 h. After cooling to room temperature, the reaction mixture was diluted with ethyl acetate (10 mL) and 1 M HCl (10 mL). The organic layer was separated, and the aqueous phase was extracted with ethyl acetate (3 × 10 mL). The combined organic extracts were dried over anhydrous Na_2SO_4, filtered, and concentrated under reduced pressure. The crude product was purified by column chromatography on silica gel using *n*-hexane /ethyl acetate (3:1 to 1:1) as the eluent to afford *rac*-**S2**.

Step 2: Synthesis of *rac*-**S3g**

rac-**S2** (1.0 mmol) was dissolved in 25% KOH in methanol (10 mL), and the mixture was stirred at 80 °C (oil bath temperature) for 24 h. After cooling to room temperature, the reaction mixture was diluted with ethyl acetate (10 mL), and the pH was adjusted to 2–3 by adding 2 M HCl. The organic layer was

separated, and the aqueous phase was extracted with ethyl acetate (3 × 10 mL). The combined organic extracts were dried over anhydrous Na_2SO_4, filtered, and concentrated under reduced pressure to give crude *rac*-**S3g**, which was used directly in the next step without further purification.

Step 3: Synthesis of *rac*-**1g**

To a solution of *rac*-**S3g** (1.0 mmol) in dry dichloromethane (5 mL) under a nitrogen atmosphere, DMAP (1.0 equiv.) and EDCI (1.2 equiv.) were added. The reaction mixture was stirred at room temperature for 16 h. After completion of the reaction (monitored by TLC), the solvent was removed under reduced pressure, and the residue was purified by column chromatography on silica gel using DCM/MeOH (50:1 to 20:1) as the eluent to afford the desired product *rac*-**1g**.

***rac*-4²-(diphenylphosphoryl)-N-(quinolin-8-yl)-1,4(1,4)-dibenzenacyclohexaphane-1²-carboxamide (*rac*-1g)**

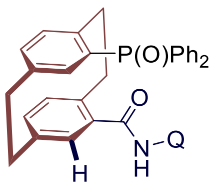

rac-**1g**

Prepared according to general procedure **2** on a 1.0 mmol scale, column chromatography (DCM/MeOH) afforded the compound *rac*-**1g** as a white solid.

¹H NMR (300 MHz, $CDCl_3$) δ 9.96 (s, 1H), 9.17 (dd, J = 7.7, 1.3 Hz, 1H), 8.63 (dd, J = 4.2, 1.7 Hz, 1H), 8.06 (dd, J = 8.3, 1.7 Hz, 1H), 7.72 (ddd, J = 11.7, 8.2, 1.5 Hz, 2H), 7.61 (t, J = 8.0 Hz, 1H), 7.47 – 7.06 (m, 11H), 6.85 – 6.58 (m, 4H), 6.29 (dd, J = 14.6, 1.7 Hz, 1H), 4.39 (ddd, J = 13.1, 9.8, 6.5 Hz, 1H), 3.74 – 3.50 (m, 1H), 3.31 (dd, J = 12.8, 10.2 Hz, 1H), 3.15 (dd, J = 13.7, 10.1 Hz, 2H), 3.00 – 2.63 (m, 3H) ppm;

¹³C NMR (75 MHz, $CDCl_3$) δ 165.4 (C_q), 147.8 (CH), 145.9 (d, J_{CP} = 7.2 Hz, C_q), 139.5 (C_q), 139.4 (C_q), 139.4 (C_q), 139.1 (C_q), 138.8 (C_q), 137.8 (C_q), 136.5 (C_q), 136.3 (CH), 136.2 (CH), 136.0 (CH), 135.9 (CH), 135.6 (CH), 135.6 (CH), 135.5 (C_q), 133.1 (CH), 133.0 (CH), 132.3 (C_q), 131.6 (CH), 131.5 (d, J_{CP} = 2.4 Hz, CH), 131.3 (C_q), 131.14 (d, J_{CP} = 2.8 Hz, CH), 130.9 (d, J_{CP} = 2.6 Hz, CH), 130.5 (C_q), 128.0 (CH), 127.9 (CH), 127.8 (CH), 121.2 (CH), 120.8 (CH), 117.0 (CH), 37.1 (d, J_{CP} = 4.1 Hz, CH_2), 35.0 (CH_2) ppm;

³¹P NMR (121 MHz, $CDCl_3$) δ 26.11 ppm.

HRMS (ESI): *m/z* $[M+H]^+$ calcd for $C_{38}H_{31}N_2O_2P$: 579.2196; found: 579.2195.

The alkenes **2d**-**2l** were synthesized according to literature procedure[2-6].

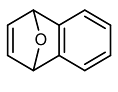

2d

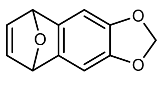

2e

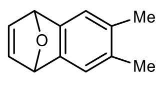

2f

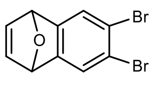

2g

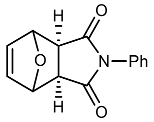

2h

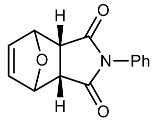

2i

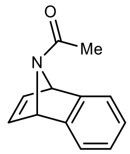

2j

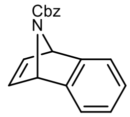

2k

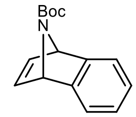

2l

The alkenes **2m-2n** were synthesized according to the following procedure.

***tert*-butyl (*R*)-(1-(2,5-dihydro-1*H*-pyrrol-1-yl)-1-oxopropan-2-yl)carbamate (2m)**

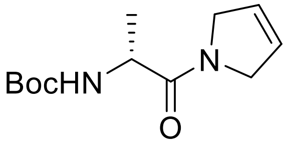

Under nitrogen atmosphere, *N*-Boc-D-alanine (946 mg, 5.00 mmol) was dissolved in 50 mL of dry DCM. Then 2,5-Dihydro-1*H*-pyrrole hydrochloride (528 mg, 5.00 mmol) and EDCI (1.15 g, 6.00 mmol) were added and the reaction was stirred at RT for 20 h. After completion of the reaction, the solvent was removed. Then HCl 1M was added and the water solution was extracted three times with EA. The combined organic phases were dried over anhydrous Na_2SO_4 and the solvent was removed under reduced pressure. Purification by column chromatography (*n*-hexane /EA = 1:1 to 1:2) yielded **2m** (346 mg, 29%) as a white solid.

^{1}H NMR (300 MHz, $CDCl_3$) δ 5.85 – 5.77 (m, 1H), 5.77 – 5.70 (m, 1H), 5.34 (d, *J* = 7.8 Hz, 1H), 4.45 – 4.31 (m, 2H), 4.31 – 4.06 (m, 3H), 1.37 (s, 9H), 1.26 (d, *J* = 6.8 Hz, 3H) ppm;

^{13}C NMR (75 MHz, $CDCl_3$) δ 171.2 (C_q), 155.2 (C_q), 126.0 (CH), 124.8 (CH), 79.5 (C_q), 53.2 (CH_2), 53.0 (CH_2), 47.7 (CH), 28.4 (CH_3), 18.5 (CH_3) ppm.

HRMS (ESI) *m/z* $[M+Na]^+$ calcd for $C_{12}H_{20}N_2O_3Na$: 263.1366; found: 263.1363.

(*S*)-1-(2,5-dihydro-1*H*-pyrrol-1-yl)-2-(4-isobutylphenyl)propan-1-one (2n)

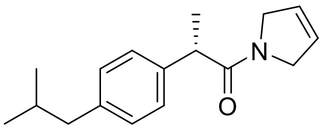

Under nitrogen atmosphere, (*S*)-ibuprofen (1.03 g, 5.00 mmol) was dissolved in 50 mL of dry DCM. Then 2,5-Dihydro-1*H*-pyrrole hydrochloride (528 mg, 5.00 mmol), DMAP (122 mg, 0.2 mmol) and EDCI (1.15 g, 6.00 mmol) were added, and the reaction was stirred at rt for 20 h. After completion of the reaction, the solvent was removed. Then HCl 1M was added and the water solution was extracted three times with EA. The combined organic phases were dried over anhydrous Na_2SO_4 and the solvent was removed under reduced

pressure. Purification by column chromatography (*n*-hexane /EA = 1:1) yielded **2n** (657 mg, 51%) as a transparent oil.

^{1}H NMR (300 MHz, $CDCl_3$) δ 7.25 – 7.19 (m, 2H), 7.13 – 7.06 (m, 2H), 5.89 – 5.79 (m, 1H), 5.72 (m, 1H), 4.40 – 4.14 (m, 3H), 4.01 (d, *J* = 3.1 Hz, 1H), 3.71 (q, *J* = 6.9 Hz, 1H), 2.46 (d, *J* = 7.2 Hz, 2H), 1.86 (d, *J* = 6.7 Hz, 1H), 1.48 (d, *J* = 6.9 Hz, 3H), 0.91 (dd, *J* = 6.6, 0.8 Hz, 6H) ppm;

^{13}C NMR (75 MHz, $CDCl_3$) δ 172.3 (C_q), 140.3 (C_q), 138.5 (C_q) 129.5 (CH), 127.3 (CH), 126.2 (CH), 125.0 (CH), 53.3 (CH_2), 53.0 (CH_2), 45.1 (CH_2), 44.5 (CH), 30.2 (CH), 22.4 (CH_3), 22.4 (CH_3), 20.3 (CH_3) ppm.

HRMS (ESI) *m/z* $[M+Na]^+$ calcd for $C_{17}H_{23}NO$: 280.1672; found: 280.1668

3 Optimization of Reaction Conditions

Table S1. Screening of the reaction parameters.

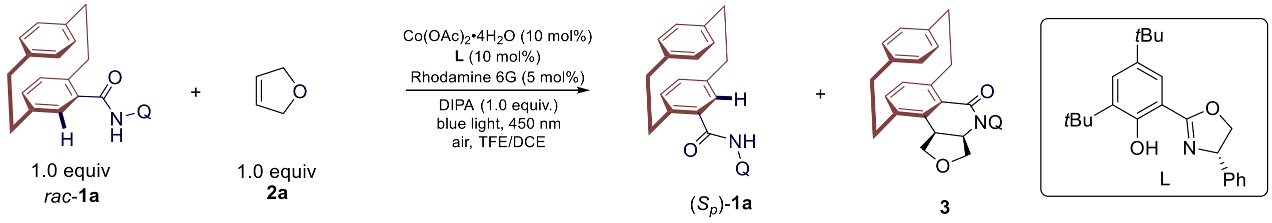

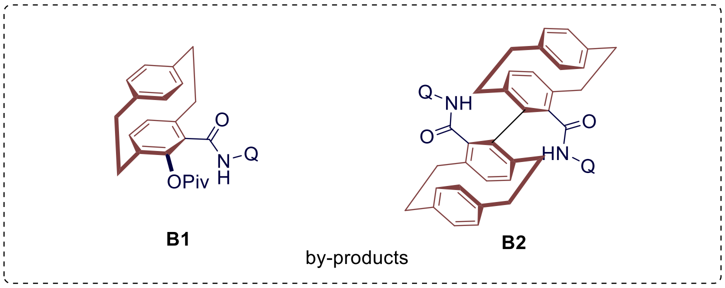

Entry[a]	Deviation from the standard conditions	Yield (S_P)-**1a**[d]	ee (S_P)-**1a**[f]	Yield **3**[d]	dr[e]	ee[f]	s-factor
1	none	50%(49%)	99%	49%(46%)	>20:1	99%	1057
2	TFE/DCE=1:4 instead of 4:1	48%	92%	41%	>20:1	90%	62
3	24h instead of 48h	69%	45%	31%	>20:1	99%	323
4	96h instead of 48h	48%	99%	49%	>20:1	>20:1	1057
5	NaOTf instead of DIPA, 24h	68%	-3%	0%[b]	-	-	-
6	NaOPiv instead of DIPA, 24h	42%	97%	28%[c]	>20:1	90%	80
7	Ag_2CO_3, no blue light and PC, 24h	29%	96%	37%	10:1	96%	194
8	$Mn(OAc)_2 \cdot 4H_2O$, no blue light and PC, 24h	91%	0%	8%	-	0%	-
9	$Mn(OAc)_3 \cdot 2H_2O$, no blue light and PC, 24h	92%	0%	trace	-	-	-

[a]Reaction conditions: **1a** (0.15 mmol, 1.0 equiv.), **2a** (0.15 mmol, 1.0 equiv.), PC (0.0075 mmol, 5 mol%), $Co(OAc)_2 \cdot 4H_2O$ (0.015 mmol, 10 mol%), **L** (0.015 mmol, 10 mol%), base (0.15 mmol, 1.0 equiv.), TFE (1.0 mL), DCE (0.25 mL), 32-35 °C, 24-96 h. [b]Dimer by-product **B2**: 16% yield. [c]Acyloxylation by-product **B1**: 19% yield. [d]Yields were determined by ^{1}H NMR using 1,3,5-trimethoxybenzene as the internal standard; isolated yields after column chromatography are shown in parentheses. [e]The dr value was determined by ^{1}H NMR analysis. [f]The ee value was determined by chiral high-performance liquid chromatography (HPLC) analysis. Q = 8-quinolinyl. PC = photocatalyst. TFE = 2,2,2-trifluoroethanol. DCE = 1,2-dichloroethane. DIPA = diisopropylamine.

Table S2. Control experiments.

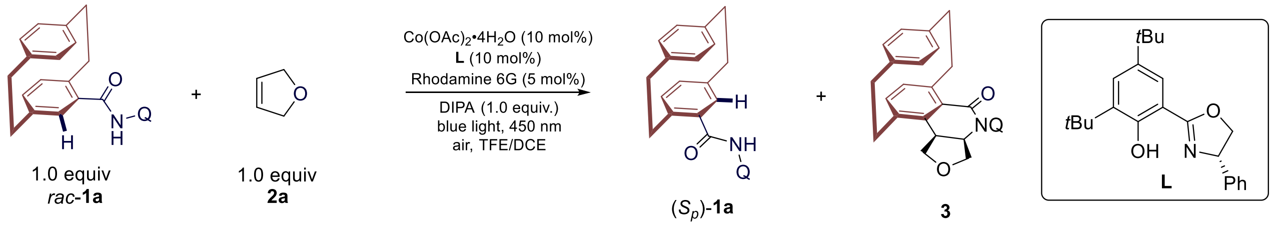

Entry[a]	Deviation from the standard conditions	Yield (S_P)-**1a** [d]	ee (S_P)-**1a** [f]	Yield **3**[d]	dr[e]	ee[f]
1	Without $Co(OAc)_2·4H_2O$	100%	0%	0%		
2	Without **L**	100%	0%	0%		
3	Without Rhodamine 6G	100%	0%	0%		
4	Without blue light	100%	0%	0%		
5	w/o: DIPA, 24h	100%	0%	0%	-	-

[a]Reaction conditions: **1a** (0.15 mmol, 1.0 equiv.), **2a** (0.15 mmol, 1.0 equiv.), PC (0.0075 mmol, 5 mol%), $Co(OAc)_2·4H_2O$ (0.015 mmol, 10 mol%), **L** (0.015 mmol, 10 mol%), base (0.15 mmol, 1.0 equiv.), TFE (1.0 mL), DCE (0.25 mL), 32-35 °C, 24-96 h. [b]dimer by-product **B2**: 16% yield. [c]acyloxylation by-product **B1**: 19% yield. [d]Yields were determined by ^{1}H NMR using 1,3,5-trimethoxybenzene as the internal standard; isolated yields after column chromatography are shown in parentheses. [e]The dr value was determined by ^{1}H NMR analysis. [f]The ee value was determined by chiral high-performance liquid chromatography (HPLC) analysis. Q = 8-quinolinyl. PC = photocatalyst. TFE = 2,2,2-trifluoroethanol. DCE = 1,2-dichloroethane. DIPA = diisopropylamine.

1^3-(quinolin-8-ylcarbamoyl)-1,4(1,4)-dibenzenacyclohexaphane-1^2-yl pivalate (B1)

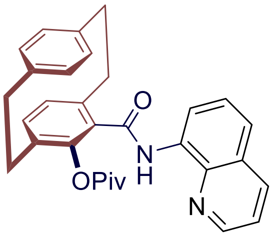

^{1}H NMR (300 MHz, $CDCl_3$) δ 9.68 (s, 1H), 8.93 – 8.77 (m, 1H), 8.67 (dd, *J* = 4.1, 1.7 Hz, 1H), 8.07 (dd, *J* = 8.4, 1.7 Hz, 1H), 7.49 (dt, *J* = 14.4, 8.0 Hz, 2H), 7.34 (dd, *J* = 8.3, 4.1 Hz, 1H), 7.16 (d, *J* = 1.9 Hz, 1H), 6.84 (d, *J* = 8.0 Hz, 1H), 6.68 – 6.44 (m, 4H), 3.36 – 3.15 (m, 2H), 3.08 – 2.83 (m, 5H), 2.80 – 2.65 (m, 1H), 1.11 – 0.96 (m, 9H) ppm;

^{13}C NMR (75 MHz, $CDCl_3$) δ 176.3 (C_q), 164.7 (C_q), 148.3 (CH), 145.9 (C_q), 141.1 (C_q), 139.6 (C_q), 139.3 (C_q), 138.4 (C_q), 136.7 (CH), 136.1 (CH), 134.7 (C_q), 133.2 (CH), 132.5 (C_q), 132.4 (CH), 132.3 (CH), 131.9 (CH), 129.5 (CH), 129.5 (C_q), 127.9 (C_q), 127.4 (CH), 121.7 (CH), 121.7 (CH), 116.3 (CH), 35.2 (CH_2), 34.4 (CH_2), 33.4 (CH_2), 30.8 (CH_2), 29.7 (C_q), 26.9 (CH_3) ppm.

HRMS (ESI) *m/z* $[M+Na]^+$ calcd for $C_{31}H_{30}N_2O_3Na$: 501.2137; found: 501.2149

m.p.: 149-152 °C.

***N*-(quinolin-8-yl)-1^3-(1^3-(quinolin-8-ylcarbamoyl)-1,4(1,4)-dibenzenacyclohexaphane-1^2-yl)-1,4(1,4)-dibenzenacyclohexaphane-1^2-carboxamide (B2)**

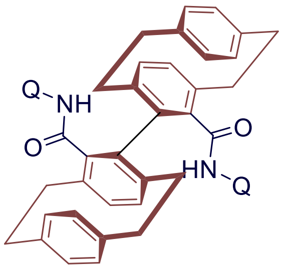

^{1}H NMR (300 MHz, $CDCl_3$) δ 10.24 (d, *J* = 8.6 Hz, 2H), 9.01 (dd, *J* = 7.9, 1.5 Hz, 2H), 8.76 (ddd, *J* = 4.6, 3.7, 1.7 Hz, 2H), 7.84 – 7.74 (m, 2H), 7.60 (dd, *J* = 7.9, 0.8 Hz, 2H), 7.27 (ddt, *J* = 8.5, 4.2, 1.2 Hz, 2H), 6.97 (d, *J* = 1.8 Hz, 2H), 6.87 – 6.82 (m, 2H), 6.70 – 6.47 (m, 10H), 3.96 – 3.82 (m, 2H), 3.46 – 2.71 (m, 14H) ppm;

^{13}C NMR (75 MHz, $CDCl_3$) δ 167.4 (C_q), 167.4 (C_q), 148.4 (CH), 140.5 (C_q), 140.4 (C_q), 140.0 (C_q), 140.0 (C_q), 139.8 (C_q), 139.4 (C_q), 138.7 (C_q), 136.5 (CH), 136.4 (CH), 136.1 (C_q), 136.0 (C_q), 135.5 (CH), 135.1 (C_q), 134.9 (CH), 132.9 (CH), 132.9 (CH), 132.8 (CH), 132.8 (CH), 132.5 (CH), 132.5 (CH), 132.1 (CH), 132.0 (CH), 131.8 (CH), 131.7 (CH), 130.7 (C_q), 129.7 (CH), 127.9 (C_q), 121.8 (CH), 115.8 (CH), 35.6 (CH_2), 35.4 (CH_2), 35.3 (CH_2), 35.1 (CH_2) ppm.

HRMS (ESI) *m/z* $[M+H]^+$ calcd for $C_{52}H_{43}N_4O_2$: 755.3380; found: 755.3399.

4 Experimental

4.1 General Procedure 3 for the Synthesis of Products 3-28

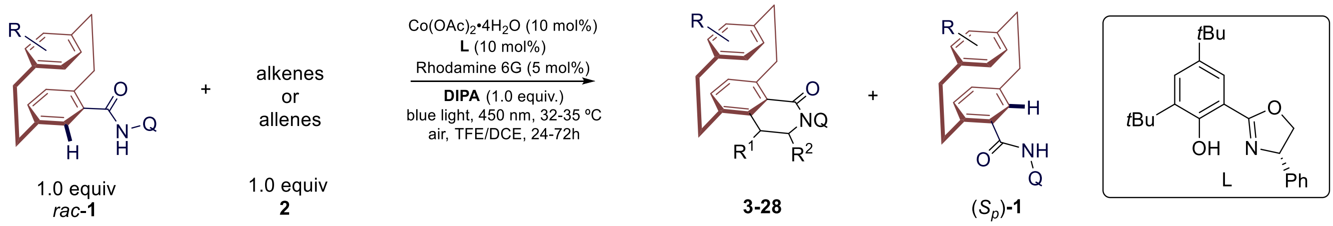

A 10 mL vial was charged with the amide *rac*-**1** (0.15 mmol, 1.0 equiv.), alkenes or allenes **2** (0.15 mmol, 1.0 equiv.), $Co(OAc)_2 \cdot 4H_2O$ (3.7 mg, 10 mol%), **L** (5.3 mg, 10 mol%), Rhodamine 6G (3.6 mg, 5 mol%), DIPA (21 μL, 0.15 mmol, 1.0 equiv.) and a teflon-coated magnetic stirring bar. Then DCE (0.25 mL) and TFE (1 mL) were added. The vial was stirred at room temperature under blue LEDs (450 nm) for 24h-72 h. After completion of the reaction, the solvent was then removed under vacuum and the residue was purified by column chromatography on silica gel with *n*-hexane/ethyl acetate (5:1-1:2) as eluent to give corresponding product **3-28** and (S_p)-**1**. Racemic samples of compounds **3-28** were prepared with racemic **L**.

4.3 General Procedure 4 for the Synthesis 6 and 29 in Continuous Flow Photochemistry

All flow experiments were performed using a self-assembled flow platform: take a 1m long piece of PTFE tubing (outer diameter 1/16", inner diameter 1/32") and wrap it around an 100 mL test tube 25 times.

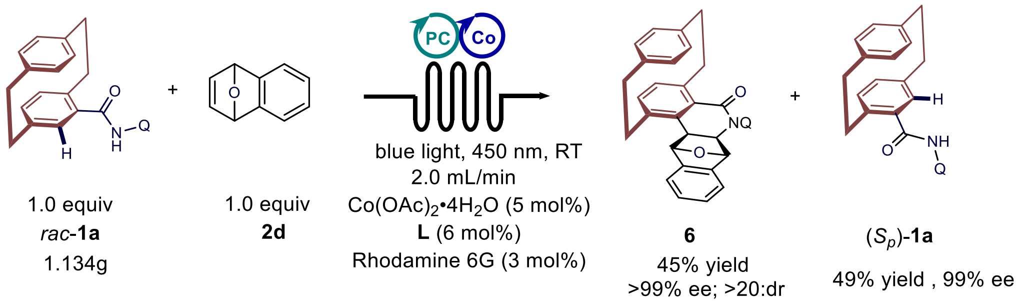

A 100 mL flask was charged with the amide *rac*-**1a** (3.0 mmol, 1.0 equiv.), alkene **2d** (3.0 mmol, 1.0 equiv.), $Co(OAc)_2{\cdot}4H_2O$ (37.4 mg, 5 mol%), **L** (64 mg, 6 mol%), Rhodamine 6G (43 mg, 3 mol%), DIPA (420 µL, 3.0 mmol, 1.0 equiv.) and a teflon-coated magnetic stirring bar. Then DCE (5 mL) and TFE (20 mL) were added. The solution was pumped to the flow reactor by a peristaltic pump with a flow speed of 2.0 mL/min. The reaction was performed at room temperature with blue LEDs (450 nm) for 48 h (**Fig. S1**). After completion of the reaction, the solvent was then removed under vacuum and the residue was purified by column chromatography on silica gel with *n*-hexane/ethyl acetate (1:1) as eluent to give **6** as a buff yellow solid (45% yield, >20:1 dr, 99% ee) and (*Sp*)-**1a** (49% yield, 99% ee).

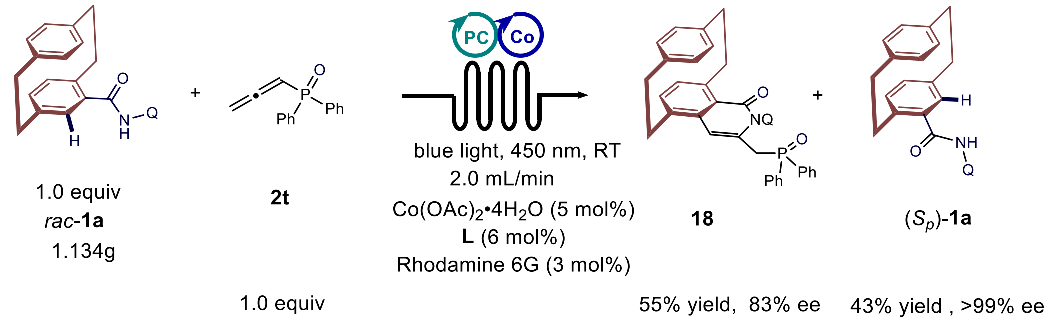

A 100 mL flask was charged with the amide *rac*-**1a** (3.0 mmol, 1.0 equiv.), allene **2t** (3.0 mmol, 1.0 equiv.), $Co(OAc)_2{\cdot}4H_2O$ (37.4 mg, 5 mol%), **L** (64 mg, 6 mol%), Rhodamine 6G (43 mg, 3 mol%), DIPA (420 µL, 3.0 mmol, 1.0 equiv.) and a teflon-coated magnetic stirring bar. Then DCE (5 mL) and TFE (20 mL) were added. The solution was pumped to the flow reactor by a peristaltic pump with a flow speed of 2.0 mL/min. The reaction was performed at room temperature with blue LEDs (450 nm) for 48 h (**Fig. S1**). After completion of the reaction, the solvent was then removed under vacuum and the residue was purified by column chromatography on silica gel with *n*-hexane/ethyl acetate (1:1) as eluent to give **18** as a buff yellow solid (55% yield, 83% ee) and (S_p)-**1a** (43% yield, 99% ee).

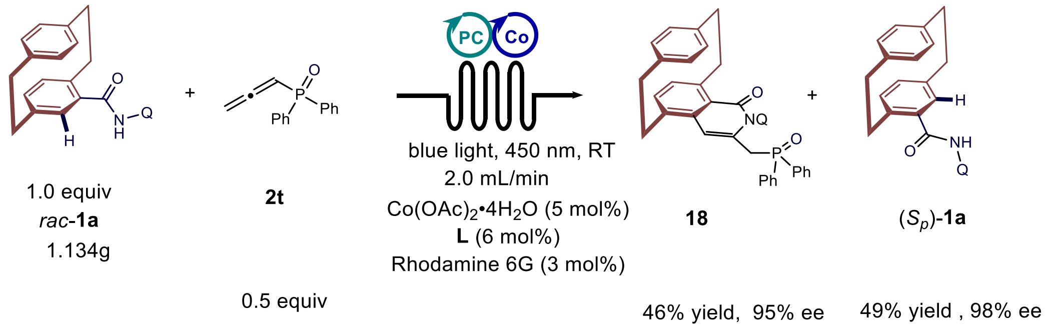

A 100 mL flask was charged with the amide *rac*-**1a** (3.0 mmol, 1.0 equiv.), allene **2t** (1.5 mmol, 0.5 equiv.), $Co(OAc)_2{\cdot}4H_2O$ (37.4 mg, 5 mol%), **L** (64 mg, 6 mol%), Rhodamine 6G (43 mg, 3 mol%), DIPA (420 µL, 3.0 mmol, 1.0 equiv.) and a teflon-coated magnetic stirring bar. Then DCE (5 mL) and TFE (20 mL) were added. The solution was pumped to the flow reactor by a peristaltic pump with a flow speed of 2.0 mL/min. The reaction was performed at room temperature with blue LEDs (450 nm) for 48 h (**Fig. S1**). After completion of the reaction, the solvent was then removed under vacuum and the residue was purified by column chromatography on silica gel with *n*-hexane/ethyl acetate (1:1) as eluent to give **18** as a buff yellow solid (46% yield, 95% ee) and (S_p)-**1a** (49% yield, 98% ee).

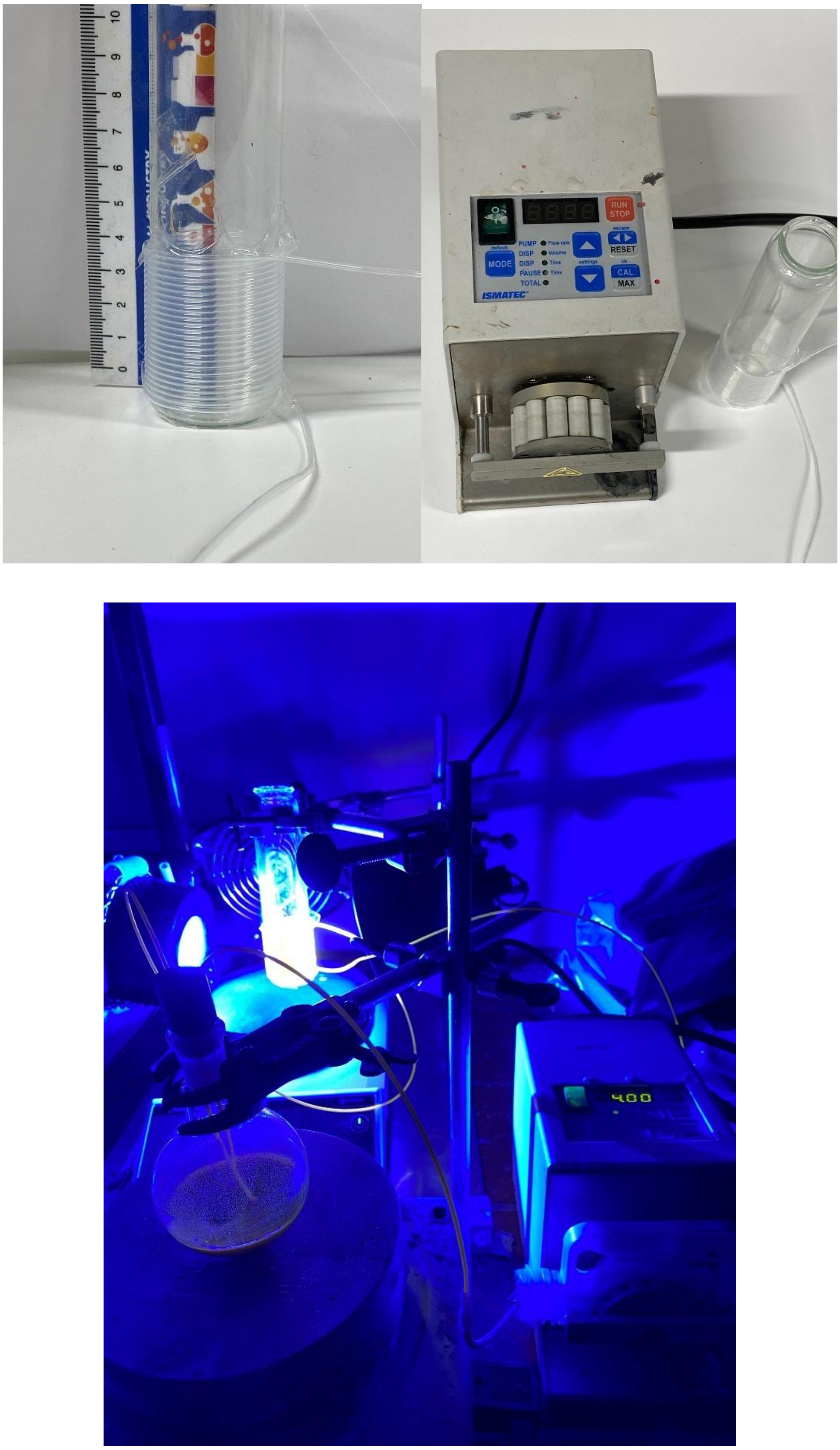

Figure S1. Pictures of the flow photochemical set up used.

Characterization data

($1^{3a}S,1^{9b}R$)-1^4-(quinolin-8-yl)-$1^1,1^3,1^{3a},1^4,1^5,1^{9b}$-hexahydro-1(6,9)-furo[3,4-*c*]isoquinolina-4(1,4)-benzenacyclohexaphan-1^5-one (3)

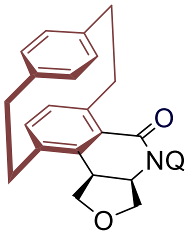

Prepared according to general procedure **3** for 48h on a 0.15 mmol scale, column chromatography (ethyl acetate/*n*-hexane = 1:3 to 1:1) afforded the compound **3** as a white solid (31 mg, 0.069 mmol, 46%), with >20:1 dr, 99% ee and chiral starting material (S_p)-**1a** as a white solid (36 mg, 0.074 mmol, 49%), with 99% ee.

^{1}H NMR (400 MHz, $CDCl_3$, major rotamer) δ 8.74 (dd, *J* = 4.3, 1.7 Hz, 1H), 8.18 (dd, *J* = 8.4, 1.8 Hz, 1H), 7.88 (ddd, *J* = 11.9, 7.8, 1.5 Hz, 2H), 7.70 (t, *J* = 7.7 Hz, 1H), 7.36 (dd, *J* = 8.3, 4.2 Hz, 1H), 6.74 (dd, *J* = 7.9, 1.8 Hz, 1H), 6.71 – 6.61 (m, 2H), 6.59 – 6.48 (m, 2H), 6.31 – 6.21 (m, 1H), 4.90 (t, *J* = 3.4 Hz, 1H), 4.73 (dd, *J* = 11.2, 7.3 Hz, 1H), 4.61 (t, *J* = 7.7 Hz, 1H), 4.40 (ddd, *J* = 11.7, 9.8, 1.4 Hz, 1H), 3.70 – 3.53 (m, 3H), 3.35 – 3.00 (m, 6H), 2.78 (ddd, *J* = 12.2, 9.8, 7.5 Hz, 1H) ppm;

^{13}C NMR (101 MHz, $CDCl_3$, major rotamer) δ 165.2 (C_q), 150.4 (CH), 144.6 (C_q), 143.8 (C_q), 141.4 (C_q), 138.5 (C_q), 137.7 (C_q), 136.8 (CH), 136.7 (CH), 136.4 (CH), 136.3 (CH), 134.6 (C_q), 133.0 (CH), 132.4 (CH), 132.2 (CH), 131.7 (CH), 131.3 (CH), 129.7 (C_q), 127.9 (CH), 126.5 (CH), 126.4 (C_q), 121.4 (CH), 71.9 (CH_2), 71.4 (CH_2), 61.3 (CH), 41.7 (CH), 36.6 (CH_2), 35.5 (CH_2), 35.0 (CH_2), 31.8 (CH_2) ppm; (one quarter carbon is missing) Because of the presence of conformers with high energy barriers, **^{13}C NMR** analysis shows the signals of major rotamer.

HRMS (ESI): *m/z* $[M+H]^+$ calcd for $C_{30}H_{26}N_2O_2$: 447.2067; found: 447.2067.

$[\alpha]^D_{20}$ = +7.2 (c = 0.50, $CHCl_3$).

R_t (AD-3 column, *n*-hexane/*i*-PrOH 80/20, 1.0 mL/min, 273.0 nm): tr(major) = 10.9 min, tr(minor) = 8.6 min, 99% ee.

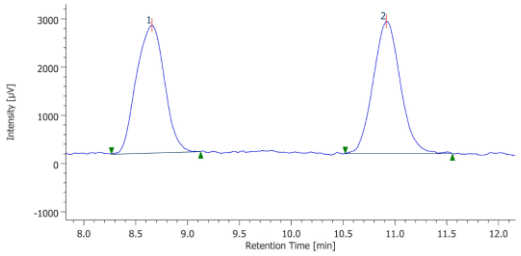

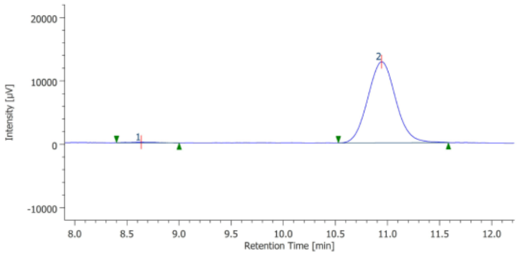

#	Peak Name	CH	tR [min]	Area [µV·sec]	Height [µV]	Area%
1	Unknown	10	8.657	49781	2652	49.822
2	Unknown	10	10.917	50137	2745	50.178

#	Peak Name	CH	tR [min]	Area [µV·sec]	Height [µV]	Area%
1	Unknown	10	8.637	1263	89	0.530
2	Unknown	10	10.943	237214	12781	99.470

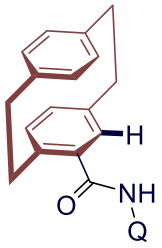

S$_p$-**1**

^{1}H NMR (400 MHz, $CDCl_3$) δ 10.18 (s, 1H), 8.98 (dd, *J* = 7.6, 1.4 Hz, 1H), 8.81 (dd, *J* = 4.2, 1.7 Hz, 1H), 8.19 (dd, *J* = 8.3, 1.7 Hz, 1H), 7.69 – 7.60 (m, 1H), 7.56 (dd, *J* = 8.3, 1.4 Hz, 1H), 7.46 (dd, *J* = 8.2, 4.2 Hz, 1H), 6.99 (d, *J* = 1.9 Hz, 1H), 6.96 – 6.81 (m, 1H), 6.77 – 6.55 (m, 5H), 3.92 (ddd, *J* = 12.8, 10.0, 2.5 Hz, 1H), 3.37 – 2.87 (m, 7H) ppm;

^{13}C NMR (101 MHz, $CDCl_3$) δ 167.3 (C_q), 148.3 (CH), 140.4 (C_q), 140.0 (C_q), 139.7 (C_q), 139.4 (C_q), 138.8 (C_q), 136.4 (CH), 136.4 (CH), 136.1 (C_q), 135.4 (CH), 135.1 (C_q), 133.0 (CH), 132.9 (CH), 132.7 (CH), 132.5 (CH), 131.9 (CH), 131.7 (CH), 128.1 (C_q), 127.5 (CH), 121.7 (CH), 121.5 (CH), 116.3 (CH), 35.5 (CH_2), 35.4 (CH_2), 35.3 (CH_2), 35.0 (CH_2) ppm.

$[\alpha]^D{}_{20}$ = -52.0 (c = 0.50, $CHCl_3$).

R_t (AD-3 column, *n*-hexane/*i*-PrOH 80/20, 1.0 mL/min, 250.0 nm): tr(major) = 10.9 min, tr(minor) = 8.7 min, 99% ee.

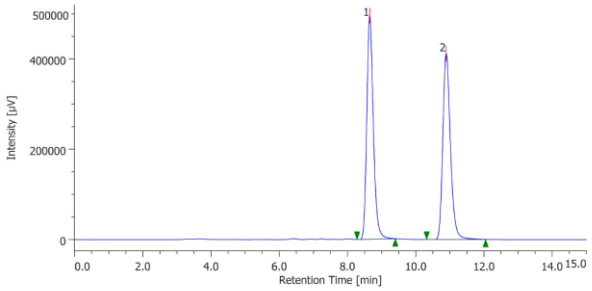

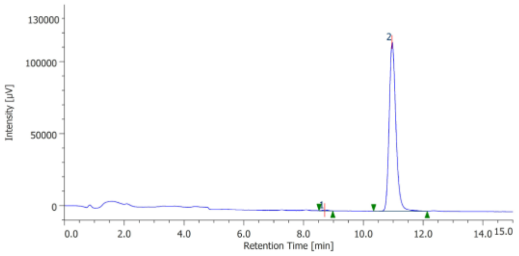

#	Peak Name	CH	tR [min]	Area [µV·sec]	Height [µV]	Area%
1	Unknown	9	8.647	6604988	494593	50.777
2	Unknown	9	10.887	6402949	412098	49.223

#	Peak Name	CH	tR [min]	Area [µV·sec]	Height [µV]	Area%
1	Unknown	9	8.703	5726	492	0.312
2	Unknown	9	10.960	1829594	117313	99.688

tert-butyl **($1^{3a}R,1^{9b}R$)-1^5-oxo-1^4-(quinolin-8-yl)-$1^2,1^3,1^{3a},1^4,1^5,1^{9b}$-hexahydro-$1^1H$-1(6,9)-pyrrolo[3,4-*c*]isoquinolina-4(1,4)-benzenacyclohexaphane-1^2-carboxylate (4)**

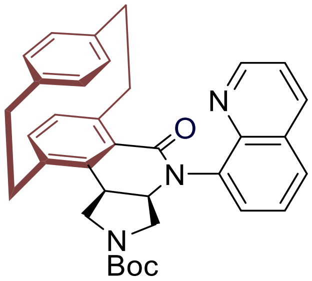

Prepared according to general procedure **3** for 72h on a 0.15 mmol scale, column chromatography (ethyl acetate/*n*-hexane = 1:3 to 1:1) afforded the compound **4** as a white solid (35 mg, 0.065 mmol, 43%), with >20:1 dr, 99% ee and chiral starting material (S_p)-**1a** as a white solid (26 mg, 0.069 mmol, 46%), with 99% ee.

^{1}H NMR (500 MHz, $CDCl_3$, major rotamer) δ 8.72 (s, 1H), 8.15 (s, 1H), 7.84 (s, 1H), 7.67 (d, *J* = 39.6 Hz, 2H), 7.37 (d, *J* = 35.8 Hz, 1H), 6.86 – 6.35 (m, 6H), 4.75 (s, 1H), 4.46 – 3.90 (m, 3H), 3.48 (s, 1H), 3.31 – 2.93 (m, 8H), 2.75 (q, *J* = 9.9 Hz, 1H), 1.37 (m, 9H) ppm;

^{13}C NMR (126 MHz, $CDCl_3$, major rotamer): 165.4 (C_q), 150.2 (CH), 144.9 (C_q), 143.7 (C_q), 141.2 (C_q), 138.4 (C_q), 137.5 (C_q), 136.7 (CH), 136.3 (CH), 136.2 (CH), 134.8 (C_q), 132.8 (CH), 132.2 (CH), 131.6 (CH), 131.2 (CH), 129.6 (C_q), 127.8 (CH), 126.6 (C_q), 126.2 (CH), 121.2 (CH), 79.5 (C_q), 60.2 (CH), 50.7 (CH_2), 49.2 (CH_2), 36.3 (CH_2), 35.4 (CH_2), 35.0 (CH_2), 31.6 (CH_2), 28.4 (CH_3) ppm. Two C_q and two CH are missing, probably overlapped. Because of the presence of conformers with high energy barriers, **^{13}C NMR** analysis shows the signals of major rotamer.

HRMS (ESI): *m/z* $[M+H]^+$ calcd for $C_{35}H_{35}N_3O_3$: 546.2751; found: 546.2752.

R_t (OD-3 column, *n*-hexane/*i*-PrOH 60/40, 1.0 mL/min, 273.0 nm): tr(major) = 10.2 min, tr(minor) = 41.1 min, 99% ee.

$[\alpha]^D_{20}$ = -31.8 (c = 0.50, $CHCl_3$).

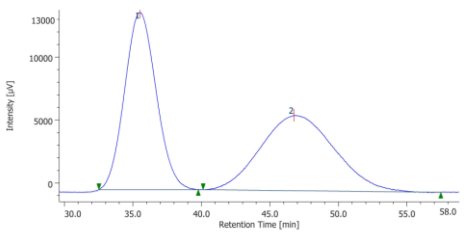

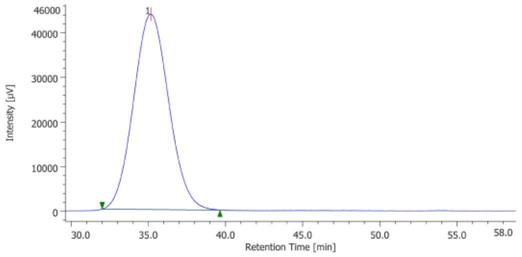

#	Peak Name	CH	tR [min]	Area [µV·sec]	Height [µV]	Area%
1	Unknown	10	35.523	2253267	14062	50.230
2	Unknown	10	46.747	2232675	5975	49.770

#	Peak Name	CH	tR [min]	Area [µV·sec]	Height [µV]	Area%
1	Unknown	10	35.160	6942729	43699	100.000

benzyl (1^{3a}*R*,1^{9b}*R*)-1^{5}-oxo-1^{4}-(quinolin-8-yl)-1^{2},1^{3},1^{3a},1^{4},1^{5},1^{9b}-hexahydro-1^{1}*H*-1(6,9)-pyrrolo[3,4-*c*]isoquinolina-4(1,4)-benzenacyclohexaphane-1^{2}-carboxylate (5)

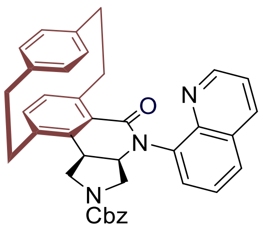

Prepared according to general procedure **3** for 72h on a 0.15 mmol scale, column chromatography (ethyl acetate/*n*-hexane = 1:2) afforded the compound **5** as a white solid (40 mg, 0.069 mmol, 46%), with >20:1 dr, 99% ee and chiral starting material (S_p)-**1a** as a white solid (27 mg, 0.072 mmol, 48%), with 99% ee.

^{1}H NMR (400 MHz, $CDCl_3$, major rotamer) δ 8.73 (dd, *J* = 4.2, 1.8 Hz, 1H), 8.29 – 8.12 (m, 1H), 7.89 – 7.78 (m, 1H), 7.63 – 7.29 (m, 7H), 7.25 – 7.13 (m, 1H), 6.78 – 6.60 (m, 4H), 6.60 – 6.50 (m, 2H), 5.39 – 4.96 (m, 2H), 4.89 – 4.80 (m, 1H), 4.41 – 4.24 (m, 3H), 3.65 – 3.43 (m, 1H), 3.39 – 2.99 (m, 8H), 2.87 – 2.71 (m, 1H) ppm;

^{13}C NMR (101 MHz, $CDCl_3$, including two rotamers) δ 165.6 (C_q), 165.4 (C_q), 164.4 (C_q), 164.4 (C_q), 154.8 (C_q), 154.6 (C_q), 154.3 (C_q), 154.1 (C_q), 151.0 (CH), 150.4 (CH), 150.3 (CH), 146.4 (C_q), 146.2 (C_q), 144.7 (C_q), 144.6 (C_q), 143.8 (C_q), 143.6 (C_q), 143.6 (C_q), 141.4 (C_q), 141.4 (C_q), 141.3 (C_q), 141.2 (C_q), 138.6 (C_q), 138.5 (C_q), 138.5 (C_q), 138.4 (C_q), 138.2 (C_q), 138.1 (C_q), 138.0 (C_q), 137.8 (C_q), 137.7 (C_q), 137.5 (C_q), 137.1 (C_q), 137.0 (C_q), 136.9 (CH), 136.8 (CH), 136.8 (CH), 136.7 (C_q), 136.6 (CH), 136.5 (C_q), 136.4 (CH), 136.3 (CH), 136.2 (C_q), 135.8 (C_q), 135.0 (C_q), 134.9 (C_q), 134.6 (C_q), 134.5 (C_q), 133.1 (CH), 133.0 (CH), 132.9 (CH), 132.4 (CH), 132.3 (CH), 132.2 (CH), 132.1 (CH), 132.0 (CH), 131.8 (CH), 131.7 (CH), 131.5 (CH), 131.5 (CH), 131.4 (CH), 131.3 (CH), 131.2 (CH), 129.7 (C_q), 129.6 (C_q), 129.3 (C_q), 129.0 (C_q), 128.7 (CH), 128.6 (CH), 128.5 (CH), 128.2 (CH), 128.0 (CH), 128.0 (CH), 127.9 (CH), 127.9 (CH), 127.7 (CH), 127.7 (CH), 127.4 (CH), 126.7, 126.6 (CH), 126.5, 126.3 (CH), 126.3 (CH), 121.8 (CH), 121.7 (CH), 121.4 (CH), 67.0 (CH_2), 66.8 (CH_2), 66.5 (CH_2), 66.2 (CH_2), 60.2 (CH_2), 60.1 (CH_2), 59.5 (CH_2), 59.5 (CH_2), 50.9 (CH_2), 50.5 (CH_2), 50.4 (CH_2), 50.2 (CH_2), 49.9 (CH_2), 49.7 (CH_2), 49.4 (CH_2), 41.6 (CH), 40.8 (CH), 40.6 (CH), 39.9 (CH), 36.7 (CH_2), 36.7 (CH_2), 35.5 (CH_2), 35.4 (CH_2), 35.4 (CH_2), 35.4 (CH_2), 35.0 (CH_2), 31.7 (CH_2), 31.6 (CH_2) ppm; Because of the presence of conformers with high energy barriers, ^{13}C-NMR analysis shows the signals of both.

HRMS (ESI): *m/z* $[M+H]^+$ calcd for $C_{38}H_{33}N_3O_3$: 580.2595; found: 580.2618.

$[\alpha]^{D}_{20}$ = -5.6 (c = 0.50, $CHCl_3$).

R_t (OD-3 column, *n*-hexane/*i*-PrOH 60/40, 1.0 mL/min, 273.0 nm): tr(major) = 10.2 min, tr(minor) = 41.1 min, 99% ee.

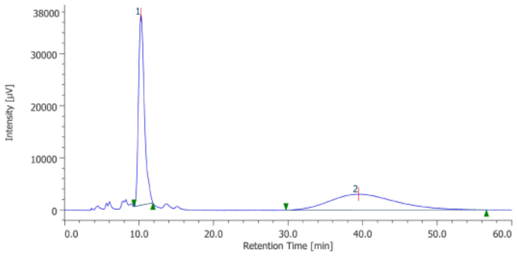

#	Peak Name	CH	tR [min]	Area [μV·sec]	Height [μV]	Area%
1	Unknown	10	10.247	1995075	36559	51.736
2	Unknown	10	39.443	1861217	3051	48.264

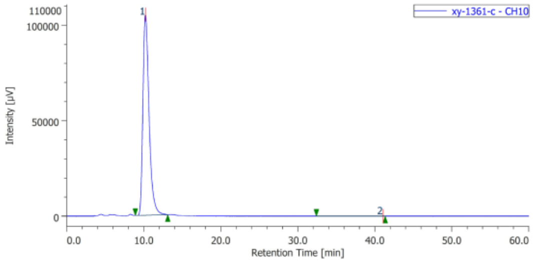

#	Peak Name	CH	tR [min]	Area [μV·sec]	Height [μV]	Area%
1	Unknown	10	10.203	5829406	104843	99.978
2	Unknown	10	41.080	1288	10	0.022

(1^{6a}*R*,1^{7}*S*,1^{12}*S*,1^{12a}*S*)-1^{6}-(quinolin-8-yl)-1^{5},1^{6},1^{6a},1^{7},1^{12},1^{12a}-hexahydro-1(1,4)-7,12-epoxybenzo[*b*]phenanthridina-4(1,4)-benzenacyclohexaphan-1^{5}-one (6)

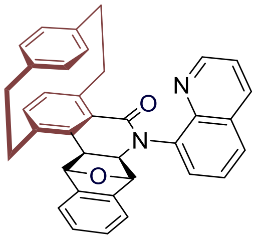

Prepared according to general procedure **3** for 24h on a 0.15 mmol scale, column chromatography (ethyl acetate/*n*-hexane = 1:2) afforded the compound **6** as a white solid (38 mg, 0.072 mmol, 48%), with >20:1 dr, 97% ee and chiral starting material (S_p)-**1a** as a white solid (28 mg, 0.0735 mmol, 49%), with 99% ee.

^{1}H NMR (400 MHz, $CDCl_3$) δ 8.73 (dd, *J* = 4.2, 1.7 Hz, 1H), 8.22 (dd, *J* = 8.3, 1.7 Hz, 1H), 8.05 (dd, *J* = 7.3, 1.4 Hz, 1H), 7.93 (dd, *J* = 8.3, 1.4 Hz, 1H), 7.77 (dd, *J* = 8.3, 7.3 Hz, 1H), 7.48 (dd, *J* = 7.3, 0.9 Hz, 1H), 7.37 (dd, *J* = 8.3, 4.1 Hz, 1H), 7.28 – 7.20 (m, 1H), 7.10 (td, *J* = 7.5, 1.0 Hz, 1H), 6.98 (d, *J* = 7.3 Hz, 1H), 6.86 – 6.66 (m, 4H), 6.60 (d, *J* = 7.6 Hz, 1H), 6.51 (d, *J* = 7.5 Hz, 1H), 6.34 (s, 1H), 4.66 – 4.52 (m, 2H), 3.52 (ddd, *J* = 13.7, 10.0, 4.2 Hz, 1H), 3.42 – 3.21 (m, 5H), 3.12 (ddd, *J* = 12.7, 9.8, 2.7 Hz, 1H), 2.97 – 2.85 (m, 1H) ppm;

^{13}C NMR (101 MHz, $CDCl_3$) δ 164.0 (C_q), 150.5 (CH), 147.8 (C_q), 144.7 (C_q), 144.1 (C_q), 141.7 (C_q), 141.6 (C_q), 138.2 (C_q), 137.7 (C_q), 137.6 (CH), 137.3 (C_q), 136.5 (CH), 136.1 (CH), 133.3 (C_q), 132.9 (CH), 132.8 (CH), 132.3 (CH), 131.3 (CH), 130.0 (C_q), 128.2 (CH), 127.6 (CH), 127.1 (CH), 126.5 (CH), 125.9 (C_q), 121.4 (CH), 121.1 (CH), 118.2 (CH), 83.8 (CH), 83.2 (CH), 61.6 (CH), 42.3 (CH), 36.5 (CH_2), 35.7 (CH_2), 35.0 (CH_2), 32.7 (CH_2) ppm;

HRMS (ESI): *m/z* $[M+H]^+$ calcd for $C_{36}H_{28}N_2O_2$: 521.2224; found: 521.2224.

$[\alpha]^{D}_{20}$ = +110.8 (c = 0.50, $CHCl_3$).

R_t (AD-3 column, *n*-hexane/*i*-PrOH 70/30, 1.0 mL/min, 273.0 nm): tr(major) = 9.2 min, tr(minor) = 21.4 min, 97% ee.

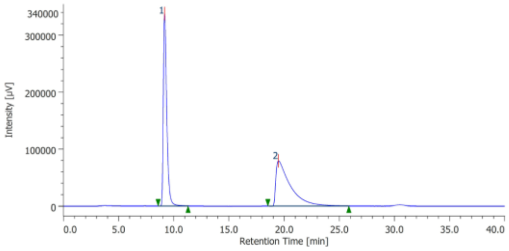

#	Peak Name	CH	tR [min]	Area [µV·sec]	Height [µV]	Area%
1	Unknown	10	9.170	7260817	337435	50.773
2	Unknown	10	19.477	7039597	79125	49.227

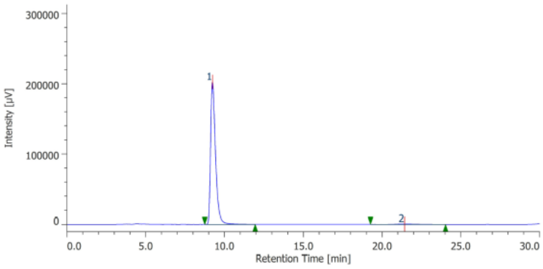

#	Peak Name	CH	tR [min]	Area [µV·sec]	Height [µV]	Area%
1	Unknown	10	9.237	4390048	202542	98.645
2	Unknown	10	21.420	60312	661	1.355

(1^{6a}*R*,1^{7}*S*,1^{13}*S*,1^{13a}*S*)-1^{6}-(quinolin-8-yl)-1^{5},1^{6},1^{6a},1^{7},1^{13},1^{13a}-hexahydro-1(1,4)-7,13-epoxy[1,3]dioxolo[4',5':4,5]benzo[1,2-*b*]phenanthridina-4(1,4)-benzenacyclohexaphan-1^{5}-one (7)

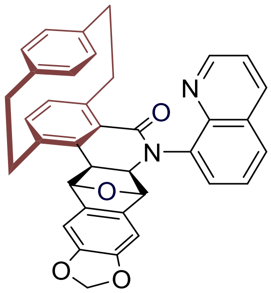

Prepared according to general procedure **3** for 48h on a 0.15 mmol scale, column chromatography (ethyl acetate/*n*-hexane = 1:2) afforded the compound **7** as a white solid (38 mg, 0.0675 mmol, 45%), with >20:1 dr, 93% ee and chiral starting material (S_p)-**1a** as a white solid (26 mg, 0.0675 mmol, 45%), with 99% ee.

^{1}H NMR (400 MHz, $CDCl_3$) δ 8.74 (dd, *J* = 4.2, 1.8 Hz, 1H), 8.21 (dd, *J* = 8.3, 1.8 Hz, 1H), 8.02 (dd, *J* = 7.3, 1.4 Hz, 1H), 7.92 (dd, *J* = 8.3, 1.5 Hz, 1H), 7.76 (dd, *J* = 8.3, 7.3 Hz, 1H), 7.37 (dd, *J* = 8.3, 4.1 Hz, 1H), 6.99 (s, 1H), 6.81 – 6.67 (m, 4H), 6.59 (d, *J* = 7.5 Hz, 1H), 6.51 (d, *J* = 7.6 Hz, 1H), 6.46 (s, 1H), 6.24 (s, 1H), 5.95 (d, *J* = 1.4 Hz, 1H), 5.89 (d, *J* = 1.4 Hz, 1H), 5.31 (s, 1H), 4.77 – 4.28 (m, 2H), 3.53 – 3.42 (m, 1H), 3.40 – 3.19 (m, 5H), 3.11 (ddd, *J* = 12.7, 9.8, 2.7 Hz, 1H), 2.96 – 2.79 (m, 1H) ppm;

^{13}C NMR (101 MHz, $CDCl_3$) δ 163.9 (C_q), 150.5 (CH), 147.2 (C_q), 146.8 (C_q), 144.7 (C_q), 141.6 (C_q), 141.4 (C_q), 137.6 (C_q), 137.6 (CH), 137.3 (C_q), 136.5 (CH), 136.1 (CH), 135.2 (C_q), 133.3 (C_q), 132.9 (CH), 132.8 (CH), 132.3 (CH), 131.3 (CH), 130.0 (C_q), 128.1 (CH), 126.5 (CH), 125.9 (C_q), 121.4 (CH), 103.2 (CH), 101.4 (C_q), 100.7 (CH), 83.8 (CH), 83.3 (CH), 61.7 (CH), 42.5 (CH), 36.5 (CH_2), 35.7 (CH_2), 35.0 (CH_2), 32.7 (CH_2) ppm;

HRMS (ESI): *m/z* $[M+H]^+$ calcd for $C_{37}H_{28}N_2O_4$: 565.2122; found: 565.2122.

$[\alpha]^{D}_{20}$ = +109.6 (c = 0.50, $CHCl_3$).

R_t (OD-3 column, *n*-hexane/*i*-PrOH 93/7, 1.0 mL/min, 273.0 nm): tr(major) = 106.8 min, tr(minor) = 87.8 min, 95% ee.

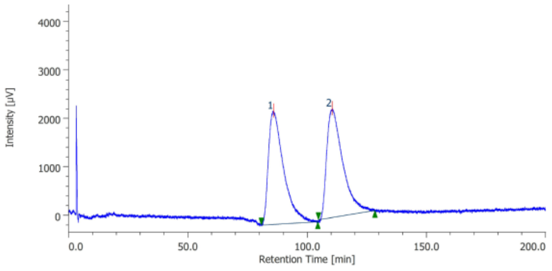

#	Peak Name	CH	tR [min]	Area [µV·sec]	Height [µV]	Area%
1	Unknown	10	85.990	1010727	2346	49.130
2	Unknown	10	110.423	1046532	2256	50.870

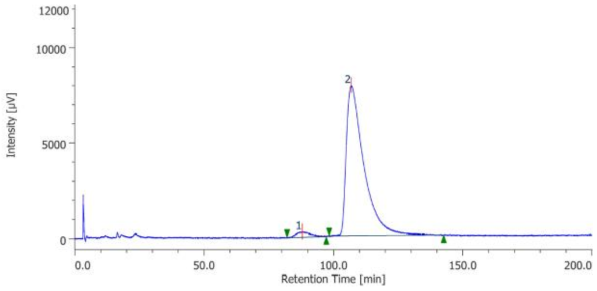

#	Peak Name	CH	tR [min]	Area [µV·sec]	Height [µV]	Area%
1	Unknown	10	87.843	97067	284	2.576
2	Unknown	10	106.830	3671417	7864	97.424

($1^{6a}R,1^7S,1^{12}S,1^{12a}S$)-$1^9,1^{10}$-dimethyl-$1^6$-(quinolin-8-yl)-$1^5,1^6,1^{6a},1^7,1^{12},1^{12a}$-hexahydro-1(1,4)-7,12-epoxybenzo[*b*]phenanthridina-4(1,4)-benzenacyclohexaphan-1^5-one (8)

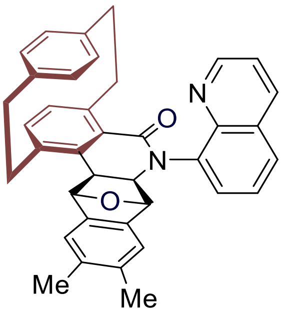

Prepared according to general procedure **3** for 24h on a 0.15 mmol scale, column chromatography (ethyl acetate/*n*-hexane = 1:2) afforded the compound **8** as a white solid (37 mg, 0.0675 mmol, 45%), with >20:1 dr, 99% ee and chiral starting material (S_P)-**1a** as a white solid (26 mg, 0.069 mmol, 46%), with 99% ee.

^{1}H NMR (400 MHz, $CDCl_3$) δ 8.73 (dd, *J* = 4.3, 1.8 Hz, 1H), 8.21 (dd, *J* = 8.4, 1.8 Hz, 1H), 8.07 – 7.98 (m, 1H), 7.92 (dd, *J* = 8.3, 1.5 Hz, 1H), 7.81 – 7.70 (m, 1H), 7.36 (dd, *J* = 8.3, 4.1 Hz, 1H), 6.84 – 6.68 (m, 5H), 6.59 (d, *J* = 7.6 Hz, 1H), 6.51 (d, *J* = 7.6 Hz, 1H), 6.28 (s, 1H), 5.35 (s, 1H), 4.66 – 4.47 (m, 2H), 3.52 (ddd, *J* = 13.6, 10.1, 4.0 Hz, 1H), 3.42 – 3.21 (m, 5H), 3.11 (ddd, *J* = 12.7, 9.8, 2.7 Hz, 1H), 2.90 (ddd, *J* = 12.8, 9.8, 6.4 Hz, 1H), 2.28 (s, 3H), 2.14 (s, 3H) ppm;

^{13}C NMR (101 MHz, $CDCl_3$) δ 163.9 (C_q), 150.5 (CH), 145.6 (C_q), 144.6 (C_q), 144.1 (C_q), 141.5 (C_q), 139.4 (C_q), 138.2 (C_q), 137.7 (C_q), 137.5 (CH), 137.3 (C_q), 136.4 (CH), 136.0 (CH), 135.7 (C_q), 135.1 (C_q), 133.5 (C_q), 132.8 (CH), 132.7 (CH), 132.3 (CH), 131.3 (CH), 130.0 (C_q), 128.0 (CH), 126.4 (CH), 125.8 (C_q), 122.3 (CH), 121.4 (CH), 119.5 (CH), 83.6 (CH), 83.0 (CH), 61.9 (CH), 42.7 (CH), 36.4 (CH_2), 35.7 (CH_2), 35.0 (CH_2), 32.7 (CH_2), 20.0 (CH_3), 19.9 (CH_3) ppm;

HRMS (ESI): *m/z* $[M+H]^+$ calcd for $C_{38}H_{32}N_2O_2$: 549.2537; found: 549.2536.

$[\alpha]^D{}_{20}$ = +128.2 (c = 0.50, $CHCl_3$).

R_t (AD-3 column, *n*-hexane/*i*-PrOH 60/40, 1.0 mL/min, 273.0 nm): tr(major) = 6.6 min, tr(minor) = 19.4 min, 99% ee.

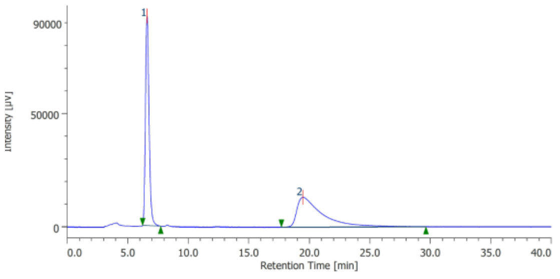

#	Peak Name	CH	tR [min]	Area [µV·sec]	Height [µV]	Area%
1	Unknown	10	6.593	1947893	92441	50.895
2	Unknown	10	19.447	1879352	13109	49.105

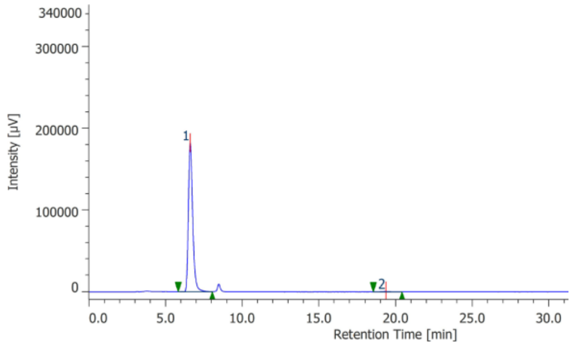

#	Peak Name	CH	tR [min]	Area [µV·sec]	Height [µV]	Area%
1	Unknown	10	6.610	3575506	181865	99.370
2	Unknown	10	19.357	22686	501	0.630

(1^{6a}*R*,1^{7}*S*,1^{12}*S*,1^{12a}*S*)-1^{9},1^{10}-dibromo-1^{6}-(quinolin-8-yl)-1^{5},1^{6},1^{6a},1^{7},1^{12},1^{12a}-hexahydro-1(1,4)-7,12-epoxybenzo[*b*]phenanthridina-4(1,4)-benzenacyclohexaphan-1^{5}-one (9)

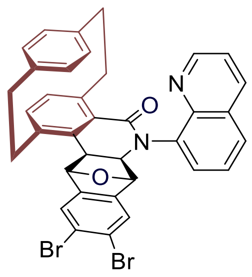

Prepared according to general procedure **3** for 48h on a 0.15 mmol scale, column chromatography (ethyl acetate/*n*-hexane = 1:2) afforded the compound **9** as a white solid (45 mg, 0.0676 mmol, 44%), with >20:1 dr, 99% ee and chiral starting material (S_P)-**1a** as a white solid (26 mg, 0.0675 mmol, 45%), with 99% ee.

^{1}H NMR (400 MHz, $CDCl_3$) δ 8.75 (dd, *J* = 4.2, 1.8 Hz, 1H), 8.23 (dd, *J* = 8.3, 1.8 Hz, 1H), 7.99 (dd, *J* = 7.2, 1.4 Hz, 1H), 7.93 (dd, *J* = 8.3, 1.4 Hz, 1H), 7.81 – 7.65 (m, 2H), 7.39 (dd, *J* = 8.3, 4.2 Hz, 1H), 7.25 (s, 1H), 6.81 – 6.68 (m, 4H), 6.61 (d, *J* = 7.6 Hz, 1H), 6.53 (d, *J* = 7.6 Hz, 1H), 6.29 (s, 1H), 5.36 (s, 1H), 4.65 (d, *J* = 8.4 Hz, 1H), 4.55 (ddd, *J* = 12.6, 9.8, 2.7 Hz, 1H), 3.52 – 3.04 (m, 7H), 2.89 (ddd, *J* = 12.8, 9.7, 6.6 Hz, 1H) ppm;

^{13}C NMR (101 MHz, $CDCl_3$) δ 163.7 (C_q), 150.6 (CH), 148.6 (C_q), 144.8 (C_q), 144.0 (C_q), 142.8 (C_q), 141.6 (C_q), 137.7 (CH), 137.5 (C_q), 137.1 (C_q), 136.5 (CH), 136.3 (CH), 132.9 (CH), 132.8 (CH), 132.6 (CH), 132.4 (C_q), 132.2 (CH), 131.1 (CH), 130.0 (C_q), 128.3 (CH), 126.5 (CH), 126.4 (CH), 125.8 (C_q), 123.7 (C_q), 123.6 (CH), 123.0 (C_q), 121.5 (CH), 83.2 (CH), 82.6 (CH), 61.2 (CH), 41.9 (CH), 36.5 (CH_2), 35.6 (CH_2), 34.9 (CH_2), 32.8 (CH_2) ppm;

HRMS (ESI): *m/z* $[M+H]^+$ calcd for $C_{36}H_{26}Br_2N_2O_2$: 677.0434; found: 677.0432.

$[\alpha]^{D}_{20}$ = +112.0 (c = 0.25, $CHCl_3$).

R_t (AD-3 column, *n*-hexane/*i*-PrOH 60/40, 1.0 mL/min, 273.0 nm): tr(major) = 7.9 min, tr(minor) = 18.7 min, 99% ee.

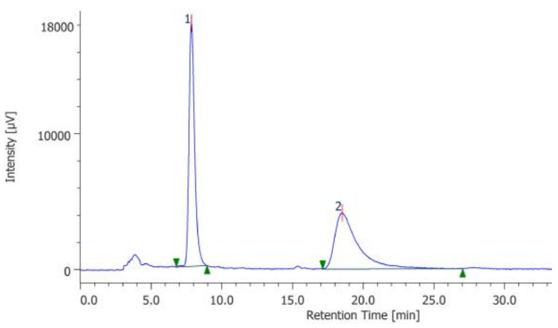

#	Peak Name	CH	tR [min]	Area [μV·sec]	Height [μV]	Area%
1	Unknown	10	7.850	497498	17896	51.390
2	Unknown	10	18.520	470576	4134	48.610

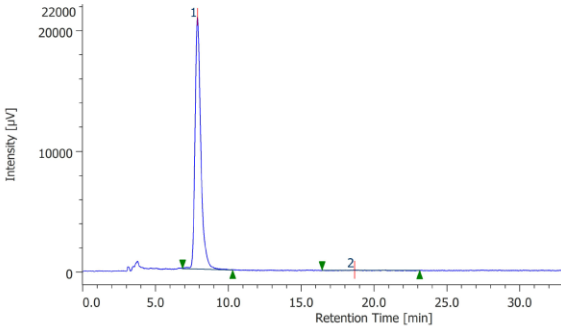

#	Peak Name	CH	tR [min]	Area [μV·sec]	Height [μV]	Area%
1	Unknown	10	7.880	575160	20892	99.720
2	Unknown	10	18.670	1616	45	0.280

(1^{6a}*R*,1^{7}*S*,1^{7a}*S*,1^{10a}*R*,1^{11}*S*,1^{11a}*S*)-19-phenyl-16-(quinolin-8-yl)-1^{6},1^{6a},1^{7},1^{7a},1^{8},1^{9},1^{10},1^{10a},1^{11},1^{11a}-decahydro-1^{5}*H*-1(1,4)-7,11-epoxypyrrolo[3,4-*b*]phenanthridina-4(1,4)-benzenacyclohexaphane-1^{5},1^{8},1^{10}-trione (10)

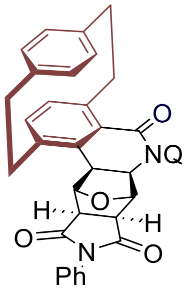

Prepared according to general procedure **3** for 30h on a 0.15 mmol scale, column chromatography (ethyl acetate/*n*-hexane = 1:2) afforded the compound **10** as a white solid (43 mg, 0.069 mmol, 46%), with >20:1 dr, 91% ee and chiral starting material (S_P)-**1a** as a white solid (23 mg, 0.062 mmol, 41%), with 99% ee.

^{1}H NMR (400 MHz, $CDCl_3$) δ 8.78 (dd, *J* = 4.2, 1.7 Hz, 1H), 8.23 (dd, *J* = 8.4, 1.8 Hz, 1H), 7.90 (dt, *J* = 7.6, 2.0 Hz, 2H), 7.72 (t, *J* = 7.8 Hz, 1H), 7.51 – 7.46 (m, 2H), 7.44 – 7.38 (m, 2H), 7.34 – 7.28 (m, 2H), 6.79 – 6.58 (m, 5H), 6.55 (d, *J* = 7.6 Hz, 1H), 5.91 (s, 1H), 5.00 – 4.84 (m, 2H), 4.51 (ddd, *J* = 12.5, 9.7, 2.4 Hz, 1H), 3.49 – 3.17 (m, 6H), 3.15 – 3.03 (m, 2H), 2.93 (d, *J* = 7.0 Hz, 1H), 2.92 – 2.81 (m, 1H) ppm;

^{13}C NMR (101 MHz, $CDCl_3$) δ 175.7 (C_q), 175.3 (C_q), 163.5 (C_q), 150.6 (CH), 144.8 (C_q), 144.0 (C_q), 141.4 (C_q), 137.9 (CH), 137.8 (C_q), 137.4 (C_q), 136.8 (CH), 136.6 (CH), 133.1 (CH), 132.9 (CH), 132.1 (C_q), 131.8 (CH), 131.6 (C_q), 131.0 (CH), 130.2 (C_q), 129.3 (CH), 129.0 (CH), 128.6 (CH), 126.6 (CH), 126.5 (CH), 125.7 (C_q), 121.6 (CH), 83.6 (CH), 82.9 (CH), 63.6 (CH), 50.8 (CH), 45.8 (CH), 43.2 (CH), 36.7 (CH_2), 35.5 (CH_2), 34.8 (CH_2), 33.0 (CH_2) ppm;

HRMS (ESI): *m/z* $[M+H]^+$ calcd for $C_{40}H_{31}N_3O_4$: 618.2387; found: 618.2387.

$[\alpha]^{D}_{20}$ = +140.6 (c = 0.50, $CHCl_3$).

R_t (AD-3 column, *n*-hexane/*i*-PrOH 60/40, 1.0 mL/min, 273.0 nm): tr(major) = 36.3 min, tr(minor) = 28.3 min, 91% ee.

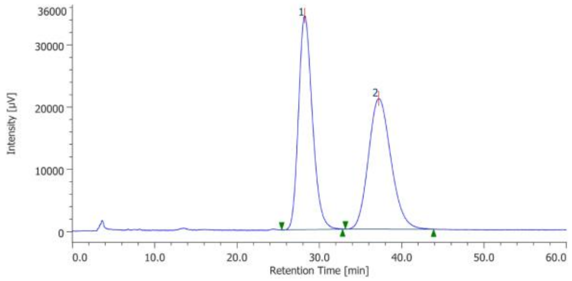

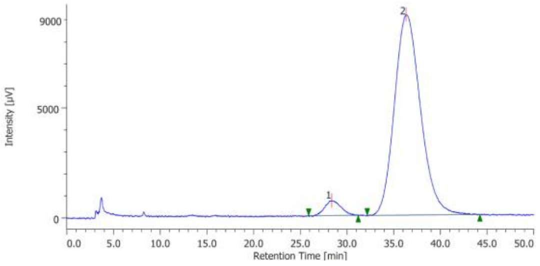

#	Peak Name	CH	tR [min]	Area [µV·sec]	Height [µV]	Area%
1	Unknown	9	28.200	4017522	34367	50.247
2	Unknown	9	37.207	3978036	20978	49.753

#	Peak Name	CH	tR [min]	Area [µV·sec]	Height [µV]	Area%
1	Unknown	10	28.360	81646	688	4.416
2	Unknown	10	36.313	1767127	9115	95.584

($1^{6a}R$,$1^{7}S$,$1^{7a}R$,$1^{10a}S$,$1^{11}S$,$1^{11a}S$)-19-phenyl-16-(quinolin-8-yl)-1^{6},1^{6a},1^{7},1^{7a},1^{8},1^{9},1^{10},1^{10a},1^{11},1^{11a}-decahydro-1^{5}*H*-1(1,4)-7,11-epoxypyrrolo[3,4-*b*]phenanthridina-4(1,4)-benzenacyclohexaphane-1^{5},1^{8},1^{10}-trione (11)

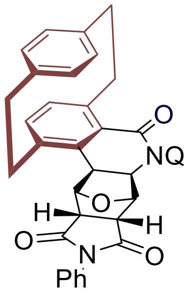

Prepared according to general procedure **3** for 30h on a 0.15 mmol scale, column chromatography (ethyl acetate/*n*-hexane = 1:2) afforded the compound **11** as a white solid (43 mg, 0.069 mmol, 46%), with >20:1 dr, 99% ee and chiral starting material (S_P)-**1a** as a white solid (27 mg, 0.072 mmol, 48%), with 99% ee.

^{1}H NMR (400 MHz, $CDCl_3$) δ 8.78 (s, 1H), 8.19 (dd, *J* = 8.3, 1.7 Hz, 1H), 8.00 – 7.84 (m, 2H), 7.71 (t, *J* = 7.8 Hz, 1H), 7.52 – 7.32 (m, 4H), 7.24 – 7.16 (m, 2H), 6.80 – 6.63 (m, 4H), 6.56 (d, *J* = 7.6 Hz, 1H), 6.50 (d, *J* = 7.6 Hz, 1H), 5.97 (dd, *J* = 5.9, 1.4 Hz, 1H), 5.28 (s, 1H), 4.99 (dd, *J* = 6.7, 1.4 Hz, 1H), 4.53 (ddd, *J* = 12.6, 9.7, 2.6 Hz, 1H), 3.90 (dd, *J* = 9.7, 5.9 Hz, 1H), 3.68 (d, *J* = 9.0 Hz, 1H), 3.53 (dd, *J* = 9.7, 6.7 Hz, 1H), 3.43 – 3.17 (m, 4H), 3.17 – 3.02 (m, 2H), 2.89 (ddd, *J* = 12.8, 9.7, 6.6 Hz, 1H) ppm;

^{13}C NMR (101 MHz, $CDCl_3$) δ 174.0 (C_q), 173.1 (C_q), 163.0 (C_q), 150.3 (CH), 144.8 (C_q), 141.4 (C_q), 138.2 (CH), 137.9 (C_q), 137.7 (C_q), 136.6 (CH), 136.5 (CH), 132.8 (CH), 132.01 (CH), 131.7 (C_q), 131.2 (CH), 131.1 (C_q), 130.0 (C_q), 129.3 (CH), 129.1 (CH), 128.4 (CH), 126.4 (CH), 126.2 (CH), 125.9 (C_q), 121.5 (CH), 82.7 (CH), 82.2 (CH), 61.7 (CH), 51.6 (CH), 48.4 (CH), 40.7 (CH), 36.7 (CH_2), 35.6 (CH_2), 34.9 (CH_2), 32.0 (CH_2) ppm;

HRMS (ESI): *m/z* $[M+H]^+$ calcd for $C_{40}H_{31}N_3O_4$: 618.2387; found: 618.2385.

$[\alpha]^{D}_{20}$ = +38.8 (c = 0.50, $CHCl_3$).

R_t (AD-3 column, *n*-hexane/*i*-PrOH 60/40, 1.0 mL/min, 250.0 nm): tr(major) = 17.5 min, tr(minor) = 20.5 min, 99% ee.

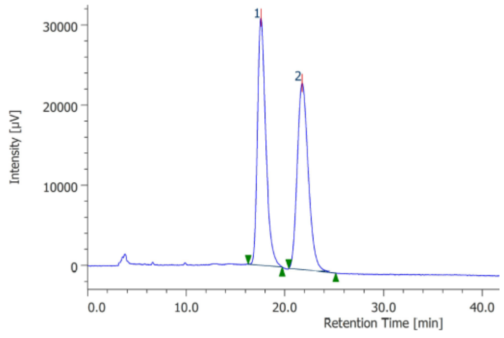

#	Peak Name	CH	tR [min]	Area [µV·sec]	Height [µV]	Area%
1	Unknown	9	17.580	1802369	30880	49.926
2	Unknown	9	21.760	1807729	23281	50.074

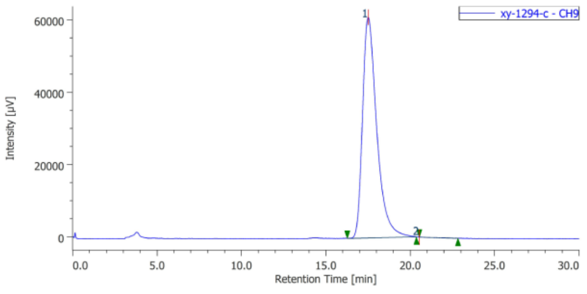

#	Peak Name	CH	tR [min]	Area [µV·sec]	Height [µV]	Area%
1	Unknown	9	17.507	3569633	61006	99.991
2	Unknown	9	20.537	306	8	0.009

($1^{6a}R$,$1^{12a}R$)-1^{13}-acetyl-1^{6}-(quinolin-8-yl)-1^{5},1^{6},1^{6a},1^{7},1^{12},1^{12a}-hexahydro-1(1,4)-7,12-epiminobenzo[*b*]phenanthridina-4(1,4)-benzenacyclohexaphan-1^{5}-one (12)

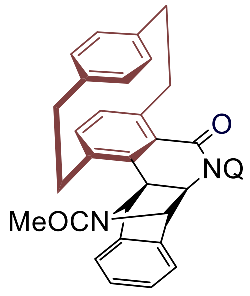

Prepared according to general procedure **3** for 30h on a 0.15 mmol scale, column chromatography (ethyl acetate/*n*-hexane = 1:2) afforded the compound **12** as a white solid (36 mg, 0.065 mmol, 43%), with >20:1 dr, 99% ee and chiral starting material (S_P)-**1a** as a white solid (28 mg, 0.075 mmol, 50%), with 99% ee.

^{1}H NMR (400 MHz, $CDCl_3$) δ 8.76 (dd, *J* = 4.2, 1.7 Hz, 1H), 8.24 (dd, *J* = 8.3, 1.8 Hz, 1H), 7.95 (d, *J* = 8.0 Hz, 2H), 7.76 (dd, *J* = 8.3, 7.2 Hz, 1H), 7.53 (d, *J* = 7.3 Hz, 1H), 7.40 (dd, *J* = 8.4, 4.1 Hz, 1H), 7.33 – 7.16 (m, 4H), 7.15 – 7.03 (m, 2H), 7.01 – 6.92 (m, 2H), 6.79 – 6.69 (m, 2H), 6.57 (td, *J* = 4.9, 2.3 Hz, 2H), 6.49 (d, *J* = 7.6 Hz, 1H), 6.36 (d, *J* = 1.5 Hz, 1H), 5.94 – 5.76 (m, 0.5H), 5.56 – 5.47 (m, 0.5H), 5.00 (d, *J* = 1.5 Hz, 1H), 4.78 (d, *J* = 8.5 Hz, 1H), 4.59 – 4.47 (m, 1H), 3.74 – 3.62 (m, 1H), 3.39 – 3.20 (m, 5H), 3.16 – 3.05 (m, 1H), 2.98 – 2.86 (m, 1H), 2.11 (s, 3H) ppm;

^{13}C NMR (101 MHz, $CDCl_3$) δ 171.3 (C_q), 166.9 (C_q), 164.01 (C_q), 150.6 (CH), 149.2 (C_q), 148.4 (C_q), 148.0 (C_q), 144.8 (C_q), 144.3 (CH), 144.3 (C_q), 142.3 (CH), 141.0 (C_q), 140.3 (C_q), 138.2 (C_q), 137.8 (CH), 137.7 (C_q), 136.6 (CH), 136.3 (CH), 132.6 (C_q), 132.6 (CH), 132.4 (CH), 132.1 (CH), 131.8 (CH), 130.1 (C_q), 128.4 (CH), 127.8 (CH), 127.0 (CH), 126.3 (CH), 125.3 (CH), 125.3 (C_q), 125.1 (CH), 121.7 (CH), 121.5 (CH), 121.4 (CH), 120.3 (CH), 119.4 (CH), 67.5 (CH), 66.2 (CH), 64.1 (CH), 63.3 (CH), 62.4 (CH), 41.5 (CH), 36.2 (CH_2), 35.5 (CH_2), 35.2 (CH_2), 32.4 (CH_2), 22.6 (CH_3) ppm;

HRMS (ESI): *m/z* $[M+H]^+$ calcd for $C_{38}H_{31}N_3O_2$: 562.2489; found: 562.2485.

$[\alpha]^{D}_{20}$ = +102.4 (c = 0.50, $CHCl_3$).

R_t (AD-3 column, *n*-hexane/*i*-PrOH 60/40, 1.0 mL/min, 250.0 nm): tr(major) = 8.3 min, tr(minor) = 19.4 min, 99% ee.

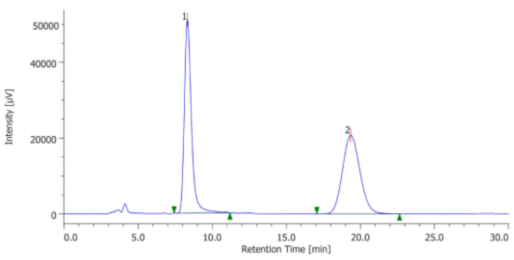

#	Peak Name	CH	tR [min]	Area [µV·sec]	Height [µV]	Area%
1	Unknown	9	8.323	1697157	51010	50.035
2	Unknown	9	19.333	1694766	20738	49.965

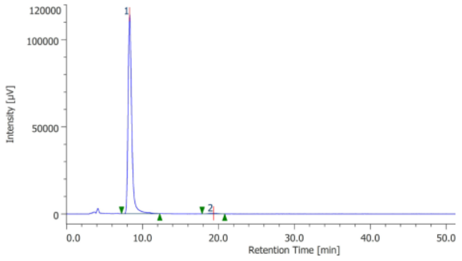

#	Peak Name	CH	tR [min]	Area [µV·sec]	Height [µV]	Area%
1	Unknown	9	8.313	3787953	114334	99.374
2	Unknown	9	19.353	23852	302	0.626

tert-butyl (1[6a]*R*,1[7]*S*,1[12]*R*,1[12a]*R*)-1[5]-oxo-1[6]-(quinolin-8-yl)-1[5],1[6],1[6a],1[7],1[12],1[12a]-hexahydro-1(1,4)-7,12-epiminobenzo[*b*]phenanthridina-4(1,4)-benzenacyclohexaphane-1[13]-carboxylate (13)

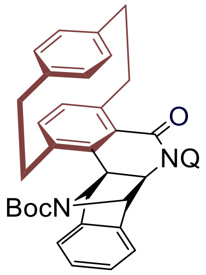

Prepared according to general procedure **3** for 60h on a 0.15 mmol scale, column chromatography (ethyl acetate/*n*-hexane = 1:2) afforded the compound **13** as a white solid (39 mg, 0.063 mmol, 42%), with >20:1 dr, 96% ee and chiral starting material (S_P)-**1a** as a white solid (24 mg, 0.063 mmol, 42%), with 99% ee.

^{1}H NMR (400 MHz, $CDCl_3$) δ 8.70 (dd, *J* = 4.3, 1.7 Hz, 1H), 8.21 (dd, *J* = 8.4, 1.8 Hz, 1H), 8.09 (dd, *J* = 7.3, 1.5 Hz, 1H), 7.93 (dd, *J* = 8.3, 1.5 Hz, 1H), 7.80 (t, *J* = 7.8 Hz, 1H), 7.50 (d, *J* = 7.2 Hz, 1H), 7.35 (dd, *J* = 8.3, 4.1 Hz, 1H), 7.26 – 7.14 (m, 2H), 7.08 (td, *J* = 7.4, 1.0 Hz, 1H), 7.01 (d, *J* = 6.7 Hz, 1H), 6.82 – 6.68 (m, 2H), 6.68 – 6.53 (m, 2H), 6.49 (d, *J* = 7.6 Hz, 1H), 6.22 – 5.51 (m, 1H), 5.28 – 5.01 (m, 2H), 4.58 (ddd, *J* = 12.7, 9.8, 2.8 Hz, 1H), 3.68 – 3.45 (m, 1H), 3.41 – 3.18 (m, 5H), 3.11 (ddd, *J* = 12.8, 9.9, 2.7 Hz, 1H), 2.99 – 2.83 (m, 1H), 1.44 (s, 9H) ppm;

^{13}C NMR (101 MHz, $CDCl_3$) δ 164.3 (C_q), 155.8 (C_q), 150.5 (CH), 148.2 (C_q), 144.5 (C_q), 144.1 (C_q), 141.6 (C_q), 138.0 (C_q), 137.7 (C_q), 137.7 (CH), 136.5 (CH), 136.3 (CH), 133.3 (CH), 132.9 (CH), 132.6 (CH), 132.2 (CH), 131.9 (CH), 130.0 (C_q), 128.1 (CH), 127.5 (CH), 126.9 (CH), 126.7 (CH), 125.7 (C_q), 122.6 (CH), 121.4 (CH), 118.6 (CH), 81.1 (C_q), 66.9 (CH), 66.1 (CH), 60.4 (CH), 43.1 (CH), 36.2 (CH_2), 35.5 (CH_2), 35.1 (CH_2), 32.5 (CH_2), 28.3 (CH_3) ppm;

HRMS (ESI): *m/z* $[M+H]^+$ calcd for $C_{41}H_{37}N_3O_3$: 620.2908; found: 620.2910.

$[\alpha]^D_{20}$ = +117.8 (c = 0.50, $CHCl_3$).

R_t (AD-3 column, *n*-hexane/*i*-PrOH 95/5, 1.0 mL/min, 273.0 nm): tr(major) = 19.8 min, tr(minor) = 26.8 min, 96% ee.

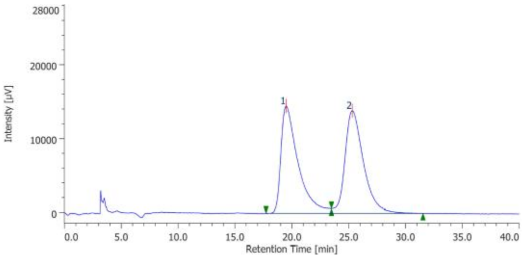

#	Peak Name	CH	tR [min]	Area [µV·sec]	Height [µV]	Area%
1	Unknown	10	19.480	1516848	14497	49.231
2	Unknown	10	25.310	1564209	13863	50.769

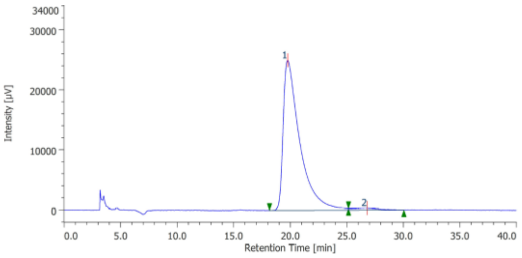

#	Peak Name	CH	tR [min]	Area [µV·sec]	Height [µV]	Area%
1	Unknown	10	19.753	2529728	24993	98.005
2	Unknown	10	26.793	51506	340	1.995

benzyl ($1^{6a}R$,$1^{7}R$,$1^{12}R$,$1^{12a}R$)-1^{5}-oxo-1^{6}-(quinolin-8-yl)-1^{5},1^{6},1^{6a},1^{7},1^{12},1^{12a}-hexahydro-1(1,4)-7,12-epiminobenzo[*b*]phenanthridina-4(1,4)-benzenacyclohexaphane-1^{13}-carboxylate (14)

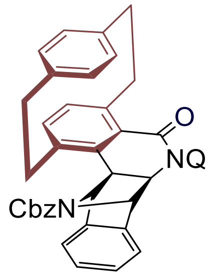

Prepared according to general procedure **3** for 48h on a 0.15 mmol scale, column chromatography (ethyl acetate/*n*-hexane = 1:2) afforded the compound **14** as a white solid (47 mg, 0.072 mmol, 48%), with >20:1 dr, 96% ee and chiral starting material (S_P)-**1a** as a white solid (26 mg, 0.0675 mmol, 45%), with 99% ee.

^{1}H NMR (300 MHz, $CDCl_3$) δ 8.68 (dd, J = 4.2, 1.8 Hz, 1H), 8.20 (dd, J = 8.3, 1.7 Hz, 1H), 7.89 (d, J = 8.1 Hz, 2H), 7.61 – 6.94 (m, 11H), 6.82 – 6.44 (m, 5H), 6.10 (s, 1H), 5.46 – 4.86 (m, 3H), 4.70 – 4.47 (m, 2H), 3.80 – 3.02 (m, 7H), 2.98 – 2.72 (m, 1H) ppm; **^{13}C NMR** (101 MHz, $CDCl_3$) δ 164.2 (C_q), 156.4 (C_q), 150.4 (CH), 148.5 (CH), 144.6 (C_q), 144.1 (C_q), 141.3 (C_q), 140.8 (CH), 138.0 (C_q), 137.7 (CH), 137.5 (C_q), 136.4 (CH), 136.4 (CH), 133.2 (CH), 132.7 (CH), 132.6 (CH), 132.6 (C_q), 132.2 (CH), 131.7 (CH), 130.0 (CH), 128.6 (CH), 128.3 (CH), 128.1 (CH), 128.0 (CH), 127.7 (CH), 127.1 (CH), 125.5 (C_q), 122.3 (CH), 121.4 (CH), 119.1 (CH), 67.7 (C_q), 66.9 (CH), 65.9 (CH), 61.1 (CH), 42.3 (CH), 36.3 (CH), 35.5 (CH_2), 35.1 (CH_2), 32.5 (CH_2), 31.6 (CH_2), 22.7 (CH_2), 14.2 (CH) ppm;

HRMS (ESI): *m/z* $[M+H]^+$ calcd for $C_{44}H_{35}N_3O_3$: 654.2751; found: 654.2756.

$[\alpha]^{D}_{20}$ = +130.4 (c = 0.50, $CHCl_3$).

R_t (AD-3 column, *n*-hexane/*i*-PrOH 60/40, 1.0 mL/min, 273.0 nm): tr(major) = 7.5 min, tr(minor) = 16.8 min, 96% ee.

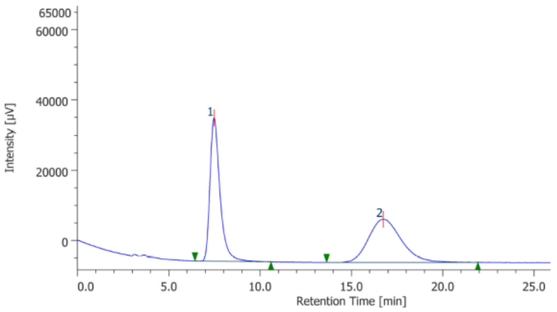

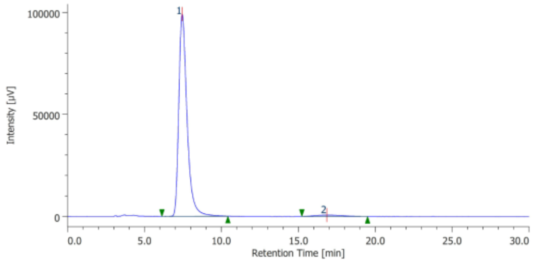

#	Peak Name	CH	tR [min]	Area [µV·sec]	Height [µV]	Area%
1	Unknown	10	7.477	1553646	40714	50.402
2	Unknown	10	16.740	1528870	12337	49.598

#	Peak Name	CH	tR [min]	Area [µV·sec]	Height [µV]	Area%
1	Unknown	10	7.450	3695610	99021	97.858
2	Unknown	10	16.843	80900	684	2.142

(*R*)-1³-(methoxymethyl)-1²-(quinolin-8-yl)-1¹,1²,1³,1⁴-tetrahydro-1(5,8)-isoquinolina-4(1,4)-benzenacyclohexaphan-1¹-one (15)

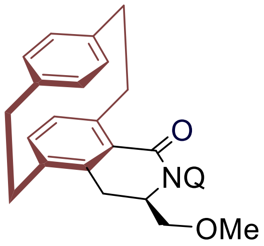

Prepared according to general procedure **3** for 48h on a 0.15 mmol scale, column chromatography (ethyl acetate/*n*-hexane = 1:2) afforded the compound **15** as a white solid (31 mg, 0.0676 mmol, 46%), with >20:1 dr, 3.3:1 rr, 99% ee and chiral starting material (S_P)-**1a** as a white solid (27 mg, 0.072 mmol, 48%), with 99% ee.

^{1}H NMR (400 MHz, $CDCl_3$) δ 8.75 (dd, *J* = 4.3, 1.8 Hz, 1H), 8.24 – 8.11 (m, 1H), 7.90 – 7.78 (m, 2H), 7.73 – 7.57 (m, 1H), 7.35 (dd, *J* = 8.3, 4.2 Hz, 1H), 6.92 (dd, *J* = 7.9, 2.0 Hz, 1H), 6.71 – 6.47 (m, 5H), 4.49 – 4.29 (m, 2H), 3.42 – 2.64 (m, 14H) ppm;

^{13}C NMR (101 MHz, $CDCl_3$) δ 166.6 (C_q), 150.4 (CH), 145.2 (C_q), 143.9 (C_q), 141.0 (C_q), 138.7 (C_q), 137.7 (C_q), 137.2 (C_q), 136.3 (CH), 136.1 (CH), 134.3 (CH), 133.4 (CH), 132.8 (CH), 132.0 (CH), 130.4 (CH), 129.1 (C_q), 128.8 (C_q), 128.1 (C_q), 127.8 (CH), 127.1 (CH), 126.3 (CH), 121.3 (CH), 73.3 (CH_2), 58.7 (CH), 55.1 (CH_3), 36.8 (CH_2), 34.2 (CH_2), 33.9 (CH_2), 32.0 (CH_2), 31.0 (CH_2) ppm;

HRMS (ESI): *m/z* $[M+H]^+$ calcd for $C_{30}H_{28}N_2O_2$: 449.2224; found: 449.2226.

$[\alpha]^D{}_{20}$ = +24.0 (c = 0.50, $CHCl_3$).

R_t (AD-3 column, *n*-hexane/*i*-PrOH 70/30, 1.0 mL/min, 273.0 nm): tr(major) = 9.1 min, tr(minor) = 18.1 min, 99% ee.

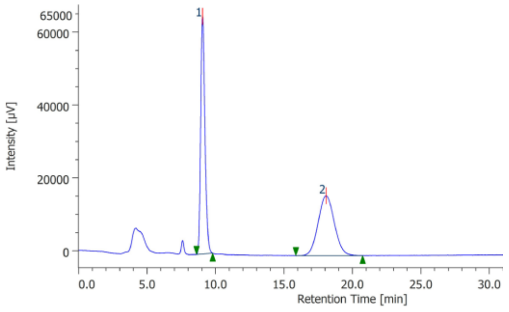

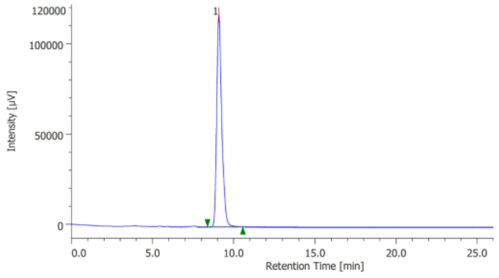

#	Peak Name	CH	tR [min]	Area [µV·sec]	Height [µV]	Area%
1	Unknown	10	9.053	1408408	65182	51.063
2	Unknown	10	18.073	1349747	16426	48.937

#	Peak Name	CH	tR [min]	Area [µV·sec]	Height [µV]	Area%
1	Unknown	10	9.083	2543039	117338	100.000

(*R*)-1³-(phenoxymethyl)-1²-(quinolin-8-yl)-1¹,1²,1³,1⁴-tetrahydro-1(5,8)-isoquinolina-4(1,4)-benzenacyclohexaphan-1¹-one (16)

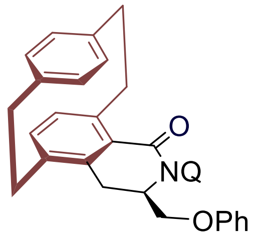

Prepared according to general procedure **3** for 24h on a 0.15 mmol scale, column chromatography (ethyl acetate/*n*-hexane = 1:2) afforded the compound **16** as a white solid (36 mg, 0.0705 mmol, 47%), with >20:1 dr, 3.3:1 rr, 99% ee and chiral starting material (S_P)-**1a** as a white solid (26 mg, 0.0675 mmol, 45%), with 99% ee.

^{1}H NMR (400 MHz, $CDCl_3$) δ 8.76 (dd, *J* = 4.1, 1.7 Hz, 1H), 8.15 (dd, *J* = 8.3, 1.8 Hz, 1H), 7.85 (dd, *J* = 7.3, 1.4 Hz, 1H), 7.78 (dd, *J* = 8.3, 1.4 Hz, 1H), 7.53 (dd, *J* = 8.2, 7.2 Hz, 1H), 7.36 (dd, *J* = 8.3, 4.2 Hz, 1H), 7.22 – 7.13 (m, 2H), 6.96 (dd, *J* = 7.8, 2.0 Hz, 1H), 6.90 (t, *J* = 7.4 Hz, 1H), 6.72 – 6.51 (m, 7H), 4.73 – 4.62 (m, 1H), 4.45 – 4.30 (m, 1H), 3.73 – 3.65 (m, 1H), 3.59 (dd, *J* = 10.1, 4.9 Hz, 1H), 3.32 – 2.91 (m, 8H), 2.75 (ddd, *J* = 12.0, 10.0, 7.0 Hz, 1H) ppm;

^{13}C NMR (101 MHz, $CDCl_3$) δ 166.7 (C_q), 158.2 (C_q), 150.4 (CH), 145.1 (C_q), 144.1 (C_q), 141.0 (C_q), 138.6 (C_q), 138.3 (C_q), 137.4 (C_q), 137.3 (C_q), 136.4 (CH), 136.2 (CH), 134.4 (CH), 133.5 (CH), 132.9 (CH), 132.1 (CH), 130.3 (CH), 129.3 (CH), 129.2 (C_q), 128.8 (C_q), 128.0 (CH), 126.9 (CH), 126.3 (CH), 121.4 (CH), 121.0 (CH), 114.3 (CH), 68.4 (CH_2), 54.7 (CH), 36.9 (CH_2), 34.2 (CH_2), 33.8 (CH_2), 32.1 (CH_2), 30.9 (CH_2) ppm;

HRMS (ESI): *m/z* $[M+H]^+$ calcd for $C_{35}H_{30}N_2O_2$: 511.2380; found: 511.2381.

$[\alpha]^D{}_{20}$ = +100.4 (c = 0.50, $CHCl_3$).

R_t (AD-3 column, *n*-hexane/*i*-PrOH 70/30, 1.0 mL/min, 273.0 nm): tr(major) = 17.1 min, tr(minor) = 25.7 min, 99% ee.

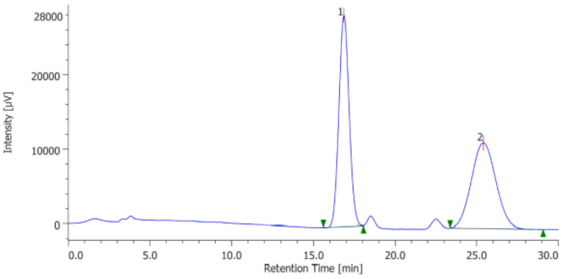

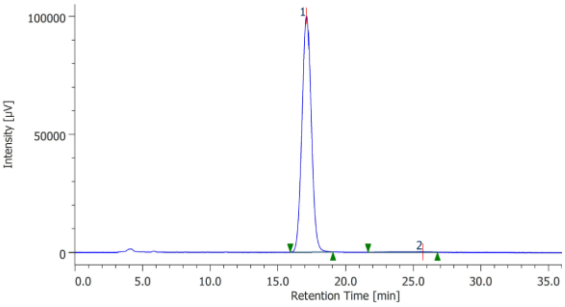

#	Peak Name	CH	tR [min]	Area [µV·sec]	Height [µV]	Area%
1	Unknown	10	16.853	1241780	28356	50.863
2	Unknown	10	25.397	1199658	11497	49.137

#	Peak Name	CH	tR [min]	Area [µV·sec]	Height [µV]	Area%
1	Unknown	10	17.127	4638410	100048	99.431
2	Unknown	10	25.723	26553	169	0.569

(*R*)-1[3]-(hydroxymethyl)-1[2]-(quinolin-8-yl)-1[1],1[2],1[3],1[4]-tetrahydro-1(5,8)-isoquinolina-4(1,4)-benzenacyclohexaphan-1[1]-one (17)

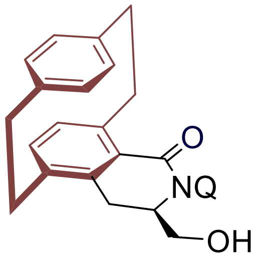

Prepared according to general procedure **3** for 72h on a 0.15 mmol scale, column chromatography (ethyl acetate/*n*-hexane = 1:2) afforded the compound **17** as a white solid (31 mg, 0.071 mmol, 47%), with 20:1 dr, 6.7:1 rr, 99% ee and chiral starting material (S_P)-**1a** as a white solid (23 mg, 0.06 mmol, 40%), with 96% ee.

^{1}H NMR (400 MHz, $CDCl_3$) δ 9.02 (dd, *J* = 4.2, 1.7 Hz, 1H), 8.31 (dd, *J* = 8.3, 1.7 Hz, 1H), 7.90 (dd, *J* = 8.2, 1.4 Hz, 1H), 7.67 – 7.58 (m, 1H), 7.56 (dd, *J* = 8.3, 4.2 Hz, 1H), 7.48 (dd, *J* = 7.3, 1.4 Hz, 1H), 7.18 (dd, *J* = 7.9, 2.0 Hz, 1H), 6.69 – 6.56 (m, 4H), 6.49 (dd, *J* = 8.1, 2.0 Hz, 1H), 5.26 (dd, *J* = 9.7, 4.0 Hz, 1H), 4.30 (ddd, *J* = 11.5, 7.8, 3.1 Hz, 1H), 4.00 – 3.87 (m, 1H), 3.67 (dd, *J* = 16.4, 13.1 Hz, 1H), 3.48 – 3.36 (m, 2H), 3.33 – 3.23 (m, 1H), 3.16 – 2.89 (m, 6H), 2.77 – 2.66 (m, 1H) ppm;

^{13}C NMR (101 MHz, $CDCl_3$) δ 166.5 (C_q), 151.0 (CH), 146.2 (C_q), 144.0 (C_q), 140.7 (C_q), 139.3 (C_q), 138.4 (C_q), 138.3 (C_q), 137.8 (C_q), 137.5 (CH), 136.5 (CH), 133.9 (CH), 133.5 (CH), 132.6 (CH), 130.9 (CH), 130.0 (C_q), 129.5 (CH), 128.7 (C_q), 128.4 (CH), 127.3 (CH), 127.1 (CH), 122.2 (CH), 62.9 (CH_2), 58.5 (CH), 37.0 (CH_2), 34.1 (CH_2), 33.6 (CH_2), 32.1 (CH_2), 28.1 (CH_2) ppm;

HRMS (ESI): *m/z* $[M+H]^+$ calcd for $C_{29}H_{26}N_2O_2$: 435.2067; found: 435.2070.

$[\alpha]^D_{20}$ = +0.6 (c = 0.50, $CHCl_3$).

R_t (AD-3 column, *n*-hexane/*i*-PrOH 70/30, 1.0 mL/min, 273.0 nm): tr(major) = 11.6 min, tr(minor) = 19.6 min, 99% ee.

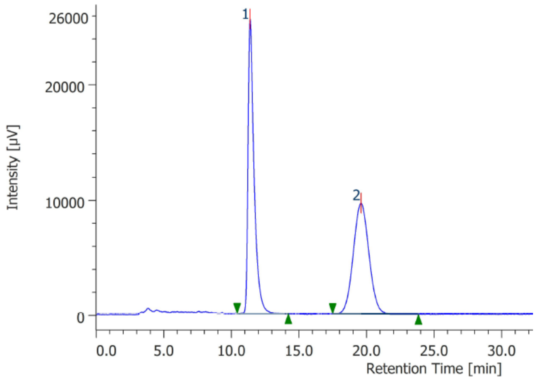

#	Peak Name	CH	tR [min]	Area [µV·sec]	Height [µV]	Area%
1	Unknown	10	11.383	793194	25554	50.626
2	Unknown	10	19.597	773592	9643	49.374

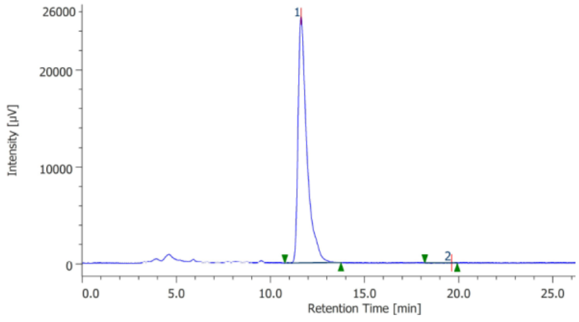

#	Peak Name	CH	tR [min]	Area [µV·sec]	Height [µV]	Area%
1	Unknown	10	11.623	828296	25425	99.979
2	Unknown	10	19.627	176	25	0.021

(R_p)-1[3]-((diphenylphosphoryl)methyl)-1[2]-(quinolin-8-yl)-1[1],1[2]-dihydro-1(5,8)-isoquinolina-4(1,4)-benzenacyclohexaphan-1[1]-one (18)

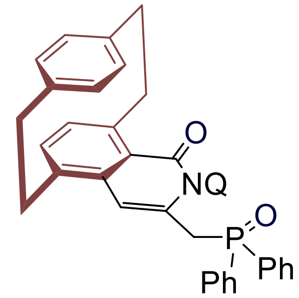

Prepared according to general procedure **3** for 24h on a 0.15 mmol scale, column chromatography (ethyl acetate/*n*-hexane = 1:1 to 100% EtOAc) afforded the compound **18** as a white solid (41 mg, 0.066 mmol, 44%), with 98% ee and chiral starting material (S_P)-**1a** as a white solid (23 mg, 0.060 mmol, 40%), with 94% ee.

^{1}H NMR (300 MHz, $CDCl_3$) δ 8.78 (dd, J = 4.2, 1.7 Hz, 1H), 8.24 (dd, J = 8.3, 1.7 Hz, 1H), 8.07 – 7.71 (m, 5H), 7.65 – 7.32 (m, 9H), 6.79 – 6.58 (m, 3H), 6.59 – 6.48 (m, 1H), 6.44 – 6.28 (m, 3H), 4.64 – 4.52 (m, 1H), 3.56 (dd, J = 16.0, 12.9 Hz, 1H), 3.28 (dd, J = 12.4, 10.0 Hz, 1H), 3.19 – 2.73 (m, 6H), 2.60 – 2.46 (m, 1H) ppm;

^{13}C NMR (75 MHz, $CDCl_3$) δ 163.3 (C_q), 151.4 (CH), 144.5 (C_q), 142.9 (C_q), 140.0 (C_q), 139.3 (d, J_{CP} = 3.0 Hz, C_q), 138.3 (C_q), 136.5 (CH), 136.3 (C_q), 136.3 (CH), 135.7 (C_q), 133.9 (C_q), 133.8 (C_q), 133.6 (CH), 133.2 (CH), 133.0 (C_q), 132.3 (d, J_{CP} = 2.8 Hz, CH), 132.1 (CH), 132.1 (CH), 131.4 (CH), 131.3 (CH), 130.7 (CH), 130.6 (CH), 123.0 (CH), 129.3 (CH), 129.2 (CH), 129.2 (C_q), 129.1 (CH), 129.0 (CH), 128.9 (CH), 128.8 (CH), 126.8 (C_q), 126.7 (CH), 121.8 (CH), 105.9 (d, J_{CP} = 6.6 Hz, CH), 36.4 (CH_2), 34.2 (CH_2), 34.0 (d, J_{CP} = 67.2 Hz, CH_2), 33.7 (CH_2), 32.8 (CH_2) ppm;

^{31}P NMR (121 MHz, $CDCl_3$) δ 27.32 ppm.

HRMS (ESI): *m/z* $[M+H]^+$ calcd for $C_{41}H_{33}N_2O_2PNa$: 639.2172; found: 639.2172.

$[\alpha]_D$ ^{20} = -254.6 (c =0.5, $CHCl_3$);

m.p.: 228-231 °C.

R_t (AD column, *n*-hexane/*i*-PrOH 60/40, 1.0 mL/min, 273.0 nm): tr(major) = 22.7 min, tr(minor) = 14.1 min, 97% ee.

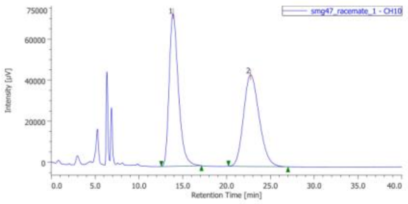

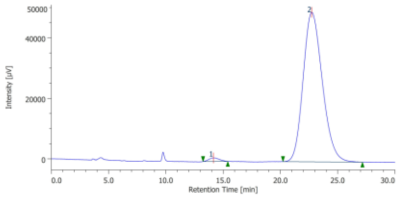

#	Peak Name	CH	tR [min]	Area [μV·sec]	Height [μV]	Area%
1	Unknown	10	13.887	5454852	74312	49.577
2	Unknown	10	22.743	5548042	44689	50.423

#	Peak Name	CH	tR [min]	Area [μV·sec]	Height [μV]	Area%
1	Unknown	10	14.143	74472	1175	1.279
2	Unknown	10	22.730	5748462	49352	98.721

(1^3*S*)-1^3-(((8*S*,9*R*,13*R*)-13-methyl-17-oxo-7,8,9,11,12,13,14,15,16,17-decahydro-6*H*-cyclopenta[*a*]phenanthren-2-y)methyl)-1^2-(quinolin-8-yl)-1^1,1^2,1^3,1^4-tetrahydro-1(5,8)-isoquinolina-4(1,4)-benzenacyclohexaphan-1^1-one (19)

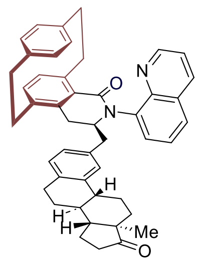

Prepared according to general procedure **3** for 24h on a 0.15 mmol scale, column chromatography (ethyl acetate/*n*-hexane = 1:2) afforded the compound **19** as a white solid (45 mg, 0.0675 mmol, 45%), with 14:1 dr, 2.0:1 rr, 99% ee and chiral starting material (S_P)-**1a** as a white solid (26 mg, 0.071 mmol, 47%), with 99% ee.

^{1}H NMR (400 MHz, $CDCl_3$, major isomer) δ 8.80 (dd, *J* = 4.2, 1.7 Hz, 1H), 8.12 (dd, *J* = 8.3, 1.7 Hz, 1H), 7.90 – 7.80 (m, 2H), 7.67 (dd, *J* = 8.3, 7.1 Hz, 1H), 7.34 (dd, *J* = 8.3, 4.2 Hz, 1H), 7.20 – 7.08 (m, 1H), 7.02 (d, *J* = 7.9 Hz, 1H), 6.89 (dd, *J* = 7.8, 2.0 Hz, 1H), 6.83 – 6.73 (m, 1H), 6.73 – 6.46 (m, 5H), 4.70 – 4.52 (m, 1H), 4.42 – 4.21 (m, 1H), 3.37 – 1.85 (m, 20H), 1.72 – 1.25 (m, 6H), 0.91 (s, 3H) ppm;

^{13}C NMR (101 MHz, $CDCl_3$, including regioisomers) δ 220.9 (C_q), 166.8 (C_q), 165.8 (C_q), 150.7 (CH), 150.3 (CH), 146.2 (C_q), 145.3 (C_q), 143.9 (C_q), 143.7 (C_q), 141.0 (C_q), 140.7 (C_q), 139.7, (C_q) 139.0 (C_q), 138.6 (C_q), 138.6 (C_q), 138.2 (C_q), 137.8 (C_q), 137.5 (C_q), 137.2 (C_q), 137.1 (C_q), 136.4 (C_q), 136.2 (CH), 136.2 (CH), 136.0 (CH), 135.4 (C_q), 135.0 (C_q), 134.4 (CH), 134.0 (CH), 133.4 (CH), 132.9 (CH), 132.9 (CH), 132.8 (CH), 132.0 (CH), 131.4 (CH), 130.7 (CH), 130.2 (CH), 129.5 (CH), 129.5 (C_q), 129.4 (C_q), 129.3, 129.2 (C_q), 129.0 (C_q), 128.1 (CH), 128.0 (CH), 127.9 (CH), 127.0 (CH), 126.4 (CH), 126.3 (CH), 126.3 (CH), 126.2 (CH), 125.3 (CH), 125.0 (CH), 121.6 (CH), 121.3 (CH), 57.2 (CH), 56.6 (CH), 50.5 (CH), 50.5 (CH), 48.0 (C_q), 44.2 (CH), 44.2 (CH), 41.3 (CH_2), 39.0 (CH_2), 38.1 (CH), 38.1 (CH), 36.8 (CH_2), 36.4 (CH_2), 35.9 (CH_2), 34.2 (CH_2), 33.9 (CH_2), 33.1 (CH_2), 32.3 (CH_2), 29.3 (CH_2), 26.4 (CH_2), 25.7 (CH_2), 21.6 (CH_2), 13.9 (CH_3) ppm;

HRMS (ESI): *m/z* $[M+H]^+$ calcd for $C_{47}H_{46}N_2O_2$: 671.3632; found: 671.3635.

$[\alpha]^D{}_{20}$ = +39.0 (c = 0.50, $CHCl_3$).

R_t (IG-3 column, *n*-hexane/*i*-PrOH 80/20, 1.0 mL/min, 250.0 nm): tr(major) = 16.2 min, tr(minor) = 12.3 min, 99% ee.

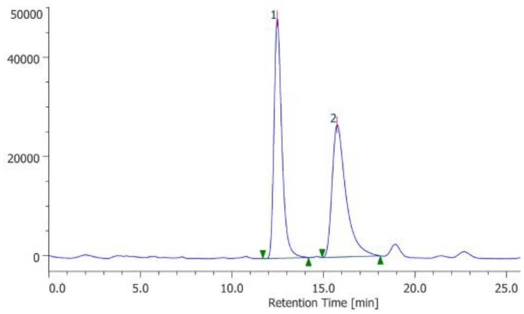

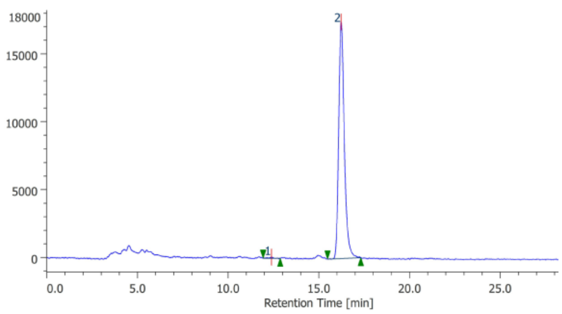

#	Peak Name	CH	tR [min]	Area [µV·sec]	Height [µV]	Area%
1	Unknown	9	12.467	1441158	48240	50.583
2	Unknown	9	15.737	1407912	26698	49.417

#	Peak Name	CH	tR [min]	Area [µV·sec]	Height [µV]	Area%
1	Unknown	9	12.387	1249	81	0.330
2	Unknown	9	16.227	376972	17419	99.670

(1^{9b}*R*)-1^{2}-((*R*)-2-(4-isobutylphenyl)propanoyl)-1^{4}-(quinolin-8-yl)-1^{2},1^{3},1^{3a},1^{4},1^{5},1^{9b}-hexahydro-11*H*-1(6,9)-pyrrolo[3,4-*c*]isoquinolina-4(1,4)-benzenacyclohexaphan-1^{5}-one (20)

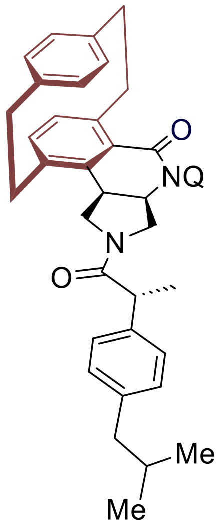

Prepared according to general procedure **3** for 72h on a 0.15 mmol scale, column chromatography (ethyl acetate/*n*-hexane = 1:3 to 2:1) afforded the compound **20** as a white solid (31 mg, 0.048 mmol, 32%), with >20:1 dr, and chiral starting material **1a** as a white solid (22 mg, 0.057 mmol, 38%), with 97% ee

^{1}H NMR (300 MHz, $CDCl_3$): 8.67 (ddd, *J* = 22.1, 4.2, 1.7 Hz, 1H), 8.13 (ddd, *J* = 29.3, 8.4, 1.8 Hz, 1H), 7.80 (ddd, *J* = 37.9, 7.9, 1.8 Hz, 1H), 7.67 – 7.60 (m, 1H), 7.44 (dd, *J* = 8.3, 7.3 Hz, 1H), 7.31 – 7.23 (m, 2H), 6.91 (s, 1H), 6.77 (dd, *J* = 7.3, 1.4 Hz, 1H), 6.68 – 6.56 (m, 2H), 6.54 (d, *J* = 7.8 Hz, 1H), 6.43 (t, *J* = 7.2 Hz, 2H), 6.21 – 6.00 (m, 1H), 4.75 (dt, *J* = 13.6, 3.4 Hz, 1H), 4.37 – 4.01 (m, 3H), 3.91 – 3.77 (m, 1H), 3.26 – 2.87 (m, 9H), 2.76 – 2.53 (m, 1H), 2.37 (dd, *J* = 38.6, 7.2 Hz, 2H), 1.89 – 1.64 (m, 1H), 1.36 (dd, *J* = 38.9, 6.9 Hz, 3H), 0.95 – 0.69 (m, 6H) ppm.

^{13}C NMR (75 MHz, $CDCl_3$) δ 172.3 (C_q), 164.6 (C_q), 150.2 (CH), 144.7 (C_q), 143.8 (C_q), 141.7 (C_q), 141.2 (C_q), 138.5 (C_q), 138.4 (C_q), 137.3 (C_q), 136.7 (CH), 136.6 (CH), 136.4 (CH), 135.8 (C_q), 134.2 (C_q), 133.2 (CH), 132.5 (CH), 131.8 (CH), 131.1 (CH), 130.8 (CH), 129.8 (CH), 129.5 (C_q), 129.5 (CH), 128.1 (CH), 127.6 (CH), 127.1 (CH), 126.5 (C_q), 126.4 (CH), 121.3 (CH), 58.5 (CH), 50.6 (CH_2), 50.1 (CH_2), 45.7 (CH), 45.2 (CH_2), 40.9 (CH), 36.9 (CH_2), 35.6 (CH_2), 34.8 (CH_2), 31.7 (CH_2), 30.5 (CH), 22.5 (CH_3), 22.5 (CH_3), 20.0 (CH_3) ppm. Because of the presence of conformers with high energy barriers, ^{13}C-NMR analysis shows the signals of both. Picks are referred to the major isomer.

HRMS (ESI): *m/z* $[M+H]^+$ calcd for $C_{43}H_{44}N_3O_2$: 634.3428; found: 634.3417.

$[\alpha]^D{}_{20}$ = +4.9 (c = 1, $CHCl_3$).

m.p.: 183-186 °C.

(1^{3a}*R*,1^{9b}*R*)-1^2-((pivaloyloxy)-*D*-alanyl)-14-(quinolin-8-yl)-1^2,1^3,1^{3a},1^4,1^5,1^{9b}-hexahydro-1^1H-1(6,9)-pyrrolo[3,4-*c*]isoquinolina-4(1,4)-benzenacyclohexaphan-1^5-one (21)

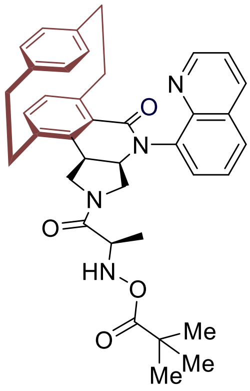

Prepared according to general procedure **3** for 72h on a 0.15 mmol scale, column chromatography (ethyl acetate/*n*-hexane = 1:2 to 3:1) afforded the compound **21** as a white solid (46 mg, 0.074 mmol, 49%), with >20:1 dr, and chiral starting material (S_p)-**1a** as a white solid (24 mg, 0.063 mmol, 42%), with 98% ee.

^{1}H NMR (400 MHz, DMSO) δ 8.99 – 8.68 (m, 1H), 8.59 – 8.28 (m, 1H), 8.18 – 7.90 (m, 1H), 7.79 – 7.44 (m, 3H), 7.44 – 6.86 (m, 1H), 6.80 – 6.66 (m, 3H), 6.64 – 6.25 (m, 3H), 4.82 – 4.16 (m, 3H), 4.19 – 3.94 (m, 1H), 3.82 – 3.39 (m, 2H), 3.33 – 2.67 (m, 8H), 1.65 – 1.20 (m, 12H) ppm.

^{13}C NMR (101 MHz, DMSO) δ 170.7, 170.5, 163.9, 163.7, 162.8, 155.0, 154.2, 154.2, 154.1, 150.2, 150.1, 149.9, 143.8, 143.7, 141.7, 139.7, 139.6, 138.5, 138.4, 137.9, 137.7, 137.6, 136.4, 136.3, 136.2, 136.0, 135.6, 135.6, 134.8, 134.6, 132.8, 132.6, 131.8, 131.7, 131.7, 131.6, 131.4, 131.3, 130.9, 130.8, 130.5, 130.4, 128.6, 127.8, 127.5, 125.8, 125.7, 125.6, 125.4, 121.4, 121.2, 77.7, 77.4, 77.3, 59.8, 59.5, 59.3, 57.7, 54.5, 49.6, 49.1, 48.6, 48.3, 46.9, 46.5, 46.1, 37.3, 35.6, 35.5, 34.2, 34.1, 34.1, 34.0, 30.6, 28.6, 27.9, 27.7, 27.4, 20.3, 17.9, 16.7, 16.3, 14.3, 13.6 ppm.(including two rotamers)

HRMS (ESI): *m/z* $[M+H]^+$ calcd for $C_{38}H_{40}N_4O_4Na$: 639.2942; found: 639.2933.

$[\alpha]^D{}_{20}$ = 33.6 (c = 1, $CHCl_3$);

m.p.: 86-90 °C.

($1^{3a}S,1^{9b}R$)-4^2-bromo-1^4-(quinolin-8-yl)-$1^1,1^3,1^{3a},1^4,1^5,1^{9b}$-hexahydro-1(6,9)-furo[3,4-*c*]isoquinolina-4(1,4)-benzenacyclohexaphan-1^5-one (22)

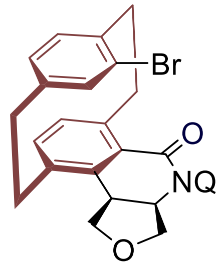

Prepared according to general procedure **3** for 48h on a 0.15 mmol scale, column chromatography (ethyl acetate/*n*-hexane = 1:3 to 1:1) afforded the compound **22** as a white solid (37 mg, 0.0705 mmol, 47%), with >20:1 dr, 99% ee and chiral starting material (S_P)-**1b** as a white solid (33 mg, 0.072 mmol, 48%), with 92% ee.

^{1}H NMR (400 MHz, $CDCl_3$) δ 8.72 (dd, J = 4.2, 1.8 Hz, 1H), 8.17 (dd, J = 8.3, 1.8 Hz, 1H), 7.90 (dd, J = 7.3, 1.5 Hz, 1H), 7.84 (dd, J = 8.3, 1.5 Hz, 1H), 7.68 (dd, J = 8.4, 7.2 Hz, 1H), 7.34 (dd, J = 8.3, 4.2 Hz, 1H), 6.81 – 6.63 (m, 3H), 6.55 (d, J = 7.7 Hz, 1H), 6.47 (d, J = 1.7 Hz, 1H), 5.10 – 4.97 (m, 2H), 4.85 (ddd, J = 12.6, 10.0, 2.5 Hz, 1H), 4.55 (dd, J = 8.1, 7.1 Hz, 1H), 3.82 – 3.54 (m, 4H), 3.29 – 2.94 (m, 5H), 2.87 (ddd, J = 12.5, 10.3, 5.6 Hz, 1H) ppm;

^{13}C NMR (101 MHz, $CDCl_3$) δ 165.4 (C_q), 150.2 (CH), 144.6 (C_q), 144.1 (C_q), 140.2 (C_q), 140.1 (C_q), 137.3 (C_q), 136.5 (CH), 136.4 (CH), 136.3 (CH), 134.8 (CH), 134.7 (CH), 134.7 (CH), 132.0 (CH), 131.9 (CH), 129.6 (C_q), 127.8 (CH), 127.1 (C_q), 126.7 (CH), 124.7 (C_q), 121.3 (CH), 72.1 (CH_2), 71.2 (CH_2), 61.5 (CH), 41.3 (CH), 34.8 (CH_2), 34.8 (CH_2), 34.7 (CH_2), 31.6 (CH_2) ppm;

HRMS (ESI): *m/z* $[M+H]^+$ calcd for $C_{30}H_{25}BrN_2O_2$: 525.1172; found: 525.1172.

$[\alpha]^D{}_{20}$ = –22.4 (c = 0.50, $CHCl_3$).

R_t (AD-3 column, *n*-hexane/*i*-PrOH 50/50, 1.0 mL/min, 273.0 nm): tr(major) = 11.2 min, tr(minor) = 29.9 min, 99% ee.

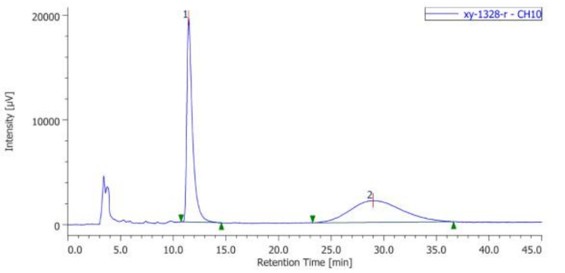

#	Peak Name	CH	tR [min]	Area [µV·sec]	Height [µV]	Area%
1	Unknown	10	11.470	729946	19501	50.578
2	Unknown	10	28.973	713267	2109	49.422

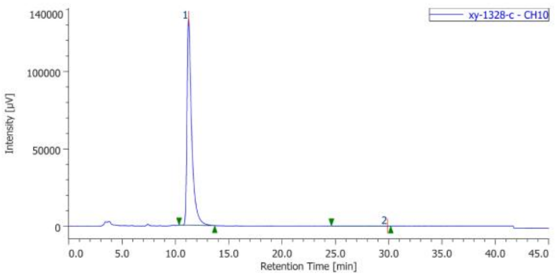

#	Peak Name	CH	tR [min]	Area [µV·sec]	Height [µV]	Area%
1	Unknown	10	11.233	4068821	133397	99.997
2	Unknown	10	29.883	116	21	0.003

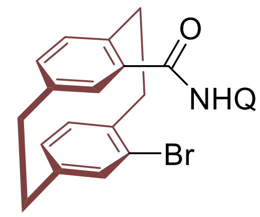

(S_p)-**1b**

^{1}H NMR (300 MHz, $CDCl_3$) δ 10.41 (s, 1H), 8.97 (dd, J = 7.6, 1.4 Hz, 1H), 8.85 (dd, J = 4.2, 1.7 Hz, 1H), 8.17 (dd, J = 8.3, 1.7 Hz, 1H), 7.61 (t, J = 7.9 Hz, 1H), 7.52 (dd, J = 8.3, 1.4 Hz, 1H), 7.46 (dd, J = 8.3, 4.2 Hz, 1H), 7.38 (d, J = 1.8 Hz, 1H), 6.76 – 6.59 (m, 5H), 4.34 – 4.16 (m, 1H), 3.58 (ddd, J = 13.5, 9.6, 2.6 Hz, 1H), 3.33 (ddd, J = 13.2, 9.9, 2.6 Hz, 1H), 3.24 – 2.99 (m, 4H), 2.91 (ddd, J = 13.5, 10.0, 6.0 Hz, 1H) ppm;

^{13}C NMR (75 MHz, $CDCl_3$) δ 165.9 (C_q), 148.3 (CH), 141.3 (C_q), 139.4 (C_q), 139.1 (C_q), 138.9 (C_q), 138.8 (C_q), 136.6 (CH), 136.3 (CH), 136.3 (CH), 135.9 (CH), 135.2 (C_q), 135.2 (C_q), 134.9 (CH), 131.3 (CH), 131.0 (CH), 128.1 (C_q), 127.6 (CH), 127.0 (C_q), 121.6 (CH), 121.3 (CH), 116.3 (CH), 36.5 (CH_2), 35.1 (CH_2), 34.7 (CH_2), 32.4 (CH_2) ppm;

$[\alpha]^{D}_{20}$ = +6.8 (c = 0.50, $CHCl_3$).

R_t (AD-3 column, *n*-hexane/*i*-PrOH 80/20, 1.0 mL/min, 273.0 nm): tr(major) = 17.6 min, tr(minor) = 13.6 min, 92% ee.

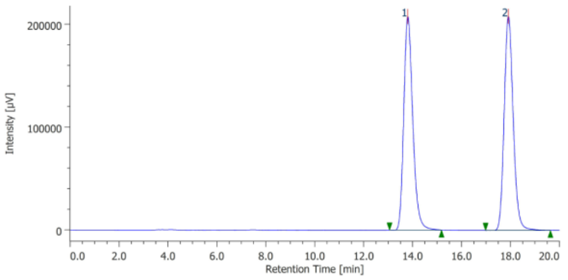

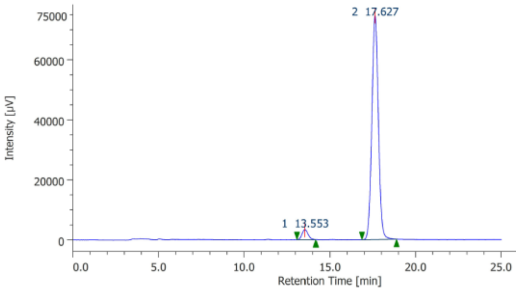

#	Peak Name	CH	tR [min]	Area [µV·sec]	Height [µV]	Area%
1	Unknown	9	13.797	5316910	207638	49.842
2	Unknown	9	17.910	5350591	208026	50.158

#	Peak Name	CH	tR [min]	Area [µV·sec]	Height [µV]	Area%
1	Unknown	10	13.553	83526	3393	4.067
2	Unknown	10	17.627	1970396	74517	95.933

(1^{3a}*S*,1^{9b}*R*)-1^{4}-(quinolin-8-yl)-4^{2}-(*p*-toly)-1^{1},1^{3},1^{3a},1^{4},1^{5},1^{9b}-hexahydro-1(6,9)-furo[3,4-*c*]isoquinolina-4(1,4)-benzenacyclohexaphan-1^{5}-one (23)

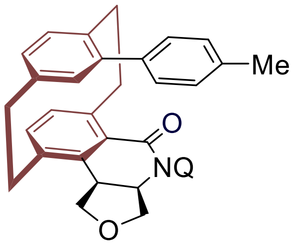

Prepared according to general procedure **3** for 72h on a 0.15 mmol scale, column chromatography (ethyl acetate/*n*-hexane = 1:3 to 1:1) afforded the compound **23** as a white solid (35 mg, 0.065 mmol, 43%), with >20:1 dr, 99% ee and chiral starting material (S_P)-**1c** as a white solid (34 mg, 0.073mmol, 48%), with 96% ee.

^{1}H NMR (400 MHz, $CDCl_3$) δ 8.70 (dd, *J* = 4.2, 1.7 Hz, 1H), 8.15 (dd, *J* = 8.4, 1.8 Hz, 1H), 7.84 (dd, *J* = 8.2, 1.6 Hz, 1H), 7.78 (dd, *J* = 7.3, 1.5 Hz, 1H), 7.67 (dd, *J* = 8.2, 7.3 Hz, 1H), 7.41 – 7.33 (m, 2H), 7.32 (dd, *J* = 8.3, 4.2 Hz, 1H), 7.27 – 7.20 (m, 2H), 6.86 (d, *J* = 7.7 Hz, 1H), 6.81 (d, *J* = 7.6 Hz, 1H), 6.77 – 6.66 (m, 2H), 6.42 (d, *J* = 2.0 Hz, 1H), 4.99 (dd, *J* = 5.1, 3.2 Hz, 1H), 4.75 (dd, *J* = 10.6, 7.5 Hz, 1H), 4.52 – 4.28 (m, 2H), 3.81 – 3.45 (m, 4H), 3.34 – 3.13 (m, 3H), 3.11 – 2.73 (m, 3H), 2.41 (s, 3H) ppm;

^{13}C NMR (101 MHz, $CDCl_3$) δ 165.3 (C_q), 150.2 (CH), 144.8 (C_q), 144.5 (C_q), 141.1 (C_q), 138.4 (C_q), 137.8 (C_q), 137.7 (C_q), 137.4 (C_q), 136.8 (CH), 136.4 (C_q), 136.2 (CH), 136.2 (CH), 135.5 (CH), 133.9 (C_q), 132.0 (CH), 131.6 (CH), 130.8 (CH), 129.7 (C_q), 129.5 (CH), 128.9 (CH), 128.0 (CH), 126.3 (CH), 124.9 (C_q), 121.2 (CH), 72.4 (CH_2), 71.6 (CH_2), 61.5 (CH), 40.9 (CH), 35.2 (CH_2), 34.8 (CH_2), 33.9 (CH_2), 32.3 (CH_2), 21.3 (CH_3) ppm;

HRMS (ESI): *m/z* $[M+H]^+$ calcd for $C_{37}H_{32}N_2O_2$: 537.2537; found: 537.2540.

$[\alpha]^{D}_{20}$ = +5.4 (c = 0.50, $CHCl_3$).

R_t (AD-3 column, *n*-hexane/*i*-PrOH 60/40, 1.0 mL/min, 273.0 nm): tr(major) = 8.5 min, tr(minor) = 18.6 min, 99% ee.

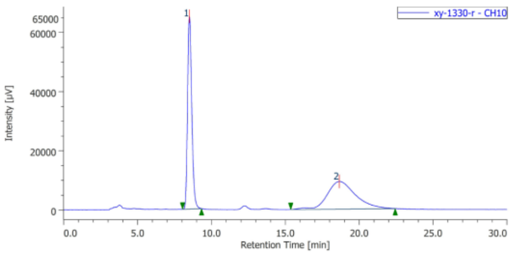

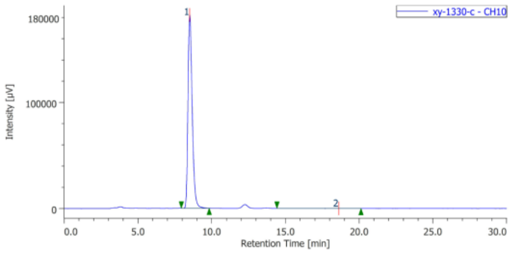

#	Peak Name	CH	tR [min]	Area [μV·sec]	Height [μV]	Area%
1	Unknown	10	8.503	1291951	65101	50.970
2	Unknown	10	18.650	1242771	9367	49.030

#	Peak Name	CH	tR [min]	Area [μV·sec]	Height [μV]	Area%
1	Unknown	10	8.507	3647182	181878	99.866
2	Unknown	10	18.620	4907	52	0.134

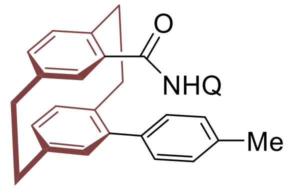

(S_p)-**1c**

^{1}H NMR (300 MHz, $CDCl_3$) δ 10.11 (s, 1H), 8.78 (dd, J = 4.2, 1.7 Hz, 1H), 8.46 (dd, J = 6.9, 2.2 Hz, 1H), 8.17 (dd, J = 8.3, 1.7 Hz, 1H), 7.59 – 7.47 (m, 2H), 7.44 (dd, J = 8.3, 4.2 Hz, 1H), 7.35 (d, J = 1.8 Hz, 1H), 7.31 – 7.26 (m, 2H), 6.92 – 6.67 (m, 6H), 6.59 (dd, J = 7.7, 1.9 Hz, 1H), 4.01 – 3.70 (m, 2H), 3.41 – 2.82 (m, 6H), 2.20 (s, 3H) ppm;

^{13}C NMR (75 MHz, $CDCl_3$) δ 165.0 (C_q), 148.1 (CH), 142.5 (C_q), 142.1 (C_q), 140.0 (C_q), 139.0 (C_q), 138.8 (C_q), 137.8 (C_q), 137.8 (C_q), 136.8 (CH), 136.3 (CH), 135.9 (C_q), 135.5 (CH), 135.4 (CH), 135.1 (C_q), 132.5 (C_q), 132.1 (CH), 131.9 (CH), 130.6 (CH), 129.4 (CH), 128.9 (CH), 127.9 (C_q), 127.2 (CH), 121.5 (CH), 121.0 (CH), 116.7 (CH), 37.1 (CH_2), 35.3 (CH_2), 35.2 (CH_2), 33.8 (CH_2), 21.0 (CH_3) ppm;

$[\alpha]^{D}_{20}$ = +43.8 (c = 0.50, $CHCl_3$).

R_t (OD-3 column, *n*-hexane/*i*-PrOH 80/20, 1.0 mL/min, 273.0 nm): tr(major) = 10.7 min, tr(minor) = 16.6 min, 96% ee.

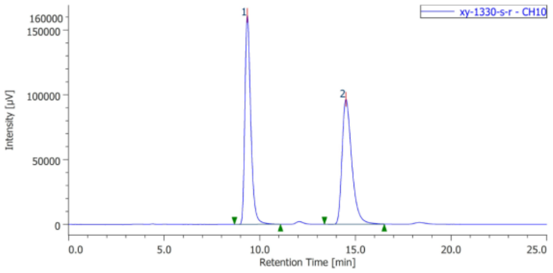

#	Peak Name	CH	tR [min]	Area [µV·sec]	Height [µV]	Area%
1	Unknown	10	9.343	3419530	161074	50.194
2	Unknown	10	14.500	3393089	96344	49.806

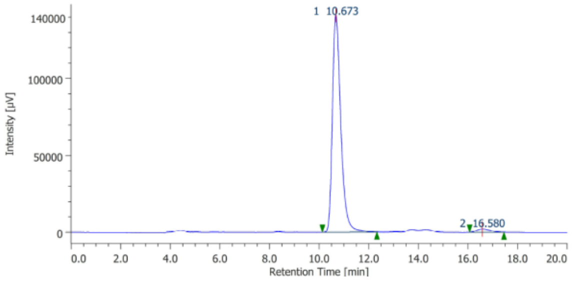

#	Peak Name	CH	tR [min]	Area [µV·sec]	Height [µV]	Area%
1	Unknown	10	10.673	3326021	141362	97.860
2	Unknown	10	16.580	72723	2015	2.140

($1^{3a}S$,$1^{9b}R$)-4^2-(naphthalen-1-yl)-1^4-(quinolin-8-yl)-1^1,1^3,1^{3a},1^4,1^5,1^{9b}-hexahydro-1(6,9)-furo[3,4-*c*]isoquinolina-4(1,4)-benzenacyclohexaphan-1^5-one (24)

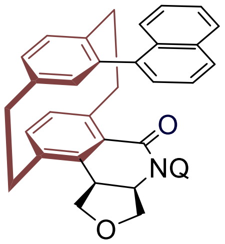

Prepared according to general procedure **3** for 72h on a 0.15 mmol scale, column chromatography (ethyl acetate/*n*-hexane = 1:3 to 1:1) afforded the compound **24** as a white solid (33 mg, 0.057 mmol, 38%), with >20:1 dr, 99% ee and chiral starting material (S_P)-**1d** as a white solid (39 mg, 0.073mmol, 52%), with 90% ee.

^{1}H NMR (400 MHz, $CDCl_3$) δ 8.72 (dd, *J* = 4.1, 1.8 Hz, 1H), 8.17 (dd, *J* = 8.3, 1.8 Hz, 1H), 8.01 (dd, *J* = 7.2, 1.5 Hz, 1H), 7.96 – 7.75 (m, 5H), 7.75 – 7.67 (m, 1H), 7.44 – 7.29 (m, 2H), 7.25 – 7.18 (m, 2H), 6.98 (d, *J* = 7.6 Hz, 1H), 6.92 – 6.83 (m, 2H), 6.79 (d, *J* = 7.6 Hz, 1H), 6.42 (d, *J* = 1.9 Hz, 1H), 5.01 (dd, *J* = 5.2, 3.2 Hz, 1H), 4.87 (dd, *J* = 10.6, 7.6 Hz, 1H), 4.51 – 4.31 (m, 2H), 3.82 (d, *J* = 10.2 Hz, 1H), 3.77 – 3.61 (m, 2H), 3.37 (ddd, *J* = 13.3, 9.6, 3.8 Hz, 1H), 3.27 – 3.15 (m, 2H), 3.11 – 2.80 (m, 4H) ppm;

^{13}C NMR (101 MHz, $CDCl_3$) δ 165.9 (C_q), 150.3 (CH), 145.2 (C_q), 144.4 (C_q), 140.4 (C_q), 138.2 (C_q), 137.7 (C_q), 137.7 (C_q), 137.7 (C_q), 137.4 (C_q), 137.2 (CH), 136.3 (CH), 136.3 (CH), 134.7 (CH), 134.0 (C_q), 133.6 (C_q), 132.6 (C_q), 132.2 (CH), 132.2 (CH), 131.9 (CH), 129.8 (C_q), 128.2 (CH), 128.1 (CH), 127.8 (CH), 126.5 (CH), 126.4 (CH), 125.7 (CH), 125.6 (CH), 125.3 (CH), 125.0 (CH), 124.7 (C_q), 121.3 (CH), 72.7 (CH_2), 71.9 (CH_2), 61.8 (CH), 40.7 (CH), 35.3 (CH_2), 34.5 (CH_2), 33.9 (CH_2), 32.3 (CH_2) ppm;

HRMS (ESI): *m/z* $[M+H]^+$ calcd for $C_{40}H_{32}N_2O_2$: 573.2537; found: 573.2541.

$[\alpha]^D_{20}$ = –11.6 (c = 0.50, $CHCl_3$).

R_t (AD-3 column, *n*-hexane/*i*-PrOH 60/40, 1.0 mL/min, 273.0 nm): tr(major) = 11.0 min, tr(minor) = 18.9 min, 99% ee.

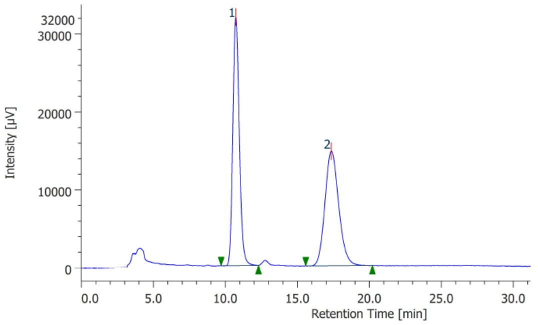

#	Peak Name	CH	tR [min]	Area [µV·sec]	Height [µV]	Area%
1	Unknown	10	10.727	991996	31877	50.492
2	Unknown	10	17.347	972665	14698	49.508

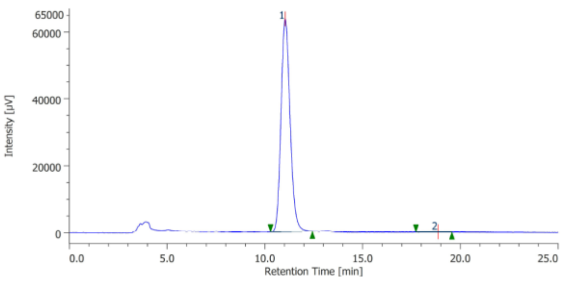

#	Peak Name	CH	tR [min]	Area [µV·sec]	Height [µV]	Area%
1	Unknown	10	11.037	2050686	63613	99.752
2	Unknown	10	18.850	5088	112	0.248

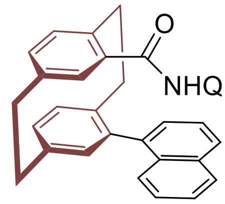

(S_p)-**1d**

^{1}H NMR (400 MHz, $CDCl_3$) δ 10.12 (s, 1H), 8.67 – 8.52 (m, 2H), 8.15 (dd, J = 8.3, 1.7 Hz, 1H), 7.83 (dd, J = 8.5, 1.2 Hz, 1H), 7.77 – 7.69 (m, 2H), 7.60 – 7.49 (m, 3H), 7.45 (d, J = 1.8 Hz, 1H), 7.42 – 7.28 (m, 3H), 7.07 (dd, J = 8.2, 7.1 Hz, 1H), 6.96 – 6.86 (m, 2H), 6.80 (d, J = 7.7 Hz, 1H), 6.77 – 6.69 (m, 2H), 3.79 (ddd, J = 13.1, 8.4, 2.8 Hz, 1H), 3.44 – 3.03 (m, 6H), 3.01 – 2.89 (m, 1H) ppm;

^{13}C NMR (101 MHz, $CDCl_3$) δ 165.3 (C_q), 148.0 (CH), 141.8 (C_q), 140.3 (C_q), 140.1 (C_q), 139.3 (C_q), 139.2 (C_q), 138.7 (C_q), 138.7 (C_q), 136.8 (CH), 136.2 (CH), 135.7 (CH), 135.1 (C_q), 134.2 (CH), 133.4 (C_q), 132.9 (CH), 132.2 (CH), 132.1 (C_q), 131.6 (CH), 131.3 (C_q), 128.2 (CH), 128.2 (CH), 127.9 (C_q), 127.4 (CH), 127.2 (CH), 125.7 (CH), 125.6 (CH), 125.2 (CH), 121.5 (CH), 121.1 (CH), 116.6 (CH), 36.8 (CH_2), 35.3 (CH_2), 35.2 (CH_2), 34.5 (CH_2) ppm;

$[\alpha]^{D}_{20}$ = –159.2 (c = 0.25, $CHCl_3$).

R_t (OD-3 column, *n*-hexane/*i*-PrOH 80/20, 1.0 mL/min, 273.0 nm): tr(major) = 14.3 min, tr(minor) = 9.9 min, 90% ee.

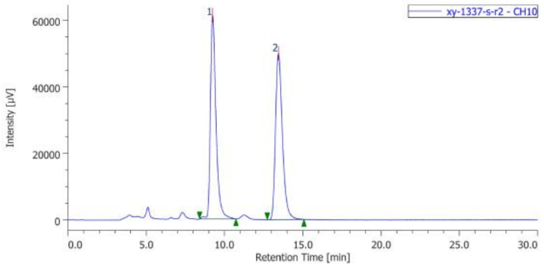

#	Peak Name	CH	tR [min]	Area [µV·sec]	Height [µV]	Area%
1	Unknown	10	9.240	1517857	61195	49.799
2	Unknown	10	13.447	1530124	50011	50.201

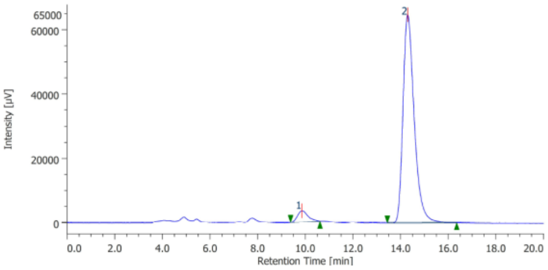

#	Peak Name	CH	tR [min]	Area [µV·sec]	Height [µV]	Area%
1	Unknown	10	9.853	106282	3509	4.800
2	Unknown	10	14.293	2107952	64702	95.200

($1^{3a}S$,$1^{9b}R$)-4^2-(benzofuran-2-yl)-1^4-(quinolin-8-yl)-1^1,1^3,1^{3a},1^4,1^5,1^{9b}-hexahydro-1(6,9)-furo[3,4-*c*]isoquinolina-4(1,4)-benzenacyclohexaphan-1^5-one (25)

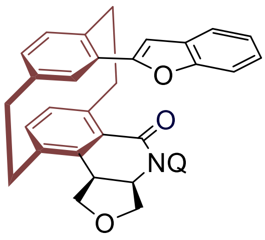

Prepared according to general procedure **3** for 72h on a 0.15 mmol scale, column chromatography (ethyl acetate/*n*-hexane = 1:3 to 1:1) afforded the compound **25** as a white solid (36 mg, 0.0705 mmol, 43%), with >20:1 dr, 99% ee and chiral starting material (S_P)-**1e** as a white solid (36 mg, 0.074 mmol, 49%), with 99% ee.

^{1}H NMR (400 MHz, $CDCl_3$) δ 8.92 (dd, J = 4.2, 1.7 Hz, 1H), 8.34 (dd, J = 8.3, 1.8 Hz, 1H), 8.00 – 7.81 (m, 3H), 7.75 – 7.48 (m, 4H), 7.35 (dd, J = 8.2, 7.3 Hz, 1H), 7.13 (d, J = 7.8 Hz, 1H), 7.04 (d, J = 7.7 Hz, 1H), 6.96 (ddd, J = 7.3, 4.2, 2.0 Hz, 3H), 5.31 (dd, J = 11.1, 7.4 Hz, 1H), 5.20 (dd, J = 4.6, 3.3 Hz, 1H), 4.99 – 4.79 (m, 2H), 4.45 (ddd, J = 13.4, 9.7, 5.5 Hz, 1H), 3.90 – 3.79 (m, 2H), 3.68 (d, J = 10.1 Hz, 1H), 3.54 – 3.37 (m, 5H), 3.21 (ddd, J = 12.7, 10.0, 5.5 Hz, 1H) ppm;

^{13}C NMR (101 MHz, $CDCl_3$, major rotamer) δ 164.3 (C_q), 154.6 (C_q), 154.1 (C_q), 149.9 (CH), 144.4 (C_q), 143.9 (C_q), 138.5 (C_q), 138.3 (C_q), 137.7 (C_q), 136.4 (C_q), 136.3 (CH), 136.3 (CH), 136.2 (CH), 135.9 (CH), 134.5 (C_q), 133.2 (CH), 132.3 (CH), 130.0 (C_q), 129.7 (C_q), 129.3 (CH), 127.4 (CH), 126.3 (CH), 125.5 (C_q), 124.1 (CH), 122.8 (CH), 121.4 (CH), 121.0 (CH), 111.2 (CH), 105.8 (CH), 71.8 (CH_2), 71.2 (CH_2), 61.2 (CH), 41.5 (CH), 35.2 (CH_2), 35.1 (CH_2), 33.9 (CH_2), 31.8 (CH_2) ppm;

HRMS (ESI): *m/z* $[M+H]^+$ calcd for $C_{38}H_{30}N_2O_3$: 563.2329; found: 563.2333.

$[\alpha]^D{}_{20}$ = +147.8 (c = 0.50, $CHCl_3$).

R_t (OD-3 column, *n*-hexane/*i*-PrOH 80/20, 1.0 mL/min, 273.0 nm): tr(major) = 9.7 min, tr(minor) = 25.8 min, 99% ee.

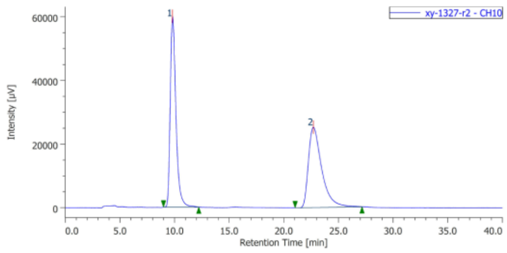

#	Peak Name	CH	tR [min]	Area [µV·sec]	Height [µV]	Area%
1	Unknown	10	9.817	2161637	59932	50.429
2	Unknown	10	22.700	2124886	25250	49.571

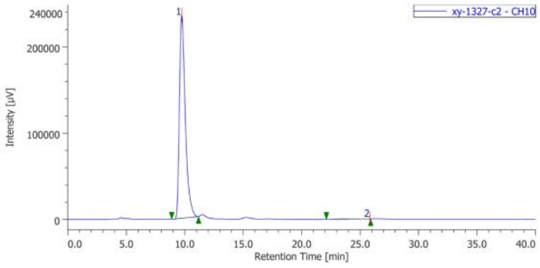

#	Peak Name	CH	tR [min]	Area [µV·sec]	Height [µV]	Area%
1	Unknown	10	9.743	8218221	235358	99.620
2	Unknown	10	25.840	31339	27	0.380

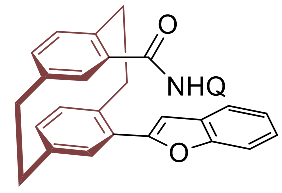

(S_p)-**1e**

^{1}H NMR (300 MHz, $CDCl_3$) δ 10.01 (s, 1H), 8.81 (dd, J = 4.2, 1.7 Hz, 1H), 8.10 (dd, J = 8.3, 1.7 Hz, 1H), 7.91 (dd, J = 7.8, 1.3 Hz, 1H), 7.43 (dd, J = 8.3, 4.2 Hz, 1H), 7.36 – 7.08 (m, 5H), 7.02 – 6.86 (m, 3H), 6.82 – 6.71 (m, 4H), 6.63 (dd, J = 7.8, 1.9 Hz, 1H), 4.31 – 3.98 (m, 2H), 3.32 – 3.07 (m, 6H) ppm;

^{13}C NMR (75 MHz, $CDCl_3$) δ 165.1 (C_q), 155.8 (C_q), 154.4 (C_q), 147.9 (CH), 140.9 (C_q), 139.9 (C_q), 139.6 (C_q), 138.6 (C_q), 137.8 (C_q), 136.5 (CH), 136.2 (CH), 136.0 (CH), 135.6 (CH), 134.7 (C_q), 134.5 (C_q), 132.8 (CH), 131.5 (CH), 131.1 (C_q), 130.9 (CH), 129.2 (C_q), 127.8 (C_q), 127.4 (CH), 123.6 (CH), 122.2 (CH), 121.3 (CH), 120.7 (CH), 120.5 (CH), 116.4 (CH), 110.8 (CH), 104.8 (CH), 35.2 (CH_2), 35.1 (CH_2), 34.8 (CH_2) ppm;

$[\alpha]^{D}_{20}$ = –83.2 (c = 0.25, $CHCl_3$).

R_t (AD-3 column, n-hexane/i-PrOH 80/20, 1.0 mL/min, 273.0 nm): tr(major) = 16.8 min, tr(minor) = 10.6 min, 88% ee.

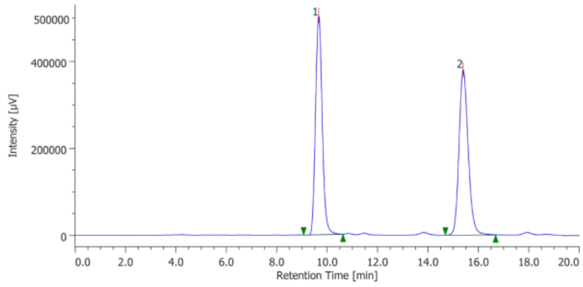

#	Peak Name	CH	tR [min]	Area [µV·sec]	Height [µV]	Area%
1	Unknown	9	9.663	9352888	504243	49.531
2	Unknown	9	15.390	9530132	379999	50.469

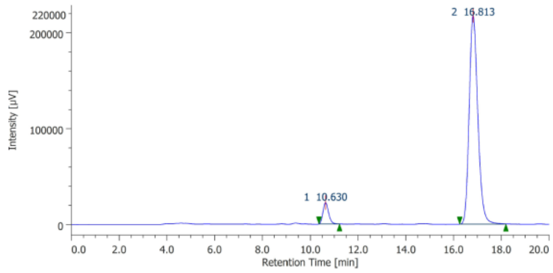

#	Peak Name	CH	tR [min]	Area [µV·sec]	Height [µV]	Area%
1	Unknown	10	10.630	365035	22385	6.243
2	Unknown	10	16.813	5481690	217452	93.757

(1^{3a}*S*,1^{9b}*R*)-1^{4}-(quinolin-8-yl)-4^{2}-(thiophen-2-yl)-1^{1},1^{3},1^{3a},1^{4},1^{5},1^{9b}-hexahydro-1(6,9)-furo[3,4-*c*]isoquinolina-4(1,4)-benzenacyclohexaphan-1^{5}-one (26)

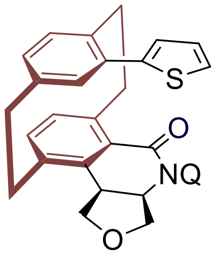

Prepared according to general procedure **3** for 48h on a 0.15 mmol scale, column chromatography (ethyl acetate/*n*-hexane = 1:3 to 1:1) afforded the compound **26** as a white solid (36 mg, 0.068 mmol, 45%), with >20:1 dr, 99% ee and chiral starting material (***S****p*)-**1f** as a white solid (32 mg, 0.070 mmol, 46%), with 99% ee.

^{1}H NMR (400 MHz, $CDCl_3$) δ 8.69 (dd, *J* = 4.2, 1.8 Hz, 1H), 8.14 (dd, *J* = 8.2, 1.8 Hz, 1H), 7.82 (dd, *J* = 7.2, 2.5 Hz, 1H), 7.70 – 7.48 (m, 2H), 7.40 – 7.27 (m, 3H), 7.14 (dd, *J* = 5.1, 3.6 Hz, 1H), 6.83 (d, *J* = 7.7 Hz, 1H), 6.78 (d, *J* = 7.6 Hz, 1H), 6.74 – 6.64 (m, 2H), 6.51 (d, *J* = 1.9 Hz, 1H), 5.05 – 4.89 (m, 1H), 4.71 – 4.55 (m, 2H), 4.49 (dd, *J* = 8.4, 7.3 Hz, 1H), 3.88 (ddd, *J* = 13.3, 9.7, 5.2 Hz, 1H), 3.68 – 3.54 (m, 3H), 3.27 – 2.79 (m, 6H) ppm; **^{13}C NMR** (101 MHz, $CDCl_3$) δ 165.10 (C_q), 150.2 (CH), 144.5 (C_q), 142.8 (C_q), 138.3 (C_q), 138.2 (C_q), 137.4 (C_q), 137.3 (C_q), 136.6 (CH), 136.3 (CH), 136.2 (CH), 135.7 (CH), 134.3 (C_q), 133.9 (C_q), 132.1 (CH), 132.1 (CH), 131.4 (CH), 129.6 (C_q), 127.8 (CH), 127.8 (CH), 126.9 (CH), 126.4 (CH), 125.0 (CH), 124.8 (C_q), 121.2 (CH), 72.2 (CH_2), 71.7 (CH_2), 61.4 (CH), 41.0 (CH), 35.0 (CH_2), 35.0 (CH_2), 33.8 (CH_2), 31.9 (CH_2) ppm;

HRMS (ESI): *m/z* $[M+H]^+$ calcd for $C_{34}H_{28}N_2O_2S$: 529.1944; found: 529.1948.

$[\alpha]^{D}_{20}$ = –54.2 (c = 0.50, $CHCl_3$).

R_t (OD-3 column, *n*-hexane/*i*-PrOH 80/20, 1.0 mL/min, 273.0 nm): tr(major) = 12.7 min, tr(minor) = 18.9 min, 99% ee.

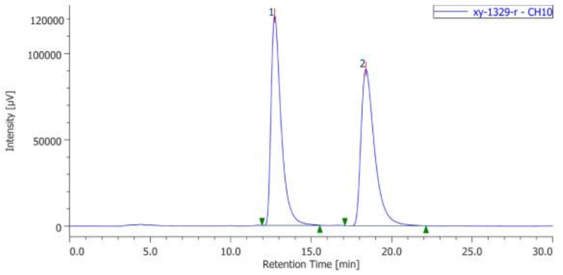

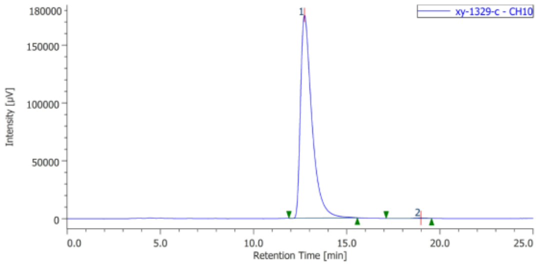

#	Peak Name	CH	tR [min]	Area [µV·sec]	Height [µV]	Area%
1	Unknown	10	12.727	5292302	121446	49.943
2	Unknown	10	18.393	5304321	90656	50.057

#	Peak Name	CH	tR [min]	Area [µV·sec]	Height [µV]	Area%
1	Unknown	10	12.727	7448090	175507	99.855
2	Unknown	10	18.967	10850	261	0.145

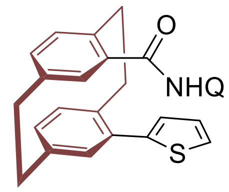

(S_p)-**1f**

^{1}H NMR (300 MHz, $CDCl_3$) δ 10.07 (s, 1H), 8.78 (dd, *J* = 4.2, 1.7 Hz, 1H), 8.51 (dd, *J* = 7.3, 1.8 Hz, 1H), 8.15 (dd, *J* = 8.3, 1.7 Hz, 1H), 7.59 – 7.44 (m, 2H), 7.43 (dd, *J* = 8.2, 4.2 Hz, 1H), 7.29 (d, *J* = 1.8 Hz, 1H), 7.08 (dd, *J* = 3.6, 1.2 Hz, 1H), 7.02 (dd, *J* = 5.1, 1.2 Hz, 1H), 6.89 – 6.80 (m, 2H), 6.79 – 6.67 (m, 3H), 6.58 (dd, *J* = 7.8, 1.9 Hz, 1H), 4.20 (ddd, *J* = 13.0, 9.3, 5.8 Hz, 1H), 4.04 (ddd, *J* = 12.3, 9.3, 2.6 Hz, 1H), 3.39 – 2.92 (m, 6H) ppm;

^{13}C NMR (75 MHz, $CDCl_3$) δ 165.30 (C_q), 148.06 (CH), 143.66 (C_q), 141.70 (C_q), 139.93 (C_q), 139.47 (C_q), 138.78 (C_q), 137.80 (C_q), 136.59 (CH), 136.25 (CH), 135.58 (CH), 135.47 (CH), 135.17 (C_q), 135.10 (C_q), 133.17 (C_q), 132.34 (CH), 131.76 (CH), 131.64 (CH), 127.93 (C_q), 127.37 (CH), 127.27 (CH), 126.19 (CH), 125.00 (CH), 121.46 (CH), 120.94 (CH), 116.50 (CH), 36.40 (CH_2), 35.21 (CH_2), 35.00 (CH_2), 34.15 (CH_2) ppm;

$[\alpha]^{D}_{20}$ = +103.0 (c = 0.50, $CHCl_3$).

R_t (OD-3 column, *n*-hexane/*i*-PrOH 80/20, 1.0 mL/min, 273.0 nm): tr(major) = 10.1 min, tr(minor) = 12.4 min, 99% ee.

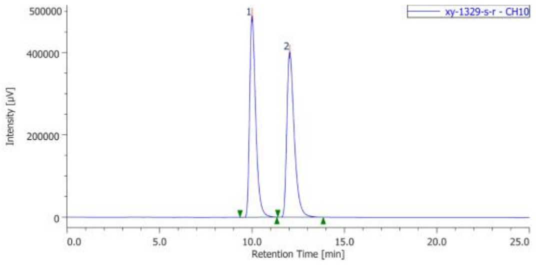

#	Peak Name	CH	tR [min]	Area [µV·sec]	Height [µV]	Area%
1	Unknown	10	9.997	11189642	490897	50.040
2	Unknown	10	12.033	11171707	402030	49.960

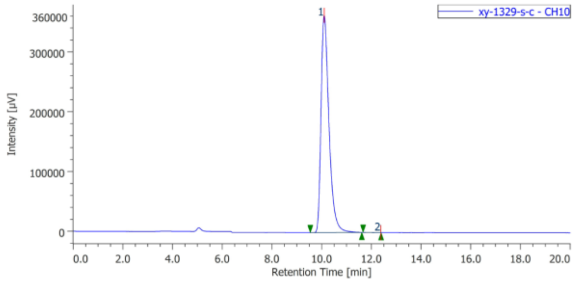

#	Peak Name	CH	tR [min]	Area [µV·sec]	Height [µV]	Area%
1	Unknown	10	10.100	8051713	362808	99.963
2	Unknown	10	12.380	3018	13	0.037

($1^{3a}S$,$1^{9b}R$)-4^2-(diphenylphosphoryl)-1^4-(quinolin-8-yl)-1^1,1^3,1^{3a},1^4,1^5,1^{9b}-hexahydro-1(6,9)-furo[3,4-*c*]isoquinolina-4(1,4)-benzenacyclohexaphan-1^5-one (27)

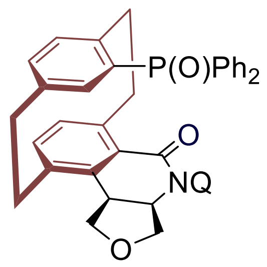

Prepared according to general procedure **3** for 72h on a 0.15 mmol scale, column chromatography (ethyl acetate/*n*-hexane = 1:1 to 3:1) afforded the compound **27** as a white solid (31 mg, 0.048 mmol, 32%), with >20:1 dr, 99% ee and chiral starting material **(S_p)-1g** as a white solid (44 mg, 0.0765mmol, 51%), with 78% ee.

^{1}H NMR (400 MHz, $CDCl_3$) δ 9.02 (dd, *J* = 6.8, 2.0 Hz, 1H), 8.70 (dd, *J* = 4.2, 1.7 Hz, 1H), 8.16 (dd, *J* = 8.3, 1.8 Hz, 1H), 7.98 – 7.83 (m, 2H), 7.80 – 7.68 (m, 2H), 7.66 – 7.56 (m, 2H), 7.48 – 7.28 (m, 7H), 6.91 – 6.80 (m, 2H), 6.76 (d, *J* = 7.6 Hz, 1H), 6.69 (d, *J* = 7.6 Hz, 1H), 6.42 (d, *J* = 1.6 Hz, 1H), 5.27 (dd, *J* = 11.3, 7.0 Hz, 1H), 5.19 – 4.96 (m, 1H), 4.53 (dd, *J* = 11.5, 8.7 Hz, 1H), 4.08 (t, *J* = 7.5 Hz, 1H), 3.73 – 3.53 (m, 2H), 3.52 – 3.24 (m, 2H), 3.24 – 2.90 (m, 4H), 2.75 – 2.54 (m, 2H) ppm;

^{13}C NMR (101 MHz, $CDCl_3$) δ 163.6 (C_q), 149.7 (CH), 146.7 (d, J_{CP} = 8.3 Hz, CH), 144.7 (C_q), 144.0 (C_q), 137.5 (C_q), 137.2 (C_q), 137.2 (CH), 137.2 (CH), 137.1 (C_q), 136.3 (CH), 136.1 (C_q), 136.1 (CH), 135.2 (CH), 135.1 (C_q), 134.8 (CH), 134.7 (CH), 134.2 (C_q), 134.2 (CH), 132.4 (C_q), 132.0 (d, J_{CP} = 8.7 Hz, CH), 131.6 (d, J_{CP} = 9.2 Hz, CH), 131.4 (C_q), 131.0 (CH), 130.9 (CH), 130.9 (CH), 129.4 (C_q), 128.1 (d, J_{CP} = 3.5 Hz, CH), 128.0 (d, J_{CP} = 3.8 Hz, CH), 127.4 (CH), 127.2 (CH), 126.6 (C_q), 120.8 (CH), 72.2 (CH_2), 71.6 (CH_2), 61.3 (CH), 41.3 (CH), 37.7 (CH), 34.8 (CH_2), 34.7 (CH_2), 31.3 (CH_2) ppm;

^{31}P NMR (162 MHz, $CDCl_3$) δ 22.65 ppm.

HRMS (ESI): *m/z* $[M+H]^+$ calcd for $C_{42}H_{35}N_2O_3P$: 647.2458; found: 647.2459.

$[\alpha]^D{}_{20}$ = –22.6 (c = 0.50, $CHCl_3$).

R_t (OD-3 column, *n*-hexane/*i*-PrOH 70/30, 1.0 mL/min, 273.0 nm): tr(major) = 9.6 min, tr(minor) = 14.4 min, 99% ee.

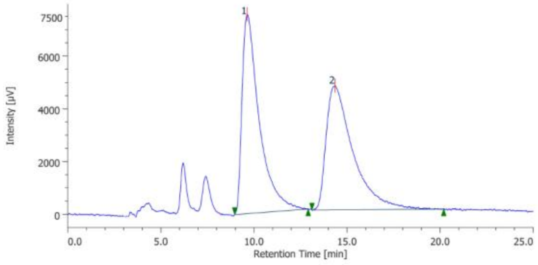

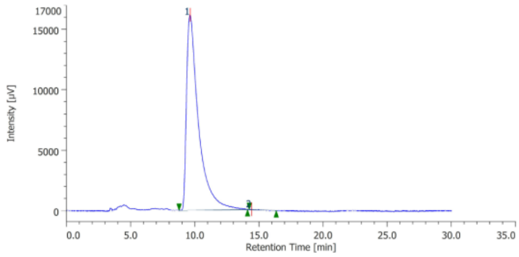

#	Peak Name	CH	tR [min]	Area [μV·sec]	Height [μV]	Area%
1	Unknown	10	9.633	485396	7553	50.751
2	Unknown	10	14.357	471037	4713	49.249

#	Peak Name	CH	tR [min]	Area [μV·sec]	Height [μV]	Area%
1	Unknown	10	9.637	1050239	16178	99.930
2	Unknown	10	14.433	736	42	0.070

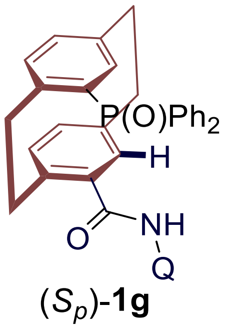

(S_p)-**1g**

^{1}H NMR (400 MHz, CDCl3) δ 10.04 (s, 1H), 9.25 (dd, J = 7.7, 1.3 Hz, 1H), 8.69 (dd, J = 4.2, 1.7 Hz, 1H), 8.12 (dd, J = 8.3, 1.7 Hz, 1H), 7.83 – 7.75 (m, 2H), 7.68 (t, J = 8.0 Hz, 1H), 7.55 – 7.42 (m, 2H), 7.41 – 7.30 (m, 4H), 7.28 – 7.16 (m, 5H), 6.83 (dd, J = 7.7, 1.8 Hz, 1H), 6.80 – 6.73 (m, 2H), 6.69 (dd, J = 7.8, 2.0 Hz, 1H), 6.36 (d, J = 1.8 Hz, 1H), 4.47 (ddd, J = 13.1, 9.8, 6.5 Hz, 1H), 3.73 – 3.54 (m, 1H), 3.42 – 3.31 (m, 1H), 3.26 – 3.14 (m, 2H), 2.97 (ddd, J = 13.2, 10.5, 6.4 Hz, 1H), 2.89 – 2.72 (m, 2H) ppm;

^{13}C NMR (101 MHz, $CDCl_3$) δ 165.4 (C_q), 147.8 (CH), 145.9 (d, J_{CP} = 7.2 Hz, C_q), 139.5 (C_q), 139.4 (C_q), 139.4 (C_q), 139.1 (C_q), 138.8 (C_q), 137.8 (C_q), 136.5 (C_q), 136.3 (CH), 136.2 (CH), 136.0 (CH), 135.9 (CH), 135.6 (CH), 135.6 (CH), 135.5 (C_q), 133.1 (CH), 133.0 (CH), 132.3 (C_q), 131.6 (CH), 131.5 (d, J_{CP} = 2.4 Hz, CH), 131.3 (C_q), 131.14 (d, J_{CP} = 2.8 Hz, CH), 130.9 (d, J_{CP} = 2.6 Hz, CH), 130.5 (C_q), 128.0 (CH), 127.9 (CH), 127.8 (CH), 121.2 (CH), 120.8 (CH), 117.0 (CH), 37.1 (d, J_{CP} = 4.1 Hz, CH_2), 35.0 (CH_2) ppm;

^{31}P NMR (162 MHz, $CDCl_3$) δ 26.15 ppm;

$[\alpha]^{D}_{20}$ = +121.6 (c = 0.50, $CHCl_3$).

R_t (AD-3 column, *n*-hexane/*i*-PrOH 80/20, 1.0 mL/min, 273.0 nm): tr(major) = 9.8 min, tr(minor) = 16.7 min, 87% ee.

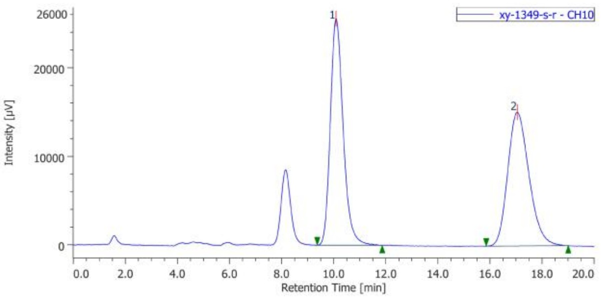

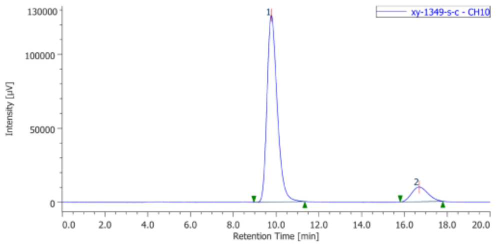

#	Peak Name	CH	tR [min]	Area [μV·sec]	Height [μV]	Area%
1	Unknown	10	10.087	875529	25568	50.569
2	Unknown	10	17.047	855815	15089	49.431

#	Peak Name	CH	tR [min]	Area [μV·sec]	Height [μV]	Area%
1	Unknown	10	9.773	4175644	126295	88.774
2	Unknown	10	16.690	528029	9949	11.226

($1^{6a}R$,1^7S,$1^{12}S$,$1^{12a}S$)-4^2-(diphenylphosphoryl)-1^6-(quinolin-8-yl)-1^5,1^6,1^{6a},1^7,1^{12},1^{12a}-hexahydro-1(1,4)-7,12-epoxybenzo[b]phenanthridina-4(1,4)-benzenacyclohexaphan-1^5-one (28)

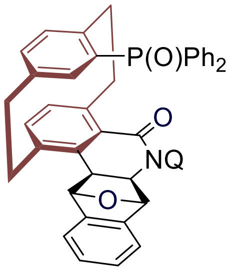

Prepared according to general procedure **3** for 48h on a 0.15 mmol scale, column chromatography (ethyl acetate/*n*-hexane = 1:1 to 3:1) afforded the compound **28** as a white solid (52 mg, 0.072 mmol, 48%), with 25:1 dr, 99% ee and chiral starting material **(*S*$_p$)-1g** as a white solid (42 mg, 0.074mmol, 49%), with 99% ee.

^{1}H NMR (400 MHz, $CDCl_3$) δ 9.05 (d, *J* = 7.0 Hz, 1H), 8.66 (dd, *J* = 4.2, 1.8 Hz, 1H), 8.19 (dd, *J* = 8.3, 1.7 Hz, 1H), 8.04 – 7.89 (m, 2H), 7.87 – 7.76 (m, 2H), 7.69 (ddt, *J* = 11.3, 6.9, 1.5 Hz, 2H), 7.47 – 7.22 (m, 9H), 7.15 (td, *J* = 7.4, 1.1 Hz, 1H), 7.04 (td, *J* = 7.5, 1.1 Hz, 1H), 6.99 – 6.86 (m, 3H), 6.79 – 6.53 (m, 3H), 5.78 (s, 1H), 5.57 (s, 1H), 4.86 (ddd, *J* = 12.9, 9.4, 3.4 Hz, 1H), 4.72 (d, *J* = 8.5 Hz, 1H), 3.65 – 3.17 (m, 6H), 2.89 (ddd, *J* = 12.9, 9.6, 5.7 Hz, 1H), 2.74 (ddd, *J* = 13.2, 9.5, 3.5 Hz, 1H) ppm;

^{13}C NMR (101 MHz, $CDCl_3$) δ 162.9 (C_q), 149.9 (CH), 147.6 (d, J_{CP} = 7.8 Hz, C_q), 147.6 (C_q), 144.9 (C_q), 144.1 (C_q), 143.0 (C_q), 138.8 (C_q), 137.6 (C_q), 136.9 (C_q), 136.7 (C_q), 136.7 (d, J_{CP} = 3.8 Hz, CH), 136.6 (C_q), 136.5 (C_q), 136.4 (CH), 136.4 (CH), 135.6 (CH), 135.1 (CH), 135.0 (CH), 134.6 (CH), 134.4 (CH), 133.7 (C_q), 133.1 (C_q), 133.0 (C_q),132.8 (CH), 132.7 (CH), 132.6 (C_q), 132.1 (C_q), 131.7 (CH), 131.6 (CH), 130.7 (d, J_{CP} = 2.8 Hz, CH), 130.6 (d, J_{CP} = 2.9 Hz, CH), 129.8 (C_q), 127.9 (CH), 127.7 (CH), 127.6 (CH), 127.5 (CH), 127.5 (d, J_{CP} = 2.8 Hz, CH), 127.1 (CH), 126.8 (CH), 126.6 (C_q), 120.9 (CH), 120.7 (CH), 118.1 (CH), 83.4 (CH), 82.3 (CH), 61.9 (CH), 41.5 (CH), 36.3 (CH_2), 35.4 (d, J_{CP} = 4.8 Hz, CH_2), 35.1 (CH_2), 32.4 (CH_2) ppm;

^{31}P NMR (162 MHz, $CDCl_3$) δ 22.06 ppm.

HRMS (ESI): *m/z* $[M+H]^+$ calcd for $C_{48}H_{37}N_2O_3P$: 721.2615; found: 721.2618.

$[\alpha]^D_{20}$ = –23.6 (c = 0.50, $CHCl_3$).

R_t (OD-3 column, *n*-hexane/*i*-PrOH 80/20, 1.0 mL/min, 273.0 nm): tr(major) = 27.4 min, tr(minor) = 37.3 min, 99% ee.

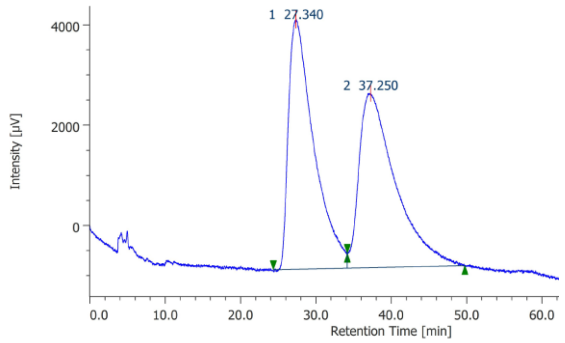

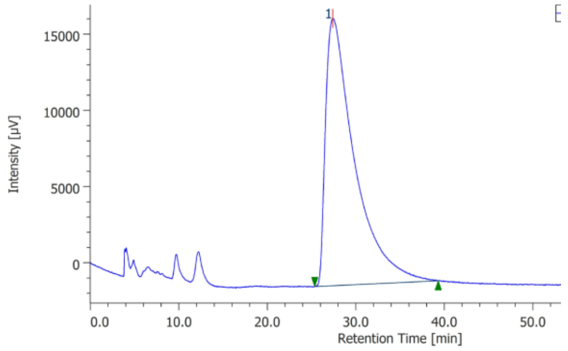

#	Peak Name	CH	tR [min]	Area [µV·sec]	Height [µV]	Area%
1	Unknown	10	27.340	1161500	4983	49.881
2	Unknown	10	37.250	1167049	3486	50.119

#	Peak Name	CH	tR [min]	Area [µV·sec]	Height [µV]	Area%
1	Unknown	10	27.373	3991696	17523	100.000

(S_p)-1g:

R$_t$ (OD-3 column, *n*-hexane/*i*-PrOH 80/20, 1.0 mL/min, 273.0 nm): tr(major) = 27.4 min, tr(minor) = 37.3 min, 99% ee.

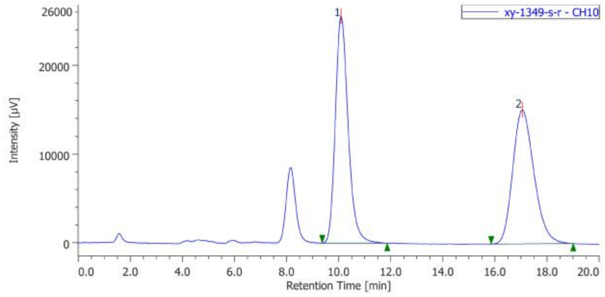

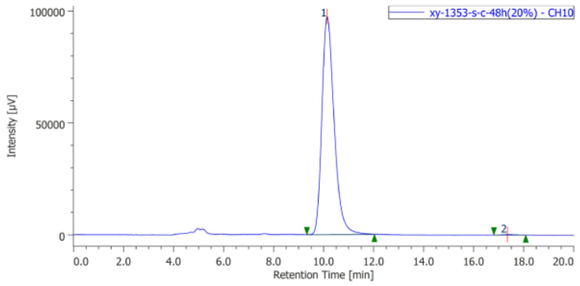

#	Peak Name	CH	tR [min]	Area [µV·sec]	Height [µV]	Area%
1	Unknown	10	10.087	875529	25568	50.569
2	Unknown	10	17.047	855815	15089	49.431

#	Peak Name	CH	tR [min]	Area [µV·sec]	Height [µV]	Area%
1	Unknown	10	10.137	3315742	97410	99.801
2	Unknown	10	17.340	6626	188	0.199

5 Transformations

Synthesis of (S_p)-29:

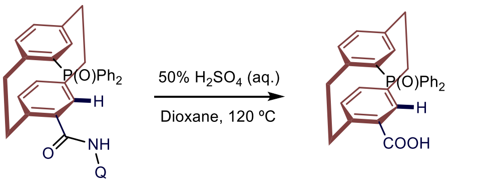

(S_p)-**1g**
99% ee

(S_p)-**29**
68% yield; 98% ee

To an oven-dried 25 mL Schlenk tube were added (S_p)-**1g** (90 mg, 0.2 mmol), dioxane (1.0 mL), 50% H_2SO_4 (3 mL) under a nitrogen atmosphere. The reaction mixture was stirred at 120 °C (oil bath temperature) for 24 h. After cooling to room temperature, the reaction mixture was diluted with ethyl acetate (10 mL), and the aqueous phase was extracted with ethyl acetate (3 × 10 mL). The combined organic extracts were dried over anhydrous Na_2SO_4, filtered, and concentrated under reduced pressure, and the residue was purified by flash column chromatography on silica gel using DCM/MeOH (20:1, v/v) as the eluent to give (S_p)-**29** as a white solid (22 mg, 93% yield, 98% ee).

^{1}H NMR (400 MHz, $CDCl_3$) δ 9.77 (s, 1H), 7.88 (dd, J = 11.9, 7.4 Hz, 2H), 7.61 – 7.09 (m, 9H), 6.89 – 6.60 (m, 4H), 6.36 – 6.09 (m, 1H), 4.76 (ddd, J = 13.7, 10.2, 4.1 Hz, 1H), 3.76 (ddd, J = 13.4, 9.9, 3.3 Hz, 1H), 3.31 – 2.72 (m, 6H) ppm;

^{13}C NMR (101 MHz, $CDCl_3$) δ 168.6 (C_q), 146.5 (d, J_{CP} = 7.3 Hz, C_q), 142.4 (C_q), 139.32 (d, J_{CP} = 13.4 Hz, C_q) 138.6 (C_q), 136.5 (d, J_{CP} = 2.9 Hz, CH), 136.2 (CH), 136.0 (d, J_{CP} = 3.9 Hz, CH), 135.9 (CH), 135.6 (CH), 135.5 (CH), 135.1 (C_q), 134.2 (CH), 134.1 (C_q), 133.1 (d, J_{CP} = 9.8 Hz, CH), 131.7 (d, J_{CP} = 9.7 Hz, CH), 131.5 (d, J_{CP} = 2.8 Hz, CH), 131.3 (d, J_{CP} = 2.6 Hz, CH), 130.4 (d, J_{CP} = 20.3 Hz, C_q), 129.2 (C_q), 128.2 (d, J_{CP} = 7.3 Hz, CH), 128.1 (d, J_{CP} = 7.2 Hz, CH), 35.6 (d, J_{CP} = 4.4 Hz, CH_2), 35.3 (CH_2), 34.8 (CH_2), 34.7 (CH_2) ppm;

^{31}P NMR (162 MHz, CDC13) δ 28.64 ppm.

HRMS (ESI): m/z $[M+H]^+$ calcd for $C_{29}H_{25}O_3P$: 453.1614; found: 453.1629.

R_t (AD-3 column, n-hexane/i-PrOH 80/20, 1.0 mL/min, 273.0 nm): tr(major) = 33.8 min, tr(minor) = 8.2 min, 98% ee.

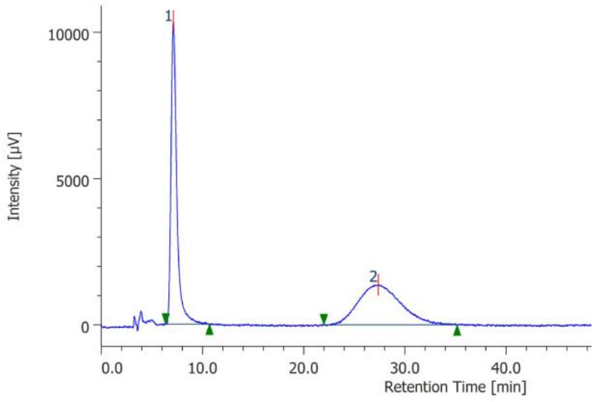

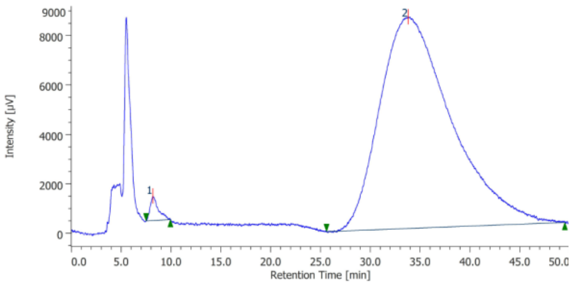

#	Peak Name	CH	tR [min]	Area [μV·sec]	Height [μV]	Area%
1	Unknown	10	7.110	413131	10349	50.708
2	Unknown	10	27.363	401596	1376	49.292

#	Peak Name	CH	tR [min]	Area [μV·sec]	Height [μV]	Area%
1	Unknown	9	8.187	55477	994	1.277
2	Unknown	9	33.800	4289171	8569	98.723

Synthesis of (S_p)-30:

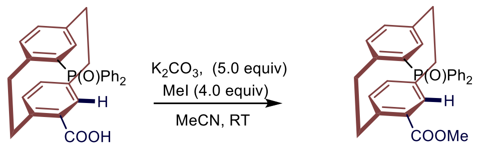

(S_p)-**29**
98% ee

(S_p)-**30**
93% yield; 99% ee

To an oven-dried 10 mL flask were added (S_p)-**29** (23 mg, 0.05 mmol), dry dichloromethane (1.0 mL), K_2CO_3 (34 mg, 0.25 mmol) and MeI (12 μL, 0.2 mmol) under a nitrogen atmosphere. The reaction mixture was stirred at room temperature for 24 h. After completion, the solvent was removed under reduced pressure, and the residue was purified by flash column chromatography on silica gel using DCM/MeOH (20:1, v/v) as the eluent to give (S_p)-**30** as a white solid (22 mg, 93% yield, 99% ee).

^{1}H NMR (400 MHz, $CDCl_3$) δ 7.64 (ddt, J = 11.7, 6.8, 1.5 Hz, 2H), 7.58 – 7.28 (m, 8H), 7.18 (d, J = 1.5 Hz, 1H), 6.81 – 6.59 (m, 4H), 6.21 (dd, J = 14.8, 1.8 Hz, 1H), 4.41 (ddd, J = 12.9, 10.2, 4.3 Hz, 1H), 4.12 (s, 3H), 3.76 – 3.57 (m, 1H), 3.27 – 2.92 (m, 4H), 2.92 – 2.75 (m, 2H) ppm;

^{13}C NMR (101 MHz, CDCl3) δ 167.1 (C_q), 146.1 (d, J_{CP} = 7.6 Hz, C_q), 142.3 (C_q), 139.2 (d, J_{CP} = 13.1 Hz, C_q), 138.9 (C_q), 136.2 (d, J_{CP} = 3.1 Hz, CH), 135.9 (CH), 135.8 (d, J_{CP} = 2.4 Hz, CH), 135.7 (C_q), 135.5 (d, J_{CP} = 103.9 Hz, C_q), 133.8 (CH), 132.8 (C_q), 132.7 (CH), 132.6 (CH), 132.0 (C_q), 131.6 (CH), 131.6 (d, J_{CP} = 36.2 Hz, C_q), 131.5 (CH), 131.3 (d, J_{CP} = 2.9 Hz, CH), 131.1 (d, J_{CP} = 2.7 Hz, CH), 130.3 (C_q), 128.2 (CH), 128.0 (CH), 127.9 (CH), 52.2 (CH_3), 35.4 (d, J_{CP} = 4.3 Hz, CH_2), 35.3 (CH_2), 34.7 (CH_2), 34.7 (CH_2) ppm;

^{31}P NMR (162 MHz, CDCl3) δ 27.16 ppm.

HRMS (ESI): *m/z* $[M+H]^+$ calcd for : $C_{30}H_{27}O_3P$: 467.1771; found: 467.1787.

$[\alpha]^D{}_{20}$ = +14.5 (c = 0.20, $CHCl_3$).

R_t (AD-3 column, *n*-hexane/*i*-PrOH 60/40, 1.0 mL/min, 250.0 nm): tr(major) = 11.0 min, tr(minor) = 8.7 min, 99% ee.

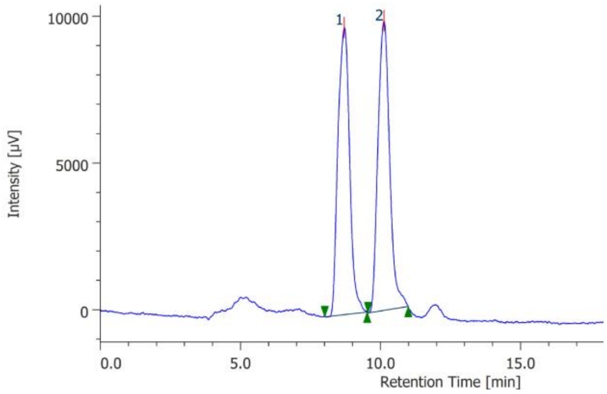

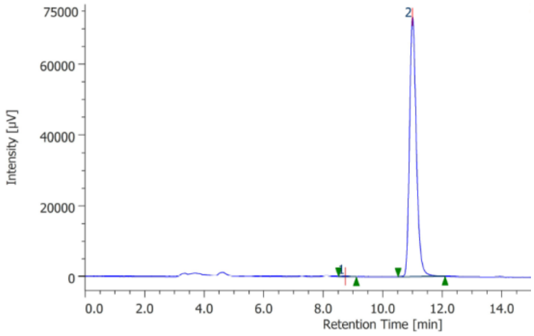

#	Peak Name	CH	tR [min]	Area [µV·sec]	Height [µV]	Area%
1	Unknown	10	8.703	281379	9776	49.675
2	Unknown	10	10.120	285066	9857	50.325

#	Peak Name	CH	tR [min]	Area [µV·sec]	Height [µV]	Area%
1	Unknown	10	8.740	2054	143	0.175
2	Unknown	10	10.997	1168456	73430	99.825

Synthesis of (*S*$_p$)-31:

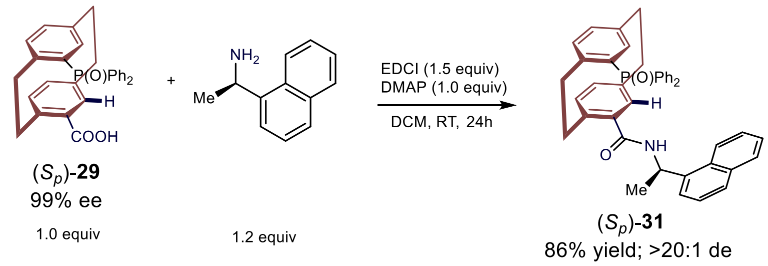

To an oven-dried 10 mL flask were added (*S*$_p$)-**29** (23 mg, 0.05 mmol), *R*-1-(2-Naphthyl)ethylamine (10 mg, 0.06 mmol), EDCI (14.3 mg, 0.073 mmol), DMAP (6 mg, 0.05 mmol), and dry dichloromethane (1.0 mL) under a nitrogen atmosphere. The reaction mixture was stirred at room temperature for 24 h. After completion, the solvent was removed under reduced pressure, and the residue was purified by flash column chromatography on silica gel using hexane/ethyl acetate (1:1, v/v) as the eluent to give (*S*$_p$)-**31** as a white solid (26 mg, 86% yield, >20:1 de).

^{1}H NMR (400 MHz, CDCl3) δ 8.12 (s, 1H), 8.09 – 7.96 (m, 2H), 7.92 – 7.79 (m, 3H), 7.57 – 7.29 (m, 10H), 7.18 (td, *J* = 7.7, 2.9 Hz, 2H), 6.83 (dt, *J* = 6.3, 1.9 Hz, 2H), 6.75 (dd, *J* = 7.6,

4.1 Hz, 1H), 6.57 (d, J = 1.2 Hz, 2H), 6.27 (d, J = 1.8 Hz, 1H), 5.79 – 5.61 (m, 1H), 3.95 (ddd, J = 13.0, 9.9, 5.2 Hz, 1H), 3.56 (t, J = 11.4 Hz, 1H), 3.15 – 2.93 (m, 4H), 2.91 – 2.74 (m, 2H), 1.85 (d, J = 6.9 Hz, 3H) ppm;

^{13}C NMR (101 MHz, $CDCl_3$) δ 167.3 (C_q), 145.4 (d, J_{CP} = 7.4 Hz, C_q), 141.4 (C_q), 139.5 (d, J_{CP} = 13.1 Hz, C_q), 139.1 (C_q), 137.0 (C_q), 136.6 (C_q), 136.3 (d, J_{CP} = 3.2 Hz, CH), 136.0 (CH), 135.9 (d, J = 5.4 Hz, CH), 135.8 (CH), 135.5 (C_q), 135.3 (CH), 134.8 (CH), 134.4 (C_q), 133.4 (C_q), 132.7 (C_q), 132.5 (d, J_{CP} = 9.7 Hz, CH), 132.4 (CH), 131.5 (d, J_{CP} = 9.2 Hz, C_q), 131.4 (CH), 131.3 (d, J_{CP} = 2.9 Hz, CH), 131.3 (d, J_{CP} = 2.8 Hz, CH), 131.1 (C_q), 130.6 (CH), 130.1 (C_q), 128.2 (d, J_{CP} = 1.9 Hz), 128.1 (CH), 128.0 (d, J_{CP} = 2.3 Hz, CH), 127.5 (CH), 126.8 (CH), 125.7 (CH), 125.5 (d, J_{CP} = 3.9 Hz, CH), 49.3 (CH), 36.2 (d, J_{CP} = 3.9 Hz, CH_2), 334.8 (CH_2), 34.7 (CH_2), 34.1 (CH_2), 20.7 (CH_3) ppm;

^{31}P NMR (162 MHz, CDC13) δ 27.38 ppm.

HRMS (ESI): *m/z* $[M+H]^+$ calcd for : $C_{41}H_{36}NO_2P$: 606.2556; found: 606.2579.

$[\alpha]^D{}_{20}$ = +61.0 (c = 0.20, $CHCl_3$).

Synthesis of (S_p)-32:

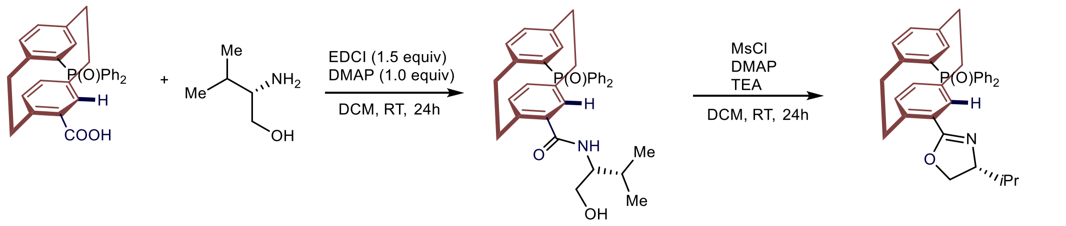

(S_p)-**29**
99% ee
1.0 equiv

***L*-valinol**
1.2 equiv

(S_p)-**33**

(S_p)-**32**
66% yield; >20:1 de

To an oven-dried 10 mL flask were added (S_p)-**29** (45 mg, 0.1 mmol), *L*-valinol (12 mg, 0.12 mmol), EDCI (28.6 mg, 0.15 mmol), DMAP (12 mg, 0.1 mmol), and dry dichloromethane (2.0 mL) under a nitrogen atmosphere. The reaction mixture was stirred at room temperature for 24 h. After completion, the crude product was purified by flash column chromatography on silica gel to afford (S_p)-**33** as a yellowish solid, which was used directly in the next step without further purification.

To an oven-dried 10 mL flask were added (S_p)-**33**, triethylamine (70 μL, 0.5 mmol), DMAP (2.4 mg, 20 mol%), and dry dichloromethane (3.0 mL) under a nitrogen atmosphere. Methanesulfonyl chloride (19 μL, 0.2 mmol) was then added dropwise to the stirred solution at

room temperature. The reaction was stirred for 24 h, then quenched by the addition of methanol (5 mL) and stirred for an additional 2 h. The solvent was removed under reduced pressure, and the residue was purified by flash column chromatography on silica gel using hexane/ethyl acetate (1:1, v/v) as the eluent to give (S_p)-**32** as a yellow solid (34 mg, 66% overall yield, >20:1 de).

^{1}H NMR (400 MHz, $CDCl_3$) δ 7.76 – 7.63 (m, 2H), 7.54 – 7.35 (m, 5H), 7.35 – 7.15 (m, 4H), 6.84 – 6.67 (m, 1H), 6.67 – 6.49 (m, 3H), 6.27 (dd, J = 14.9, 1.8 Hz, 1H), 4.83 (dd, J = 9.9, 7.7 Hz, 1H), 4.53 – 4.28 (m, 2H), 4.19 (t, J = 7.7 Hz, 1H), 3.89 – 3.70 (m, 1H), 3.21 – 2.94 (m, 4H), 2.91 – 2.62 (m, 2H), 1.98 (q, J = 6.7 Hz, 1H), 1.10 (d, J = 6.7 Hz, 3H), 0.92 (d, J = 6.7 Hz, 3H) ppm;

^{13}C NMR (101 MHz, $CDCl_3$) δ 164.0 (C_q), 146.4 (d, J_{CP} = 7.5 Hz, C_q), 140.8 (C_q), 138.79 (d, J_{CP} = 1.6 Hz, C_q), 138.7 (C_q), 136.2 (d, J_{CP} = 3.2 Hz, CH), 135.9 (C_q), 135.8 (d, J_{CP} = 6.2 Hz, CH), 135.6 (d, J_{CP} = 5.0 Hz, CH), 135.5 (CH), 134.9 (CH), 134.4 (CH), 133.2 (CH), 132.9 (CH), 132.7 (d, J_{CP} = 9.6 Hz, CH), 132.1 (C_q), 131.7 (d, J_{CP} = 9.1 Hz, CH), 131.4, 131.2 (d, J_{CP} = 2.7 Hz, CH), 131.0 (d, J_{CP} = 2.8 Hz, CH), 130.4 (C_q), 129.1 (C_q), 128.1 (d, J_{CP} = 12.0 Hz, CH), 127.9 (d, J_{CP} = 11.8 Hz, CH), 71.8 (CH), 70.3 (CH_2), 35.7 (CH_2), 35.0 (d, J_{CP} = 4.2 Hz, CH_2), 34.7 (CH_2), 34.6 (CH_2), 32.8 (CH), 19.5 (CH_3), 18.2 (CH_3) ppm;

^{31}P NMR (162 MHz, $CDCl_3$) δ 27.95 ppm.

HRMS (ESI): *m/z* $[M+H]^+$ calcd for : $C_{34}H_{34}NO_2P$: 520.2400; found: 520.2423.

$[\alpha]^D{}_{20}$ = –40.4 (c = 0.25, $CHCl_3$).

6 Mechanistic Studies

H/D Exchange Experiment with D_2O

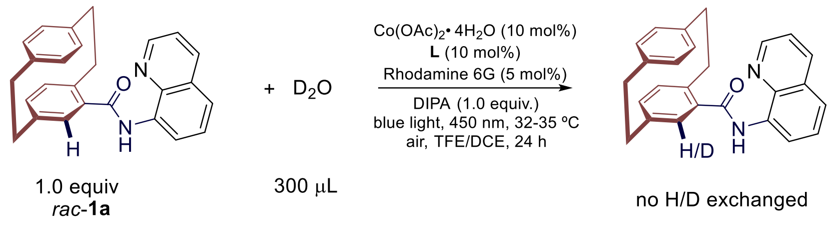

A 10 mL vial was charged with racemic amide *rac*-**1a** (0.15 mmol, 1.0 equiv.), $Co(OAc)_2{\cdot}4H_2O$ (3.7 mg, 10 mol%), ligand L (5.3 mg, 10 mol%), Rhodamine 6G (3.6 mg, 5 mol%), DIPA (21 μL, 0.15 mmol, 1.0 equiv.), and a Teflon-coated magnetic stirring bar. Then, DCE (0.25 mL), TFE (1.0 mL), and D_2O (300 μL) were added. No alkene or allene coupling partner was added in this experiment. The vial was stirred at room temperature under blue LEDs (450 nm) for 24 hours. After completion, the reaction mixture was concentrated under vacuum, and the residue was analyzed by ^1H NMR.

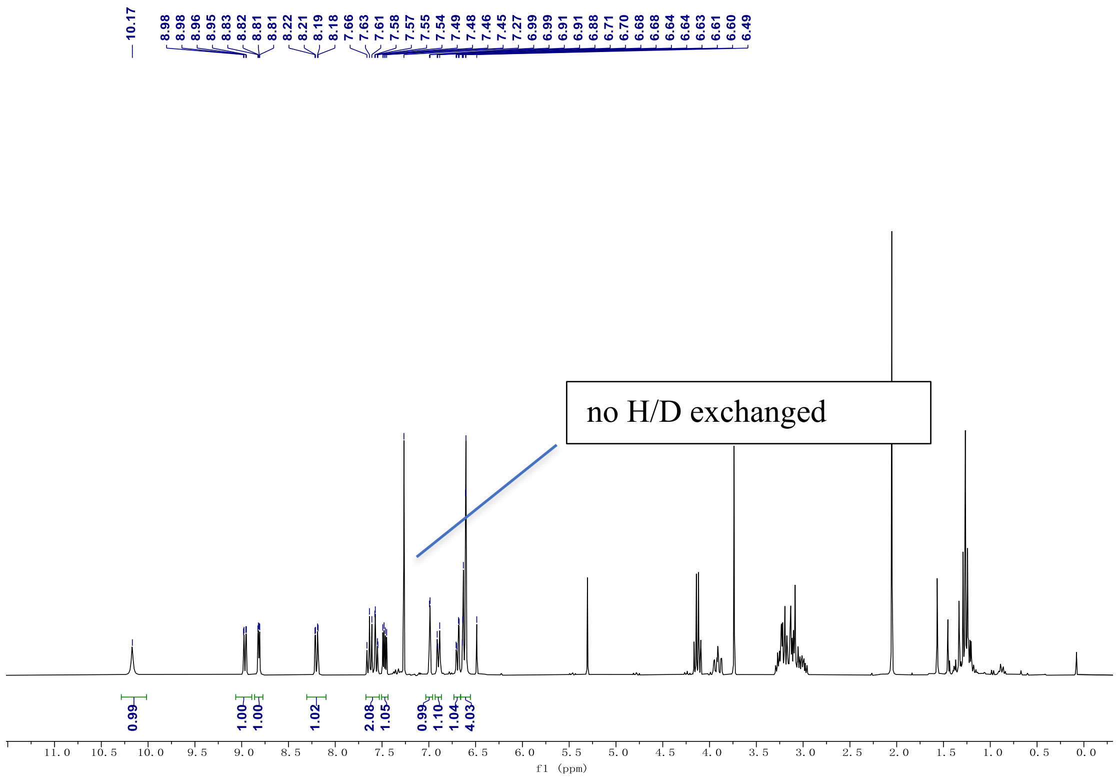

H/D Exchange Experiment with CD_3OD

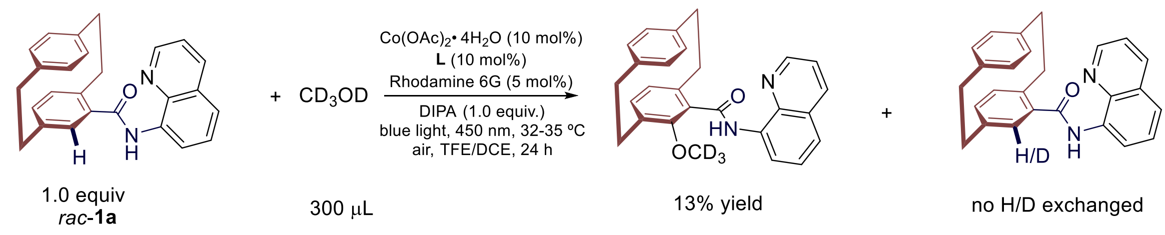

A 10 mL vial was charged with racemic amide *rac*-**1a** (0.15 mmol, 1.0 equiv.), $Co(OAc)_2{\cdot}4H_2O$ (3.7 mg, 10 mol%), ligand L (5.3 mg, 10 mol%), Rhodamine 6G (3.6 mg, 5 mol%), DIPA (21 µL, 0.15 mmol, 1.0 equiv.), and a Teflon-coated magnetic stirring bar. Then, DCE (0.25 mL), TFE (1.0 mL), and CD_3OD (300 µL) were added. No alkene or allene coupling partner was added in this experiment. The vial was stirred at room temperature under blue LEDs (450 nm) for 24 hours. After completion, the reaction mixture was concentrated under vacuum, and the residue was analyzed by ^1H NMR.

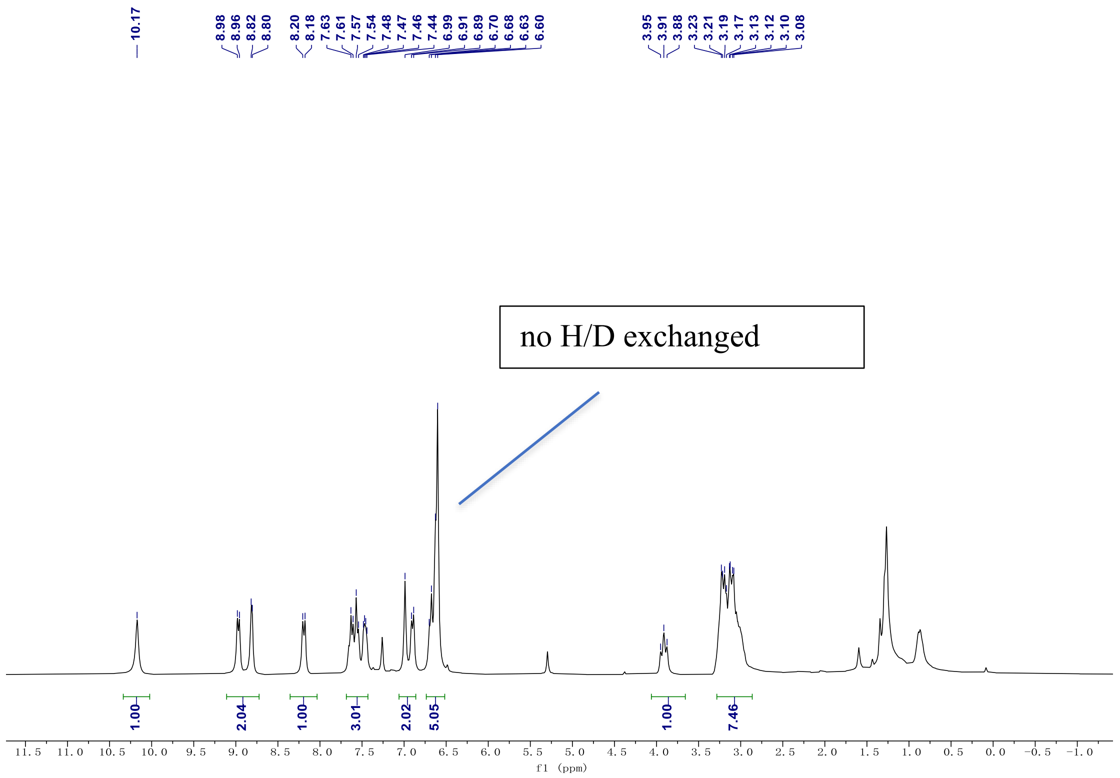

Experimental results indicate that the C–H activation is not reversible under our reaction conditions.

7 Computational Studies

Computational details

All DFT calculations were performed with the Gaussian 16, Revision A.03 package[12]. Geometry optimization of all stationary points was carried out at the TPSS[13] level of theory in combination with Grimme's D3 dispersion corrections with a Becke-Johnson damping scheme D3(BJ)[14] All atoms were described with a def2-SVP basis set[15]. Analytical frequency calculations were carried out at the same level of theory in order to identify intermediates (no imaginary frequencies) and transition states (single imaginary frequency) and to provide thermal and non-thermal corrections to the free energy in gas-phase at 308.15 K and 1 atm. Intrinsic reaction coordinate (IRC) calculations[16] were also performed from the transition state structures to ensure their connectivity with corresponding local minima. The electronic energy was then refined through PW6B95[17] single-point calculations on the optimized geometries with a def2-TZVPP basis set[15] in combination with a standalone version of Grimme's D4 dispersion correction[18]. Solvent effects were accounted for the single point calculations through the SMD model[19] with a dielectric constant of ε = 26.726, which corresponds to 2,2,2-TriFluoroEthanol (TFE).

The optimized structures were visualized using PyMOL software.[24]

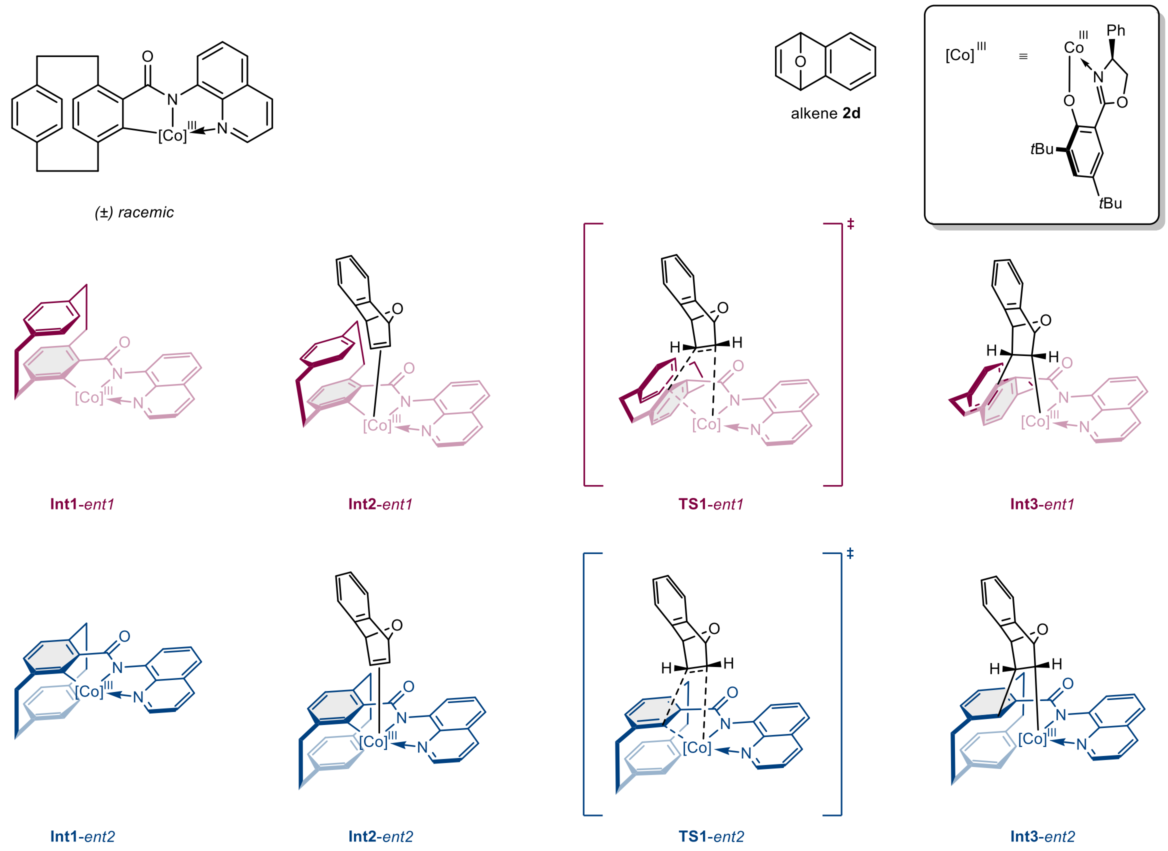

Figure S2. Illustrated structures considered for computing energies of intermediates and transition states for alkene migratory insertion with both enantiomers of racemic paracyclophanecarboxamides.

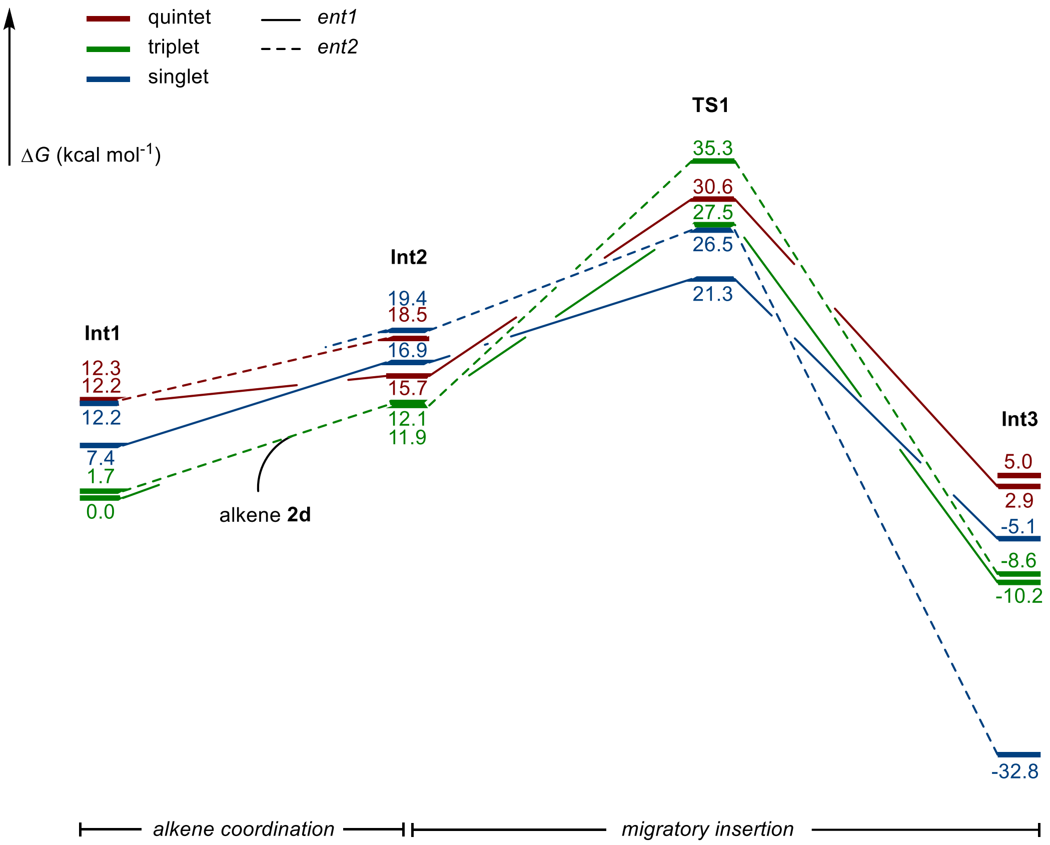

Figure S3. Computed Gibbs free energies ($\Delta G_{308.15}$) in kcal·mol^{-1} for the elementary step *via* both substrate enantiomers (*ent1*/*ent2*) at the PW6B95-D4/def2-TZVPP+SMD(TFE)//TPSS-D3(BJ)/def2-SVP level of theory.

Table S3. Calculated electronic and Gibbs free energies at the PW6B95-D4/def2-TZVPP+SMD(TFE)//TPSS-D3(BJ)/def2-SVP level of theory for all the reported structures (in Hartree).

Enantiomer	Structures[a]	Electronic Energy	Total Gibbs Free Energy
	Alkene **2d**[1]	-461.825703	-461.718134
ent1	**Int1**[1]	-3673.342868	-3672.627351
	**Int1**[3]	-3673.352106	-3672.639165
	**Int1**[5]	-3673.328236	-3672.619702
	**Int2**[1]	-4135.178894	-4134.330316
	**Int2**[3]	-4135.182681	-4134.337971
	**Int2**[5]	-4135.172584	-4134.332317
	**TS1**[1]	-4135.176069	-4134.323375
	**TS1**[3]	-4135.160109	-4134.313461
	**TS1**[5]	-4135.150696	-4134.308567
	**Int3**[1]	-4135.219523	-4134.365417
	**Int3**[3]	-4135.223853	-4134.373558
	**Int3**[5]	-4135.200034	-4134.352615
ent2	**Int1**[1]	-3673.336178	-3672.619670
	**Int1**[3]	-3673.349404	-3672.636391
	**Int1**[5]	-3673.328032	-3672.619534
	**Int2**[1]	-4135.177118	-4134.326416
	**Int2**[3]	-4135.181793	-4134.338272
	**Int2**[5]	-4135.167154	-4134.327866

	**TS1**^{1}	-4135.166563	-4134.315101
	**TS1**^{3}	-4135.146052	-4134.301117
	**Int3**^{1}	-4135.259995	-4134.409648
	**Int3**^{3}	-4135.218655	-4134.370984
	**Int3**^{5}	-4135.191777	-4134.349377

[a]Superscripted 1, 3 and 5 represents singlet, triplet, and quintet spin states, respectively.

Distortion-interaction analysis

Distortion-interaction analyses were performed on the alkene insertion transition states (**TS1**) by dissecting the optimized TS geometry into two parts: the catalyst complex (**Int1**) and the alkene **2d** substrate. Single-point energies of respective fragments were calculated in their distorted states separately and compared to their relaxed forms to obtain distortion energies. Interaction energies were computed as the difference between electronic activation energies and the sum of the distorted fragment energies.

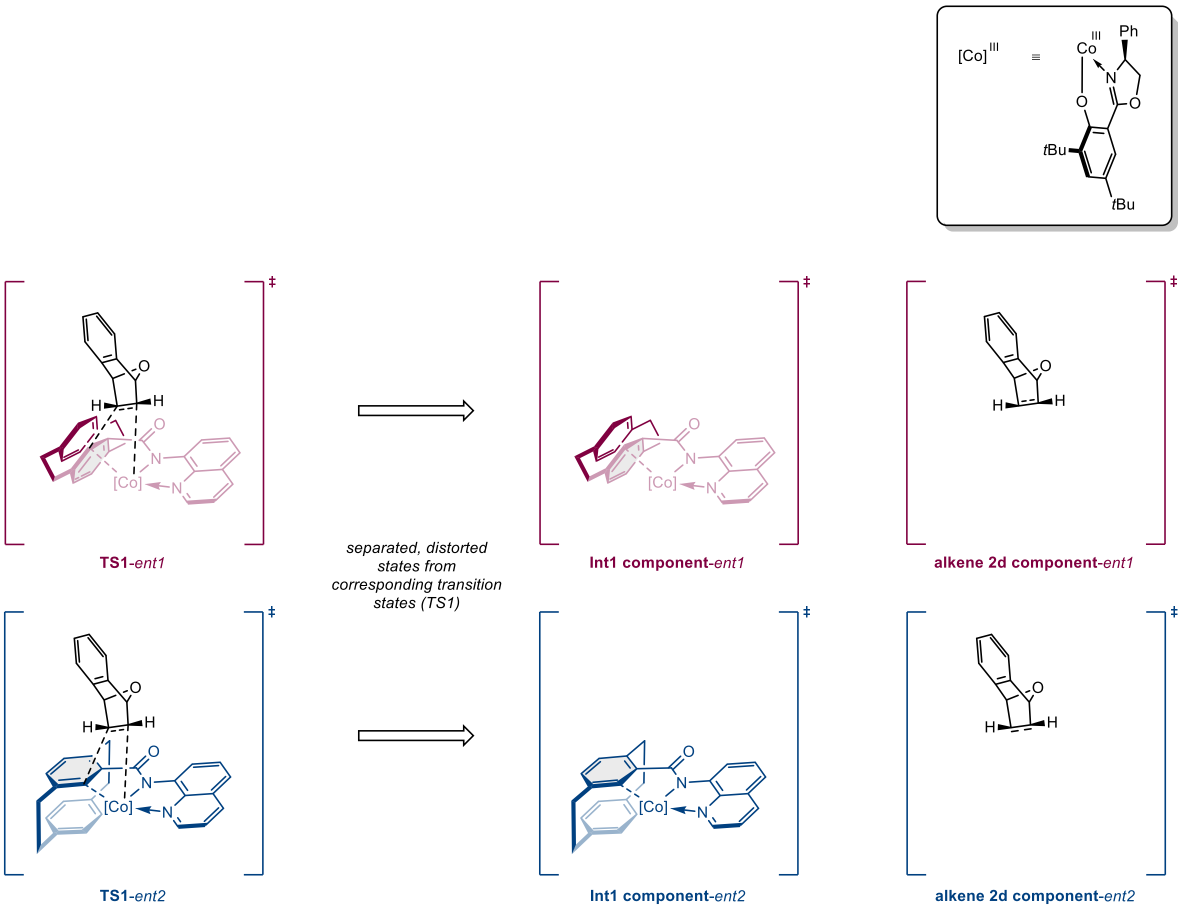

Figure S4. Dissection of the **TS1** geometries into their corresponding distorted **Int1** and alkene **2d** components, as extracted directly from the optimized transition states for use in distortion-interaction energy analysis.

Table S4. Calculated electronic energies at the PW6B95-D3(BJ)/def2-TZVPP+SMD(TFE)//TPSS-D3(BJ)/def2-SVP level of theory for all the reported structures (in Hartree).

Enantiomer	Structures[a]	Electronic Energy
ent1	alkene **2d** component[1]	-3673.308955
	**Int1** component[1]	-461.788380
ent2	alkene **2d** component[1]	-3673.287161
	**Int1** component[1]	-461.776622

[a]Superscripted 1 represents singlet spin state.

NICS calculations

Due to the limited support from selected level of theory for the nucleus independent Chemical Shift (NICS) studies[20], additional energy refinements were compared with the DFT long-range-corrected hybrid UCAM-B3LYP (unrestricted Coulomb-Attenuating Method B3LYP)[21] and hybrid PBE0[22] functionals, in combination with 6-311+G(d,p) and 6-311++G(d,p) basis set, respectively (**Table S4**). Later, the considered one was used for Gauge-Independent Atomic Orbital (GIAO) method[22] with ghost (Bq) atoms.

All the single-point energy refinements for comparison were performed on same set of geometries obtained at the TPSS-D3(BJ)/def2-SVP level of theory, which includes dispersion corrections during geometry optimization. Since dispersion corrections are not supported in NICS calculations with ghost atoms using the GIAO method in Gaussian, we conducted a comparative analysis to identify the most appropriate dispersion-free level of theory that still reflects the qualitative thermodynamic behavior of the system. As anticipated, the removal of dispersion corrections led to an increase in energy barriers and changes in intermediate stabilities. However, only PBE0 preserved the exergonic nature of the migratory insertion step ($\Delta G^{\mathrm{Int3}}{}_{ent1}$ = -2.0 kcal·mol^{-1} and $\Delta G^{\mathrm{Int3}}{}_{ent1}$ = -14.8 kcal·mol^{-1}), in line with the dispersion-corrected reference profile. In contrast, UCAM-B3LYP predicted the ent1 insertion step to be endergonic ($\Delta G^{\mathrm{Int3}}{}_{ent1}$ = 1.6 kcal·mol^{-1} and $\Delta G^{\mathrm{Int3}}{}_{ent1}$ = -14.4 kcal·mol^{-1}), altering the reaction thermodynamics.

Based on this evaluation, PBE0/6-311++G(d,p) was selected as the level of theory for NICS (GIAO) calculations, as it offers the best qualitative agreement with the full DFT profile while remaining compatible with ghost atom computations.

Table S5. Computed relative Gibbs free energies ($\Delta G_{308.15}$) in kcal·mol^{-1} at selected level of theories for the comparison. The energy refinements were performed on the optimized geometries obtained from TPSS-D3(BJ)/def2-SVP level of theory.

Selected level of theory	$\Delta\Delta G^{\ddagger}{}_{(ent2\text{ - }ent1)}$	$\Delta G^{\mathrm{Int3}}{}_{ent1}$	$\Delta G^{\mathrm{Int3}}{}_{ent2}$
PW6B95-D4/def2-TZVPP+SMD(TFE)	5.2	-10.2	-32.8
PW6B95/def2-TZVPP+SMD(TFE)	3.8	1.1	-18.7
UCAM-B3LYP/6-311+G(d,p)+SMD(TFE)	1.1	1.6	-14.4
PBE0-D4/6-311++G(d,p)+SMD(TFE)	3.3	-18.3	-34.1
PBE0/6-311++G(d,p)+SMD(TFE)	1.1	-2.0	-14.8

Table S6. Calculated electronic and Gibbs free energies at the PW6B95/def2-TZVPP+SMD(TFE)//TPSS-D3(BJ)/def2-SVP level of theory without dispersion corrections for all the reported structures (in Hartree).

Enantiomer	Structures[a]	Electronic Energy	Total Gibbs Free Energy
	Alkene **2d**[1]	-461.825703	-461.697834

ent1	**Int1**^{1}	-3673.342868	-3672.466799
	**Int1**^{3}	-3673.352106	-3672.480779
	**Int1**^{5}	-3673.328236	-3672.464140
	**Int2**^{1}	-4135.178894	-4134.132714
	**Int2**^{3}	-4135.182681	-4134.141359
	**Int2**^{5}	-4135.172584	-4134.137605
	**TS1**^{1}	-4135.176069	-4134.121383
	**TS1**^{3}	-4135.160109	-4134.114919
	**TS1**^{5}	-4135.150696	-4134.111831
	**Int3**^{1}	-4135.219523	-4134.166829
	**Int3**^{3}	-4135.223853	-4134.176830
	**Int3**^{5}	-4135.200034	-4134.158011
ent2	**Int1**^{1}	-3673.336178	-3672.458406
	**Int1**^{3}	-3673.349404	-3672.478454
	**Int1**^{5}	-3673.328032	-3672.463855
	**Int2**^{1}	-4135.177118	-4134.126750
	**Int2**^{3}	-4135.181793	-4134.143222
	**Int2**^{5}	-4135.167154	-4134.135726
	**TS1**^{1}	-4135.166563	-4134.115366
	**TS1**^{3}	-4135.146052	-4134.104167
	**Int3**^{1}	-4135.259995	-4134.208459
	**Int3**^{3}	-4135.218655	-4134.171286
	**Int3**^{5}	-4135.191777	-4134.152255

[a]Superscripted 1, 3 and 5 represents singlet, triplet, and quintet spin states, respectively.

Table S7. Calculated electronic and Gibbs free energies at the UCAM-B3LYP/6-311+G(d,p)+SMD(TFE)//TPSS-D3(BJ)/def2-SVP level of theory without dispersion corrections for all the reported structures (in Hartree).

Enantiomer	Structures[a]	Electronic Energy	Total Gibbs Free Energy
	Alkene **2d**^{1}	-460.917887	-460.801474
ent1	**Int1**^{1}	-3668.033406	-3667.268932
	**Int1**^{3}	-3668.043185	-3667.282217
	**Int1**^{5}	-3668.019666	-3667.263193
	**Int2**^{1}	-4128.935648	-4128.029029
	**Int2**^{3}	-4128.934076	-4128.032328
	**Int2**^{5}	-4128.924070	-4128.027157
	**TS1**^{1}	-4128.922596	-4128.010505
	**TS1**^{3}	-4128.909099	-4128.003706
	**TS1**^{5}	-4128.901132	-4128.001482
	**Int3**^{1}	-4128.981808	-4128.070025
	**Int3**^{3}	-4128.988417	-4128.081178
	**Int3**^{5}	-4128.965513	-4128.061660
ent2	**Int1**^{1}	-3668.024488	-3667.258661
	**Int1**^{3}	-3668.040416	-3667.279164
	**Int1**^{5}	-3668.018642	-3667.262098
	**Int2**^{1}	-4128.928072	-4128.017949
	**Int2**^{3}	-4128.937595	-4128.037479
	**Int2**^{5}	-4128.925128	-4128.029149
	**TS1**^{1}	-4128.919792	-4128.008792
	**TS1**^{3}	-4128.898745	-4127.994809
	**Int3**^{1}	-4129.016671	-4128.106564
	**Int3**^{3}	-4128.980601	-4128.073656
	**Int3**^{5}	-4128.953482	-4128.052295

[a]Superscripted 1, 3 and 5 represents singlet, triplet, and quintet spin states, respectively.

Table S8. Calculated electronic and Gibbs free energies at the PBE0-D4/6-311++G(d,p)+SMD(TFE)//TPSS-D3(BJ)/def2-SVP level of theory for all the reported structures (in Hartree).

Enantiomer	Structures[a]	Electronic Energy	Total Gibbs Free Energy
	Alkene **2d**^{1}	-460.645821	-460.529913

ent1	**Int1**^{1}	-3666.479855	-3665.720732
	**Int1**^{3}	-3666.491349	-3665.735320
	**Int1**^{5}	-3666.466481	-3665.714902
	**Int2**^{1}	-4127.137356	-4126.237510
	**Int2**^{3}	-4127.140250	-4126.244725
	**Int2**^{5}	-4127.128389	-4126.237469
	**TS1**^{1}	-4127.136438	-4126.232201
	**TS1**^{3}	-4127.123392	-4126.225582
	**TS1**^{5}	-4127.112638	-4126.219721
	**Int3**^{1}	-4127.186887	-4126.281486
	**Int3**^{3}	-4127.195607	-4126.294338
	**Int3**^{5}	-4127.171681	-4126.273504
ent2	**Int1**^{1}	-3666.472222	-3665.711894
	**Int1**^{3}	-3666.488000	-3665.731842
	**Int1**^{5}	-3666.465460	-3665.713806
	**Int2**^{1}	-4127.137270	-4126.234929
	**Int2**^{3}	-4127.140990	-4126.246805
	**Int2**^{5}	-4127.125057	-4126.234786
	**TS1**^{1}	-4127.130142	-4126.226923
	**TS1**^{3}	-4127.111230	-4126.214778
	**Int3**^{1}	-4127.221994	-4126.319527
	**Int3**^{3}	-4127.191347	-4126.292294
	**Int3**^{5}	-4127.165274	-4126.275105

[a]Superscripted 1, 3 and 5 represents singlet, triplet, and quintet spin states, respectively.

Table S9. Calculated electronic and Gibbs free energies at the PBE0/6-311++G(d,p)+SMD(TFE)//TPSS-D3(BJ)/def2-SVP level of theory without dispersion corrections for all the reported structures (in Hartree).

**Enantiomer**	**Structures**[a]	**Electronic Energy**	**Total Gibbs Free Energy**
	Alkene **2d**^{1}	-460.645821	-460.509746
ent1	**Int1**^{1}	-3666.479855	-3665.539557
	**Int1**^{3}	-3666.491349	-3665.556686
	**Int1**^{5}	-3666.466481	-3665.540229
	**Int2**^{1}	-4127.137356	-4126.012220
	**Int2**^{3}	-4127.140250	-4126.020106
	**Int2**^{5}	-4127.128389	-4126.015335
	**TS1**^{1}	-4127.136438	-4126.000858
	**TS1**^{3}	-4127.123392	-4125.998779
	**TS1**^{5}	-4127.112638	-4125.995010
	**Int3**^{1}	-4127.186887	-4126.054560
	**Int3**^{3}	-4127.195607	-4126.069663
	**Int3**^{5}	-4127.171681	-4126.051634
ent2	**Int1**^{1}	-3666.472222	-3665.529713
	**Int1**^{3}	-3666.488000	-3665.553933
	**Int1**^{5}	-3666.465460	-3665.539020
	**Int2**^{1}	-4127.137270	-4126.007157
	**Int2**^{3}	-4127.140990	-4126.024278
	**Int2**^{5}	-4127.125057	-4126.016679
	**TS1**^{1}	-4127.130142	-4125.999253
	**TS1**^{3}	-4127.111230	-4125.990790
	**Int3**^{1}	-4127.221994	-4126.089944
	**Int3**^{3}	-4127.191347	-4126.064221
	**Int3**^{5}	-4127.165274	-4126.047032

[a]Superscripted 1, 3 and 5 represents singlet, triplet, and quintet spin states, respectively.

NICS scans were performed using the GIAO method with 9 ghost atoms at PBE0-D4/6-311++G(d,p) level of theory as a part of energy refinement. To accurately place these ghost atoms in the π-stacked systems, we first determined the geometric center (centroid) of each

phenyl ring. The center of each ring ($\vec{g}_{ring}$) was calculated as the average of the 3D cartesian coordinates ($\vec{r}_i$) of all n atoms ($n = 6$) in that ring (**Equation S1** and **S2**). A midpoint vector between the two ring centroids ($\vec{G}_{stacked}$) was computed as the average of the two ring centers using **Equation S3**. The distance between D was calculated with the help of computed centroids which was further divided by 4 to get the unit distance d. To define a uniform direction for ghost atom placement, a unit direction vector ($\vec{v}_{dir}$) was constructed along the axis joining the two ring centroids. This was obtained by normalizing the vector difference between the two ring centers (**Equation S4**). This axis forms the basis for placing ghost atoms at regular intervals before, between, and beyond the two rings, ensuring symmetric sampling for NICS analysis. Finally, all the non-central ghost atoms were places at equidistance d on the same scan axis (**Figure S4** and **Table S9**). As due to the deformations in the π-stacked system throughout the elementary steps changed, the observed distances have been provided in **Table S10**. To automate the generation of Gaussian input (.com) files with uniformly placing the ghost atoms in each system, a python script was developed and used.

Single-point magnetic shielding calculations were carried out at the PBE0-GIAO/6-311++G(d,p) level of theory. The isotropic shielding values (σ_{iso}) at each ghost atom were extracted from the Gaussian output files, and the NICS(0) values were calculated using **Equation S5** and the results are provided in **Table S10**.

$$\vec{g}_{ring} = \frac{1}{n}\sum_{i=1}^{n}\vec{r}_i$$ **Equation S1**

$$\vec{r}_i = (x_i, y_i, z_i)$$ **Equation S2**

$$\vec{G}_{stacked} = \frac{\vec{g}_{ring1} + \vec{g}_{ring2}}{2}$$ **Equation S3**

$$\vec{v}_{dir} = \frac{\vec{g}_{ring1} - \vec{g}_{ring2}}{\left\| \vec{g}_{ring1} - \vec{g}_{ring2} \right\|}$$ **Equation S4**

$$NICS_{iso}(0) = -\sigma_{iso}$$ **Equation S5**

Were, $\sigma_{iso} = \frac{(\sigma_{xx}+\sigma_{yy}+\sigma_{zz})}{3}$

Table S10. Description of placed 9 ghost atoms for the NICS scan with their name and coordinate expressions.

Ghost atom	Coordinate Expression	Description
Bq+4d	$\vec{g}_{ring1} - (2d)\cdot\vec{v}_{dir}$	Above ring1 (2 units)
Bq+3d	$\vec{g}_{ring1} - (d)\cdot\vec{v}_{dir}$	Above ring1 (1 unit)

Bq+2d	$\vec{g}_{ring1}$	At ring1 center
Bq+d	$\vec{G}_{stacked} - (d) \cdot \vec{v}_{dir}$	Above π-stacked center (origin)
Bq	$\vec{G}_{stacked}$	At π-stacked center (origin)
Bq-d	$\vec{G}_{stacked} + (d) \cdot \vec{v}_{dir}$	Below π-stacked center (origin)
Bq-2d	$\vec{g}_{ring2}$	At ring2 center
Bq-3d	$\vec{g}_{ring2} + (d) \cdot \vec{v}_{dir}$	Below ring2 (1 unit)
Bq-4d	$\vec{g}_{ring2} + (2d) \cdot \vec{v}_{dir}$	Below ring2 (2 units)

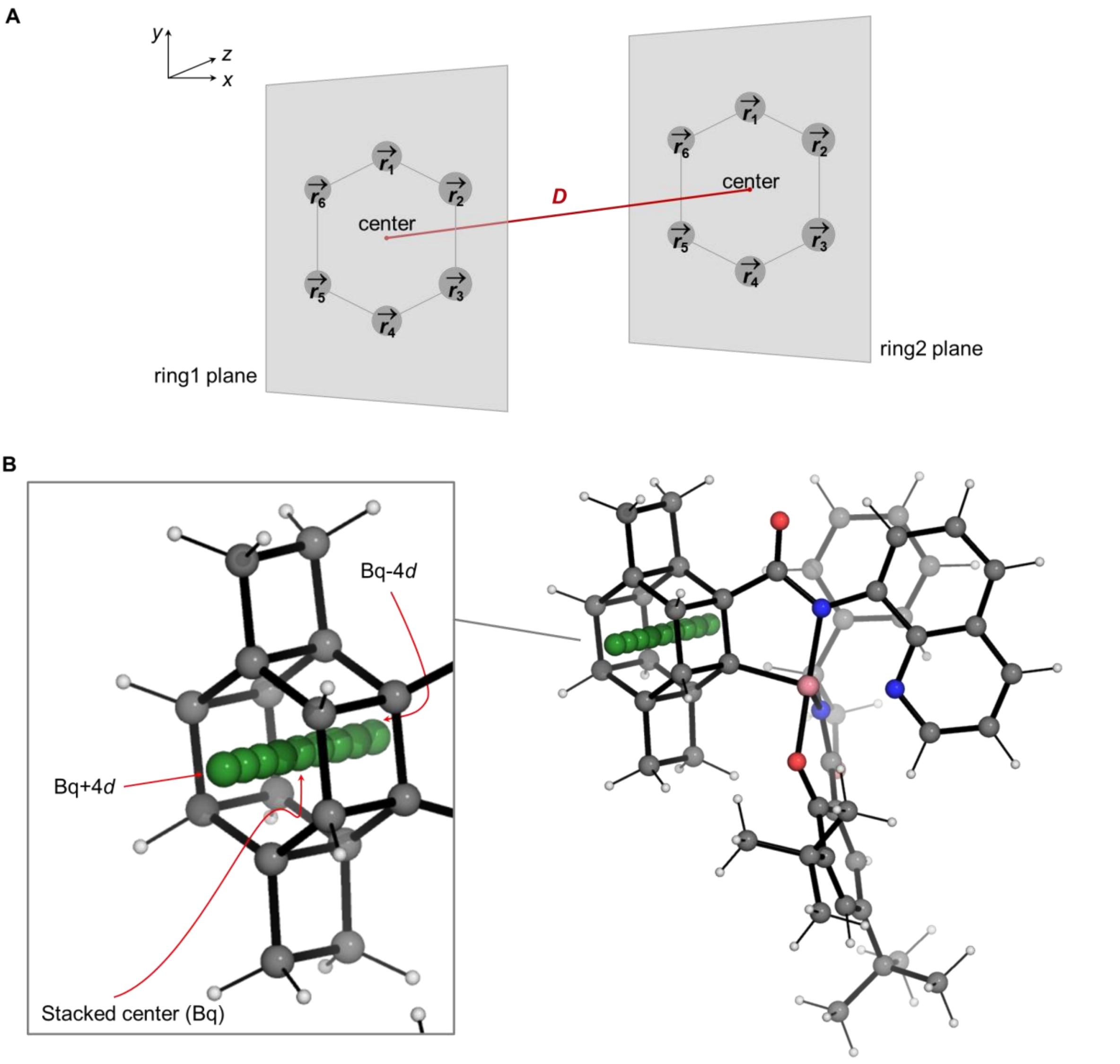

Figure S5. Geometric Setup for NICS(0): A. Schematic presentation of π-stacked ring planes with corresponding centroids and D as the distance between the centroids, B. Placed ghost atoms (in green) for NICS(0) calculation. Here Int1-*ent1* is taken for illustration purpose.

Table S11. Observed distance between ring centers (D) and unit distances (d) from the optimized geometries throughout the elementary steps (in Å).

Structures[a]		d	D
ent1	**Int1[3]**	0.395	1.581
	**Int2[3]**	0.396	1.584
	**TS1[1]**	0.395	1.579
	**Int3[3]**	0.393	1.573
ent2	**Int1[3]**	0.395	1.581
	**Int2[3]**	0.395	1.582
	**TS1[1]**	0.398	1.592
	**Int3[1]**	0.485	1.940

[a]Superscripted 1 and 3 represents singlet and triplet spin states, respectively.

Table S12. Calculated $NICS_{iso}(0)$ for the ghost atoms at the PBE0/6-311++G(d,p)//TPSS-D3(BJ)/def2-SVP level of theory (in ppm).

Structures[a]		Bq+4d	Bq+3d	Bq+2d	Bq+1d	Bq	Bq-1d	Bq-2d	Bq-3d	Bq-4d
ent1	**Int1[3]**	-0.216	0.719	1.976	4.246	5.737	4.375	2.191	0.844	-0.315
	**Int2[3]**	-0.189	0.694	1.896	4.148	5.743	4.554	2.568	1.551	0.688
	**TS1[1]**	0.227	1.338	2.738	5.181	6.837	5.475	3.255	2.277	1.848
	**Int3[3]**	-0.989	-0.311	0.583	2.516	3.617	1.582	-1.643	-4.214	-6.820
ent2	**Int1[3]**	-0.122	0.994	2.237	4.334	5.676	4.178	1.882	0.638	-0.247
	**Int2[3]**	-1.268	-0.030	1.557	4.046	5.520	4.001	1.738	0.545	-0.287
	**TS1[1]**	0.110	0.983	2.310	4.749	6.271	4.796	2.460	1.053	0.011
	**Int3[1]**	-0.398	2.717	4.326	3.584	3.542	2.475	1.770	0.566	-1.497

[a]Superscripted 1 and 3 represents singlet and triplet spin states, respectively.

Script for NICS input file generation

The following Python script was used to automate the placing of ghost atoms and generating of Gaussian input files (.com) for performing NICS calculations. The script calculates the centroids of the two planar rings from a. xyz geometry file and places ghost atoms at defined positions along the axis connecting the ring centers, enabling systematic magnetic shielding analysis in π-stacked systems. This script requires. xyz geometry file and exact atoms indices from the rings or planar geometries.

```
import numpy as np
import pandas as pd
import os

def generate_nics_com(xyz_file, ring1_indices, ring2_indices, output_com):
    """
    Generates a Gaussian '.com' input file for NICS calculations with placed ghost atoms.
    Parameters:
    xyz_file (str): Path to the input .xyz file (in standard format).
    ring1_indices (list): List of atoms indices for phenyl ring 1.
    ring2_indices (list): List of atoms indices for phenyl ring 2.
    output_com (str): Path to the output .com file.
    """

    try:
        # Read the .xyz file
        with open(xyz_file, "r") as file:
            lines = file.readlines()

        num_atoms = int(lines[0].strip())  # Total number of atoms
        atom_data = [line.strip().split() for line in lines[2:num_atoms + 2]]  # Comment line

        # Convert to df
        df = pd.DataFrame(atom_data, columns=["Element", "X", "Y", "Z"])
        df[["X", "Y", "Z"]] = df[["X", "Y", "Z"]].astype(float)  # Convert to float

        # Convert indices from 1-based to 0-based
        ring1_indices = [i - 1 for i in ring1_indices]
        ring2_indices = [i - 1 for i in ring2_indices]

        # Extract ring coordinates
        coords_ring1 = df.iloc[ring1_indices][["X", "Y", "Z"]].to_numpy()
        coords_ring2 = df.iloc[ring2_indices][["X", "Y", "Z"]].to_numpy()

        # Compute ring centers
        g_r1 = np.mean(coords_ring1, axis=0)  # ring1
        g_r2 = np.mean(coords_ring2, axis=0)  # ring2

        # Compute the midpoint between the two rings
        g_c = (g_r1 + g_r2) / 2  # Global center
        # g_c_mid = (g_r1 + g_c) / 2  # Further midpoint for spacing refinement

        # Compute spacing factor (for equidistance placement)
        x = np.linalg.norm(g_r1 - g_r2) / 4  # Distance (Euclidean) unit for ghost (Bq) atoms

        # For reference axis
        direction_vector = (g_r2 - g_r1) / np.linalg.norm(g_r2 - g_r1)

        # Compute other ghost atoms along the same alignment
        g_r1_o2 = g_r1 - direction_vector * (2 * x)
        g_r1_o1 = g_r1 - direction_vector * x
        g_r1_c_m = g_c - direction_vector * x
        g_r2_c_m = g_c + direction_vector * x
        g_r2_o1 = g_r2 + direction_vector * x
        g_r2_o2 = g_r2 + direction_vector * (2 * x)

        # Store 9 ghost atoms in a dictionary
        ghost_atoms = {
            "Ring1_Outer2": g_r1_o2,
```

```
            "Ring1_Outer1": g_r1_o1,
            "Ring1_Center": g_r1,
            "Ring1_CentralPoint_mid": g_r1_c_m,
            "CentralPoint": g_c,
            "Ring2_CentralPoint_mid": g_r2_c_m,
            "Ring2_Center": g_r2,
            "Ring2_Outer1": g_r2_o1,
            "Ring2_Outer2": g_r2_o2
        }

        # For Gaussian input file content preparation
        chk_name = os.path.splitext(output_com)[0] + ".chk"
        com_content = (
            f"%nprocshared=24\n"
            f"%mem=70GB\n"
            f"%chk={chk_name}\n"
            f"#p nmr=GIAO PBE1PBE/6-311++G(d,p)\n\n" # Selected method and level of theory
            f"NICS_calculation_input\n\n"
            f"0 1\n"                                 # Charge and spin (change accordingly)
        )

        # Add atomic coordinates
        for row in df.itertuples(index=False):
            com_content += f"{row.Element} {row.X:.6f} {row.Y:.6f} {row.Z:.6f}\n"

        # Add ghost atoms (Bq)
        for coords in ghost_atoms.values():
            com_content += f"Bq {coords[0]:.6f} {coords[1]:.6f} {coords[2]:.6f}\n"

        com_content += "\n" * 10

        # Write the Gaussian input file
        with open(output_com, "w") as com_file:
            com_file.write(com_content)

        # Check if the file was successfully created
        if os.path.exists(output_com) and os.path.getsize(output_com) > 0:
            print(f"Gaussian input file '{output_com}' SUCCESSFULLY generated!")
            print(f"Unit distance for ghost atom placement: {x:.6f} angstrom.") # The d value
        else:
            print(f"Error: Gaussian input file '{output_com}' was NOT generated.")

    except Exception as e:
        print(f"An ERROR occurred while generating '{output_com}': {e}")
```

8 X-Ray Crystallographic Data

The datasets were collected using a Bruker D8 three-circle diffractometer, which was equipped with a Bruker Photon III C7 CMOS detector and an INCOATEC microfocus source (Mo K_α radiation) in conjunction with INCOATEC Quazar mirror optics. Data integration was performed using SAINT[7], and a multi-scan absorption correction was applied via SADABS[8]. The structures were solved using SHELXT[9] and refined against F^2 using SHELXL[10] within the graphical user interface ShelXle[11]. Hydrogen atoms were placed according to geometrical criteria and refined using a riding model.

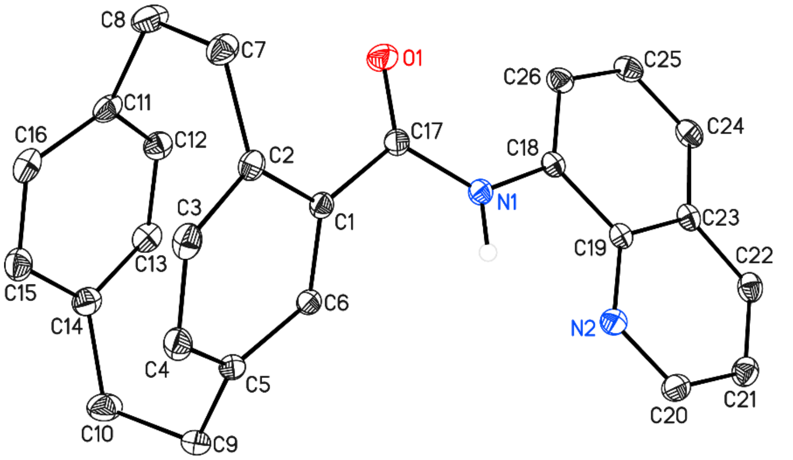

Figure S6. The asymmetric unit of **2** with thermal ellipsoids at 50% probability level. The hydrogen atoms except that bond to N1 are omitted for clarity. The hydrogen atom H1, connected to N1, was refined freely using a distance restraint.

Table S13. Crystal data and structure refinement for compound (S_p)-**1a**.

Compound	(S_p)-**1a**
CCDC	2444488
Empirical formula	$C_{26}H_{22}N_2O$
Formula weight	378.45
Temperature (K)	100(2)
Wavelength (Å)	0.71073
Crystal system	Orthorhombic
Space group	$P2_12_12_1$
a (Å)	7.742(2)
b (Å)	11.448(3)
c (Å)	21.021(4)
β (deg)	

V (Å^3)	1863.1(8)
Z	4
Density (Mg/m3)	1.349
μ (mm^{-1})	0.083
Crystal size (mm)	0.356 x 0.355 x 0.154
Crystal colour, shape	Colorless block
Θ range (deg)	2.631 to 36.592
Reflections collected	155291
Independent reflections	9163
R_{int}	0.0480
Data/restraints/parameters	9163 / 1 / 266
R_1 (I >2σ(I))	0.0340
wR_2 (all data)	0.0950
Absolute structure parameter[6]	0.11(18)
$\Delta\rho_{max}$/$\Delta\rho_{min}$(e Å^{-3})	0.442 and -0.217

Table S14. Bond lengths [Å] and angles [°] of (S_p)-**1a**.

O(1)-C(17)	1.2242(11)	C(7)-C(8)	1.5926(15)
N(1)-C(17)	1.3715(12)	C(8)-C(11)	1.5104(15)
N(1)-C(18)	1.3981(12)	C(9)-C(10)	1.5824(15)
N(2)-C(20)	1.3201(13)	C(10)-C(14)	1.5092(15)
N(2)-C(19)	1.3649(12)	C(11)-C(16)	1.3965(15)
C(1)-C(6)	1.4009(13)	C(11)-C(12)	1.4012(14)
C(1)-C(2)	1.4085(13)	C(12)-C(13)	1.3909(14)
C(1)-C(17)	1.4994(13)	C(13)-C(14)	1.4008(15)
C(5)-C(6)	1.3956(13)	C(14)-C(15)	1.3931(15)
C(5)-C(4)	1.4003(14)	C(15)-C(16)	1.3974(15)
C(5)-C(9)	1.5127(15)	C(18)-C(26)	1.3789(13)
C(7)-C(2)	1.5111(14)	C(18)-C(19)	1.4373(13)

C(19)-C(23)	1.4179(12)	C(15)-C(14)-C(10)	120.07(10)
C(20)-C(21)	1.4124(14)	C(13)-C(14)-C(10)	121.24(9)
C(21)-C(22)	1.3715(15)	C(14)-C(15)-C(16)	120.90(9)
C(22)-C(23)	1.4159(14)	C(11)-C(16)-C(15)	120.35(9)
C(23)-C(24)	1.4169(14)	O(1)-C(17)-N(1)	123.60(9)
C(24)-C(25)	1.3683(15)	O(1)-C(17)-C(1)	123.90(8)
C(25)-C(26)	1.4168(13)	N(1)-C(17)-C(1)	112.50(8)
C(2)-C(3)	1.4055(13)	C(26)-C(18)-N(1)	126.16(8)
C(3)-C(4)	1.3924(15)	C(26)-C(18)-C(19)	119.55(8)
C(17)-N(1)-C(18)	129.08(8)	N(1)-C(18)-C(19)	114.28(8)
C(20)-N(2)-C(19)	117.75(8)	N(2)-C(19)-C(23)	123.08(8)
C(6)-C(1)-C(2)	119.55(8)	N(2)-C(19)-C(18)	117.33(8)
C(6)-C(1)-C(17)	117.59(8)	C(23)-C(19)-C(18)	119.59(8)
C(2)-C(1)-C(17)	122.38(8)	N(2)-C(20)-C(21)	123.62(9)
C(6)-C(5)-C(4)	116.39(9)	C(22)-C(21)-C(20)	118.94(9)
C(6)-C(5)-C(9)	119.89(9)	C(21)-C(22)-C(23)	119.56(9)
C(4)-C(5)-C(9)	122.67(9)	C(22)-C(23)-C(24)	123.35(9)
C(5)-C(6)-C(1)	122.11(8)	C(22)-C(23)-C(19)	117.03(8)
C(2)-C(7)-C(8)	112.24(8)	C(24)-C(23)-C(19)	119.61(9)
C(11)-C(8)-C(7)	112.85(8)	C(25)-C(24)-C(23)	119.39(9)
C(5)-C(9)-C(10)	112.89(8)	C(24)-C(25)-C(26)	122.15(9)
C(14)-C(10)-C(9)	112.09(8)	C(18)-C(26)-C(25)	119.67(9)
C(16)-C(11)-C(12)	117.17(9)	C(3)-C(2)-C(1)	115.95(8)
C(16)-C(11)-C(8)	121.49(9)	C(3)-C(2)-C(7)	118.42(8)
C(12)-C(11)-C(8)	120.09(9)	C(1)-C(2)-C(7)	124.52(8)
C(13)-C(12)-C(11)	120.89(9)	C(4)-C(3)-C(2)	121.93(9)
C(12)-C(13)-C(14)	120.37(9)	C(3)-C(4)-C(5)	120.08(8)
C(15)-C(14)-C(13)	117.31(9)		

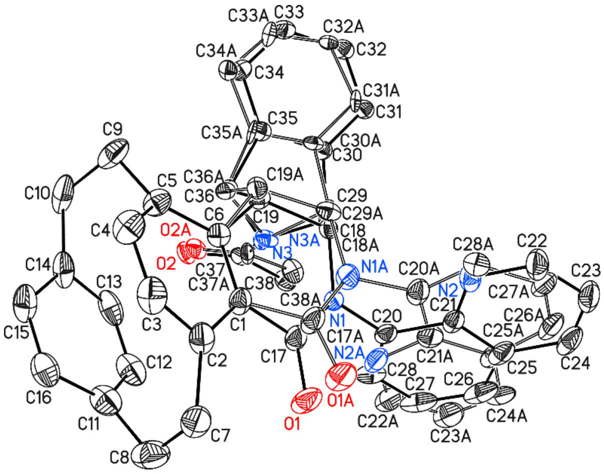

Figure S7. The asymmetric unit of **3** is depicted with thermal ellipsoids at a 50% probability level. Hydrogen atoms have been omitted for clarity. Except for the dibenzenacyclohexaphanyl moiety, all other parts of the molecule are disordered over two positions. In the minor position the quinoline group is inverted with an occupation of 0.077(2). The disorder was refined using distance restraints and restraints for the anisotropic displacement parameters.

Table S15. Crystal data and structure refinement for compound **12**.

Compound	**12**
CCDC	2444489
Empirical formula	$C_{38}H_{31}N_3O_2$
Formula weight	561.66
Temperature (K)	100(2)
Wavelength (Å)	0.71073
Crystal system	Monoclinic
Space group	$P2_1$
a (Å)	10.723(2)
b (Å)	12.046(4)
c (Å)	11.116(3)
β (deg)	100.77(2)
V (Å^3)	1410.6(7)
Z	2
Density (Mg/m3)	1.322
μ (mm^{-1})	0.082
Crystal size (mm)	0.441 x 0.240 x 0.162

Crystal colour, shape	Colorless block
Θ range (deg)	1.865 to 30.570
Reflections collected	196450
Independent reflections	8644
R_{int}	0.0380
Data/restraints/parameters	8644 / 1476 / 627
R_1 ($I > 2\sigma(I)$)	0.0302
wR_2 (all data)	0.0845
Absolute structure parameter[6]	0.09(14)
$\Delta\rho_{max}/\Delta\rho_{min}$(e Å^{-3})	0.238 and -0.161

Table S16. Bond lengths [Å] and angles [°] of **6**.

C(1)-C(6)	1.3999(17)	C(11)-C(12)	1.400(2)
C(1)-C(2)	1.4233(19)	C(12)-C(13)	1.375(3)
C(1)-C(17)	1.488(2)	C(13)-C(14)	1.402(2)
C(1)-C(17A)	1.510(16)	C(14)-C(15)	1.402(2)
C(8)-C(11)	1.506(3)	C(15)-C(16)	1.385(3)
C(8)-C(7)	1.571(3)	C(18)-N(1)	1.466(2)
C(9)-C(5)	1.516(2)	C(18)-C(19)	1.558(2)
C(9)-C(10)	1.599(3)	C(18)-C(29)	1.564(2)
C(10)-C(14)	1.508(3)	C(19)-C(36)	1.575(2)
C(2)-C(3)	1.393(2)	C(36)-N(3)	1.480(3)
C(2)-C(7)	1.514(2)	C(36)-C(35)	1.522(3)
C(3)-C(4)	1.387(2)	C(30)-C(31)	1.385(3)
C(4)-C(5)	1.390(2)	C(30)-C(35)	1.401(3)
C(5)-C(6)	1.4132(19)	C(30)-C(29)	1.521(2)
C(6)-C(19A)	1.51(2)	C(31)-C(32)	1.410(2)
C(6)-C(19)	1.513(2)	C(32)-C(33)	1.391(3)
C(11)-C(16)	1.397(2)	C(33)-C(34)	1.402(3)

C(34)-C(35)	1.388(2)	C(34A)-C(35A)	1.41(2)
C(29)-N(3)	1.484(2)	C(29A)-N(3A)	1.48(2)
N(3)-C(37)	1.368(2)	N(3A)-C(37A)	1.32(2)
C(37)-O(2)	1.228(2)	C(37A)-O(2A)	1.22(2)
C(37)-C(38)	1.508(3)	C(37A)-C(38A)	1.53(2)
C(17)-O(1)	1.228(2)	C(17A)-O(1A)	1.234(19)
C(17)-N(1)	1.371(2)	C(17A)-N(1A)	1.355(18)
N(1)-C(20)	1.4341(17)	N(1A)-C(20A)	1.443(17)
C(20)-C(28)	1.372(2)	C(20A)-C(28A)	1.36(2)
C(20)-C(21)	1.425(2)	C(20A)-C(21A)	1.386(19)
C(21)-N(2)	1.359(3)	C(21A)-N(2A)	1.35(2)
C(21)-C(25)	1.423(3)	C(21A)-C(25A)	1.44(2)
N(2)-C(22)	1.326(2)	N(2A)-C(22A)	1.38(2)
C(22)-C(23)	1.405(3)	C(22A)-C(23A)	1.41(2)
C(23)-C(24)	1.350(4)	C(23A)-C(24A)	1.32(2)
C(24)-C(25)	1.424(3)	C(24A)-C(25A)	1.39(2)
C(25)-C(26)	1.404(3)	C(25A)-C(26A)	1.44(2)
C(26)-C(27)	1.367(4)	C(26A)-C(27A)	1.37(2)
C(27)-C(28)	1.412(3)	C(27A)-C(28A)	1.40(2)
C(18A)-N(1A)	1.425(19)		
C(18A)-C(19A)	1.53(2)	C(6)-C(1)-C(2)	120.50(12)
C(18A)-C(29A)	1.55(2)	C(6)-C(1)-C(17)	119.94(12)
C(19A)-C(36A)	1.58(2)	C(2)-C(1)-C(17)	119.22(13)
C(36A)-N(3A)	1.49(2)	C(6)-C(1)-C(17A)	113.9(7)
C(36A)-C(35A)	1.51(2)	C(2)-C(1)-C(17A)	123.5(7)
C(30A)-C(35A)	1.35(2)	C(11)-C(8)-C(7)	112.56(14)
C(30A)-C(31A)	1.44(2)	C(5)-C(9)-C(10)	112.60(14)
C(30A)-C(29A)	1.52(2)	C(14)-C(10)-C(9)	113.24(13)
C(31A)-C(32A)	1.40(2)	C(3)-C(2)-C(1)	116.05(14)
C(32A)-C(33A)	1.33(2)	C(3)-C(2)-C(7)	116.25(14)
C(33A)-C(34A)	1.32(2)	C(1)-C(2)-C(7)	126.66(14)

C(4)-C(3)-C(2)	121.29(13)	C(31)-C(30)-C(29)	133.20(17)
C(3)-C(4)-C(5)	120.87(14)	C(35)-C(30)-C(29)	106.15(17)
C(4)-C(5)-C(6)	117.03(13)	C(30)-C(31)-C(32)	117.9(2)
C(4)-C(5)-C(9)	118.43(13)	C(33)-C(32)-C(31)	121.26(19)
C(6)-C(5)-C(9)	123.73(12)	C(32)-C(33)-C(34)	120.53(19)
C(1)-C(6)-C(5)	119.76(12)	C(35)-C(34)-C(33)	117.95(19)
C(1)-C(6)-C(19A)	127.9(10)	C(34)-C(35)-C(30)	121.65(18)
C(5)-C(6)-C(19A)	111.7(10)	C(34)-C(35)-C(36)	133.1(2)
C(1)-C(6)-C(19)	120.85(13)	C(30)-C(35)-C(36)	105.23(18)
C(5)-C(6)-C(19)	119.37(13)	N(3)-C(29)-C(30)	100.90(17)
C(2)-C(7)-C(8)	112.50(14)	N(3)-C(29)-C(18)	97.89(14)
C(16)-C(11)-C(12)	117.06(17)	C(30)-C(29)-C(18)	109.04(16)
C(16)-C(11)-C(8)	121.75(16)	C(37)-N(3)-C(36)	120.68(19)
C(12)-C(11)-C(8)	119.73(16)	C(37)-N(3)-C(29)	125.16(18)
C(13)-C(12)-C(11)	121.31(16)	C(36)-N(3)-C(29)	97.22(17)
C(12)-C(13)-C(14)	120.38(14)	O(2)-C(37)-N(3)	120.6(2)
C(15)-C(14)-C(13)	117.01(17)	O(2)-C(37)-C(38)	122.8(2)
C(15)-C(14)-C(10)	120.65(16)	N(3)-C(37)-C(38)	116.53(19)
C(13)-C(14)-C(10)	121.01(14)	O(1)-C(17)-N(1)	119.62(15)
C(16)-C(15)-C(14)	120.97(16)	O(1)-C(17)-C(1)	122.33(15)
C(15)-C(16)-C(11)	120.33(15)	N(1)-C(17)-C(1)	118.05(14)
N(1)-C(18)-C(19)	112.45(14)	C(17)-N(1)-C(20)	117.24(12)
N(1)-C(18)-C(29)	108.93(13)	C(17)-N(1)-C(18)	126.18(13)
C(19)-C(18)-C(29)	102.06(13)	C(20)-N(1)-C(18)	116.32(12)
C(6)-C(19)-C(18)	116.45(15)	C(28)-C(20)-C(21)	120.26(16)
C(6)-C(19)-C(36)	119.56(19)	C(28)-C(20)-N(1)	120.34(16)
C(18)-C(19)-C(36)	101.91(15)	C(21)-C(20)-N(1)	119.37(13)
N(3)-C(36)-C(35)	100.86(19)	N(2)-C(21)-C(25)	122.54(17)
N(3)-C(36)-C(19)	101.28(17)	N(2)-C(21)-C(20)	119.05(14)
C(35)-C(36)-C(19)	104.1(2)	C(25)-C(21)-C(20)	118.41(16)
C(31)-C(30)-C(35)	120.64(18)	C(22)-N(2)-C(21)	117.1(2)

N(2)-C(22)-C(23)	124.5(2)	C(37A)-N(3A)-C(29A)	125(3)
C(24)-C(23)-C(22)	118.85(18)	C(37A)-N(3A)-C(36A)	115(3)
C(23)-C(24)-C(25)	119.5(2)	C(29A)-N(3A)-C(36A)	97.6(19)
C(26)-C(25)-C(21)	119.6(2)	O(2A)-C(37A)-N(3A)	127(3)
C(26)-C(25)-C(24)	123.0(2)	O(2A)-C(37A)-C(38A)	114(3)
C(21)-C(25)-C(24)	117.3(2)	N(3A)-C(37A)-C(38A)	119(2)
C(27)-C(26)-C(25)	121.0(2)	O(1A)-C(17A)-N(1A)	118.1(15)
C(26)-C(27)-C(28)	119.9(2)	O(1A)-C(17A)-C(1)	121.3(14)
C(20)-C(28)-C(27)	120.8(2)	N(1A)-C(17A)-C(1)	120.6(13)
N(1A)-C(18A)-C(19A)	116.3(17)	C(17A)-N(1A)-C(18A)	127.4(15)
N(1A)-C(18A)-C(29A)	110.4(17)	C(17A)-N(1A)-C(20A)	116.1(14)
C(19A)-C(18A)-C(29A)	103.6(16)	C(18A)-N(1A)-C(20A)	114.2(14)
C(6)-C(19A)-C(18A)	113.2(18)	C(28A)-C(20A)-C(21A)	112.3(17)
C(6)-C(19A)-C(36A)	111(2)	C(28A)-C(20A)-N(1A)	131.2(19)
C(18A)-C(19A)-C(36A)	101.7(17)	C(21A)-C(20A)-N(1A)	116.3(16)
N(3A)-C(36A)-C(35A)	99(2)	N(2A)-C(21A)-C(20A)	114.2(16)
N(3A)-C(36A)-C(19A)	102(2)	N(2A)-C(21A)-C(25A)	121.1(17)
C(35A)-C(36A)-C(19A)	103(3)	C(20A)-C(21A)-C(25A)	124.7(17)
C(35A)-C(30A)-C(31A)	127(2)	C(21A)-N(2A)-C(22A)	114.2(19)
C(35A)-C(30A)-C(29A)	107.3(19)	N(2A)-C(22A)-C(23A)	125(2)
C(31A)-C(30A)-C(29A)	126(2)	C(24A)-C(23A)-C(22A)	121(2)
C(32A)-C(31A)-C(30A)	110(2)	C(23A)-C(24A)-C(25A)	116(2)
C(33A)-C(32A)-C(31A)	120(2)	C(24A)-C(25A)-C(21A)	123(2)
C(34A)-C(33A)-C(32A)	131(3)	C(24A)-C(25A)-C(26A)	120(2)
C(33A)-C(34A)-C(35A)	112(2)	C(21A)-C(25A)-C(26A)	117(2)
C(30A)-C(35A)-C(34A)	119(2)	C(27A)-C(26A)-C(25A)	118.4(19)
C(30A)-C(35A)-C(36A)	107(2)	C(26A)-C(27A)-C(28A)	118(2)
C(34A)-C(35A)-C(36A)	132(3)	C(20A)-C(28A)-C(27A)	128(2)
N(3A)-C(29A)-C(30A)	98(2)		
N(3A)-C(29A)-C(18A)	97.8(17)		
C(30A)-C(29A)-C(18A)	110(2)		

References

1. Lennartz, P.; Raabe, G.; Bolm, C. Palladium-Catalyzed C−H Bond Acetoxylation: An Approach to *ortho*-Substituted Hydroxy [2.2]Paracyclophane Derivatives. *Adv. Synth. Catal.* **2012**, *354*, 3237–3249.
2. Gandeepan, P.; Rajamalli, P.; Cheng, C.-H. Diastereoselective [3+2] Annulation of Aromatic/Vinylic Amides with Bicyclic Alkenes through Cobalt-Catalyzed C−H Activation and Intramolecular Nucleophilic Addition. *Angew. Chem. Int. Ed.* **2016**, *55*, 4308−4311.
3. Skhiri, A.; Chatani, N. Nickel-Catalyzed Reaction of Benzamides with Bicyclic Alkenes: Cleavage of C–H and C–N Bonds. *Org. Lett.* **2019**, *21*, 1774−1778.
4. Cui, Y.; Zhao, L.; Wu, W.; Li, W.; Yi, J.; Li, C. Synthesis of Naphthalene-Substituted Aromatic Esters via Rh(III)-Catalyzed C–H Bond Naphthylation and Cascade Directing Group Transformation. *Chem. Commun.* **2022**, *58*, 13230−13233.
5. Arrayás, R. G.; Cabrera, S.; Carretero, J. C. Copper-Catalyzed Anti-Stereocontrolled Ring-Opening of Azabicyclic Alkenes with Grignard Reagents. *Org. Lett.* **2005**, *7*, 219−221.
6. von Münchow, T.; Pandit, N. K.; Dana, S.; Boos, P.; Peters, S. E.; Boucat, J.; Liu, Y.-R.; Scheremetjew, A.; Ackermann, L. Enantioselective C–H Annulations Enabled by Either Nickel- or Cobalt-Electrocatalyzed C–H Activation for Catalyst-Controlled Chemodivergence. *Nat. Catal.* **2025**, *8*, 257–269.
7. Bruker AXS Inc. *Bruker Apex CCD, SAINT v8.40B*; Bruker AXS Inc.: Madison, WI, USA, **2019**.
8. Krause, L.; Herbst-Irmer, R.; Sheldrick, G. M.; Stalke, D. *J. Appl. Crystallogr.* **2015**, *48*, 3–10.
9. Sheldrick, G. M. *Acta Crystallogr.*, Sect. A **2015**, *71*, 3–8.
10. Sheldrick, G. M. *Acta Crystallogr.*, Sect. C **2015**, *71*, 3–8.
11. Hübschle, C. B.; Sheldrick, G. M.; Dittrich, B. *J. Appl. Crystallogr.* **2011**, *44*, 1281–1284.
12. Gaussian 16, Revision A.03, M. J. Frisch, G. W. Trucks, H. B. Schlegel, G. E. Scuseria, M. A. Robb, J. R. Cheeseman, G. Scalmani, V. Barone, G. A. Petersson, H. Nakatsuji, X. Li, M. Caricato, A. V. Marenich, J. Bloino, B. G. Janesko, R. Gomperts, B. Mennucci, H. P. Hratchian, J. V. Ortiz, A. F. Izmaylov, J. L. Sonnenberg, D. Williams-Young, F. Ding, F. Lipparini, F. Egidi, J. Goings, B. Peng, A. Petrone, T. Henderson, D. Ranasinghe, V. G. akrzewski, J. Gao, N. Rega, G. Zheng, W. Liang, M. Hada, M. Ehara, K. Toyota, R. Fukuda, J. Hasegawa, M. Ishida, T. Nakajima, Y. Honda, O. Kitao, H. Nakai, T. Vreven, K. Throssell, J. A. Montgomery, Jr., J. E. Peralta, F. Ogliaro, M. J. Bearpark, J. J. Heyd, E. N. Brothers, K. N. Kudin, V. N. Staroverov, T. A. Keith, R. Kobayashi, J. Normand, K. Raghavachari, A. P. Rendell, J. C. Burant, S. S. Iyengar, J. Tomasi, M. Cossi, J. M. Millam, M. Klene, C. Adamo, R. Cammi, J. W. Ochterski, R. L. Martin, K. Morokuma, O. Farkas, J. B. Foresman, D. J. Fox, Gaussian, Inc., Wallingford CT, **2016**.
13. Tao, J.; Perdew, J. P.; Staroverov, V. N.; Scuseria, G. E. Climbing the Density Functional Ladder: Nonempirical Meta–Generalized Gradient Approximation Designed for Molecules and Solids. *Phys. Rev. Lett.* **2003**, *91*, 146401.
14. (a) Grimme, S.; Ehrlich, S.; Goerigk, L. Effect of the Damping Function in Dispersion Corrected Density Functional Theory. *J Comput. Chem.* **2011**, *32*, 1456–1465. (b) Grimme, S.; Antony, J.; Ehrlich, S.; Krieg, H.

A Consistent and Accurate Ab Initio Parametrization of Density Functional Dispersion Correction (DFT-D) for the 94 Elements H-Pu. *J. Chem. Phys.* **2010**, *132*, 154104.

15. (a) Weigend, F. Accurate Coulomb-Fitting Basis Sets for H to Rn. *Phys. Chem. Chem. Phys.* **2006**, *8*, 1057. (b) Weigend, F.; Ahlrichs, R. Balanced Basis Sets of Split Valence, Triple Zeta Valence and Quadruple Zeta Valence Quality for H to Rn: Design and Assessment of Accuracy. *Phys. Chem. Chem. Phys.* **2005**, *7*, 3297–3305. (c) Schäfer, A.; Huber, C.; Ahlrichs, R. Fully Optimized Contracted Gaussian Basis Sets of Triple Zeta Valence Quality for Atoms Li to Kr. *J. Chem. Phys.* **1994**, *100*, 5829–5835. (d) Schäfer, A.; Horn, H.; Ahlrichs, R. Fully Optimized Contracted Gaussian Basis Sets for Atoms Li to Kr. *J. Chem. Phys.* **1992**, *97*, 2571–2577.
16. Fukui, K. The Path of Chemical Reactions - the IRC Approach. *Acc. Chem. Res.* **1981**, *14*, 363–368.
17. Zhao, Y.; Truhlar, D. G. Design of Density Functionals That Are Broadly Accurate for Thermochemistry, Thermochemical Kinetics, and Nonbonded Interactions. *J. Phys. Chem. A* **2005**, *109*, 5656–5667.
18. Caldeweyher, E.; Bannwarth, C.; Grimme, S. Extension of the D3 Dispersion Coefficient Model. *J. Chem. Phys.* **2017**, *147*, 034112. (b) Caldeweyher, E.; Ehlert, S.; Hansen, A.; Neugebauer, H.; Spicher, S.; Bannwarth, C.; Grimme, S. A Generally Applicable Atomic-Charge Dependent London Dispersion Correction. *J. Chem. Phys.* **2019**, *150*, 154122
19. Marenich, A. V.; Cramer, C. J.; Truhlar, D. G. Universal Solvation Model Based on Solute Electron Density and on a Continuum Model of the Solvent Defined by the Bulk Dielectric Constant and Atomic Surface Tensions. *J. Phys. Chem. B* **2009**, *113*, 6378–6396.
20. Gershoni-Poranne R.; Stanger A. NICS—Nucleus-independent Chemical Shift. *Aromaticity: Modern Computational Methods and Applications*; Fernandez I., Ed.; Elsevier, 2022; pp 99−154. DOI: 10.1016/C2019-0-04193-3.
21. Yanai, T.; Tew, D. P.; Handy, N. C. A new hybrid exchange–correlation functional using the Coulomb-attenuating method (CAM-B3LYP). *Chem. Phys. Lett.* **2004**, *393*, 51−57.
22. Adamo, C.; Barone, V. Toward reliable density functional methods without adjustable parameters: The PBE0 model. *J. Chem. Phys.* **1999**, *110*, 6158−6170.
23. (a) London F. The quantic theory of inter-atomic currents in aromatic combinations. *J. Phys. Radium*, **1973**, *8* 397-409. (b) McWeeny R. Perturbation Theory for Fock-Dirac Density Matrix. *Phys. Rev.* **1962**, *126*, 1028. (c) Ditchfield D. Self-consistent perturbation theory of diamagnetism. 1. Gauge-invariant LCAO method for N.M.R. chemical shifts. *Mol. Phys.* **1974**, *27*, 789-807. (d) Wolinski K.; Hilton J. F.; Pulay P., Efficient Implementation of the Gauge-Independent Atomic Orbital Method for NMR Chemical Shift Calculations, *J. Am. Chem. Soc.* **1990**, *112*, 8251-60. (e) Cheeseman J. R.; Trucks G. W.; Keith T. A.; Frisch M. J. A Comparison of Models for Calculating Nuclear Magnetic Resonance Shielding Tensors. *J. Chem. Phys.* **1996**, *104*, 5497-509.
24. The PyMOL Molecular Graphics System, Version 2.5.8 Schrödinger, LLC.

Cartesian coordinates of the optimized structures

Alkene (7-oxabenzonorbornadiene)

Lowest frequency = 133.5696 cm^{-1}

Charge = 0, Multiplicity = 1

19

C	1.338508	1.154844	0.457477
O	1.543043	0.319029	1.620280
C	2.253752	0.454195	-0.569596
C	2.195677	-0.850396	-0.257093
C	1.245945	-0.931487	0.956904
C	-0.165648	-0.617443	0.424313
C	-0.104474	0.753961	0.096220
C	-1.201074	1.417401	-0.436184
C	-2.390612	0.675566	-0.631399
C	-2.451380	-0.684117	-0.306104
C	-1.324762	-1.354870	0.227092
H	1.531716	2.211922	0.684512
H	2.747978	0.938951	-1.414446
H	2.630119	-1.704329	-0.781535
H	1.354164	-1.783918	1.640983

H	-1.161362	2.481915	-0.692218
H	-3.276916	1.174353	-1.038187
H	-3.384580	-1.236241	-0.461426
H	-1.380093	-2.419336	0.480408

Int1-*ent1*

Lowest frequency = 15.3754 cm^{-1}

Charge = 0, Multiplicity = 1

104

C	-1.365649	-1.552593	0.391762
C	-3.862915	-2.071671	-0.512703
C	-0.905351	-2.668372	-0.583137
C	-3.366014	-3.154795	-1.505508
C	-1.845700	-3.461584	-1.537379
H	-3.879589	-3.195600	-2.481762
H	-1.433459	-3.703248	-2.532689
N	-2.081282	0.929225	0.896708
C	-0.799235	2.882603	1.270502
C	1.499447	2.500101	1.231825

C	-0.622805	4.288062	1.474902
C	-2.101875	2.299573	1.152675
C	1.769092	3.877144	1.406881
H	2.314999	1.778143	1.144236
C	0.713798	4.767783	1.535455
C	-1.779657	5.108763	1.593154
C	-3.216828	3.139523	1.286776
H	2.808548	4.215612	1.446997
H	0.895290	5.838215	1.682741
C	-3.036627	4.526885	1.508717
H	-1.661161	6.185422	1.752474
H	-4.212064	2.704409	1.211412
H	-3.928834	5.155500	1.603853
N	0.257982	2.015263	1.169128
C	-3.230674	0.178760	0.734570
O	-4.383430	0.592990	0.912329
O	1.276331	-0.603727	1.152374
C	2.401843	-0.473507	0.493899
C	2.428282	-0.125333	-0.900853
C	3.662165	-0.695492	1.157331
C	3.649031	-0.029193	-1.613184

C	1.184861	0.078833	-1.606212
C	4.828686	-0.566357	0.402440
C	4.867904	-0.240519	-0.979835
H	3.593196	0.220899	-2.674394
N	-0.012857	0.211793	-1.088556
O	1.215164	0.139075	-2.960101
H	5.783096	-0.727470	0.910161
C	-1.021978	0.375542	-2.153907
C	-0.157572	0.182566	-3.432711
H	-1.768152	-0.431436	-2.059196
C	-1.725514	1.716203	-2.076531
H	-0.366557	-0.771593	-3.944103
C	-0.981786	2.904048	-1.952734
C	-3.125493	1.785436	-2.148060
C	-1.631357	4.143709	-1.908601
H	0.109980	2.855416	-1.870738
C	-3.776802	3.025435	-2.098161
H	-3.710582	0.861788	-2.216607
C	-3.030378	4.206427	-1.983661
H	-1.045286	5.061527	-1.797386
H	-4.870159	3.065300	-2.133541

H	-3.538619	5.174668	-1.931418
C	-2.901390	-1.249425	0.404274
Co	-0.400617	0.111434	0.709928
C	3.702464	-1.044312	2.657467
C	6.223229	-0.136022	-1.697882
C	6.963686	-1.492347	-1.597261
H	7.946406	-1.434725	-2.100048
H	7.138281	-1.781077	-0.546315
H	6.374406	-2.294014	-2.075654
C	7.077358	0.966716	-1.025218
H	7.253328	0.747367	0.042227
H	8.062203	1.049842	-1.520234
H	6.571418	1.945949	-1.091777
C	6.055126	0.218033	-3.187783
H	5.467614	-0.548718	-3.722430
H	5.551900	1.192084	-3.318829
H	7.045826	0.282391	-3.671159
C	2.928714	-2.360818	2.914483
H	1.875075	-2.252698	2.618586
H	3.374020	-3.191923	2.339559
H	2.971891	-2.621096	3.987821

C	5.146492	-1.237513	3.162657
H	5.658642	-2.063345	2.637844
H	5.751540	-0.321151	3.043195
H	5.124471	-1.486037	4.238064
C	3.061073	0.101314	3.480941
H	3.622427	1.042643	3.341106
H	2.015021	0.264748	3.181171
H	3.082374	-0.150720	4.556882
H	-0.247403	1.018078	-4.142958
C	0.584626	-3.097568	-0.683289
H	1.321004	-2.308575	-0.503092
H	0.819702	-3.578367	-1.649037
C	-5.394209	-1.804285	-0.511175
H	-5.683193	-0.771253	-0.275564
H	-5.876450	-2.121282	-1.452573
C	0.385399	-4.094442	0.501392
H	0.928677	-3.810878	1.415829
H	0.597889	-5.155791	0.283000
C	-5.552648	-2.819659	0.671422
H	-5.851555	-2.344390	1.620884
H	-6.216469	-3.682800	0.487460

C	-1.114806	-3.728551	0.541009
C	-2.040300	-4.548222	-0.402307
C	-1.584019	-2.667703	1.554776
C	-3.563941	-4.245876	-0.370019
H	-1.768506	-5.609671	-0.534386
C	-3.091843	-2.345684	1.552028
H	-1.075961	-2.649875	2.532830
C	-4.035440	-3.129751	0.599407
H	-4.223847	-5.123652	-0.478528
H	-3.518968	-2.049231	2.524454

Int1-*ent1*

Lowest frequency = 15.5964 cm^{-1}

Charge = 0, Multiplicity = 3

104

C	1.499023	-1.459603	-0.340088
C	4.080685	-1.815286	0.298988
C	1.177867	-2.504665	0.755769
C	3.740415	-2.833938	1.419241

C	2.247281	-3.192787	1.648333
H	4.359886	-2.782059	2.331344
H	1.961696	-3.380354	2.697965
N	2.015409	1.043771	-0.972000
C	0.641703	2.959378	-1.154165
C	-1.627205	2.510607	-0.873592
C	0.403709	4.363109	-1.315959
C	1.964746	2.411590	-1.220761
C	-1.955647	3.879552	-1.012737
H	-2.409940	1.765936	-0.709691
C	-0.945749	4.803349	-1.229546
C	1.513763	5.219782	-1.556543
C	3.028133	3.286252	-1.494552
H	-3.004485	4.184330	-0.950948
H	-1.171723	5.869315	-1.343969
C	2.786079	4.671423	-1.651649
H	1.346799	6.294832	-1.679142
H	4.035466	2.879789	-1.566999
H	3.641038	5.326776	-1.852209
N	-0.372036	2.065226	-0.931358
C	3.201894	0.328291	-0.993531

O	4.296811	0.777750	-1.349037
O	-1.248813	-0.710862	-1.017658
C	-2.431185	-0.571323	-0.465813
C	-2.607295	-0.184270	0.913281
C	-3.615520	-0.836963	-1.244695
C	-3.898947	-0.110525	1.490685
C	-1.462661	0.113355	1.750955
C	-4.856776	-0.731104	-0.617181
C	-5.041741	-0.380194	0.746155
H	-3.960344	0.174578	2.542492
N	-0.227750	0.276951	1.361548
O	-1.655495	0.196947	3.096326
H	-5.750585	-0.926132	-1.215277
C	0.643706	0.404274	2.544138
C	-0.388630	0.605788	3.681453
H	1.185134	-0.553003	2.666837
C	1.658545	1.511807	2.350508
H	-0.205713	-0.022308	4.565231
C	1.241887	2.844561	2.175615
C	3.024561	1.203006	2.253289
C	2.180025	3.853655	1.929878

H	0.174468	3.091530	2.205393
C	3.964949	2.209241	1.991986
H	3.349839	0.161566	2.355105
C	3.543383	3.536336	1.835604
H	1.845250	4.884938	1.779685
H	5.023520	1.950634	1.890943
H	4.273724	4.322149	1.617921
C	2.994738	-1.098598	-0.566616
Co	0.378785	0.144830	-0.527189
C	-3.493260	-1.184022	-2.740535
C	-6.463423	-0.303499	1.325413
C	-7.147362	-1.686002	1.186636
H	-8.176388	-1.649399	1.588399
H	-7.207323	-2.003679	0.131309
H	-6.584345	-2.456608	1.741516
C	-7.279817	0.754814	0.543223
H	-7.344254	0.504834	-0.529926
H	-8.310113	0.817377	0.938454
H	-6.812645	1.751359	0.632611
C	-6.454868	0.089550	2.815002
H	-5.899049	-0.644187	3.424503

H	-5.998900	1.083154	2.969905
H	-7.490212	0.130661	3.196449
C	-2.670757	-2.484396	-2.921477
H	-1.657761	-2.359745	-2.513184
H	-3.161244	-3.328156	-2.404156
H	-2.595894	-2.735765	-3.995172
C	-4.873350	-1.404406	-3.392544
H	-5.419822	-2.243560	-2.926499
H	-5.506589	-0.501213	-3.334302
H	-4.734802	-1.647146	-4.460480
C	-2.799300	-0.014158	-3.484992
H	-3.394359	0.911952	-3.389966
H	-1.793280	0.171708	-3.080490
H	-2.708392	-0.253160	-4.560254
H	-0.475381	1.662480	3.988540
C	-0.282905	-2.958132	1.024186
H	-1.042057	-2.194576	0.825557
H	-0.430786	-3.351586	2.045064
C	5.586012	-1.487321	0.103970
H	5.788356	-0.467105	-0.250320
H	6.187060	-1.693385	1.007012

C	-0.164954	-4.045637	-0.089180
H	-0.815316	-3.863198	-0.958927
H	-0.302830	-5.090036	0.241222
C	5.666165	-2.596212	-1.000865
H	5.851476	-2.199451	-2.013290
H	6.378663	-3.418433	-0.811913
C	1.306588	-3.635695	-0.319528
C	2.361219	-4.349603	0.571846
C	1.619400	-2.628246	-1.442149
C	3.858669	-4.001648	0.349217
H	2.145889	-5.405840	0.808136
C	3.109489	-2.266210	-1.644181
H	0.998234	-2.681571	-2.350546
C	4.178207	-2.948594	-0.744677
H	4.559857	-4.847779	0.449333
H	3.412648	-2.043271	-2.680397

Int1-*ent1*

Lowest frequency = 10.8408 cm^{-1}

Charge = 0, Multiplicity = 5

104

C	-1.761702	-1.643140	0.291578
C	-4.241016	-1.876403	-0.615696
C	-1.338307	-2.653989	-0.779981
C	-3.818492	-2.914883	-1.689432
C	-2.323761	-3.321175	-1.765979
H	-4.343539	-2.858015	-2.657959
H	-1.937545	-3.539597	-2.775634
N	-2.269088	0.977320	0.821646
C	-0.928147	2.889704	1.229045
C	1.362368	2.512942	0.991497
C	-0.723950	4.277876	1.525989
C	-2.247916	2.315354	1.181634
C	1.656332	3.872006	1.245796
H	2.161766	1.792251	0.793455
C	0.617392	4.748692	1.518582
C	-1.855701	5.093973	1.800571
C	-3.332758	3.156946	1.483025
H	2.697856	4.206798	1.236347
H	0.814803	5.805578	1.730058

C	-3.121282	4.523484	1.784291
H	-1.711989	6.155657	2.026256
H	-4.336561	2.733738	1.467624
H	-3.995545	5.147113	2.003118
N	0.115251	2.044557	0.985655
C	-3.435179	0.255425	0.765993
O	-4.572330	0.669370	1.042207
O	1.217965	-0.678504	1.024358
C	2.426083	-0.630259	0.522030
C	2.668466	-0.290806	-0.857582
C	3.565598	-0.939212	1.349097
C	3.982095	-0.299497	-1.390480
C	1.574469	0.084444	-1.731969
C	4.831662	-0.920326	0.763652
C	5.081816	-0.613758	-0.599745
H	4.097551	-0.044190	-2.445178
N	0.310599	0.216989	-1.423562
O	1.875796	0.362072	-3.030596
H	5.691654	-1.156545	1.395834
C	-0.432336	0.723465	-2.596774
C	0.625260	0.601941	-3.725825

H	-1.293473	0.060450	-2.785992
C	-0.941840	2.127151	-2.319220
H	0.435872	-0.259128	-4.389150
C	-0.034914	3.192667	-2.159990
C	-2.315055	2.366008	-2.158721
C	-0.498781	4.476012	-1.852221
H	1.042062	3.012782	-2.257418
C	-2.780874	3.650614	-1.842948
H	-3.024104	1.536791	-2.257594
C	-1.874278	4.707562	-1.691567
H	0.215087	5.295434	-1.720522
H	-3.852286	3.819400	-1.699246
H	-2.235818	5.707173	-1.431734
C	-3.224012	-1.197642	0.361572
Co	-0.470414	0.067964	0.420855
C	3.366839	-1.262103	2.841739
C	6.524268	-0.634186	-1.131249
C	7.120387	-2.050629	-0.940230
H	8.162966	-2.083634	-1.305918
H	7.125388	-2.347299	0.122875
H	6.532012	-2.799525	-1.498627

C	7.376184	0.392038	-0.344255
H	7.389466	0.163924	0.735605
H	8.421118	0.384149	-0.704547
H	6.972796	1.412045	-0.470904
C	6.588915	-0.275693	-2.628219
H	6.012021	-0.990031	-3.241292
H	6.197090	0.738633	-2.819824
H	7.637149	-0.303329	-2.974180
C	2.464717	-2.512752	2.997876
H	1.477020	-2.336061	2.547710
H	2.927858	-3.387346	2.506823
H	2.332319	-2.749911	4.069346
C	4.705843	-1.554736	3.548423
H	5.219902	-2.429160	3.111103
H	5.392216	-0.690327	3.505614
H	4.513637	-1.776852	4.612591
C	2.711331	-0.048698	3.549946
H	3.359769	0.842259	3.469769
H	1.733327	0.186443	3.104607
H	2.566249	-0.271074	4.622912
H	0.737690	1.517141	-4.326114

C	0.134433	-3.130350	-0.868604
H	0.858992	-2.359482	-0.579084
H	0.404757	-3.531092	-1.860688
C	-5.748254	-1.508418	-0.586865
H	-5.957436	-0.479528	-0.263698
H	-6.255693	-1.710163	-1.546578
C	-0.123358	-4.212080	0.227388
H	0.431957	-4.041111	1.163131
H	0.031495	-5.258054	-0.090057
C	-5.976737	-2.599324	0.513485
H	-6.252096	-2.182729	1.496602
H	-6.689725	-3.403712	0.260509
C	-1.606174	-3.790352	0.309754
C	-2.578477	-4.461670	-0.693723
C	-2.016945	-2.782953	1.390931
C	-4.078846	-4.068158	-0.626905
H	-2.367408	-5.523084	-0.910062
C	-3.498782	-2.356128	1.433489
H	-1.480888	-2.836790	2.351736
C	-4.482144	-3.000528	0.422442
H	-4.786851	-4.897180	-0.797034

H	-3.907153	-2.114140	2.428531

Int1-*ent2*

Lowest frequency = 7.8733 cm^{-1}

Charge = 0, Multiplicity = 1

104

C	-1.508094	-2.538773	-0.024927
C	0.719603	-2.601999	1.404490
C	-1.789715	-4.000141	0.483971
C	0.413238	-4.027546	1.948325
C	-0.875390	-4.749085	1.479992
H	0.704422	-4.217519	2.995359
H	-1.380197	-5.363689	2.244625
N	-2.457042	-0.460783	0.611941
C	-2.921967	1.794762	1.148179
C	-0.998171	3.094546	1.331184
C	-3.780702	2.912230	1.389899
C	-3.440286	0.496497	0.845419
C	-1.765167	4.259322	1.564340

H	0.094000	3.118796	1.293114
C	-3.148959	4.168038	1.604701
C	-5.187207	2.698357	1.375944
C	-4.831478	0.325081	0.837767
H	-1.255124	5.215692	1.713037
H	-3.764885	5.055358	1.787645
C	-5.679331	1.424917	1.115201
H	-5.862529	3.539713	1.560636
H	-5.249346	-0.650498	0.589426
H	-6.762432	1.260158	1.102695
N	-1.557371	1.901003	1.146422
C	-2.703415	-1.631268	-0.080834
O	-3.758742	-1.899385	-0.674175
O	1.005479	0.839013	1.217848
C	2.096505	0.972553	0.502183
C	2.087561	0.766633	-0.918810
C	3.349027	1.345616	1.118203
C	3.287567	0.759048	-1.673611
C	0.835119	0.606987	-1.612343
C	4.494986	1.289772	0.322566
C	4.515316	0.969735	-1.060884

H	3.206957	0.570784	-2.745591
N	-0.356451	0.415406	-1.097097
O	0.851671	0.671296	-2.968360
H	5.452767	1.516783	0.796202
C	-1.374501	0.381446	-2.174026
C	-0.478321	0.330931	-3.438988
H	-1.968420	-0.540703	-2.093000
C	-2.311031	1.571444	-2.100583
H	-0.434418	-0.679570	-3.879658
C	-1.800576	2.878065	-1.995214
C	-3.700876	1.372724	-2.140231
C	-2.670964	3.972804	-1.932812
H	-0.717542	3.036721	-1.943209
C	-4.572148	2.469935	-2.081976
H	-4.096513	0.352061	-2.178133
C	-4.059263	3.769754	-1.978469
H	-2.266412	4.986063	-1.839954
H	-5.653935	2.304967	-2.097119
H	-4.740419	4.625394	-1.920882
C	-0.195217	-1.805266	0.416778
Co	-0.654147	0.106929	0.697534

C	3.428113	1.864000	2.573110
C	5.858032	0.889831	-1.803908
C	6.723109	-0.220367	-1.157392
H	7.699209	-0.299448	-1.670130
H	6.916092	-0.010969	-0.090850
H	6.213845	-1.197800	-1.223308
C	6.596868	2.246316	-1.698508
H	6.792262	2.521877	-0.647656
H	7.569993	2.197241	-2.220283
H	5.997702	3.053172	-2.155655
C	5.665367	0.553523	-3.294884
H	5.160469	-0.419289	-3.427972
H	5.068822	1.326021	-3.811119
H	6.647909	0.495532	-3.795348
C	2.932411	0.834528	3.615450
H	1.882805	0.560526	3.433826
H	3.547853	-0.080208	3.593185
H	3.008291	1.273805	4.626936
C	4.870947	2.252710	2.959885
H	5.554077	1.385741	2.925937
H	5.277407	3.042925	2.304536

H	4.873993	2.639682	3.993739
C	2.552888	3.136092	2.687220
H	2.851410	3.891742	1.939323
H	1.494061	2.881243	2.536376
H	2.657539	3.580934	3.693451
H	-0.765142	1.065321	-4.205670
C	-3.083658	-4.734012	0.039943
H	-3.925277	-4.069608	-0.203134
H	-3.415833	-5.496082	0.766221
C	2.069487	-2.024400	1.897474
H	2.069491	-0.941613	2.037157
H	2.420762	-2.502238	2.828444
C	-2.315970	-5.299021	-1.203902
H	-2.693620	-4.925555	-2.171194
H	-2.238224	-6.398968	-1.262072
C	2.778910	-2.532305	0.602986
H	3.099590	-1.722506	-0.073184
H	3.629350	-3.220983	0.752104
C	-1.028734	-4.582571	-0.722950
C	-0.089219	-5.360985	0.240744
C	-0.747553	-3.155589	-1.273245

C	1.200911	-4.636324	0.713503
H	0.014758	-6.445492	0.064566
C	0.563497	-2.458000	-0.834098
H	-1.076094	-2.959699	-2.309393
C	1.459518	-3.209588	0.167339
H	2.091998	-5.276798	0.831676
H	1.092865	-1.888634	-1.615702

Int1-*ent2*

Lowest frequency = 17.3133 cm^{-1}

Charge = 0, Multiplicity = 3

104

C	-3.107091	-1.194458	0.255594
C	-1.078235	-2.746829	-0.465597
C	-3.776925	-2.467988	0.865474
C	-1.712750	-4.007788	0.177464
C	-3.098690	-3.864491	0.864557
H	-1.016533	-4.712919	0.661339
H	-3.237781	-4.473350	1.773910

N	-2.175486	0.923736	0.879308
C	-0.834457	2.825187	1.304137
C	1.412615	2.511744	0.783700
C	-0.603199	4.165401	1.754137
C	-2.140913	2.235352	1.346696
C	1.737158	3.818271	1.216965
H	2.189591	1.843400	0.403777
C	0.733032	4.645378	1.693525
C	-1.708706	4.927941	2.223906
C	-3.201414	3.015747	1.831524
H	2.777449	4.153194	1.168092
H	0.953369	5.665004	2.028550
C	-2.969205	4.347975	2.253331
H	-1.547939	5.957173	2.560703
H	-4.197855	2.578268	1.873130
H	-3.822133	4.930758	2.618694
N	0.169319	2.033907	0.815642
C	-3.333075	0.159379	0.870243
O	-4.443875	0.548440	1.246975
O	0.914443	-0.766328	0.836144
C	2.177286	-0.713786	0.511795

C	2.637046	-0.199806	-0.757338
C	3.167705	-1.225128	1.428705
C	4.017679	-0.200258	-1.086878
C	1.700270	0.331665	-1.723950
C	4.505968	-1.186868	1.044495
C	4.971924	-0.684766	-0.201552
H	4.300238	0.197875	-2.062881
N	0.400901	0.435271	-1.598943
O	2.197131	0.786325	-2.907396
H	5.251761	-1.567563	1.747289
C	-0.155515	1.107946	-2.793277
C	1.069979	1.168588	-3.737797
H	-0.948210	0.471702	-3.219265
C	-0.771770	2.430482	-2.367129
H	0.997564	0.440979	-4.563468
C	0.023505	3.563995	-2.118175
C	-2.149677	2.489860	-2.098257
C	-0.553735	4.738366	-1.620251
H	1.105365	3.523905	-2.288437
C	-2.727602	3.661891	-1.590716
H	-2.772349	1.605269	-2.270833

C	-1.930787	4.789770	-1.354159
H	0.076144	5.611642	-1.422635
H	-3.798408	3.687467	-1.367427
H	-2.377763	5.701740	-0.946893
C	-1.713745	-1.340210	-0.422813
Co	-0.582080	0.187699	0.101711
C	2.726962	-1.782099	2.794535
C	6.477740	-0.699581	-0.509732
C	6.996947	-2.157769	-0.459356
H	8.081961	-2.188587	-0.668000
H	6.830377	-2.612801	0.532285
H	6.480409	-2.781297	-1.209814
C	7.225601	0.148911	0.548141
H	7.062868	-0.240364	1.568028
H	8.313330	0.140265	0.351918
H	6.876775	1.196316	0.525027
C	6.783609	-0.120968	-1.904331
H	6.290114	-0.703323	-2.701975
H	6.453464	0.929297	-1.988996
H	7.871716	-0.148593	-2.090027
C	1.774147	-2.988495	2.587207

H	0.875725	-2.688279	2.028480
H	2.287005	-3.792171	2.028806
H	1.465715	-3.394545	3.567970
C	3.929891	-2.269403	3.627447
H	4.477648	-3.084849	3.122195
H	4.641786	-1.452166	3.841269
H	3.567504	-2.658754	4.594773
C	2.009430	-0.670789	3.603702
H	2.692035	0.180372	3.778097
H	1.119751	-0.304291	3.070882
H	1.695219	-1.065554	4.587142
H	1.269290	2.170250	-4.147523
C	-5.161748	-2.366782	1.559052
H	-5.376127	-1.389812	2.014782
H	-5.326593	-3.164932	2.303498
C	0.283747	-2.974049	-1.174015
H	0.960521	-2.113874	-1.146859
H	0.826737	-3.853084	-0.787599
C	-5.853442	-2.610298	0.173539
H	-6.417612	-1.738732	-0.199266
H	-6.501648	-3.501739	0.106008

C	-0.445512	-3.197344	-2.536089
H	-0.243982	-2.415142	-3.289275
H	-0.292644	-4.182060	-3.011464
C	-4.459160	-2.766850	-0.486924
C	-3.818522	-4.179764	-0.515917
C	-3.820549	-1.503682	-1.129278
C	-2.431295	-4.310070	-1.204045
H	-4.500738	-5.034335	-0.664273
C	-2.449893	-1.631652	-1.830986
H	-4.520075	-0.773541	-1.570140
C	-1.804023	-3.028289	-1.821669
H	-2.264785	-5.241783	-1.771729
H	-2.320463	-1.033728	-2.749713

Int1-*ent2*

Lowest frequency = 13.9836 cm^{-1}

Charge = 0, Multiplicity = 5

104

C	-3.224747	-1.201534	0.198160

C	-1.238234	-2.902854	-0.152271
C	-4.035325	-2.377657	0.828890
C	-2.012588	-4.079243	0.478735
C	-3.450861	-3.806395	0.994805
H	-1.421036	-4.799441	1.066614
H	-3.730273	-4.343424	1.916399
N	-2.269748	0.930972	0.831678
C	-0.949644	2.849018	1.292119
C	1.328308	2.551835	0.899888
C	-0.755407	4.206332	1.713757
C	-2.257837	2.246803	1.267717
C	1.617460	3.874239	1.308263
H	2.127030	1.878509	0.573438
C	0.577927	4.698774	1.709646
C	-1.888984	4.973255	2.101062
C	-3.344787	3.041419	1.669594
H	2.653898	4.224615	1.301374
H	0.768397	5.730521	2.025906
C	-3.145393	4.383858	2.073068
H	-1.753482	6.011623	2.420863
H	-4.341989	2.602787	1.653339

H	-4.021419	4.969553	2.374313
N	0.089636	2.064859	0.890015
C	-3.435019	0.222833	0.699001
O	-4.588611	0.644463	0.884514
O	1.122262	-0.652029	0.914901
C	2.372875	-0.642416	0.530802
C	2.770418	-0.199144	-0.781428
C	3.402475	-1.103365	1.427815
C	4.132219	-0.227401	-1.177540
C	1.789594	0.301275	-1.724785
C	4.721419	-1.102570	0.975521
C	5.127165	-0.676224	-0.317037
H	4.368573	0.118624	-2.185150
N	0.497515	0.407817	-1.555617
O	2.246996	0.731591	-2.934044
H	5.498158	-1.451000	1.661353
C	-0.102736	1.045669	-2.747259
C	1.096829	1.118843	-3.727330
H	-0.888625	0.379487	-3.140523
C	-0.748659	2.362648	-2.351527
H	1.003283	0.402902	-4.560963

C	0.027399	3.507724	-2.093648
C	-2.136804	2.416813	-2.145171
C	-0.579752	4.688522	-1.650540
H	1.116043	3.471636	-2.215663
C	-2.745525	3.595312	-1.691388
H	-2.744621	1.522801	-2.322377
C	-1.968076	4.735032	-1.448295
H	0.034289	5.571164	-1.444939
H	-3.824601	3.614703	-1.512427
H	-2.438970	5.652431	-1.082406
C	-1.817528	-1.500513	-0.309793
Co	-0.490026	0.108376	0.184454
C	3.029347	-1.565039	2.848586
C	6.615123	-0.724208	-0.700980
C	7.124077	-2.183247	-0.599578
H	8.196523	-2.237456	-0.861797
H	7.005264	-2.583049	0.422315
H	6.563973	-2.839905	-1.288035
C	7.424781	0.170699	0.269496
H	7.313855	-0.163033	1.315719
H	8.500458	0.140524	0.016842

H	7.083137	1.218958	0.209645
C	6.852846	-0.223902	-2.138572
H	6.313232	-0.842059	-2.877263
H	6.527894	0.824052	-2.262315
H	7.929521	-0.273942	-2.378491
C	2.050718	-2.765619	2.774956
H	1.124733	-2.486076	2.251657
H	2.518536	-3.611616	2.239932
H	1.794832	-3.103779	3.795816
C	4.268628	-2.017049	3.647389
H	4.777804	-2.872523	3.168631
H	5.001587	-1.199658	3.769027
H	3.953582	-2.336448	4.656096
C	2.371196	-0.390327	3.617456
H	3.076060	0.456084	3.703708
H	1.463842	-0.040785	3.103154
H	2.097197	-0.714141	4.638206
H	1.280164	2.127043	-4.129313
C	-5.476362	-2.161790	1.359011
H	-5.676145	-1.146310	1.727927
H	-5.766281	-2.901662	2.125179

C	0.176018	-3.220672	-0.706434
H	0.858817	-2.363559	-0.661256
H	0.655961	-4.081802	-0.212266
C	-6.031395	-2.455351	-0.075700
H	-6.490583	-1.576777	-0.558314
H	-6.726120	-3.309118	-0.163408
C	-0.410750	-3.517863	-2.121284
H	-0.087053	-2.809748	-2.902946
H	-0.257611	-4.545466	-2.494190
C	-4.590993	-2.736742	-0.575055
C	-4.035336	-4.176578	-0.436603
C	-3.819410	-1.569693	-1.241025
C	-2.593971	-4.429670	-0.954524
H	-4.743608	-5.005894	-0.603970
C	-2.407377	-1.837902	-1.786124
H	-4.426170	-0.834362	-1.795495
C	-1.835456	-3.241851	-1.600462
H	-2.416570	-5.408547	-1.432090
H	-2.114978	-1.304828	-2.705929

Int2-*ent1*

Lowest frequency = 9.5827 cm^{-1}

Charge = 0, Multiplicity = 1

123

C	0.898889	-0.744245	-1.676533
C	2.519947	-2.901804	-1.819819
C	-0.097816	-1.581983	-2.522906
C	1.474907	-3.735039	-2.609858
C	0.126442	-3.054445	-2.974151
H	1.412069	-4.807105	-2.353313
H	-0.758448	-3.714530	-2.957996
N	1.928144	-0.413772	0.803900
C	1.060722	0.518179	2.813970
C	-1.186369	1.149865	2.675922
C	1.138531	0.882190	4.198636
C	2.185676	-0.038134	2.113892
C	-1.213524	1.491187	4.049885
H	-2.070977	1.263753	2.041647
C	-0.054308	1.371031	4.801345
C	2.379672	0.729629	4.873663

C	3.396073	-0.160173	2.817987
H	-2.143892	1.857999	4.493365
H	-0.042662	1.647978	5.861481
C	3.472438	0.229766	4.175024
H	2.458682	1.010961	5.928721
H	4.261692	-0.561482	2.293642
H	4.433567	0.119542	4.689570
N	-0.085417	0.689521	2.088539
C	2.854048	-1.026352	-0.004326
O	4.036170	-1.252885	0.289841
O	-1.375952	1.014880	-0.485417
C	-2.614902	0.626308	-0.302752
C	-2.952962	-0.696942	0.140057
C	-3.706903	1.526036	-0.598219
C	-4.301553	-1.095341	0.324664
C	-1.918687	-1.683990	0.338358
C	-5.009267	1.077172	-0.380279
C	-5.351482	-0.218229	0.088447
H	-4.480024	-2.118909	0.658047
N	-0.617604	-1.515381	0.356180
O	-2.318691	-2.967055	0.533419

H	-5.827992	1.769005	-0.593870
C	0.042262	-2.813164	0.646635
C	-1.135123	-3.801364	0.468165
H	0.820969	-2.998458	-0.107384
C	0.664416	-2.856286	2.028409
H	-1.122948	-4.294042	-0.519128
C	-0.055380	-2.418857	3.154164
C	1.961123	-3.368139	2.197516
C	0.522246	-2.477144	4.428958
H	-1.062525	-2.006203	3.030262
C	2.540234	-3.423443	3.471620
H	2.533431	-3.690779	1.321575
C	1.822580	-2.975941	4.590388
H	-0.039361	-2.115061	5.296299
H	3.561827	-3.799674	3.587007
H	2.280997	-3.001692	5.584245
C	2.265661	-1.427393	-1.333812
C	-3.434328	2.938426	-1.148691
C	-6.828790	-0.590170	0.292420
C	-7.585385	-0.451233	-1.051610
H	-8.653282	-0.705952	-0.922580

H	-7.529092	0.579183	-1.443420
H	-7.155765	-1.126953	-1.811651
C	-7.457751	0.364707	1.336671
H	-7.392879	1.417691	1.011652
H	-8.525442	0.122751	1.489891
H	-6.938777	0.276563	2.307308
C	-6.989686	-2.037555	0.794985
H	-6.578359	-2.765051	0.073553
H	-6.482523	-2.188525	1.764054
H	-8.060480	-2.268573	0.934231
C	-2.633090	2.839774	-2.471480
H	-1.682976	2.310251	-2.311790
H	-3.213680	2.290443	-3.233254
H	-2.417955	3.851026	-2.862046
C	-4.737228	3.706257	-1.451811
H	-5.354009	3.187494	-2.206765
H	-5.349434	3.855877	-0.544879
H	-4.484329	4.703777	-1.851811
C	-2.641014	3.755266	-0.101288
H	-3.233350	3.877332	0.822977
H	-1.702876	3.241789	0.147500

H	-2.398860	4.759604	-0.493908
H	-1.201096	-4.552628	1.268004
C	-1.453967	-0.968066	-2.969325
H	-1.848536	-0.207076	-2.291148
H	-2.237549	-1.723817	-3.150110
C	3.853293	-3.623145	-1.482433
H	4.329151	-3.297048	-0.546873
H	3.754905	-4.722991	-1.488998
C	-0.781574	-0.392683	-4.255128
H	-0.739601	0.709871	-4.284746
H	-1.191029	-0.748682	-5.216720
C	4.488447	-3.015391	-2.779579
H	5.281871	-2.277860	-2.573422
H	4.865764	-3.741005	-3.521430
C	0.549827	-1.053547	-3.836249
C	0.767395	-2.513938	-4.318035
C	1.563011	-0.200721	-3.058401
C	2.114299	-3.199639	-3.960228
H	0.358362	-2.755890	-5.314018
C	2.892707	-0.893683	-2.682276
H	1.631346	0.857417	-3.361023

C	3.126757	-2.363555	-3.133079
H	2.530744	-3.862698	-4.737714
H	3.788381	-0.249989	-2.660892
Co	0.262314	0.132648	0.057254
C	2.372863	2.929854	0.809246
C	2.707811	2.400251	-1.250239
H	2.556116	2.834402	1.886290
H	3.209239	1.833346	-2.042103
O	3.230051	2.059146	0.047460
C	2.689981	4.277172	0.127738
C	2.907890	3.929340	-1.223660
C	2.763632	5.596115	0.554219
C	3.206264	4.893017	-2.177047
C	3.081975	6.582701	-0.408447
H	2.592554	5.872539	1.600163
C	3.298985	6.237904	-1.747867
H	3.374499	4.630486	-3.227075
H	3.162911	7.630596	-0.100435
H	3.547169	7.019753	-2.473547
C	1.191440	2.203828	-1.040002
H	0.442684	2.141104	-1.828978

C 0.982204 2.555753 0.263565

H 0.039462 2.833423 0.730810

Int2-*ent1*

Lowest frequency = 13.1701 cm^{-1}

Charge = 0, Multiplicity = 3

123

C 0.050473 -0.505612 1.802217

C 0.281809 -3.075895 2.501017

C 1.548401 -0.356388 2.142791

C 1.810709 -2.929315 2.743974

C 2.465431 -1.528185 2.570148

H 2.436250 -3.781081 2.424499

H 3.490375 -1.531717 2.163292

N -1.985678 -1.166076 0.225658

C -2.716266 -0.428208 -1.889651

C -1.121530 0.865606 -2.993541

C -3.618853 -0.376043 -2.998283

C -3.016589 -1.175940 -0.706919

C	-1.946314	0.971031	-4.136998
H	-0.129852	1.324503	-2.946367
C	-3.192893	0.361055	-4.137172
C	-4.859914	-1.064165	-2.889165
C	-4.243468	-1.844955	-0.639832
H	-1.588273	1.530923	-5.006051
H	-3.850947	0.428896	-5.010513
C	-5.147126	-1.771049	-1.728273
H	-5.570148	-1.029267	-3.721489
H	-4.482359	-2.431147	0.246790
H	-6.103359	-2.299500	-1.645287
N	-1.505211	0.205136	-1.904177
C	-1.837807	-2.137403	1.184813
O	-2.612194	-3.087114	1.376595
O	0.851574	1.259918	-0.724429
C	2.150476	1.128579	-0.887717
C	2.802793	-0.150695	-0.890235
C	2.980348	2.298174	-1.066598
C	4.212189	-0.257727	-0.962387
C	2.032716	-1.379363	-0.878206
C	4.363938	2.122997	-1.128579

C	5.020818	0.869694	-1.054514
H	4.638374	-1.261911	-0.944524
N	0.753357	-1.513646	-0.684563
O	2.712257	-2.529888	-1.151913
H	4.988249	3.013564	-1.233099
C	0.378422	-2.920514	-0.933737
C	1.759791	-3.619562	-1.065207
H	-0.160963	-3.318469	-0.061652
C	-0.525283	-3.035431	-2.147690
H	2.010064	-4.223478	-0.177029
C	-0.164043	-2.431216	-3.365853
C	-1.743781	-3.728475	-2.058997
C	-1.007530	-2.522926	-4.479402
H	0.772529	-1.866400	-3.434542
C	-2.588316	-3.822596	-3.174518
H	-2.049613	-4.161208	-1.100351
C	-2.222480	-3.220230	-4.385258
H	-0.723252	-2.039382	-5.419945
H	-3.544460	-4.348097	-3.088269
H	-2.888825	-3.281190	-5.252269
C	-0.622291	-1.887747	2.043195

C	2.367272	3.711374	-1.163116
C	6.555998	0.807761	-1.089303
C	7.122588	1.601387	0.113907
H	8.227734	1.578105	0.107709
H	6.805752	2.658390	0.085844
H	6.770818	1.167215	1.066313
C	7.070253	1.435795	-2.407857
H	6.754367	2.488970	-2.504906
H	8.174715	1.409461	-2.443349
H	6.681061	0.882637	-3.280444
C	7.072238	-0.641363	-1.004461
H	6.748720	-1.132148	-0.069813
H	6.715917	-1.249372	-1.854241
H	8.176271	-0.646780	-1.024480
C	1.650117	4.085456	0.156488
H	0.847243	3.370226	0.382187
H	2.363571	4.091632	0.998506
H	1.204346	5.093472	0.074256
C	3.438525	4.790033	-1.428221
H	4.168893	4.859418	-0.603102
H	3.989558	4.601557	-2.366454

H	2.944921	5.773454	-1.518855
C	1.359264	3.764173	-2.336160
H	1.858345	3.521757	-3.291068
H	0.546907	3.042194	-2.173609
H	0.923021	4.776211	-2.420509
H	1.857450	-4.230756	-1.975310
C	2.215117	1.047375	2.052415
H	1.702423	1.710443	1.349705
H	3.285889	1.022245	1.791179
C	-0.315132	-4.494369	2.707577
H	-1.172188	-4.735978	2.062485
H	0.445232	-5.291307	2.628823
C	1.877667	1.345980	3.542312
H	1.197268	2.201264	3.700185
H	2.745897	1.467659	4.212690
C	-0.695507	-4.099823	4.175820
H	-1.783633	-4.024014	4.337272
H	-0.262531	-4.725381	4.976115
C	1.200160	-0.033256	3.637633
C	2.129434	-1.197682	4.084959
C	-0.304714	-0.124016	3.356685

C	1.503676	-2.603652	4.267473
H	2.905276	-0.917583	4.817991
C	-0.920181	-1.527193	3.550455
H	-0.921548	0.729939	3.685746
C	-0.013773	-2.721300	3.975740
H	1.903626	-3.195998	5.108114
H	-1.955625	-1.550382	3.928560
Co	-0.510466	0.036657	-0.084019
C	-3.328066	2.492889	-0.270800
C	-2.912574	1.746743	1.706112
H	-3.762327	2.413977	-1.275951
H	-2.971583	0.986651	2.494305
O	-3.645570	1.336592	0.531621
C	-3.845240	3.606427	0.661863
C	-3.574032	3.114675	1.958408
C	-4.433366	4.848962	0.470755
C	-3.887211	3.855241	3.089411
C	-4.765492	5.605925	1.619540
H	-4.645985	5.236419	-0.531593
C	-4.497688	5.118646	2.903765
H	-3.680058	3.479897	4.097507

H	-5.242650	6.584697	1.501287
H	-4.767458	5.721098	3.777774
C	-1.526885	2.059028	1.100645
H	-0.599884	2.141758	1.667730
C	-1.795952	2.534134	-0.139925
H	-1.108736	2.999146	-0.845940

Int2-*ent1*

Lowest frequency = 11.0415 cm^{-1}

Charge = 0, Multiplicity = 5

123

C	-0.021822	-0.518101	1.996044
C	0.161490	-3.121268	2.370521
C	1.492497	-0.428338	2.135158
C	1.705953	-3.042420	2.517599
C	2.392060	-1.652972	2.405959
H	2.281945	-3.884615	2.097917
H	3.387660	-1.646594	1.934181
N	-2.058427	-1.146415	0.197779

C	-2.862268	-0.346193	-1.880389
C	-1.361488	1.044435	-3.004970
C	-3.816570	-0.270075	-2.946804
C	-3.104797	-1.144443	-0.707703
C	-2.229436	1.156844	-4.114308
H	-0.390106	1.548829	-2.974714
C	-3.457278	0.511118	-4.079143
C	-5.043141	-0.979112	-2.816533
C	-4.331229	-1.819876	-0.620616
H	-1.926377	1.756526	-4.977801
H	-4.156351	0.588729	-4.919340
C	-5.278804	-1.722229	-1.667093
H	-5.780068	-0.933194	-3.624939
H	-4.526009	-2.435677	0.257022
H	-6.224462	-2.266410	-1.564131
N	-1.677205	0.327138	-1.928512
C	-1.987157	-2.029749	1.237130
O	-2.821181	-2.901420	1.540104
O	0.946751	1.305931	-0.826350
C	2.237770	1.125752	-0.950154
C	2.836202	-0.183361	-0.946727

C	3.126725	2.258082	-1.091086
C	4.243127	-0.351357	-0.988668
C	2.013652	-1.373770	-0.950990
C	4.501254	2.023673	-1.115638
C	5.101404	0.739643	-1.045541
H	4.625266	-1.373381	-0.972307
N	0.725290	-1.461335	-0.750304
O	2.639116	-2.550845	-1.230666
H	5.166839	2.887498	-1.189323
C	0.290288	-2.848194	-1.011306
C	1.639347	-3.599561	-1.186774
H	-0.247410	-3.231577	-0.131414
C	-0.639292	-2.917233	-2.208683
H	1.870525	-4.258999	-0.334244
C	-0.314825	-2.251427	-3.404593
C	-1.841545	-3.638734	-2.128920
C	-1.179724	-2.308957	-4.504037
H	0.606670	-1.661576	-3.463869
C	-2.707359	-3.699463	-3.229834
H	-2.118620	-4.122882	-1.186055
C	-2.378656	-3.033996	-4.418116

H	-0.925528	-1.775200	-5.425728
H	-3.651997	-4.245946	-3.148350
H	-3.062859	-3.066628	-5.272539
C	-0.730067	-1.866037	2.082048
C	2.569146	3.692506	-1.186642
C	6.633001	0.612162	-1.043994
C	7.203668	1.361984	0.185088
H	8.306746	1.292246	0.204781
H	6.932157	2.431760	0.167429
H	6.811134	0.928159	1.121713
C	7.206750	1.237166	-2.339261
H	6.937765	2.304051	-2.427426
H	8.309739	1.164694	-2.348148
H	6.816593	0.714066	-3.229742
C	7.084686	-0.858923	-0.970351
H	6.718783	-1.349060	-0.051013
H	6.722188	-1.438306	-1.837343
H	8.187655	-0.912076	-0.965454
C	1.845751	4.072816	0.127788
H	1.022901	3.374663	0.334795
H	2.549677	4.049644	0.977897

H	1.427352	5.093237	0.053718
C	3.682975	4.733500	-1.422559
H	4.399631	4.769728	-0.583218
H	4.244845	4.531093	-2.351484
H	3.228198	5.735428	-1.514634
C	1.582245	3.789483	-2.375815
H	2.094280	3.554930	-3.325795
H	0.753159	3.079954	-2.241089
H	1.169986	4.812542	-2.448108
H	1.701966	-4.167889	-2.127469
C	2.199805	0.954658	2.101040
H	1.694504	1.673695	1.445761
H	3.257845	0.896757	1.798361
C	-0.472837	-4.531636	2.493285
H	-1.389750	-4.674478	1.903259
H	0.240537	-5.346328	2.276625
C	1.951392	1.168656	3.624225
H	1.285092	2.013702	3.868586
H	2.860244	1.252044	4.244743
C	-0.723468	-4.265653	4.016559
H	-1.791903	-4.185098	4.274614

H	-0.240320	-4.968389	4.717713
C	1.260001	-0.205792	3.702045
C	2.177853	-1.428351	3.961928
C	-0.265117	-0.252080	3.558702
C	1.512102	-2.824578	4.079481
H	3.022433	-1.236676	4.645692
C	-0.924861	-1.644852	3.645523
H	-0.827526	0.599071	3.980434
C	-0.025089	-2.886691	3.890004
H	1.951867	-3.490576	4.841225
H	-1.936188	-1.682050	4.082845
Co	-0.489060	0.093396	-0.205928
C	-3.194273	2.618078	-0.169542
C	-2.756516	1.792759	1.770200
H	-3.644294	2.582089	-1.170307
H	-2.806471	1.001989	2.528090
O	-3.510353	1.435312	0.592153
C	-3.687267	3.699380	0.812981
C	-3.401844	3.155260	2.085479
C	-4.267282	4.953210	0.679348
C	-3.691874	3.853886	3.248863

C	-4.576472	5.667361	1.861504
H	-4.490863	5.381364	-0.303891
C	-4.294417	5.128333	3.121739
H	-3.473712	3.437598	4.238461
H	-5.047215	6.653654	1.788416
H	-4.547009	5.698127	4.022408
C	-1.377729	2.119052	1.157765
H	-0.443721	2.168360	1.716087
C	-1.659430	2.640026	-0.061909
H	-0.978340	3.121731	-0.763335

Int2-*ent2*

Lowest frequency = 12.6129 cm^{-1}

Charge = 0, Multiplicity = 1

123

C	-1.796467	-1.140001	-1.958724
C	0.174282	-2.696230	-1.110489
C	-2.525773	-2.453117	-2.420548
C	-0.558033	-4.005671	-1.516008

C	-1.949058	-3.878203	-2.193865
H	-0.434040	-4.869320	-0.840235
H	-2.680587	-4.659907	-1.925319
N	-2.114582	0.702200	-0.457408
C	-1.928572	2.445397	1.123165
C	0.198854	2.571559	2.080308
C	-2.389509	3.599114	1.832838
C	-2.754126	1.747652	0.181968
C	-0.159199	3.738132	2.796649
H	1.192200	2.121067	2.156493
C	-1.447243	4.240205	2.685371
C	-3.733913	4.016324	1.634815
C	-4.079978	2.185639	0.023633
H	0.584741	4.221620	3.436547
H	-1.750267	5.132910	3.243239
C	-4.545031	3.300430	0.759558
H	-4.111616	4.893430	2.169473
H	-4.724071	1.670783	-0.688011
H	-5.584063	3.616831	0.614348
N	-0.667994	1.949804	1.287999
C	-2.638481	0.036355	-1.531484

O	-3.641792	0.400605	-2.165696
O	1.254994	-0.099618	1.161815
C	2.490759	0.071504	0.770603
C	2.831297	0.677356	-0.483137
C	3.577283	-0.362093	1.621636
C	4.175752	0.741734	-0.932902
C	1.805936	1.284858	-1.293999
C	4.874765	-0.271085	1.118559
C	5.216383	0.248638	-0.158388
H	4.357020	1.195424	-1.908424
N	0.512219	1.296886	-1.100173
O	2.222637	1.953681	-2.404183
H	5.691771	-0.628951	1.749880
C	-0.121694	2.145559	-2.138066
C	1.038676	2.338801	-3.145483
H	-0.955231	1.598366	-2.600261
C	-0.647632	3.458533	-1.587242
H	0.953267	1.677641	-4.025458
C	0.179470	4.291515	-0.812374
C	-1.956371	3.871472	-1.886305
C	-0.298565	5.519914	-0.339873

H	1.197727	3.970527	-0.566382
C	-2.434664	5.101843	-1.414261
H	-2.610879	3.208803	-2.462916
C	-1.607239	5.927682	-0.641512
H	0.349000	6.157473	0.271069
H	-3.462144	5.405906	-1.637129
H	-1.984590	6.884198	-0.264594
C	-0.405922	-1.263384	-1.252087
C	3.313459	-0.899959	3.044231
C	6.684528	0.249675	-0.612888
C	7.200873	-1.209744	-0.658268
H	8.259494	-1.237227	-0.975447
H	7.131111	-1.693093	0.331758
H	6.607957	-1.809830	-1.370650
C	7.538761	1.066907	0.386965
H	7.484365	0.645824	1.405895
H	8.600200	1.067200	0.078490
H	7.189945	2.113443	0.432955
C	6.847711	0.869169	-2.013951
H	6.274401	0.309052	-2.773273
H	6.511329	1.920590	-2.033914

H	7.911126	0.848962	-2.310673
C	2.478353	-2.202462	3.001996
H	1.497913	-2.021496	2.540203
H	3.002223	-2.985033	2.426805
H	2.314878	-2.578290	4.028465
C	4.627902	-1.214082	3.789056
H	5.205641	-2.011011	3.288565
H	5.272695	-0.322241	3.881323
H	4.390449	-1.565296	4.808388
C	2.553093	0.166191	3.871137
H	3.130628	1.106589	3.922442
H	1.572858	0.377341	3.419739
H	2.392722	-0.196633	4.902607
H	1.163680	3.381927	-3.469810
C	-3.917395	-2.391159	-3.104849
H	-4.551499	-1.549904	-2.794285
H	-4.477841	-3.336563	-3.000502
C	1.573486	-2.907778	-0.469005
H	1.853366	-2.154735	0.275897
H	1.693495	-3.908186	-0.018151
C	-3.206236	-2.208526	-4.489212

H	-3.357436	-1.207940	-4.927966
H	-3.431398	-2.968057	-5.258567
C	2.247949	-2.736804	-1.866798
H	2.870533	-1.830775	-1.952329
H	2.834021	-3.601079	-2.224563
C	-1.828349	-2.362597	-3.794148
C	-1.252385	-3.791848	-3.617177
C	-1.085095	-1.065450	-3.373999
C	0.140188	-3.908924	-2.935999
H	-1.444286	-4.513678	-4.429293
C	0.307061	-1.173328	-2.715119
H	-1.239889	-0.190763	-4.029440
C	0.848259	-2.593331	-2.505316
H	0.799336	-4.700410	-3.331713
H	1.050392	-0.420161	-3.017774
Co	-0.414819	0.205682	0.234742
C	-2.301852	-2.305518	1.016254
C	-2.759817	-0.627094	2.289797
H	-2.457868	-2.921992	0.124657
H	-3.329056	0.274848	2.546814
O	-3.244332	-1.220630	1.071798

C	-0.951923	-1.588055	1.196677
H	-0.018933	-2.148013	1.226566
C	-1.244165	-0.509235	2.034934
H	-0.566632	-0.116442	2.794789
C	-2.847107	-1.844129	3.231409
C	-3.094762	-2.018193	4.587000
C	-2.543975	-2.943492	2.395212
C	-3.054718	-3.334754	5.101622
H	-3.323293	-1.170858	5.242489
C	-2.481541	-4.236307	2.898291
C	-2.753433	-4.423230	4.273327
H	-3.264492	-3.505791	6.162949
H	-2.238581	-5.089897	2.256153
H	-2.730382	-5.433381	4.696044

Int2-*ent2*

Lowest frequency = 13.9129 cm^{-1}

Charge = 0, Multiplicity = 3

123

C	-1.101239	-0.853264	-2.329024
C	0.330536	-2.489829	-0.834155
C	-1.749623	-2.128088	-2.976830
C	-0.332898	-3.758749	-1.439344
C	-1.409514	-3.576280	-2.541947
H	-0.503437	-4.595242	-0.740688
H	-2.251600	-4.288091	-2.506783
N	-1.885072	0.764131	-0.784574
C	-2.288618	2.247827	0.998271
C	-0.435624	2.499252	2.390010
C	-3.067955	3.178895	1.753233
C	-2.792122	1.622533	-0.183155
C	-1.127585	3.434894	3.195509
H	0.598064	2.207632	2.594442
C	-2.438273	3.767402	2.885708
C	-4.393757	3.451979	1.317338
C	-4.100782	1.919276	-0.584608
H	-0.616734	3.882194	4.053310
H	-2.993726	4.487624	3.496488
C	-4.880734	2.820805	0.176435
H	-5.012776	4.157134	1.881155

H	-4.500961	1.459407	-1.487920
H	-5.902896	3.031926	-0.156887
N	-1.006312	1.916128	1.340147
C	-2.010657	0.319736	-2.081775
O	-2.756847	0.813406	-2.939258
O	1.247643	0.202918	1.310082
C	2.537184	0.236905	1.052924
C	3.068411	0.842940	-0.137977
C	3.479495	-0.314875	1.998552
C	4.442091	0.736047	-0.463716
C	2.218609	1.627910	-1.011304
C	4.813643	-0.418922	1.598880
C	5.327010	0.055009	0.364708
H	4.774870	1.198656	-1.394280
N	0.920123	1.713749	-0.977987
O	2.829179	2.367002	-1.979859
H	5.518627	-0.888890	2.288487
C	0.458887	2.639495	-2.027747
C	1.794642	3.117400	-2.669177
H	-0.143688	2.070017	-2.756111
C	-0.410887	3.740995	-1.453037

H	1.867366	2.891471	-3.744518
C	0.054029	4.521847	-0.378566
C	-1.700494	3.965483	-1.960566
C	-0.758244	5.516715	0.177327
H	1.047630	4.328543	0.042116
C	-2.512729	4.966127	-1.407037
H	-2.087509	3.320258	-2.756813
C	-2.044729	5.741916	-0.338928
H	-0.393584	6.109765	1.022579
H	-3.523648	5.120231	-1.796967
H	-2.685861	6.512260	0.102414
C	-0.013866	-1.026965	-1.223977
C	3.060254	-0.695810	3.437880
C	6.805503	-0.174650	0.015123
C	7.075490	-1.698926	-0.037011
H	8.134784	-1.896550	-0.283408
H	6.856702	-2.180064	0.932094
H	6.443257	-2.179801	-0.803938
C	7.706026	0.471925	1.096009
H	7.511976	0.045078	2.095219
H	8.772577	0.304543	0.859397

H	7.528955	1.560292	1.150894
C	7.170470	0.433871	-1.352346
H	6.568402	-0.008136	-2.165400
H	7.016853	1.527131	-1.364056
H	8.234342	0.240286	-1.575665
C	2.013630	-1.832697	3.490988
H	1.088984	-1.544768	2.971310
H	2.405933	-2.756059	3.033385
H	1.762884	-2.052485	4.544715
C	4.271685	-1.148767	4.280592
H	4.734619	-2.066638	3.877157
H	5.047527	-0.365808	4.344186
H	3.932549	-1.370791	5.307391
C	2.459894	0.560246	4.116920
H	3.174441	1.401818	4.093202
H	1.536210	0.861099	3.602113
H	2.214122	0.341208	5.171965
H	1.984668	4.190411	-2.502310
C	-2.836439	-1.970457	-4.073293
H	-3.422778	-1.043389	-4.003769
H	-3.520269	-2.835138	-4.126158

C	1.424787	-2.784207	0.220337
H	1.507541	-2.021426	1.000039
H	1.309526	-3.771717	0.699212
C	-1.710099	-1.953812	-5.162850
H	-1.616270	-0.990943	-5.693235
H	-1.752703	-2.762528	-5.913426
C	2.529141	-2.713561	-0.881353
H	3.215103	-1.857888	-0.773633
H	3.130440	-3.629503	-1.016571
C	-0.645548	-2.153087	-4.053809
C	-0.277299	-3.608392	-3.656120
C	0.018103	-0.881764	-3.454332
C	0.797214	-3.785381	-2.548184
H	-0.246712	-4.355241	-4.467842
C	1.116478	-1.065086	-2.385006
H	0.154506	-0.030615	-4.143347
C	1.432257	-2.502282	-1.949345
H	1.482076	-4.640870	-2.677707
H	1.971155	-0.374981	-2.443383
Co	-0.230995	0.471933	0.124891
C	-2.639215	-2.046081	0.676716

C	-3.805213	-1.010846	2.160466
H	-2.356137	-2.279588	-0.355268
H	-4.581877	-0.303033	2.478926
O	-3.742122	-1.110818	0.721120
C	-1.603105	-1.323421	1.563711
H	-0.524711	-1.495321	1.553936
C	-2.338192	-0.682884	2.498348
H	-1.993782	-0.132438	3.376011
C	-3.991169	-2.500243	2.515565
C	-4.672602	-3.195295	3.505136
C	-3.226604	-3.176617	1.539124
C	-4.587823	-4.608221	3.498031
H	-5.267315	-2.677989	4.266116
C	-3.130714	-4.560897	1.530737
C	-3.831741	-5.278134	2.529490
H	-5.125617	-5.184856	4.258393
H	-2.540486	-5.089711	0.774595
H	-3.785008	-6.372437	2.541693

Int2-*ent2*

Lowest frequency = 12.5943 cm^{-1}

Charge = 0, Multiplicity = 5

123

C	-2.578457	0.258545	-1.774884
C	-1.405722	-2.092059	-1.963904
C	-3.883291	-0.433214	-2.271383
C	-2.690194	-2.806086	-2.444109
C	-3.973892	-1.949345	-2.585405
H	-2.838028	-3.843510	-2.098648
H	-4.912493	-2.448306	-2.292947
N	-1.670966	1.444012	0.133311
C	-0.425755	2.359950	1.934013
C	1.699724	1.435844	2.196640
C	-0.225739	3.270855	3.023484
C	-1.644630	2.362688	1.164372
C	1.997333	2.315604	3.263672
H	2.430476	0.697370	1.853364
C	1.033684	3.219368	3.682576
C	-1.276351	4.162005	3.375492
C	-2.657325	3.257206	1.556954

H	2.976214	2.259821	3.749066
H	1.229543	3.903989	4.515361
C	-2.462337	4.129446	2.653388
H	-1.135565	4.858677	4.208165
H	-3.580476	3.283896	0.978396
H	-3.275792	4.812213	2.924063
N	0.526591	1.453664	1.568874
C	-2.672425	1.425675	-0.802715
O	-3.546114	2.297479	-0.948446
O	1.374497	-1.109798	0.318574
C	2.675323	-1.081634	0.146656
C	3.308487	-0.166354	-0.767050
C	3.530614	-1.989676	0.874715
C	4.703969	-0.213977	-1.006367
C	2.542199	0.870085	-1.432381
C	4.900311	-1.973219	0.603589
C	5.524219	-1.115665	-0.336817
H	5.113190	0.496505	-1.726352
N	1.286332	1.176181	-1.256968
O	3.214263	1.677229	-2.304106
H	5.538393	-2.669449	1.153853

C	0.994140	2.431376	-1.983596
C	2.229783	2.552754	-2.910617
H	0.067203	2.309728	-2.565845
C	0.812394	3.590395	-1.019189
H	2.027407	2.182642	-3.931428
C	1.858666	3.961545	-0.152195
C	-0.399117	4.296643	-0.974485
C	1.692971	5.023960	0.742292
H	2.802875	3.405616	-0.168218
C	-0.565430	5.362565	-0.078124
H	-1.233853	3.992375	-1.615182
C	0.478002	5.727715	0.781028
H	2.508595	5.297530	1.419590
H	-1.522726	5.890835	-0.038259
H	0.341939	6.550095	1.490700
C	-1.321353	-0.602143	-1.642713
C	2.942033	-2.954424	1.923175
C	7.042561	-1.204011	-0.560851
C	7.405843	-2.629089	-1.046020
H	8.496435	-2.717958	-1.202910
H	7.106411	-3.395104	-0.309820

H	6.897565	-2.857601	-1.999063
C	7.777267	-0.912276	0.770713
H	7.490040	-1.631271	1.557445
H	8.871666	-0.982106	0.631938
H	7.537218	0.102444	1.133860
C	7.525622	-0.191208	-1.616551
H	7.044735	-0.365534	-2.594944
H	7.314292	0.847785	-1.308558
H	8.617016	-0.286836	-1.754475
C	2.004213	-3.967907	1.223692
H	1.210583	-3.446282	0.670775
H	2.570730	-4.586448	0.505654
H	1.538739	-4.638783	1.968704
C	4.037870	-3.760250	2.651044
H	4.607409	-4.405040	1.958719
H	4.750011	-3.101600	3.179159
H	3.564658	-4.415705	3.403027
C	2.162759	-2.156435	2.998622
H	2.843293	-1.475290	3.540315
H	1.361268	-1.559732	2.541464
H	1.714327	-2.848321	3.734403

H	2.654105	3.566294	-2.954833
C	-5.203804	0.360567	-2.436692
H	-5.318223	1.206407	-1.746042
H	-6.095637	-0.287553	-2.383763
C	-0.130672	-2.965827	-1.856213
H	0.565216	-2.607697	-1.084433
H	-0.342079	-4.036984	-1.694902
C	-4.768663	0.748930	-3.890259
H	-4.543212	1.822062	-4.008416
H	-5.449273	0.441024	-4.703635
C	0.275683	-2.594844	-3.318067
H	1.203008	-2.002281	-3.390481
H	0.343630	-3.442084	-4.022290
C	-3.494035	-0.120478	-3.741132
C	-3.587865	-1.626592	-4.094232
C	-2.188330	0.583448	-3.300791
C	-2.292812	-2.472320	-3.939136
H	-4.223494	-1.901602	-4.953334
C	-0.906377	-0.252687	-3.186569
H	-2.060530	1.628956	-3.631078
C	-1.005909	-1.747299	-3.468468

H	-2.138583	-3.261211	-4.695052
H	0.045008	0.231717	-3.457583
Co	-0.055178	0.182487	-0.086287
C	-2.817062	-2.399287	0.945829
C	-3.086375	-0.571034	2.049093
H	-2.913069	-3.029534	0.055352
H	-3.413997	0.469782	2.157118
O	-3.418980	-1.101053	0.743545
C	-1.402354	-1.999655	1.405999
H	-0.499895	-2.599540	1.294488
C	-1.573469	-0.861438	2.109076
H	-0.856540	-0.331469	2.736637
C	-3.748549	-1.640431	2.936398
C	-4.389272	-1.628875	4.167494
C	-3.568874	-2.840680	2.215045
C	-4.867870	-2.860710	4.673880
H	-4.531977	-0.700914	4.731777
C	-4.025425	-4.054573	2.708976
C	-4.689386	-4.050567	3.958820
H	-5.389013	-2.882089	5.637071
H	-3.888739	-4.989403	2.154254

H	-5.072513	-4.990915	4.369591

TS1-*ent1*

Lowest frequency = -184.3112 cm^{-1}

Charge = 0, Multiplicity = 1

123

C	-0.212351	0.600954	1.871940
C	-0.652121	-1.626313	3.277440
C	1.297614	0.392201	2.169531
C	0.863621	-1.873872	3.494646
C	1.873242	-0.840412	2.922534
H	1.199186	-2.924147	3.459587
H	2.813433	-1.260462	2.529092
N	-2.277356	-0.509009	0.336821
C	-2.637656	-0.734294	-1.962139
C	-0.677585	-0.392047	-3.194868
C	-3.399221	-1.042759	-3.130430
C	-3.207362	-0.764769	-0.650469

C	-1.353476	-0.698657	-4.398499
H	0.380389	-0.122934	-3.168217
C	-2.703947	-1.014001	-4.371886
C	-4.778380	-1.349508	-2.962652
C	-4.572645	-1.059920	-0.524287
H	-0.794877	-0.677883	-5.338680
H	-3.243434	-1.246949	-5.296222
C	-5.331420	-1.342209	-1.685296
H	-5.388050	-1.583171	-3.841057
H	-5.017105	-1.079260	0.470478
H	-6.396552	-1.569438	-1.564596
N	-1.308615	-0.403460	-2.022048
C	-2.492126	-0.660037	1.673695
O	-3.549717	-1.029073	2.207127
O	1.004569	0.824190	-1.096012
C	2.222605	0.350143	-1.090146
C	2.566292	-0.928938	-0.543081
C	3.290882	1.149361	-1.649472
C	3.916209	-1.355056	-0.458196
C	1.532394	-1.834187	-0.098181
C	4.595947	0.672451	-1.531869

C	4.955011	-0.559348	-0.923918
H	4.105183	-2.333314	-0.013344
N	0.252381	-1.603497	0.039179
O	1.926016	-3.106727	0.183883
H	5.403969	1.292089	-1.928674
C	-0.429032	-2.891642	0.323663
C	0.761499	-3.827827	0.652804
H	-1.085598	-2.773390	1.195860
C	-1.259913	-3.396005	-0.841991
H	0.877148	-4.006407	1.733535
C	-0.693872	-3.529949	-2.122853
C	-2.594197	-3.783821	-0.638054
C	-1.453919	-4.038226	-3.183236
H	0.343711	-3.221557	-2.293472
C	-3.355938	-4.295369	-1.698028
H	-3.044787	-3.659133	0.352554
C	-2.787242	-4.423113	-2.972031
H	-1.007193	-4.128489	-4.178882
H	-4.399876	-4.577693	-1.530046
H	-3.384433	-4.812482	-3.803323
C	-1.241193	-0.396482	2.483129

C	2.996720	2.495948	-2.345320
C	6.436684	-0.953819	-0.817604
C	7.188792	0.114229	0.014265
H	8.260714	-0.142705	0.098540
H	7.115543	1.112978	-0.450116
H	6.766293	0.182365	1.032247
C	7.056646	-1.031738	-2.234310
H	6.982690	-0.065122	-2.762179
H	8.126607	-1.303113	-2.174594
H	6.538569	-1.792148	-2.844496
C	6.616077	-2.321216	-0.130972
H	6.206501	-2.315757	0.894432
H	6.117573	-3.127553	-0.696838
H	7.690152	-2.569023	-0.064623
C	2.379857	3.511972	-1.352050
H	1.433698	3.131176	-0.940831
H	3.075182	3.715093	-0.519149
H	2.173850	4.467254	-1.868423
C	4.278529	3.132571	-2.922246
H	5.012985	3.370116	-2.132609
H	4.766007	2.475989	-3.664484

H	4.016629	4.077924	-3.428821
C	2.016373	2.276659	-3.524275
H	2.429249	1.547920	-4.244689
H	1.048483	1.904164	-3.159217
H	1.845552	3.229484	-4.057710
H	0.717489	-4.787487	0.116845
C	2.338196	1.436268	1.673209
H	2.033781	1.975333	0.769672
H	3.333861	1.000774	1.489515
C	-1.620966	-2.691547	3.858824
H	-2.563500	-2.812227	3.303915
H	-1.143930	-3.678218	3.994635
C	2.193117	2.241860	3.001071
H	1.760846	3.251067	2.881253
H	3.109644	2.329085	3.609407
C	-1.748957	-1.834808	5.165026
H	-2.756508	-1.413489	5.314126
H	-1.429446	-2.329610	6.098613
C	1.187906	1.185034	3.503729
C	1.776585	-0.021339	4.277263
C	-0.302132	1.446441	3.262071

C	0.775461	-1.059417	4.851550
H	2.654635	0.197302	4.908165
C	-1.307154	0.415754	3.827595
H	-0.606963	2.505267	3.319370
C	-0.735721	-0.813639	4.586631
H	1.046311	-1.489661	5.830481
H	-2.264182	0.823254	4.192583
Co	-0.622237	0.164583	-0.286578
C	-2.752274	2.400828	-0.808211
C	-2.183152	2.665876	1.250284
H	-3.409172	2.000687	-1.591706
H	-2.351847	2.522389	2.322179
O	-3.222698	2.026795	0.499429
C	-2.658039	3.924567	-0.644248
C	-2.273838	4.096804	0.704682
C	-2.795515	5.015521	-1.495536
C	-2.021480	5.359076	1.229188
C	-2.565676	6.303423	-0.962361
H	-3.081182	4.890645	-2.545649
C	-2.185933	6.473181	0.376091
H	-1.713774	5.496723	2.271665

H	-2.688800	7.183026	-1.603470
H	-2.016692	7.482607	0.765960
C	-0.874106	2.124340	0.600742
H	0.046843	2.669652	0.825226
C	-1.281384	1.955353	-0.778719
H	-0.632655	2.287821	-1.594307

TS1-*ent1*

Lowest frequency = -154.3125 cm^{-1}

Charge = 0, Multiplicity = 3

123

C	-0.404566	0.102096	2.038058
C	-0.810664	-2.378551	2.888381
C	1.099059	-0.120306	2.308161
C	0.701793	-2.620545	3.124605
C	1.687581	-1.460353	2.820500
H	1.079999	-3.631016	2.894886
H	2.662894	-1.757265	2.402198
N	-2.329750	-0.736703	0.169258

C	-2.646617	-0.357793	-2.129610
C	-0.740289	0.449429	-3.218207
C	-3.391570	-0.412622	-3.353308
C	-3.218952	-0.770889	-0.874991
C	-1.389523	0.399028	-4.472848
H	0.291264	0.796156	-3.107244
C	-2.709835	-0.020483	-4.538404
C	-4.744172	-0.847433	-3.303872
C	-4.567366	-1.167525	-0.871227
H	-0.843132	0.701280	-5.371124
H	-3.239439	-0.056410	-5.496885
C	-5.302427	-1.197427	-2.078356
H	-5.328181	-0.895629	-4.228499
H	-5.016234	-1.465574	0.076710
H	-6.349436	-1.519546	-2.041066
N	-1.356023	0.085244	-2.095782
C	-2.619140	-1.145001	1.425291
O	-3.683466	-1.621113	1.860012
O	1.170220	1.079373	-0.875994
C	2.396345	0.640715	-0.931534
C	2.734776	-0.729468	-0.664274

C	3.478691	1.550076	-1.241908
C	4.085698	-1.160398	-0.629679
C	1.697362	-1.714306	-0.464843
C	4.783474	1.064646	-1.182991
C	5.130791	-0.276239	-0.865232
H	4.271917	-2.212697	-0.409955
N	0.405843	-1.532690	-0.321094
O	2.104062	-3.013610	-0.449767
H	5.600468	1.760659	-1.389739
C	-0.254367	-2.862445	-0.337589
C	0.942632	-3.831435	-0.170030
H	-0.942963	-2.935025	0.514363
C	-1.034392	-3.111082	-1.615381
H	1.035692	-4.222489	0.856053
C	-0.452480	-2.869228	-2.872622
C	-2.336693	-3.633152	-1.551570
C	-1.165302	-3.141234	-4.046950
H	0.556812	-2.447277	-2.932465
C	-3.050972	-3.907824	-2.725845
H	-2.803508	-3.797017	-0.574276
C	-2.466828	-3.661522	-3.975328

H	-0.707969	-2.937010	-5.020660
H	-4.072937	-4.294036	-2.662121
H	-3.029338	-3.862178	-4.893244
C	-1.409968	-1.026092	2.343541
C	3.189893	3.020334	-1.607459
C	6.611395	-0.684297	-0.805783
C	7.330439	0.162536	0.273287
H	8.400976	-0.107436	0.327826
H	7.264515	1.241681	0.050792
H	6.878091	-0.007267	1.266230
C	7.272797	-0.430796	-2.182858
H	7.207342	0.631911	-2.473912
H	8.342345	-0.708521	-2.155016
H	6.778466	-1.028957	-2.968146
C	6.781219	-2.173530	-0.449776
H	6.342921	-2.406529	0.536539
H	6.305258	-2.827002	-1.201573
H	7.854842	-2.428838	-0.411247
C	2.513702	3.755515	-0.423062
H	1.553000	3.285188	-0.167638
H	3.165266	3.738689	0.468110

H	2.324786	4.810922	-0.691507
C	4.481825	3.789144	-1.954216
H	5.176240	3.836931	-1.096827
H	5.014448	3.334354	-2.808103
H	4.223484	4.825965	-2.232019
C	2.264537	3.078193	-2.848318
H	2.734964	2.569559	-3.708534
H	1.301466	2.592095	-2.634621
H	2.072751	4.129338	-3.131068
H	0.931895	-4.663680	-0.889142
C	2.120575	1.031369	2.093385
H	1.847537	1.720555	1.285770
H	3.140544	0.664954	1.895347
C	-1.764793	-3.565435	3.183728
H	-2.678719	-3.579924	2.570972
H	-1.265317	-4.548728	3.128812
C	1.873677	1.558819	3.539110
H	1.401016	2.555216	3.590860
H	2.754736	1.559439	4.203392
C	-1.976304	-3.008651	4.633516
H	-3.004556	-2.660156	4.822503

H	-1.670739	-3.678718	5.455823
C	0.892494	0.393128	3.778353
C	1.500142	-0.930961	4.306199
C	-0.599612	0.656174	3.538936
C	0.524024	-2.098520	4.611876
H	2.347347	-0.817633	5.003449
C	-1.574593	-0.512112	3.821014
H	-0.955508	1.666962	3.799763
C	-0.983893	-1.855221	4.332133
H	0.777936	-2.705848	5.496952
H	-2.568965	-0.229878	4.202643
Co	-0.531547	0.243020	-0.190910
C	-2.678889	2.523631	-0.543259
C	-2.388641	2.356730	1.584076
H	-3.196833	2.273727	-1.477874
H	-2.659381	1.962296	2.569899
O	-3.272709	1.844466	0.576799
C	-2.732973	3.977403	-0.046839
C	-2.540785	3.867404	1.349118
C	-2.864317	5.218176	-0.659078
C	-2.479611	4.994673	2.159636

C	-2.825650	6.367756	0.162179
H	-3.003529	5.311470	-1.741583
C	-2.637199	6.258263	1.546328
H	-2.324795	4.916697	3.241438
H	-2.947825	7.358282	-0.289107
H	-2.614545	7.164092	2.161654
C	-0.988082	2.042059	1.003924
H	-0.103579	2.459360	1.489697
C	-1.192810	2.156446	-0.400543
H	-0.451246	2.593856	-1.073259

TS1-*ent1*

Lowest frequency = -211.3521 cm^{-1}

Charge = 0, Multiplicity = 5

123

C	-0.208003	0.921345	1.951279
C	-0.315916	-1.360044	3.302112
C	1.326597	0.790053	1.992319
C	1.225106	-1.487846	3.367231

C	2.071854	-0.374139	2.699703
H	1.643776	-2.504708	3.290829
H	3.006537	-0.706900	2.222966
N	-2.196678	-0.679389	0.510697
C	-2.866133	-1.074595	-1.735135
C	-1.200023	-0.660079	-3.321342
C	-3.796431	-1.492033	-2.742801
C	-3.219453	-1.065403	-0.337789
C	-2.043407	-1.073815	-4.378287
H	-0.174119	-0.320270	-3.498316
C	-3.336415	-1.482471	-4.089308
C	-5.105040	-1.880089	-2.344806
C	-4.531192	-1.435956	0.003147
H	-1.666982	-1.061166	-5.405502
H	-4.015689	-1.801348	-4.887667
C	-5.446617	-1.833985	-0.998651
H	-5.825828	-2.201408	-3.103649
H	-4.818057	-1.414078	1.053995
H	-6.459086	-2.119839	-0.691738
N	-1.606072	-0.658784	-2.054840
C	-2.354183	-0.594342	1.866335

O	-3.360061	-0.872631	2.535142
O	1.066674	0.616529	-1.328203
C	2.330192	0.293681	-1.269053
C	2.779058	-0.896361	-0.597593
C	3.328422	1.127502	-1.899001
C	4.162038	-1.177260	-0.455480
C	1.824890	-1.861119	-0.095721
C	4.669938	0.800894	-1.709197
C	5.128988	-0.324720	-0.973239
H	4.436431	-2.089442	0.077193
N	0.523427	-1.742592	-0.018612
O	2.325045	-3.053365	0.332730
H	5.424700	1.451714	-2.158062
C	-0.054223	-3.020833	0.439032
C	1.205021	-3.896074	0.702897
H	-0.613456	-2.837169	1.369329
C	-1.008731	-3.614964	-0.579128
H	1.321763	-4.180785	1.760442
C	-0.631643	-3.731587	-1.929084
C	-2.278040	-4.063789	-0.180757
C	-1.511152	-4.289692	-2.864478

H	0.347006	-3.358752	-2.251836
C	-3.159397	-4.625809	-1.114896
H	-2.587419	-3.946189	0.863464
C	-2.777422	-4.738934	-2.458001
H	-1.214026	-4.362856	-3.915761
H	-4.153328	-4.954022	-0.795629
H	-3.470962	-5.163061	-3.191649
C	-1.075099	-0.182926	2.575846
C	2.911429	2.328493	-2.772370
C	6.638660	-0.556875	-0.801970
C	7.256856	0.654950	-0.061812
H	8.345954	0.516507	0.067190
H	7.098574	1.593421	-0.620984
H	6.800933	0.774438	0.936881
C	7.302856	-0.703072	-2.193100
H	7.150062	0.198045	-2.811930
H	8.391958	-0.859196	-2.086885
H	6.879495	-1.564914	-2.737920
C	6.933014	-1.829343	0.015031
H	6.496094	-1.770489	1.027405
H	6.533570	-2.731148	-0.480971

H	8.024018	-1.959551	0.124822
C	2.147724	3.388098	-1.941390
H	1.242108	2.954984	-1.491930
H	2.788591	3.789353	-1.137045
H	1.845454	4.230208	-2.590165
C	4.131773	3.025607	-3.408063
H	4.808450	3.448904	-2.644730
H	4.713616	2.336174	-4.044948
H	3.783466	3.858529	-4.043534
C	2.002628	1.828287	-3.923273
H	2.531922	1.080936	-4.540629
H	1.088784	1.369585	-3.517764
H	1.713687	2.673575	-4.574097
H	1.236542	-4.798009	0.070833
C	2.236895	1.896329	1.399474
H	1.840201	2.372486	0.494509
H	3.249202	1.527629	1.168366
C	-1.144659	-2.495112	3.960925
H	-2.130114	-2.670383	3.502527
H	-0.598273	-3.452400	4.029625
C	2.130037	2.728423	2.715059

H	1.578742	3.679052	2.610636
H	3.084896	2.935388	3.228195
C	-1.195791	-1.663151	5.287864
H	-2.210288	-1.331707	5.562402
H	-0.726801	-2.137575	6.167225
C	1.286506	1.602287	3.351231
C	2.047913	0.451663	4.050710
C	-0.242504	1.747280	3.304685
C	1.202466	-0.663739	4.725545
H	2.969942	0.742641	4.581900
C	-1.097049	0.619356	3.936968
H	-0.631766	2.776110	3.396545
C	-0.343679	-0.553399	4.619945
H	1.610233	-1.053903	5.673477
H	-2.054449	0.928002	4.387116
Co	-0.525267	0.004019	-0.385669
C	-3.021804	2.243272	-0.819023
C	-2.359600	2.762980	1.164485
H	-3.659323	1.685085	-1.517627
H	-2.415611	2.688668	2.256121
O	-3.342267	1.914301	0.549413

C	-3.159142	3.772434	-0.771195
C	-2.713150	4.112872	0.526666
C	-3.535289	4.755982	-1.679703
C	-2.635506	5.436321	0.941044
C	-3.476572	6.103953	-1.258483
H	-3.876570	4.503154	-2.689565
C	-3.035779	6.439716	0.028348
H	-2.285992	5.704257	1.944419
H	-3.785312	6.898225	-1.946940
H	-3.004127	7.491537	0.332293
C	-1.050363	2.323505	0.453759
H	-0.131411	2.894837	0.608721
C	-1.508637	2.002050	-0.850190
H	-0.912260	2.143353	-1.756182

TS1-*ent2*

Lowest frequency = -240.3855 cm^{-1}

Charge = 0, Multiplicity = 1

123

C	-1.499323	-1.652899	-1.728957
C	0.778471	-2.818552	-0.965035
C	-1.872850	-3.039648	-2.371510
C	0.397285	-4.207377	-1.537485
C	-0.973352	-4.308213	-2.265151
H	0.696980	-5.094457	-0.954001
H	-1.509987	-5.261719	-2.121499
N	-2.272917	0.274198	-0.456035
C	-2.567609	2.085234	1.003390
C	-0.586188	2.628636	2.134795
C	-3.292582	3.142037	1.635589
C	-3.163809	1.206477	0.042820
C	-1.229400	3.698897	2.795740
H	0.464184	2.377523	2.295580
C	-2.570230	3.955877	2.552699
C	-4.665545	3.298385	1.302889
C	-4.527484	1.389565	-0.250941
H	-0.651205	4.312423	3.492535
H	-3.083265	4.781866	3.056531
C	-5.247499	2.426431	0.387543
H	-5.245239	4.100266	1.770278

H	-5.002313	0.732267	-0.977874
H	-6.307815	2.545411	0.137630
N	-1.245583	1.851791	1.274347
C	-2.616537	-0.683768	-1.376372
O	-3.687067	-0.682219	-1.998525
O	1.128794	0.429558	1.286738
C	2.323282	0.688251	0.817402
C	2.554423	1.268537	-0.475985
C	3.482901	0.403222	1.635065
C	3.867565	1.437382	-0.986575
C	1.445560	1.725028	-1.279295
C	4.744183	0.578049	1.065419
C	4.981575	1.065085	-0.246974
H	3.964030	1.863269	-1.986595
N	0.173039	1.506091	-1.086802
O	1.744269	2.447251	-2.398430
H	5.616691	0.322705	1.671830
C	-0.595668	2.238723	-2.115908
C	0.509134	2.591615	-3.144156
H	-1.347752	1.566151	-2.554665
C	-1.308590	3.460219	-1.560454

H	0.536460	1.885414	-3.993305
C	-0.630890	4.376113	-0.736183
C	-2.648471	3.706709	-1.904500
C	-1.285627	5.520949	-0.264009
H	0.409539	4.185146	-0.451987
C	-3.304722	4.851120	-1.431406
H	-3.185638	2.983599	-2.527426
C	-2.623408	5.761469	-0.611719
H	-0.751703	6.223867	0.384076
H	-4.353621	5.022824	-1.692477
H	-3.137184	6.652130	-0.234636
C	-0.182113	-1.586146	-0.881346
C	3.333833	-0.056367	3.101943
C	6.420787	1.159120	-0.777355
C	7.042621	-0.259305	-0.798565
H	8.082380	-0.222438	-1.172305
H	7.060465	-0.706410	0.210628
H	6.459725	-0.929119	-1.455102
C	7.260059	2.075702	0.145833
H	7.288518	1.692722	1.180683
H	8.301736	2.141485	-0.218059

H	6.836153	3.094805	0.172473
C	6.466096	1.733228	-2.206242
H	5.902595	1.100824	-2.914495
H	6.046917	2.753850	-2.246333
H	7.512080	1.783192	-2.556685
C	2.615611	-1.423758	3.197080
H	1.614072	-1.367999	2.748331
H	3.195735	-2.208161	2.681373
H	2.505236	-1.719296	4.256316
C	4.704160	-0.202414	3.796505
H	5.328915	-0.977365	3.318343
H	5.268513	0.746843	3.799271
H	4.546224	-0.504045	4.846696
C	2.521274	0.998893	3.893624
H	2.998606	1.992708	3.825071
H	1.495917	1.067860	3.502727
H	2.468587	0.713484	4.960011
H	0.452301	3.624847	-3.515572
C	-3.194993	-3.264825	-3.156640
H	-4.070859	-2.715240	-2.791396
H	-3.433637	-4.339969	-3.236120

C	2.148971	-2.694336	-0.239089
H	2.184930	-1.884808	0.502378
H	2.495137	-3.625295	0.242226
C	-2.508930	-2.677620	-4.436172
H	-2.885586	-1.673776	-4.694552
H	-2.523619	-3.306482	-5.343977
C	2.834297	-2.319342	-1.590120
H	3.297772	-1.319283	-1.600741
H	3.567558	-3.054189	-1.963337
C	-1.158742	-2.622267	-3.675603
C	-0.283233	-3.900395	-3.632618
C	-0.732825	-1.262529	-3.057463
C	1.082182	-3.780833	-2.903817
H	-0.289673	-4.543820	-4.528597
C	0.615509	-1.135692	-2.338397
H	-1.045201	-0.366488	-3.618903
C	1.455393	-2.403473	-2.280609
H	1.918524	-4.340401	-3.355116
H	1.163018	-0.199694	-2.486688
Co	-0.558312	0.336289	0.348257
C	-1.622754	-2.894483	0.916679

C	-2.555163	-1.326028	2.075011
H	-1.608432	-3.612587	0.089996
H	-3.371530	-0.617234	2.266457
O	-2.827415	-2.109897	0.890569
C	-0.517177	-1.796749	0.956238
H	0.471696	-2.133305	1.270523
C	-1.138168	-0.777550	1.816036
H	-0.582451	-0.442028	2.700476
C	-2.297977	-2.442028	3.091971
C	-2.472225	-2.577779	4.465988
C	-1.663068	-3.453006	2.336655
C	-2.019226	-3.767875	5.075656
H	-2.951530	-1.794118	5.062695
C	-1.189512	-4.615514	2.935331
C	-1.387854	-4.769906	4.324485
H	-2.164441	-3.912174	6.151784
H	-0.685768	-5.394788	2.352903
H	-1.047294	-5.684343	4.821899

TS1-*ent2*

Lowest frequency = -245.6777 cm^{-1}

Charge = 0, Multiplicity = 3

123

C	-1.785865	-1.532129	-1.729845
C	0.382742	-2.886830	-1.005784
C	-2.330017	-2.896859	-2.286376
C	-0.148656	-4.251316	-1.502555
C	-1.554333	-4.243816	-2.163926
H	0.096780	-5.141023	-0.898875
H	-2.174800	-5.132635	-1.958113
N	-2.284995	0.506777	-0.512761
C	-2.376131	2.343824	0.981302
C	-0.384589	2.709510	2.156137
C	-3.014117	3.466226	1.605151
C	-3.055829	1.521280	0.008168
C	-0.927629	3.842379	2.803578
H	0.635875	2.359526	2.341473
C	-2.235108	4.215230	2.530904
C	-4.363069	3.756348	1.266179
C	-4.399353	1.837428	-0.273885

H	-0.311361	4.404936	3.511096
H	-2.682939	5.086196	3.021919
C	-5.023319	2.938687	0.355026
H	-4.862807	4.614147	1.727276
H	-4.934434	1.223672	-0.997870
H	-6.067286	3.155179	0.100809
N	-1.094879	1.999000	1.284371
C	-2.771334	-0.411254	-1.396196
O	-3.841798	-0.329551	-2.017919
O	1.336331	0.238454	1.238071
C	2.542787	0.418466	0.787939
C	2.807337	0.985443	-0.510357
C	3.686205	0.045056	1.596841
C	4.129387	1.069677	-1.022710
C	1.729679	1.511293	-1.307400
C	4.953473	0.143622	1.028890
C	5.216856	0.627526	-0.283095
H	4.255079	1.489983	-2.021757
N	0.437120	1.435594	-1.085666
O	2.073321	2.168521	-2.450760
H	5.810671	-0.171450	1.629566

C	-0.272416	2.208146	-2.128867
C	0.848952	2.457686	-3.170255
H	-1.075566	1.582658	-2.546885
C	-0.893732	3.487694	-1.598042
H	0.788279	1.772348	-4.034244
C	-0.160404	4.356059	-0.770592
C	-2.204668	3.833034	-1.967689
C	-0.732191	5.552827	-0.319942
H	0.855409	4.085301	-0.462823
C	-2.776964	5.030783	-1.518371
H	-2.787264	3.145363	-2.589876
C	-2.040655	5.893273	-0.694756
H	-0.157511	6.217519	0.333506
H	-3.805233	5.279896	-1.797976
H	-2.490373	6.824221	-0.333565
C	-0.428571	-1.574803	-0.975941
C	3.494917	-0.434200	3.049900
C	6.660271	0.645071	-0.809848
C	7.207390	-0.803839	-0.831915
H	8.249401	-0.819251	-1.200631
H	7.197708	-1.254280	0.175831

H	6.594319	-1.440699	-1.493660
C	7.542851	1.514232	0.119286
H	7.547190	1.127717	1.153148
H	8.587745	1.525888	-0.240998
H	7.173032	2.554113	0.146940
C	6.741766	1.219402	-2.237009
H	6.147034	0.620686	-2.948981
H	6.379507	2.261599	-2.275783
H	7.790160	1.212932	-2.583604
C	2.672530	-1.745203	3.098456
H	1.675462	-1.593542	2.661668
H	3.186408	-2.549348	2.543592
H	2.548782	-2.074757	4.146163
C	4.844652	-0.703714	3.746554
H	5.409852	-1.513056	3.251241
H	5.481234	0.198342	3.774400
H	4.658392	-1.017027	4.788672
C	2.759168	0.663929	3.858788
H	3.334914	1.606568	3.848640
H	1.762451	0.851777	3.433032
H	2.638141	0.344006	4.909770

H	0.897332	3.498818	-3.520967
C	-3.705199	-3.018998	-2.998554
H	-4.497701	-2.361215	-2.621743
H	-4.058404	-4.065094	-3.013914
C	1.792089	-2.863933	-0.348607
H	1.924864	-2.034974	0.359854
H	2.073325	-3.806560	0.151251
C	-3.029012	-2.561874	-4.334842
H	-3.318727	-1.538464	-4.624529
H	-3.148016	-3.227552	-5.207999
C	2.451834	-2.605550	-1.738240
H	3.006270	-1.655530	-1.810199
H	3.096863	-3.420606	-2.107297
C	-1.642159	-2.606711	-3.641811
C	-0.892284	-3.959956	-3.576475
C	-1.050446	-1.272494	-3.116797
C	0.510709	-3.945040	-2.912110
H	-0.998947	-4.636074	-4.441396
C	0.335673	-1.245512	-2.470300
H	-1.314939	-0.370949	-3.693817
C	1.048121	-2.583961	-2.378014

H	1.268307	-4.602106	-3.370977
H	0.958007	-0.364001	-2.664550
Co	-0.435482	0.317097	0.281353
C	-1.987446	-2.631837	1.076226
C	-2.602063	-0.897913	2.210564
H	-2.112595	-3.357741	0.264499
H	-3.275203	-0.052903	2.403597
O	-3.043295	-1.655903	1.063511
C	-0.723569	-1.741182	1.082355
H	0.239334	-2.211947	1.280085
C	-1.120255	-0.615430	1.896149
H	-0.472207	-0.263219	2.705455
C	-2.507548	-2.016877	3.256802
C	-2.666857	-2.083774	4.636734
C	-2.088205	-3.145200	2.515580
C	-2.422631	-3.321875	5.271364
H	-2.980061	-1.211155	5.220103
C	-1.820861	-4.359565	3.137587
C	-2.006356	-4.440214	4.535441
H	-2.562303	-3.410369	6.354167
H	-1.486137	-5.233457	2.567916

H	-1.825807	-5.389446	5.051226

Int3-*ent1*

Lowest frequency = 12.7805 cm^{-1}

Charge = 0, Multiplicity = 1

123

C	-0.430682	1.957169	1.504344
C	-0.362112	-0.049441	3.340354
C	1.146356	1.903778	1.627990
C	1.181202	-0.082314	3.412520
C	1.958384	0.923213	2.524841
H	1.658703	-1.069993	3.521787
H	2.894636	0.539816	2.088266
N	-2.202160	-0.468829	0.683930
C	-2.753039	-1.556688	-1.308521
C	-0.966478	-1.520609	-2.840685
C	-3.581439	-2.305169	-2.199691
C	-3.186573	-1.143297	-0.010973

C	-1.721054	-2.275551	-3.764058
H	0.051304	-1.187044	-3.044495
C	-3.016594	-2.663914	-3.455699
C	-4.896117	-2.623782	-1.764198
C	-4.503448	-1.453716	0.370547
H	-1.261860	-2.539156	-4.721015
H	-3.612236	-3.244622	-4.167657
C	-5.326991	-2.188211	-0.513252
H	-5.554747	-3.200189	-2.421023
H	-4.860782	-1.119614	1.343787
H	-6.347421	-2.423759	-0.190813
N	-1.479128	-1.170314	-1.653987
C	-2.432229	0.195803	1.867795
O	-3.471951	0.130756	2.535189
O	1.002762	0.225783	-1.299413
C	2.245413	-0.161468	-1.145627
C	2.656215	-1.117540	-0.154466
C	3.271216	0.390891	-2.005307
C	4.026034	-1.418651	0.059563
C	1.671172	-1.838396	0.610768
C	4.598184	0.063631	-1.729499

C	5.020771	-0.814221	-0.695472
H	4.263528	-2.141957	0.841274
N	0.373921	-1.643505	0.628080
O	2.108807	-2.868894	1.381502
H	5.373893	0.507962	-2.357462
C	-0.260349	-2.744072	1.386045
C	0.956433	-3.429109	2.061327
H	-0.945088	-2.319132	2.131162
C	-1.051147	-3.668774	0.477531
H	1.039694	-3.187919	3.134141
C	-0.502599	-4.136145	-0.730201
C	-2.338426	-4.087989	0.853306
C	-1.231418	-5.009695	-1.547319
H	0.492622	-3.799946	-1.041563
C	-3.068520	-4.963004	0.037514
H	-2.778456	-3.707850	1.781260
C	-2.515325	-5.426039	-1.164008
H	-0.799492	-5.359572	-2.490704
H	-4.076829	-5.268366	0.333551
H	-3.088747	-6.100808	-1.808303
C	-1.210108	0.905123	2.399486

C	2.917778	1.291817	-3.209210
C	6.520757	-1.064343	-0.474785
C	7.212340	0.278199	-0.131460
H	8.296183	0.126786	0.023911
H	7.086242	1.016381	-0.942406
H	6.785914	0.711116	0.790457
C	7.145109	-1.649507	-1.765336
H	7.023181	-0.964270	-2.622044
H	8.227293	-1.825050	-1.625037
H	6.668311	-2.610038	-2.027928
C	6.771268	-2.052469	0.680302
H	6.360843	-1.673915	1.632831
H	6.317567	-3.038144	0.476472
H	7.856828	-2.202096	0.815769
C	2.252972	2.611371	-2.752527
H	1.339483	2.405823	-2.176260
H	2.944434	3.200035	-2.126079
H	1.983007	3.220904	-3.634086
C	4.169972	1.661180	-4.032494
H	4.895952	2.245906	-3.440665
H	4.682894	0.766889	-4.428658

H	3.864785	2.283420	-4.891703
C	1.945325	0.542591	-4.153507
H	2.359832	-0.436496	-4.453085
H	0.977383	0.387408	-3.656931
H	1.772123	1.139410	-5.067160
H	0.977841	-4.519932	1.919792
C	2.010603	2.888498	0.800990
H	1.602231	3.180447	-0.175893
H	3.035957	2.517428	0.633945
C	-1.128397	-1.070185	4.231787
H	-2.108966	-1.388119	3.843028
H	-0.531782	-1.965912	4.480078
C	1.871494	3.956136	1.937048
H	1.270095	4.838142	1.654072
H	2.816189	4.312427	2.383804
C	-1.209622	-0.002378	5.373140
H	-2.237141	0.322064	5.605259
H	-0.702145	-0.274817	6.314746
C	1.088412	2.926637	2.788765
C	1.915215	1.986777	3.699923
C	-0.455691	2.966592	2.735194

C	1.122638	0.984192	4.585848
H	2.821429	2.414493	4.161925
C	-1.256572	1.949340	3.584421
H	-0.910568	3.968122	2.641964
C	-0.430376	0.991396	4.478596
H	1.543476	0.800371	5.589147
H	-2.218784	2.305624	3.985080
Co	-0.591952	-0.225020	-0.338156
C	-2.786952	1.726430	-1.295233
C	-2.473652	3.103183	0.330632
H	-3.344932	0.917459	-1.784874
H	-2.728258	3.513972	1.316422
O	-3.349802	2.012956	0.002927
C	-2.843342	3.102754	-1.950031
C	-2.645079	4.004641	-0.883072
C	-2.956100	3.556381	-3.262534
C	-2.560929	5.376661	-1.104128
C	-2.902246	4.947394	-3.489284
H	-3.093454	2.860725	-4.097875
C	-2.709128	5.843974	-2.427120
H	-2.394473	6.080507	-0.280939

H	-3.017212	5.334722	-4.507596
H	-2.675714	6.920254	-2.628355
C	-1.037022	2.472146	0.181942
H	-0.348922	3.266453	-0.153046
C	-1.287496	1.480185	-0.990777
H	-0.658185	1.684479	-1.870124

Int3-*ent1*

Lowest frequency = 14.1178 cm^{-1}

Charge = 0, Multiplicity = 3

123

C	0.094781	1.991287	1.530053
C	0.090103	-0.139155	3.221465
C	1.651380	1.724725	1.460745
C	1.614242	-0.373352	3.112757
C	2.418984	0.591713	2.204513
H	1.960828	-1.420289	3.105045
H	3.246783	0.129631	1.644423
N	-2.140149	-0.176632	0.832158

C	-3.211613	-1.153001	-1.061422
C	-1.819157	-1.396244	-2.925219
C	-4.331569	-1.716934	-1.754339
C	-3.316356	-0.684751	0.297448
C	-2.868494	-1.961979	-3.685233
H	-0.814539	-1.252555	-3.336317
C	-4.114904	-2.125852	-3.100562
C	-5.571785	-1.823404	-1.069219
C	-4.571213	-0.785129	0.923338
H	-2.682055	-2.265505	-4.719612
H	-4.944936	-2.565774	-3.664073
C	-5.667223	-1.356025	0.236069
H	-6.435000	-2.260712	-1.580932
H	-4.674141	-0.413473	1.941605
H	-6.626379	-1.423694	0.761528
N	-1.995732	-1.012206	-1.663566
C	-2.090900	0.475344	2.052294
O	-3.016998	0.541263	2.868789
O	0.835611	-0.099173	-1.566609
C	2.072777	-0.494166	-1.388836
C	2.432243	-1.468287	-0.396399

C	3.119002	-0.002473	-2.255554
C	3.783159	-1.843316	-0.187546
C	1.402748	-2.136334	0.361687
C	4.429378	-0.389530	-1.978654
C	4.806998	-1.282779	-0.939855
H	3.985512	-2.579143	0.592558
N	0.132031	-1.822435	0.424997
O	1.761618	-3.235298	1.073718
H	5.225111	0.015291	-2.608786
C	-0.582414	-2.840518	1.216276
C	0.576708	-3.729032	1.750287
H	-1.103738	-2.333090	2.040480
C	-1.613700	-3.595386	0.398809
H	0.733828	-3.621226	2.836069
C	-1.308699	-4.070239	-0.888730
C	-2.886595	-3.843861	0.939180
C	-2.264146	-4.784229	-1.622995
H	-0.327268	-3.856959	-1.326499
C	-3.842841	-4.560393	0.206930
H	-3.135719	-3.453275	1.931541
C	-3.532001	-5.032542	-1.075575

H	-2.022576	-5.138872	-2.630370
H	-4.836070	-4.733752	0.632461
H	-4.281403	-5.582195	-1.654867
C	-0.717839	0.980372	2.438284
C	2.788136	0.885626	-3.471802
C	6.293777	-1.590695	-0.704626
C	7.027193	-0.274699	-0.344637
H	8.102735	-0.464632	-0.174759
H	6.938068	0.471299	-1.153373
H	6.602837	0.168288	0.573599
C	6.912683	-2.190793	-1.990765
H	6.827635	-1.496789	-2.844782
H	7.985682	-2.406676	-1.837343
H	6.404606	-3.131662	-2.265439
C	6.494561	-2.592958	0.447969
H	6.089394	-2.203316	1.398306
H	6.005862	-3.559717	0.234685
H	7.572190	-2.784269	0.593573
C	2.171790	2.233032	-3.027744
H	1.282318	2.071397	-2.402313
H	2.905326	2.822461	-2.452112

H	1.875336	2.823731	-3.913470
C	4.044231	1.201715	-4.309946
H	4.792019	1.773572	-3.732509
H	4.525915	0.284780	-4.693109
H	3.753037	1.817864	-5.178422
C	1.790207	0.134638	-4.389884
H	2.218264	-0.826656	-4.725525
H	0.850003	-0.062860	-3.854775
H	1.566797	0.745353	-5.283385
H	0.460027	-4.792858	1.493162
C	2.547506	2.646876	0.594191
H	2.084168	3.056679	-0.315366
H	3.490928	2.159614	0.294849
C	-0.701699	-1.114718	4.141596
H	-1.751765	-1.286564	3.855631
H	-0.200229	-2.089777	4.273186
C	2.674314	3.642183	1.795763
H	2.167944	4.610145	1.633376
H	3.701082	3.845446	2.147280
C	-0.521745	-0.124956	5.339584
H	-1.467900	0.310656	5.699778

H	0.039224	-0.521765	6.203502
C	1.857330	2.664179	2.676483
C	2.643044	1.564772	3.433526
C	0.336794	2.907174	2.807383
C	1.823695	0.606826	4.344558
H	3.642630	1.839547	3.811828
C	-0.495021	1.936432	3.679591
H	0.013696	3.962415	2.833164
C	0.284221	0.819535	4.416739
H	2.320337	0.300684	5.281248
H	-1.355889	2.377374	4.206267
Co	-0.642652	-0.177861	-0.377249
C	-2.575778	2.147932	-0.941524
C	-1.874428	3.458058	0.619634
H	-3.294112	1.426958	-1.351610
H	-1.945708	3.894771	1.624341
O	-2.929070	2.501573	0.413082
C	-2.514431	3.521268	-1.599683
C	-2.064999	4.380543	-0.574289
C	-2.718599	3.992521	-2.895178
C	-1.816510	5.728315	-0.820538

C	-2.497610	5.362900	-3.143083
H	-3.051471	3.326968	-3.699376
C	-2.054965	6.218065	-2.121838
H	-1.455656	6.397705	-0.031632
H	-2.678051	5.767881	-4.144866
H	-1.895274	7.279913	-2.338536
C	-0.572527	2.629824	0.305639
H	0.175000	3.323481	-0.114949
C	-1.102461	1.691008	-0.809722
H	-0.554552	1.783584	-1.759964

Int3-*ent1*

Lowest frequency = 11.9177 cm^{-1}

Charge = 0, Multiplicity = 5

123

C	-0.272147	2.444844	-0.478680
C	0.709141	1.688375	-2.891830
C	-1.659100	1.997628	-1.080654
C	-0.670782	1.271416	-3.451039

C	-1.890630	1.427482	-2.510581
H	-0.700375	0.392558	-4.115793
H	-2.651434	0.634941	-2.587422
N	2.354232	0.500719	-0.313615
C	3.412852	-1.244232	0.933930
C	1.930360	-2.745025	1.972535
C	4.552135	-1.956840	1.434387
C	3.546829	-0.049631	0.134128
C	2.996889	-3.500740	2.500915
H	0.879436	-2.997892	2.145339
C	4.300556	-3.108407	2.227760
C	5.853334	-1.477208	1.121734
C	4.859336	0.400661	-0.111612
H	2.783610	-4.380300	3.115037
H	5.149699	-3.677844	2.621671
C	5.978776	-0.317849	0.368221
H	6.731056	-2.018150	1.489410
H	4.990192	1.319327	-0.680689
H	6.977789	0.066906	0.134547
N	2.144163	-1.665487	1.217110
C	2.324964	1.757532	-0.906324

O	3.310818	2.468051	-1.142130
O	-0.769527	-1.654121	1.158737
C	-1.979711	-1.698375	0.672816
C	-2.227929	-1.626689	-0.746929
C	-3.130751	-1.880099	1.531030
C	-3.538252	-1.660634	-1.278771
C	-1.101106	-1.627611	-1.648496
C	-4.397913	-1.843979	0.949676
C	-4.647779	-1.718510	-0.443619
H	-3.644862	-1.623429	-2.365078
N	0.119396	-1.250016	-1.366236
O	-1.278155	-2.098125	-2.908640
H	-5.265449	-1.932048	1.608753
C	1.007775	-1.541328	-2.506246
C	0.036853	-2.211821	-3.519432
H	1.393402	-0.588233	-2.893421
C	2.189671	-2.398761	-2.096403
H	0.007966	-1.709217	-4.497932
C	1.995470	-3.580885	-1.358997
C	3.495708	-2.010865	-2.437437
C	3.090927	-4.362616	-0.973037

H	0.982358	-3.870676	-1.057383
C	4.594104	-2.792194	-2.052974
H	3.652371	-1.077119	-2.987304
C	4.393024	-3.968985	-1.319600
H	2.930626	-5.273998	-0.387824
H	5.607907	-2.469050	-2.308546
H	5.250732	-4.572973	-1.005707
C	0.975201	2.224161	-1.422076
C	-2.950056	-2.155262	3.036539
C	-6.094820	-1.658514	-0.956574
C	-6.796984	-0.423456	-0.339386
H	-7.844459	-0.359624	-0.686539
H	-6.807052	-0.473721	0.763146
H	-6.276865	0.506035	-0.630767
C	-6.850888	-2.943277	-0.538235
H	-6.867756	-3.064506	0.558688
H	-7.898065	-2.906801	-0.890033
H	-6.369287	-3.837270	-0.971448
C	-6.152123	-1.534758	-2.491219
H	-5.643934	-0.619342	-2.842264
H	-5.682018	-2.402379	-2.986447

H	-7.203895	-1.485473	-2.823640
C	-2.160973	-1.021388	3.734342
H	-1.136021	-0.968247	3.342664
H	-2.650398	-0.044073	3.577940
H	-2.114422	-1.209695	4.822571
C	-4.303435	-2.298199	3.762473
H	-4.898883	-1.369681	3.704915
H	-4.908408	-3.125924	3.352449
H	-4.121259	-2.513950	4.829769
C	-2.179077	-3.490945	3.195683
H	-2.758059	-4.327359	2.766396
H	-1.208205	-3.436805	2.678892
H	-2.000187	-3.705169	4.265464
H	0.248305	-3.285109	-3.658720
C	-2.944712	2.119240	-0.222747
H	-2.831803	1.949088	0.861024
H	-3.748164	1.452203	-0.578251
C	1.929569	1.516380	-3.845499
H	2.893481	1.330851	-3.345604
H	1.774482	0.741302	-4.617771
C	-3.090289	3.612009	-0.679272

H	-2.914127	4.347371	0.125819
H	-4.039126	3.872397	-1.180840
C	1.736382	2.985947	-4.347636
H	2.573912	3.656152	-4.094368
H	1.508449	3.092012	-5.422471
C	-1.867388	3.437568	-1.618782
C	-2.121166	2.899670	-3.048490
C	-0.482106	3.886300	-1.094775
C	-0.879579	2.751963	-3.978233
H	-3.050184	3.222206	-3.549326
C	0.767274	3.693906	-1.988051
H	-0.471578	4.786751	-0.456149
C	0.517491	3.131810	-3.405658
H	-1.053123	2.991608	-5.041282
H	1.559078	4.451748	-1.879298
Co	0.722416	-0.507561	0.381515
C	1.383563	1.360493	2.599823
C	1.021105	3.269752	1.665366
H	2.044462	0.533225	2.893293
H	1.352000	4.140843	1.084592
O	2.105350	2.336374	1.828020

C	0.806928	2.224897	3.716194
C	0.592000	3.482613	3.109387
C	0.420042	1.984894	5.034057
C	-0.001670	4.527175	3.812126
C	-0.154100	3.049541	5.758335
H	0.563272	1.004801	5.502243
C	-0.359021	4.302129	5.158829
H	-0.182422	5.501362	3.344199
H	-0.440413	2.899385	6.805056
H	-0.802065	5.114907	5.744510
C	-0.127541	2.388296	1.039137
H	-1.083370	2.786596	1.425639
C	0.167141	1.034416	1.688590
H	-0.684814	0.589601	2.216345

Int3-*ent2*

Lowest frequency = 14.0838 cm^{-1}

Charge = 0, Multiplicity = 1

123

C -2.191132 -0.961620 -1.591634

C	-0.317959	-2.513068	-1.227651
C	-3.132495	-2.077768	-2.132702
C	-1.043234	-3.614773	-1.957758
C	-2.556084	-3.509525	-2.242103
H	-0.725817	-4.583778	-1.529230
H	-3.173888	-4.345825	-1.873035
N	-2.003004	1.036540	-0.286438
C	-1.368638	2.671179	1.273651
C	0.794330	2.326786	2.096264
C	-1.548973	3.854655	2.054592
C	-2.382726	2.181282	0.387261
C	0.708628	3.497799	2.884075
H	1.684239	1.693141	2.093583
C	-0.453628	4.254275	2.871299
C	-2.793583	4.536029	1.960825
C	-3.602867	2.876787	0.330513
H	1.565312	3.785803	3.500456
H	-0.540515	5.159810	3.481431
C	-3.784750	4.034567	1.122269
H	-2.956016	5.444047	2.549944
H	-4.384523	2.512650	-0.335754

H	-4.745510	4.557925	1.058828
N	-0.217491	1.930270	1.324077
C	-2.820845	0.345968	-1.139798
O	-3.878874	0.776609	-1.620678
O	1.264531	-0.440884	1.023048
C	2.515745	-0.379373	0.650082
C	2.952723	0.271824	-0.554509
C	3.524969	-1.023903	1.462895
C	4.316111	0.253465	-0.948428
C	2.012103	0.995424	-1.380277
C	4.846642	-0.999366	1.019891
C	5.284347	-0.379482	-0.180110
H	4.573744	0.763928	-1.877712
N	0.723756	1.133778	-1.210861
O	2.529550	1.650180	-2.463185
H	5.600765	-1.495735	1.636042
C	0.236378	2.144646	-2.181543
C	1.412759	2.220246	-3.188259
H	-0.685550	1.780102	-2.658375
C	-0.050558	3.486050	-1.530081
H	1.243050	1.614903	-4.096124

C	0.932839	4.128049	-0.754688
C	-1.284283	4.123194	-1.737600
C	0.683174	5.387236	-0.196212
H	1.895111	3.635316	-0.578086
C	-1.534583	5.385285	-1.180813
H	-2.062530	3.614283	-2.316594
C	-0.551648	6.019377	-0.410127
H	1.451341	5.873359	0.414062
H	-2.506971	5.863244	-1.334513
H	-0.751227	6.999768	0.035034
C	-1.011252	-1.389501	-0.704014
C	3.135102	-1.741503	2.770505
C	6.770820	-0.432914	-0.568007
C	7.206504	-1.910748	-0.724470
H	8.277297	-1.973431	-0.992054
H	7.058022	-2.477071	0.211334
H	6.620418	-2.407072	-1.517853
C	7.618039	0.236922	0.541624
H	7.483672	-0.268556	1.513689
H	8.692748	0.197737	0.285615
H	7.328770	1.295063	0.666734

C	7.042060	0.298089	-1.896744
H	6.477463	-0.154633	-2.730635
H	6.768196	1.365832	-1.834998
H	8.116555	0.238364	-2.144462
C	2.177118	-2.916642	2.453093
H	1.274418	-2.545265	1.947945
H	2.674219	-3.653910	1.797661
H	1.875808	-3.428375	3.385304
C	4.364616	-2.323186	3.498148
H	4.886963	-3.080378	2.886962
H	5.090338	-1.537087	3.772582
H	4.034394	-2.815876	4.429364
C	2.444587	-0.750484	3.740487
H	3.122205	0.087576	3.983986
H	1.525596	-0.344234	3.294390
H	2.181693	-1.264375	4.683047
H	1.677930	3.249383	-3.470943
C	-4.640302	-1.897058	-2.452020
H	-5.148194	-1.113060	-1.876192
H	-5.199983	-2.845403	-2.369716
C	1.172443	-2.737328	-1.279987

H	1.716024	-2.026657	-0.651914
H	1.438530	-3.766544	-0.978201
C	-4.252372	-1.499909	-3.920446
H	-4.423125	-0.431501	-4.127452
H	-4.701178	-2.101798	-4.729948
C	1.532978	-2.492993	-2.821027
H	2.192041	-1.612730	-2.888411
H	2.065419	-3.364559	-3.239980
C	-2.766458	-1.811664	-3.601389
C	-2.235525	-3.255548	-3.774155
C	-1.761557	-0.707455	-3.236272
C	-0.717193	-3.425236	-3.492366
H	-2.649092	-3.857875	-4.598948
C	-0.325201	-0.971617	-3.462029
H	-2.090961	0.301622	-3.526254
C	0.194013	-2.224879	-3.500072
H	-0.266791	-4.306308	-3.979571
H	0.366308	-0.155916	-3.243178
Co	-0.339864	0.299916	0.258608
C	-2.777704	-2.301033	1.057676
C	-2.733703	-0.434514	2.159111

H	-3.182136	-2.967750	0.286378
H	-3.118696	0.572583	2.363517
O	-3.469392	-1.039901	1.061858
C	-1.296254	-1.828192	0.890745
H	-0.567106	-2.601615	1.159939
C	-1.255341	-0.598234	1.737939
H	-0.546525	-0.608978	2.575799
C	-2.902400	-1.507812	3.232194
C	-2.943609	-1.500248	4.624191
C	-2.906515	-2.724040	2.513194
C	-3.021859	-2.740925	5.291564
H	-2.924840	-0.563095	5.190958
C	-2.953580	-3.951960	3.167201
C	-3.027033	-3.947879	4.576192
H	-3.082396	-2.763031	6.385141
H	-2.945437	-4.897018	2.612729
H	-3.091681	-4.897385	5.118310

Int3-*ent2*

Lowest frequency = 12.4017 cm^{-1}

Charge = 0, Multiplicity = 3

123

C	-2.321388	-0.650132	-1.596462
C	-0.493550	-2.291541	-1.582051
C	-3.382983	-1.716865	-2.046669
C	-1.418041	-3.507989	-1.940140
C	-2.923354	-3.198873	-2.170040
H	-1.190884	-4.452175	-1.417908
H	-3.627512	-3.967674	-1.807275
N	-1.878330	1.218814	-0.184306
C	-1.093034	2.671802	1.493753
C	1.043542	2.096862	2.243413
C	-1.162801	3.810403	2.358138
C	-2.153097	2.328105	0.594926
C	1.079716	3.224645	3.095541
H	1.878538	1.394665	2.183719
C	-0.019224	4.068453	3.163750
C	-2.351841	4.588526	2.350228
C	-3.319400	3.113003	0.631332
H	1.972449	3.405351	3.701422

H	-0.018567	4.936146	3.832461
C	-3.397474	4.217505	1.511015
H	-2.429949	5.463031	3.003670
H	-4.142741	2.861363	-0.035558
H	-4.320169	4.808621	1.513121
N	-0.015675	1.834073	1.478543
C	-2.812713	0.665706	-1.033204
O	-3.845474	1.228547	-1.414412
O	1.290371	-0.606337	0.850073
C	2.552868	-0.565498	0.501506
C	3.018976	0.134415	-0.660339
C	3.516055	-1.291066	1.296128
C	4.387106	0.112424	-1.025526
C	2.093290	0.890184	-1.470475
C	4.848204	-1.263653	0.886575
C	5.323075	-0.578128	-0.263728
H	4.676624	0.660918	-1.923013
N	0.812178	1.068654	-1.263698
O	2.605418	1.534705	-2.553730
H	5.579451	-1.812089	1.485073
C	0.341058	2.109588	-2.209082

C	1.486853	2.146596	-3.247701
H	-0.606485	1.788885	-2.662199
C	0.137236	3.435142	-1.496128
H	1.269607	1.539101	-4.141870
C	1.159324	3.979705	-0.695316
C	-1.066083	4.142261	-1.644726
C	0.977065	5.209435	-0.052550
H	2.098217	3.431934	-0.559902
C	-1.248407	5.375956	-1.004316
H	-1.879421	3.705821	-2.234734
C	-0.229381	5.910593	-0.205746
H	1.773779	5.616845	0.578370
H	-2.199551	5.906653	-1.110563
H	-0.379057	6.865898	0.307889
C	-1.228655	-1.272305	-0.737424
C	3.072619	-2.089050	2.538558
C	6.818314	-0.631348	-0.613926
C	7.236897	-2.105053	-0.841882
H	8.313048	-2.165743	-1.085974
H	7.058818	-2.720782	0.056620
H	6.665426	-2.547922	-1.676152

C	7.639533	-0.038048	0.557504
H	7.472350	-0.597458	1.494180
H	8.719942	-0.077094	0.328331
H	7.360101	1.015078	0.735775
C	7.137394	0.170319	-1.890242
H	6.593293	-0.226471	-2.765046
H	6.876198	1.236843	-1.775398
H	8.217865	0.109053	-2.108739
C	2.066762	-3.194542	2.126441
H	1.168418	-2.758814	1.666727
H	2.530312	-3.890613	1.405532
H	1.760472	-3.772328	3.017139
C	4.268264	-2.777967	3.228595
H	4.768848	-3.501641	2.561337
H	5.020007	-2.048040	3.578199
H	3.904784	-3.333050	4.110631
C	2.414557	-1.145787	3.576345
H	3.121014	-0.354962	3.886906
H	1.511917	-0.673174	3.163437
H	2.125882	-1.719692	4.475318
H	1.784394	3.163794	-3.540452

C	-4.884780	-1.407043	-2.279002
H	-5.270660	-0.555380	-1.702066
H	-5.533490	-2.287254	-2.125379
C	0.976863	-2.767544	-1.431784
H	1.555962	-2.180623	-0.716354
H	1.072630	-3.833875	-1.165217
C	-4.558029	-1.086474	-3.781593
H	-4.686611	-0.021178	-4.034699
H	-5.082731	-1.699098	-4.535755
C	1.273607	-2.423451	-2.920181
H	1.960038	-1.567482	-3.029657
H	1.636425	-3.250914	-3.553845
C	-3.078550	-1.475173	-3.527637
C	-2.626795	-2.949136	-3.706676
C	-1.994752	-0.386536	-3.194147
C	-1.120946	-3.265259	-3.457959
H	-3.104552	-3.526165	-4.516640
C	-0.636120	-0.731465	-3.644102
H	-2.330224	0.620233	-3.489298
C	-0.203936	-2.027501	-3.126739
H	-0.669760	-4.029611	-4.113105

H	-0.112380	-0.186211	-4.437847
Co	-0.241410	0.277103	0.214489
C	-3.162271	-2.017956	0.980998
C	-2.541142	-0.361313	2.235162
H	-3.827118	-2.467734	0.234886
H	-2.650102	0.667600	2.601530
O	-3.527013	-0.639826	1.212160
C	-1.645214	-1.887309	0.620319
H	-1.116220	-2.842244	0.777429
C	-1.200712	-0.775226	1.582622
H	-0.430807	-1.047546	2.317444
C	-2.807467	-1.499926	3.211912
C	-2.647394	-1.663380	4.587158
C	-3.200162	-2.580815	2.391142
C	-2.925873	-2.929557	5.140431
H	-2.325428	-0.834142	5.226760
C	-3.444783	-3.841939	2.929819
C	-3.318673	-4.002879	4.324968
H	-2.838945	-3.078462	6.222356
H	-3.736776	-4.686818	2.296053
H	-3.533159	-4.976048	4.779926

Int3-*ent2*

Lowest frequency = 12.8836 cm^{-1}

Charge = 0, Multiplicity = 5

123

C	-2.278148	-0.883598	-1.612361
C	-0.347971	-2.409561	-1.409621
C	-3.229935	-2.055112	-2.058165
C	-1.150911	-3.710252	-1.748423
C	-2.660352	-3.508416	-2.069583
H	-0.892318	-4.611747	-1.168607
H	-3.334786	-4.301250	-1.702398
N	-2.057271	1.135547	-0.334588
C	-1.487219	2.722562	1.307480
C	0.606494	2.360411	2.272761
C	-1.713581	3.895064	2.095734
C	-2.463432	2.250564	0.360596
C	0.490686	3.533103	3.051542
H	1.484017	1.711040	2.326921

C	-0.668713	4.291983	2.973731
C	-2.955964	4.576240	1.958508
C	-3.691074	2.934494	0.285877
H	1.310241	3.814433	3.719289
H	-0.792504	5.193828	3.583229
C	-3.915372	4.079839	1.083085
H	-3.141986	5.478061	2.550567
H	-4.444729	2.574910	-0.414154
H	-4.876179	4.598097	0.989938
N	-0.355853	1.970431	1.434963
C	-2.898149	0.432464	-1.180192
O	-3.954507	0.860488	-1.657820
O	1.479169	-0.252219	1.027104
C	2.723387	-0.287488	0.650230
C	3.150840	0.278179	-0.602332
C	3.723241	-0.929130	1.476285
C	4.509391	0.229065	-1.006707
C	2.193323	0.941134	-1.451022
C	5.038346	-0.944996	1.018869
C	5.473057	-0.376569	-0.210793
H	4.766583	0.681836	-1.965568

N	0.892269	1.035937	-1.292710
O	2.686569	1.584165	-2.545961
H	5.791805	-1.436160	1.640121
C	0.377149	2.001794	-2.291763
C	1.555872	2.098839	-3.290555
H	-0.521221	1.586140	-2.769102
C	0.024463	3.330226	-1.644223
H	1.413521	1.462481	-4.180841
C	0.933278	3.972587	-0.782324
C	-1.206836	3.945249	-1.921059
C	0.612931	5.208403	-0.208315
H	1.889861	3.494957	-0.544378
C	-1.528755	5.183651	-1.348184
H	-1.933030	3.434315	-2.562878
C	-0.620276	5.816961	-0.490416
H	1.322670	5.693193	0.469896
H	-2.500823	5.641659	-1.555577
H	-0.877406	6.776975	-0.030909
C	-1.200862	-1.404868	-0.679072
C	3.318006	-1.616513	2.794142
C	6.956418	-0.465677	-0.603053

C	7.373040	-1.954539	-0.695987
H	8.440758	-2.040182	-0.968720
H	7.226922	-2.476882	0.265326
H	6.775078	-2.479073	-1.461608
C	7.815156	0.242082	0.473974
H	7.678120	-0.218162	1.467840
H	8.888015	0.177332	0.216063
H	7.540299	1.308490	0.552699
C	7.230837	0.203297	-1.963419
H	6.660187	-0.280412	-2.775390
H	6.968164	1.275538	-1.948121
H	8.304011	0.121670	-2.210159
C	2.327306	-2.769720	2.490404
H	1.428114	-2.390902	1.982594
H	2.802579	-3.527173	1.842688
H	2.019011	-3.261952	3.430775
C	4.535844	-2.220324	3.523034
H	5.038011	-2.994119	2.915939
H	5.280559	-1.448800	3.788431
H	4.198151	-2.698018	4.459247
C	2.652044	-0.600123	3.754929

H	3.334179	0.243660	3.963862
H	1.722072	-0.206643	3.320542
H	2.410273	-1.091933	4.714702
H	1.790090	3.128608	-3.597297
C	-4.735042	-1.884113	-2.397572
H	-5.236332	-1.049369	-1.888874
H	-5.312108	-2.813229	-2.245133
C	1.146978	-2.707095	-1.131274
H	1.591618	-1.966363	-0.460602
H	1.348035	-3.715204	-0.730760
C	-4.336577	-1.602573	-3.889970
H	-4.521583	-0.559870	-4.195771
H	-4.769365	-2.280379	-4.646639
C	1.506925	-2.483320	-2.629198
H	2.153924	-1.605470	-2.790119
H	1.946693	-3.346684	-3.157353
C	-2.852214	-1.871718	-3.529517
C	-2.296774	-3.319478	-3.599971
C	-1.864644	-0.694620	-3.218692
C	-0.789573	-3.519233	-3.259474
H	-2.691170	-3.969476	-4.399464

C	-0.460205	-0.969417	-3.553345
H	-2.240928	0.269732	-3.593998
C	0.021940	-2.208160	-2.952725
H	-0.252672	-4.279763	-3.851370
H	0.148778	-0.323817	-4.193692
Co	-0.281577	0.311712	0.184961
C	-3.162473	-2.073694	1.045354
C	-2.500188	-0.392664	2.248421
H	-3.838588	-2.526424	0.310627
H	-2.588711	0.646160	2.591037
O	-3.499260	-0.680494	1.245657
C	-1.645561	-1.987161	0.680026
H	-1.141943	-2.949383	0.874257
C	-1.179066	-0.845002	1.587388
H	-0.355120	-1.056278	2.281296
C	-2.766816	-1.504156	3.255838
C	-2.585085	-1.635087	4.632340
C	-3.198431	-2.596836	2.469584
C	-2.882862	-2.879261	5.222918
H	-2.232592	-0.797225	5.243865
C	-3.461508	-3.837448	3.046106

C	-3.315150	-3.964000	4.442599
H	-2.779784	-3.002313	6.306593
H	-3.783013	-4.691769	2.440071
H	-3.544035	-4.919472	4.926940

NMR Spectra

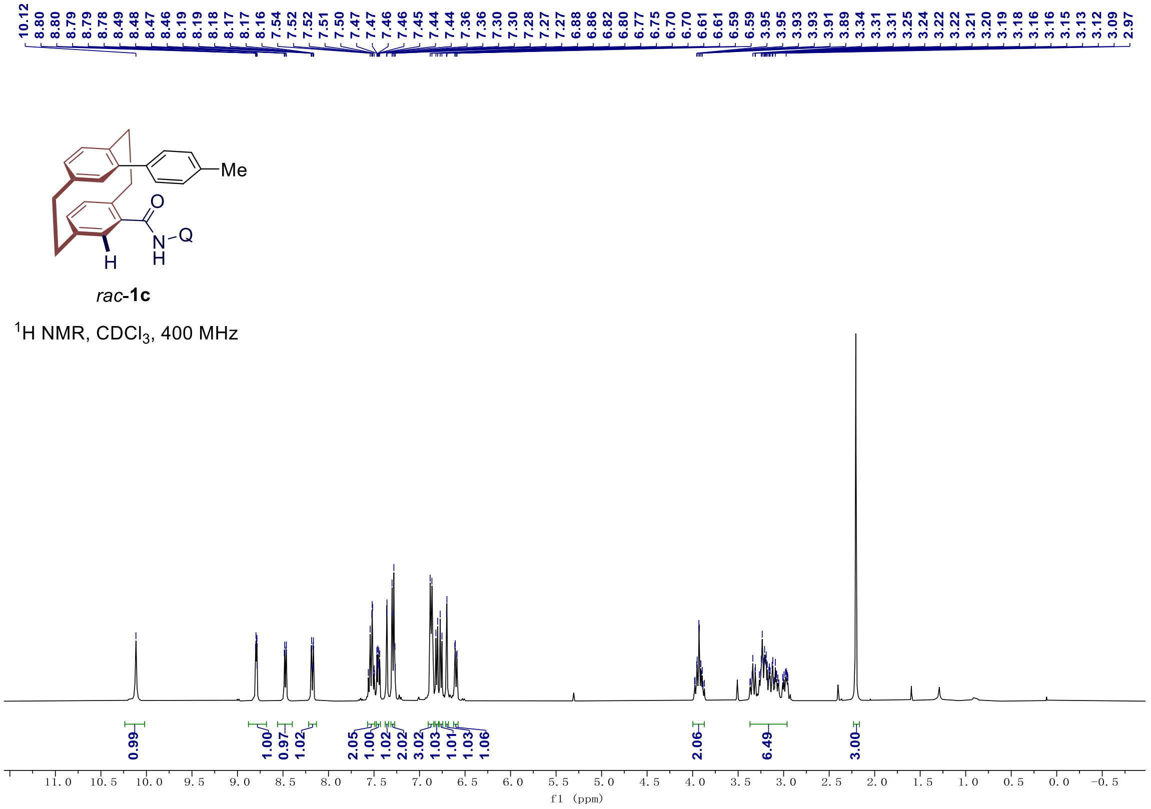

10.12 8.80 8.80 8.79 8.78 8.49 8.48 8.47 8.46 8.19 8.19 8.18 8.17 8.17 8.16 7.54 7.52 7.52 7.51 7.50 7.47 7.46 7.46 7.45 7.44 7.44 7.36 7.36 7.30 7.28 7.27 7.27 6.88 6.86 6.82 6.80 6.77 6.75 6.70 6.70 6.61 6.61 6.59 6.59 3.95 3.95 3.93 3.93 3.91 3.89 3.34 3.31 3.31 3.25 3.24 3.22 3.22 3.21 3.20 3.19 3.18 3.16 3.16 3.15 3.13 3.12 3.09 2.97
Me
O
N
Q
H
H
rac-1c
^{1}H NMR, $CDCl_3$, 400 MHz
0.99 1.00 0.97 1.02 2.05 1.00 1.02 2.02 3.02 1.03 1.01 1.03 1.06 2.06 6.49 3.00
11.0 10.5 10.0 9.5 9.0 8.5 8.0 7.5 7.0 6.5 6.0 5.5 5.0 4.5 4.0 3.5 3.0 2.5 2.0 1.5 1.0 0.5 0.0 -0.5
f1 (ppm)

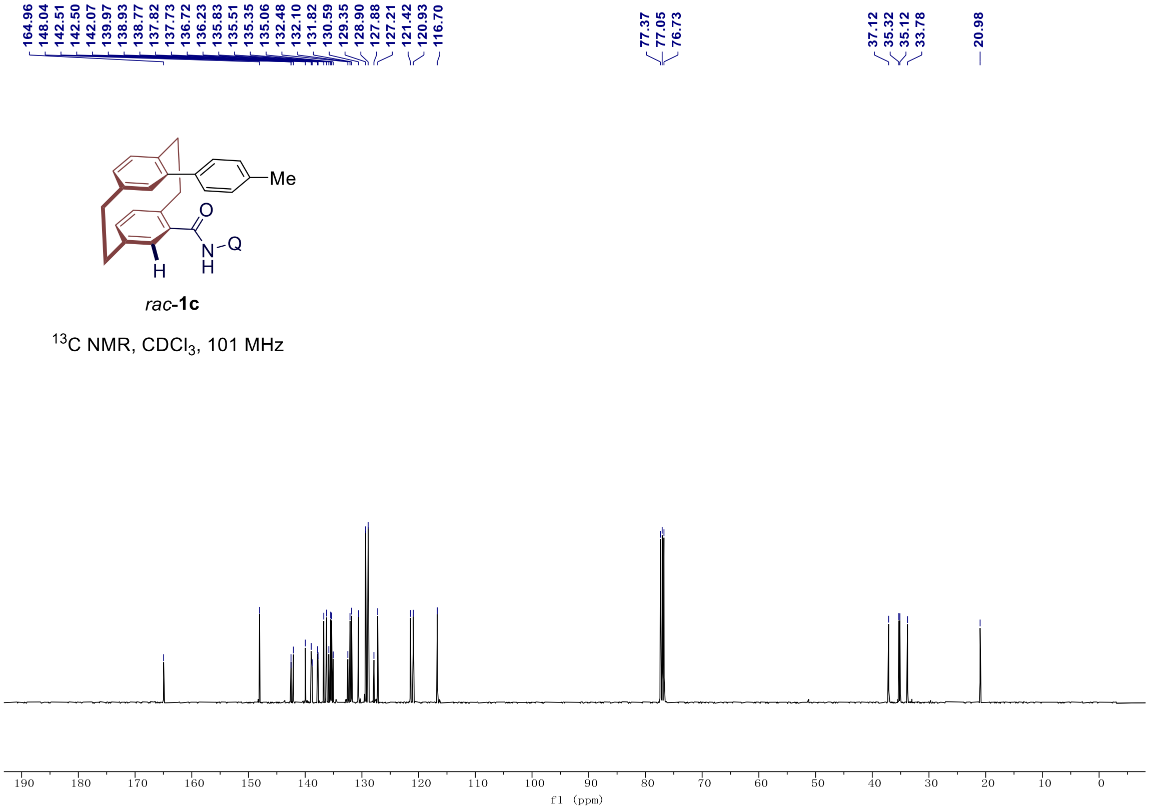

164.96 148.04 142.51 142.50 142.07 139.97 138.93 138.77 137.82 137.73 136.72 136.23 135.83 135.51 135.35 135.06 132.48 132.10 131.82 130.59 129.35 128.90 127.88 127.21 121.42 120.93 116.70
77.37 77.05 76.73
37.12 35.32 35.12 33.78
20.98
Me
O
N
Q
H
H
rac-1c
^{13}C NMR, $CDCl_3$, 101 MHz
190 180 170 160 150 140 130 120 110 100 90 80 70 60 50 40 30 20 10 0
f1 (ppm)

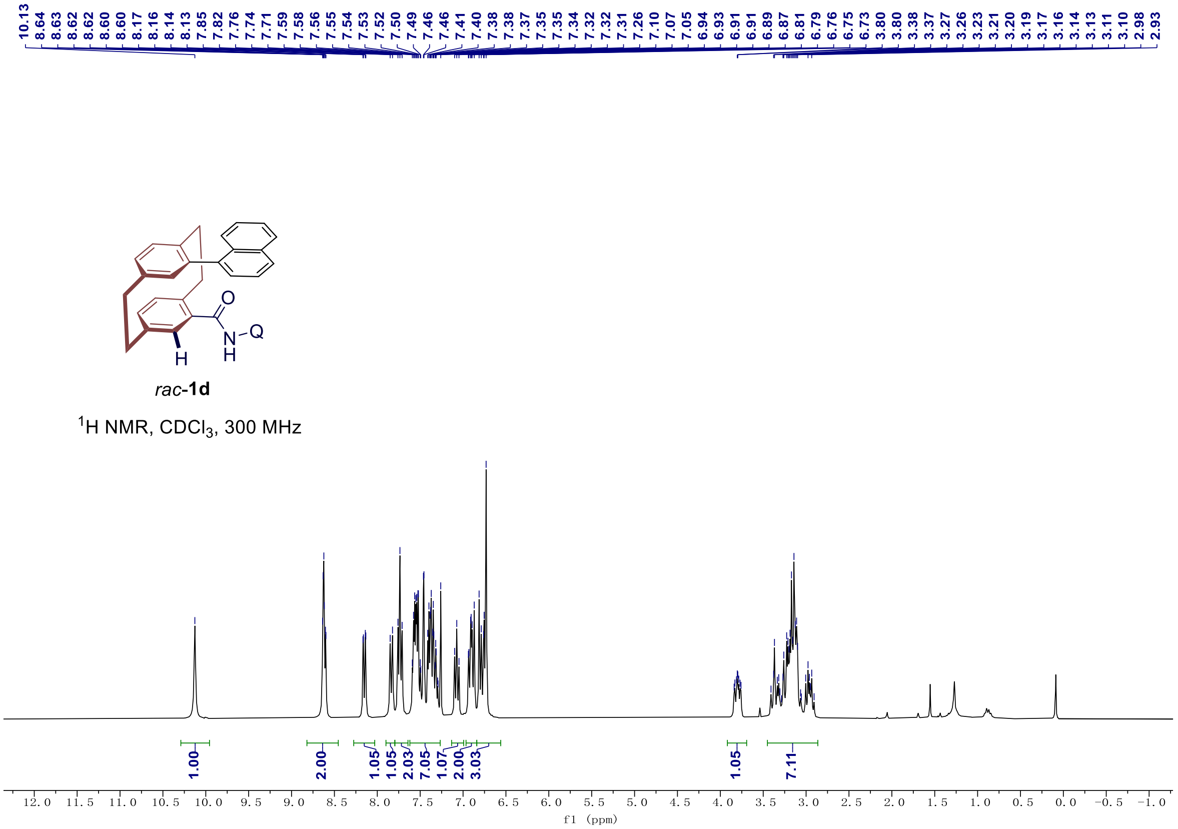
10.13 8.64 8.63 8.62 8.62 8.60 8.60 8.17 8.16 8.14 8.13 7.85 7.82 7.76 7.74 7.71 7.59 7.58 7.56 7.55 7.54 7.53 7.52 7.50 7.49 7.46 7.46 7.41 7.40 7.38 7.38 7.37 7.35 7.35 7.34 7.32 7.32 7.31 7.26 7.10 7.07 7.05 6.94 6.93 6.91 6.91 6.89 6.87 6.81 6.79 6.76 6.75 6.73 3.80 3.80 3.38 3.37 3.27 3.26 3.23 3.21 3.20 3.19 3.17 3.16 3.14 3.13 3.11 3.10 2.98 2.93
O
N
Q
H
H
rac-1d
^{1}H NMR, $CDCl_3$, 300 MHz
1.00 2.00 1.05 1.05 2.03 7.05 1.07 2.00 3.03 1.05 7.11
f1 (ppm)

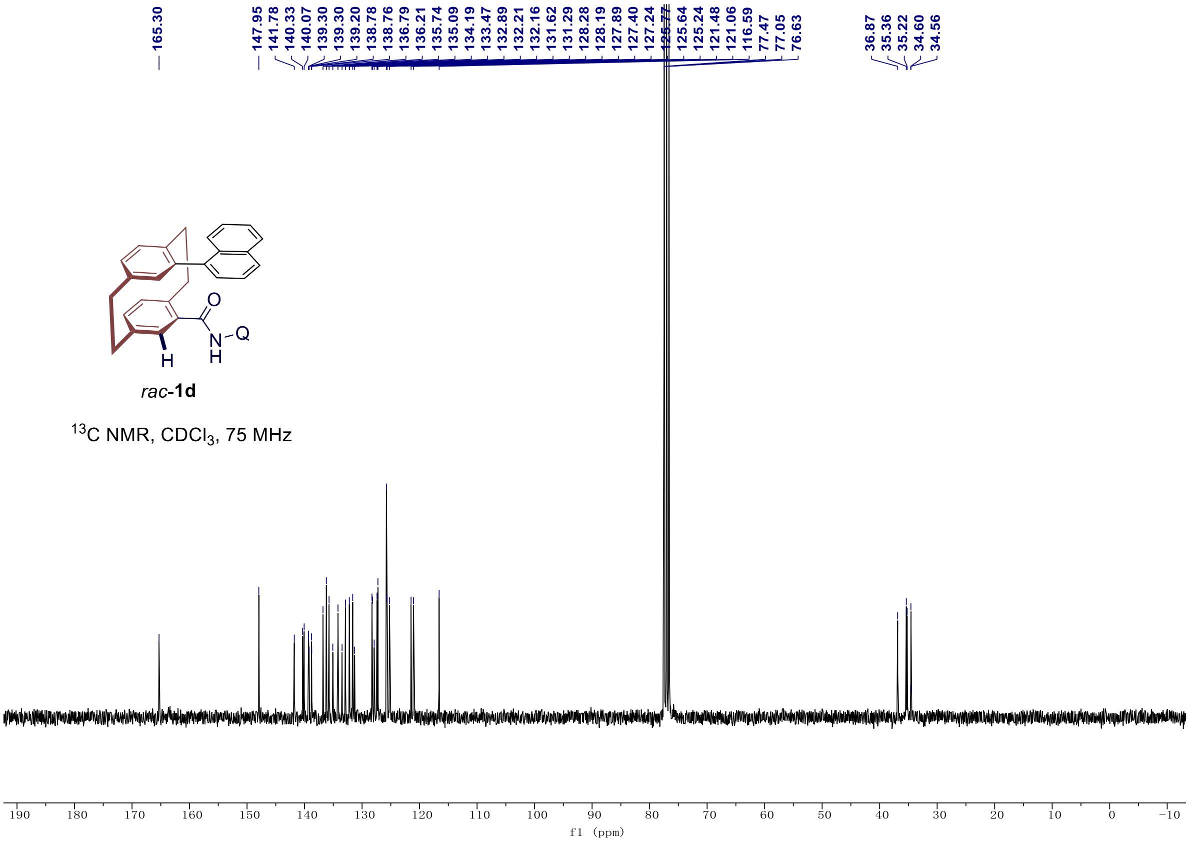
165.30 147.95 141.78 140.33 140.07 139.30 139.30 139.20 138.78 138.76 136.79 136.21 135.74 135.09 134.19 133.47 132.89 132.21 132.16 131.62 131.29 128.28 128.19 127.89 127.40 127.24 125.77 125.64 125.24 121.48 121.06 116.59 77.47 77.05 76.63 36.87 35.36 35.22 34.60 34.56
O
N
Q
H
H
rac-1d
^{13}C NMR, $CDCl_3$, 75 MHz
f1 (ppm)

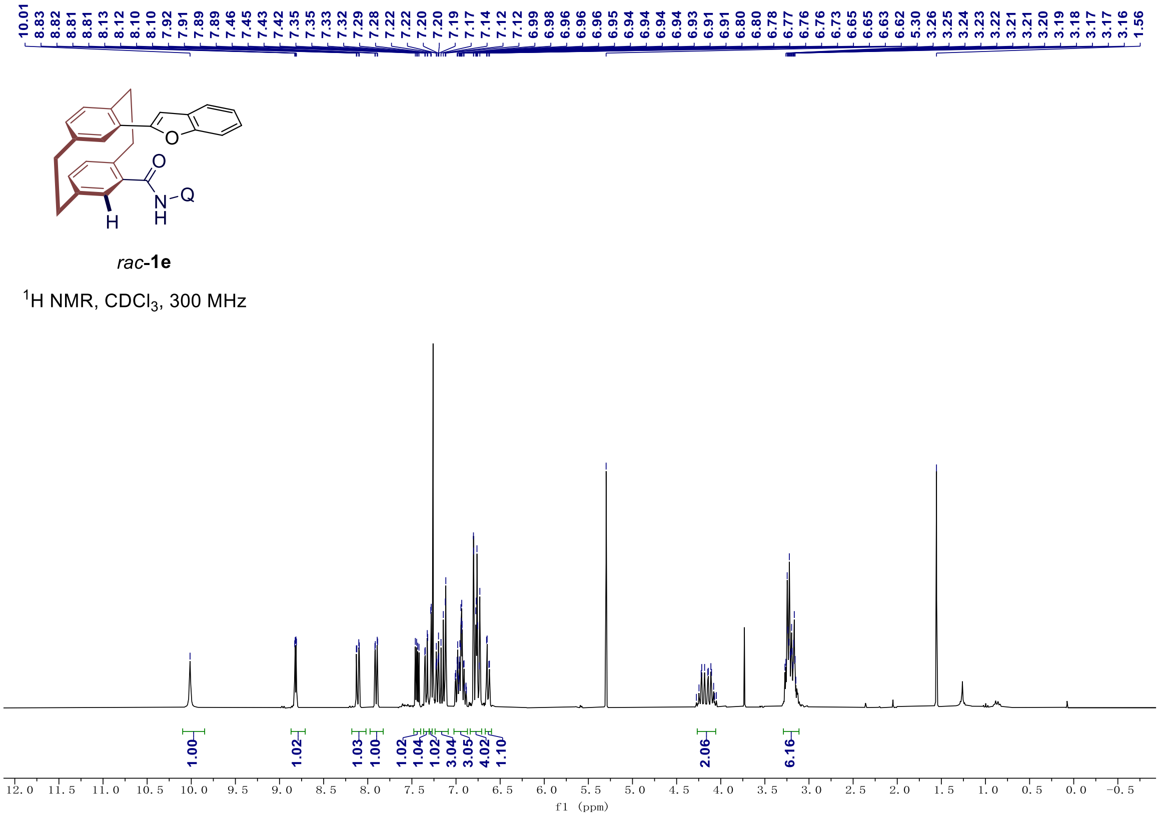
10.01 8.83 8.82 8.81 8.81 8.13 8.12 8.10 8.10 7.92 7.91 7.89 7.89 7.46 7.45 7.43 7.42 7.35 7.35 7.33 7.32 7.29 7.28 7.22 7.22 7.20 7.20 7.19 7.17 7.14 7.12 7.12 6.99 6.98 6.96 6.96 6.96 6.95 6.94 6.94 6.94 6.93 6.91 6.91 6.80 6.78 6.77 6.76 6.76 6.73 6.65 6.63 6.62 5.30 3.26 3.25 3.24 3.23 3.22 3.21 3.21 3.20 3.19 3.18 3.17 3.16 1.56
O
N-Q
H
H
rac-1e
1H NMR, CDCl3, 300 MHz
1.00 1.02 1.03 1.00 1.02 1.04 1.02 3.04 3.05 4.02 1.10 2.06 6.16
12.0 11.5 11.0 10.5 10.0 9.5 9.0 8.5 8.0 7.5 7.0 6.5 6.0 5.5 5.0 4.5 4.0 3.5 3.0 2.5 2.0 1.5 1.0 0.5 0.0 -0.5
f1 (ppm)

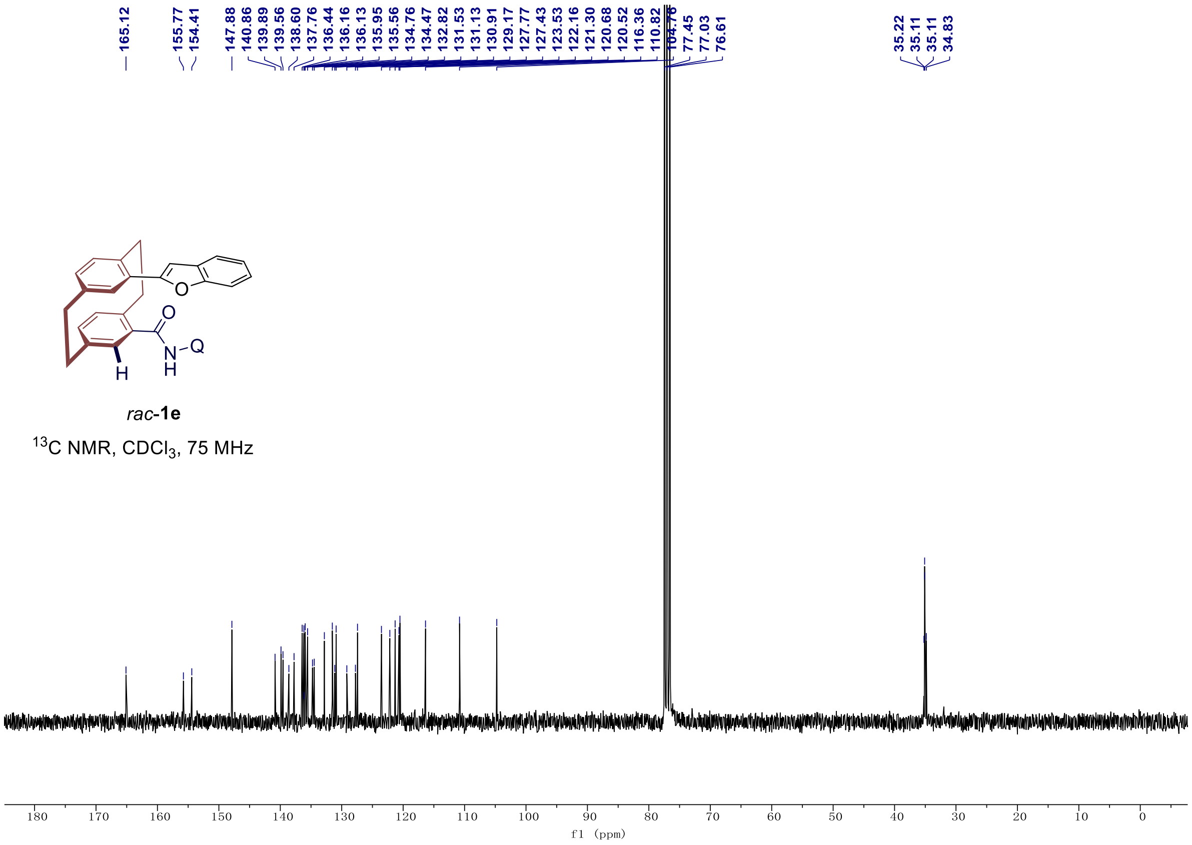
165.12 155.77 154.41 147.88 140.86 139.89 139.56 138.60 137.76 136.44 136.16 136.13 135.95 135.56 134.76 134.47 132.82 131.53 131.13 130.91 129.17 127.77 127.43 123.53 122.16 121.30 120.68 120.52 116.36 110.82 104.76 77.45 77.03 76.61 35.22 35.11 35.11 34.83
O
N-Q
H
H
rac-1e
13C NMR, CDCl3, 75 MHz
180 170 160 150 140 130 120 110 100 90 80 70 60 50 40 30 20 10 0
f1 (ppm)

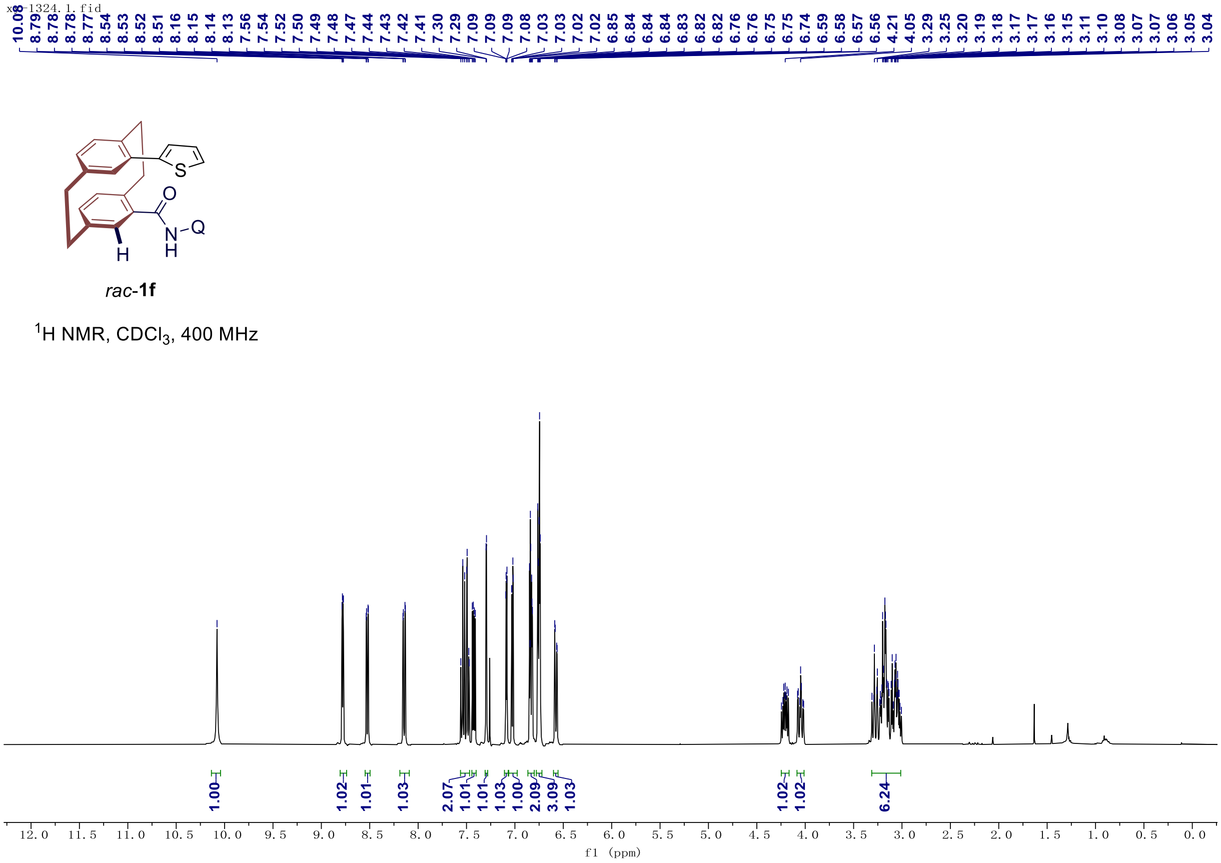
xy-1324.1.fid
10.08 8.79 8.78 8.78 8.77 8.54 8.53 8.52 8.51 8.16 8.15 8.14 8.13 7.56 7.54 7.52 7.50 7.49 7.48 7.47 7.44 7.43 7.42 7.41 7.30 7.29 7.09 7.09 7.09 7.08 7.03 7.03 7.02 6.85 6.84 6.84 6.83 6.82 6.82 6.76 6.76 6.75 6.74 6.59 6.58 6.57 6.56 4.21 4.05 3.29 3.25 3.20 3.19 3.18 3.17 3.17 3.16 3.15 3.11 3.10 3.08 3.07 3.06 3.05 3.04
S
O
N-Q
H
H
rac-1f
1H NMR, CDCl3, 400 MHz
1.00 1.02 1.01 1.03 2.07 1.01 1.01 1.03 1.00 2.09 3.09 1.03 1.02 1.02 6.24
f1 (ppm)

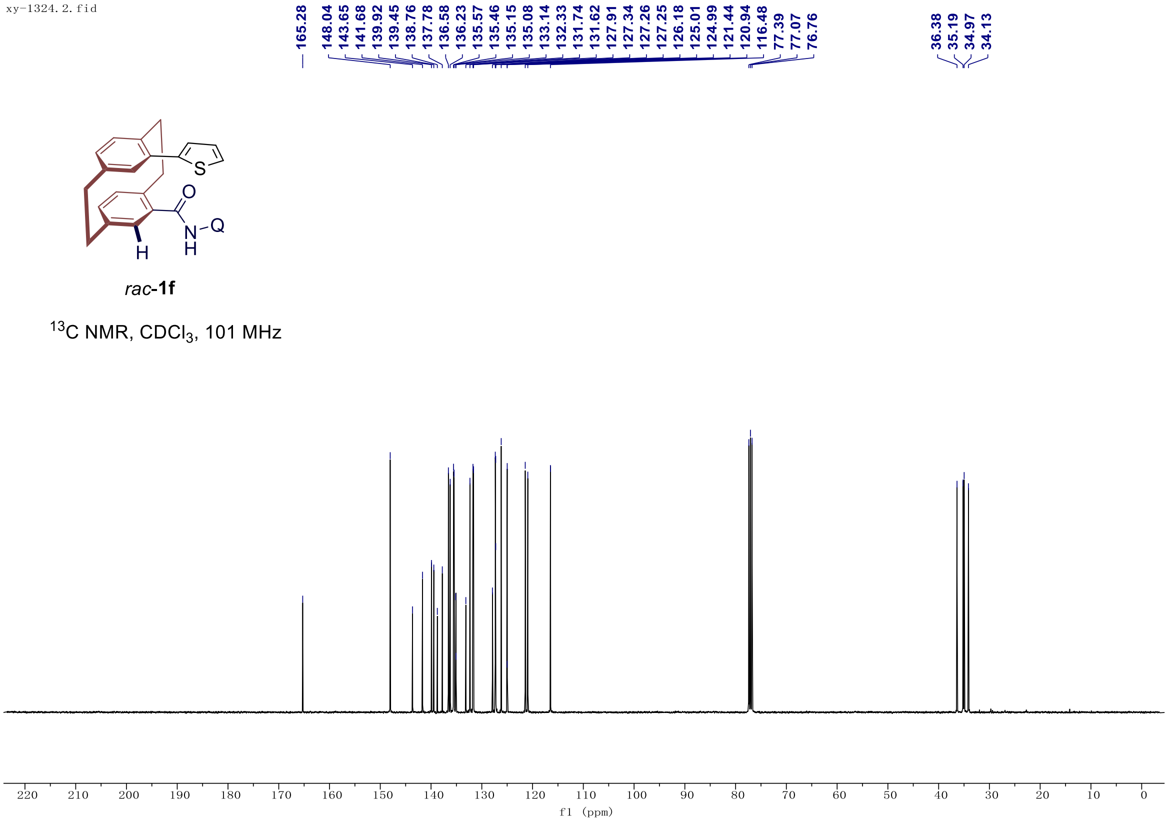
xy-1324.2.fid
165.28 148.04 143.65 141.68 139.92 139.45 138.76 137.78 136.58 136.23 135.57 135.46 135.15 135.08 133.14 132.33 131.74 131.62 127.91 127.34 127.26 127.25 126.18 125.01 124.99 121.44 120.94 116.48 77.39 77.07 76.76 36.38 35.19 34.97 34.13
S
O
N-Q
H
H
rac-1f
13C NMR, CDCl3, 101 MHz
f1 (ppm)

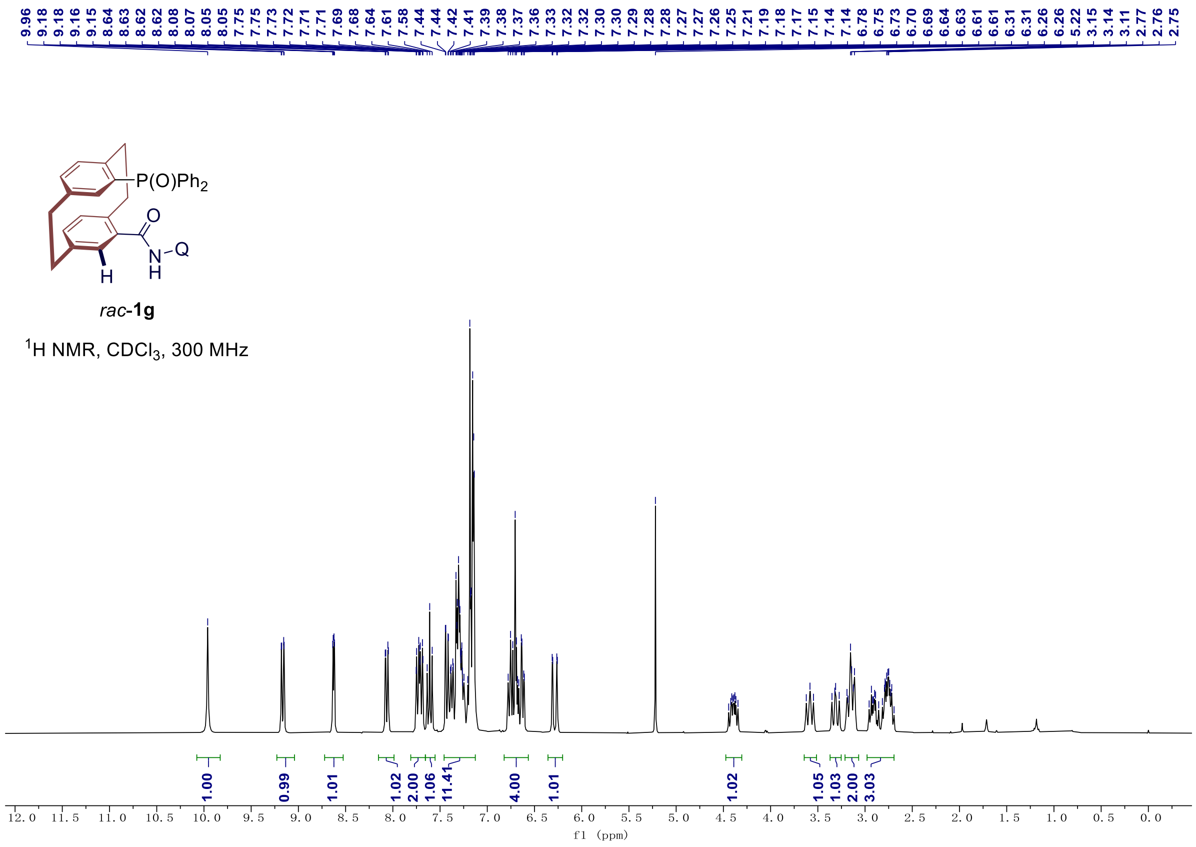
9.96 9.18 9.18 9.16 9.15 8.64 8.63 8.62 8.62 8.08 8.07 8.05 8.05 7.75 7.75 7.73 7.72 7.71 7.71 7.69 7.68 7.64 7.61 7.58 7.44 7.44 7.42 7.41 7.39 7.38 7.37 7.36 7.33 7.32 7.32 7.30 7.30 7.29 7.28 7.28 7.27 7.26 7.25 7.21 7.19 7.18 7.17 7.15 7.14 7.14 6.78 6.75 6.73 6.70 6.69 6.64 6.63 6.61 6.61 6.31 6.31 6.26 6.26 5.22 3.15 3.14 3.11 2.77 2.76 2.75
P(O)Ph2
O
N-Q
H
H
rac-1g
1H NMR, CDCl3, 300 MHz
1.00
0.99
1.01
1.02
2.00
1.06
11.41
4.00
1.01
1.02
1.05
1.03
2.00
3.03
f1 (ppm)

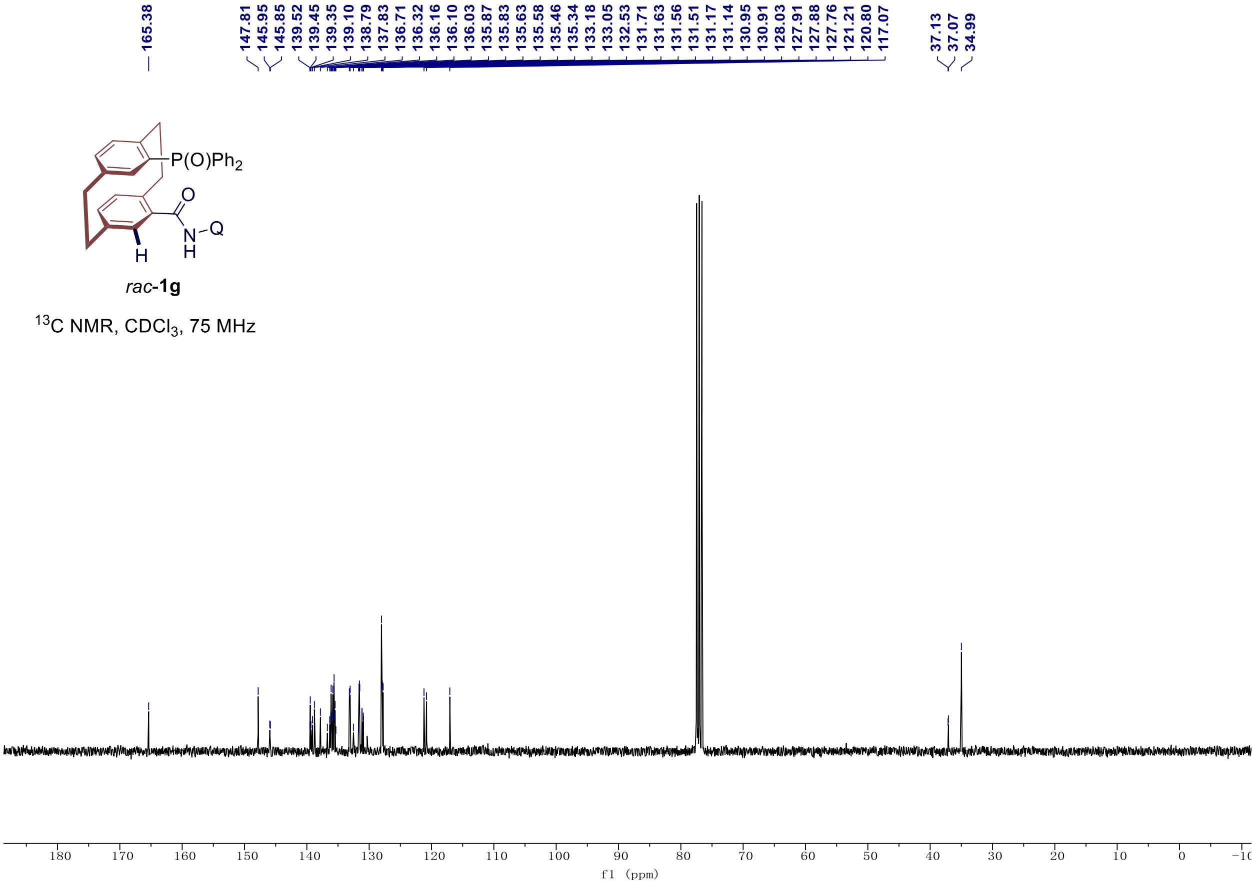
165.38
147.81 145.95 145.85 139.52 139.45 139.35 139.10 138.79 137.83 136.71 136.32 136.16 136.10 136.03 135.87 135.83 135.63 135.58 135.46 135.34 133.18 133.05 132.53 131.71 131.63 131.56 131.51 131.17 131.14 130.95 130.91 128.03 127.91 127.88 127.76 121.21 120.80 117.07
37.13 37.07 34.99
P(O)Ph2
O
N-Q
H
H
rac-1g
13C NMR, CDCl3, 75 MHz
f1 (ppm)

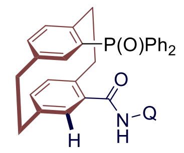

rac-**1g**

^{31}P NMR, $CDCl_3$, 121 MHz

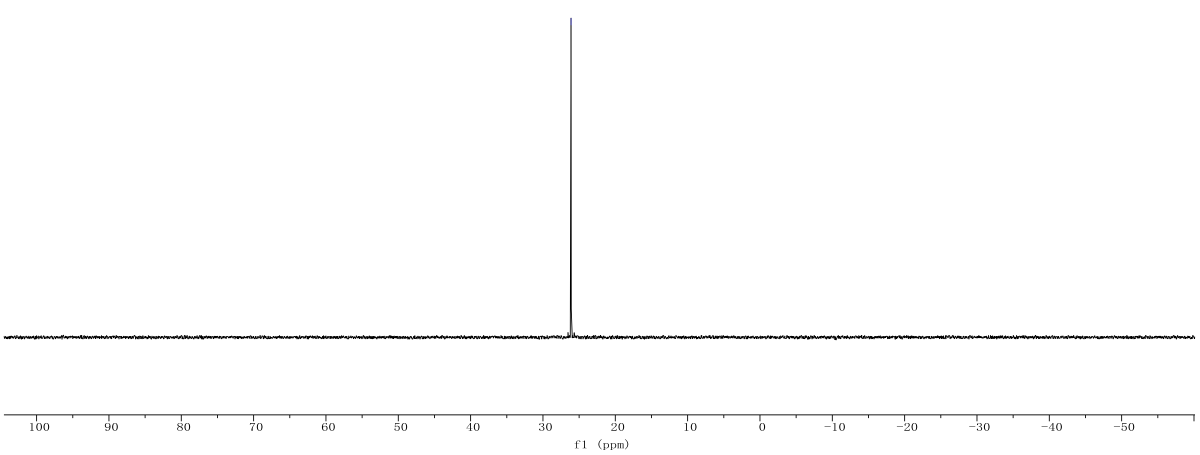

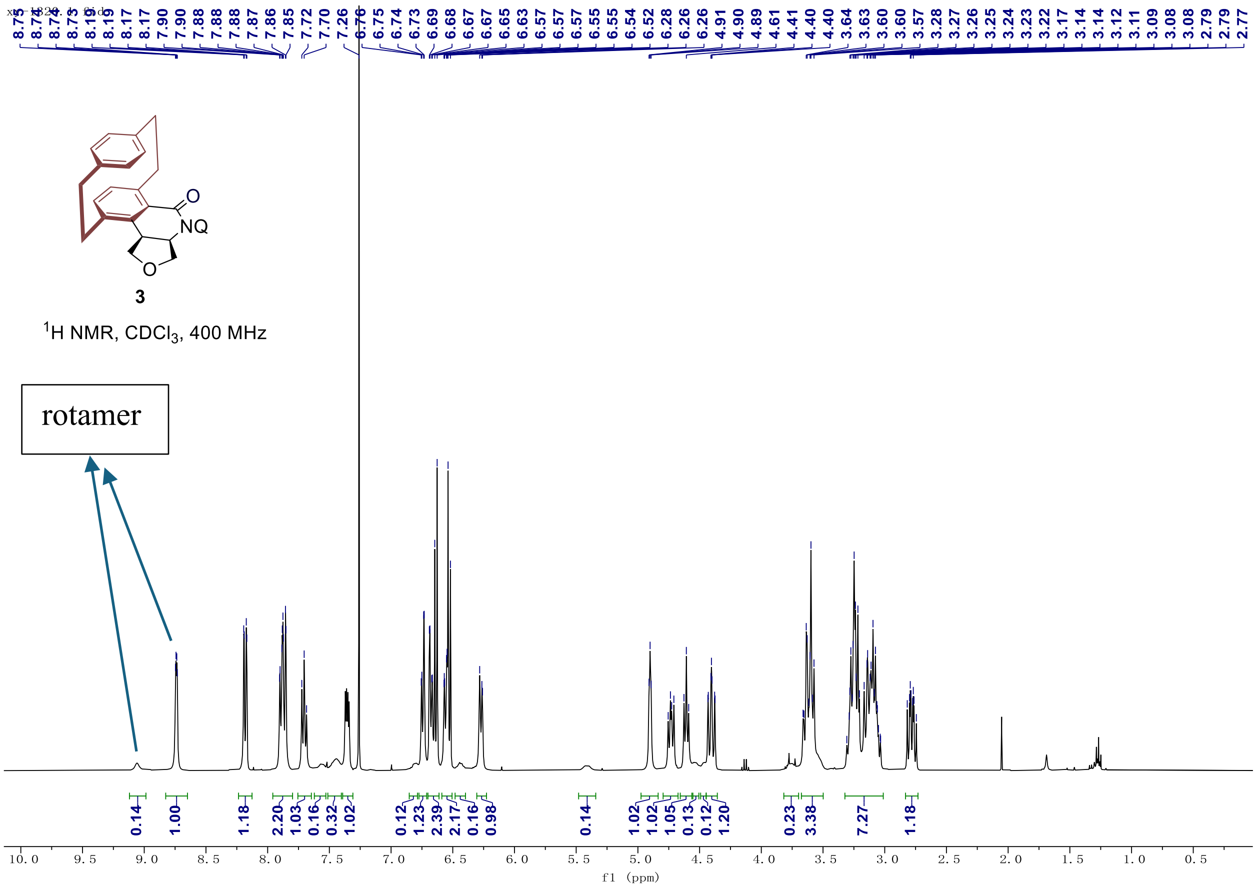

3
^{1}H NMR, $CDCl_3$, 400 MHz
rotamer
f1 (ppm)

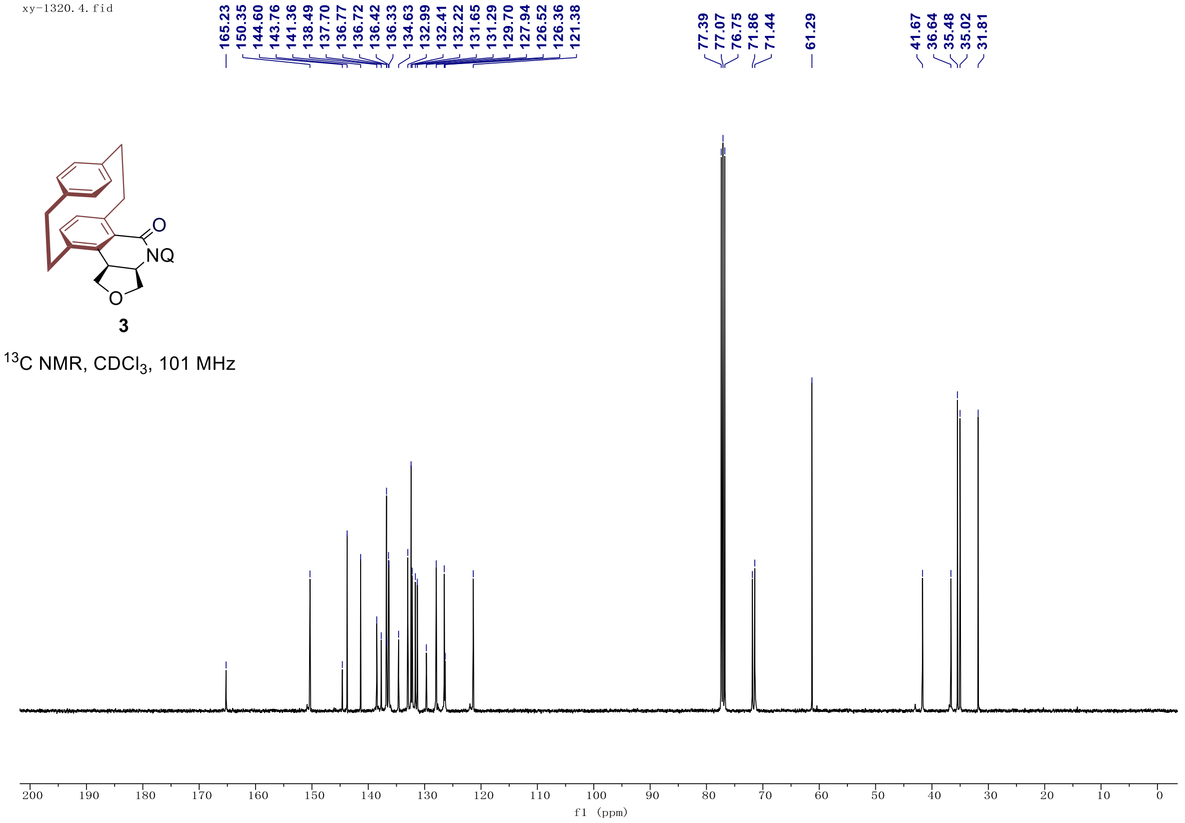

xy-1320.4.fid
3
^{13}C NMR, $CDCl_3$, 101 MHz
f1 (ppm)

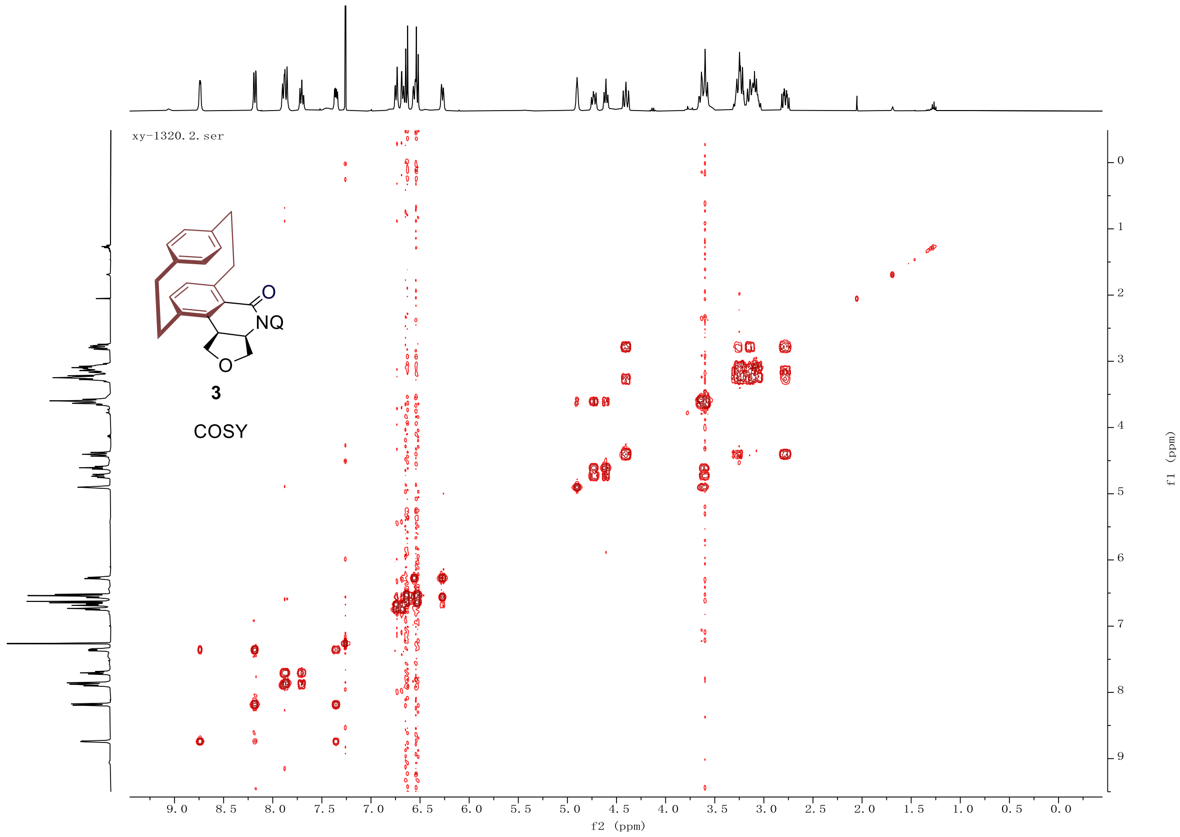
xy-1320. 2. ser
3
COSY
f2 (ppm)
f1 (ppm)

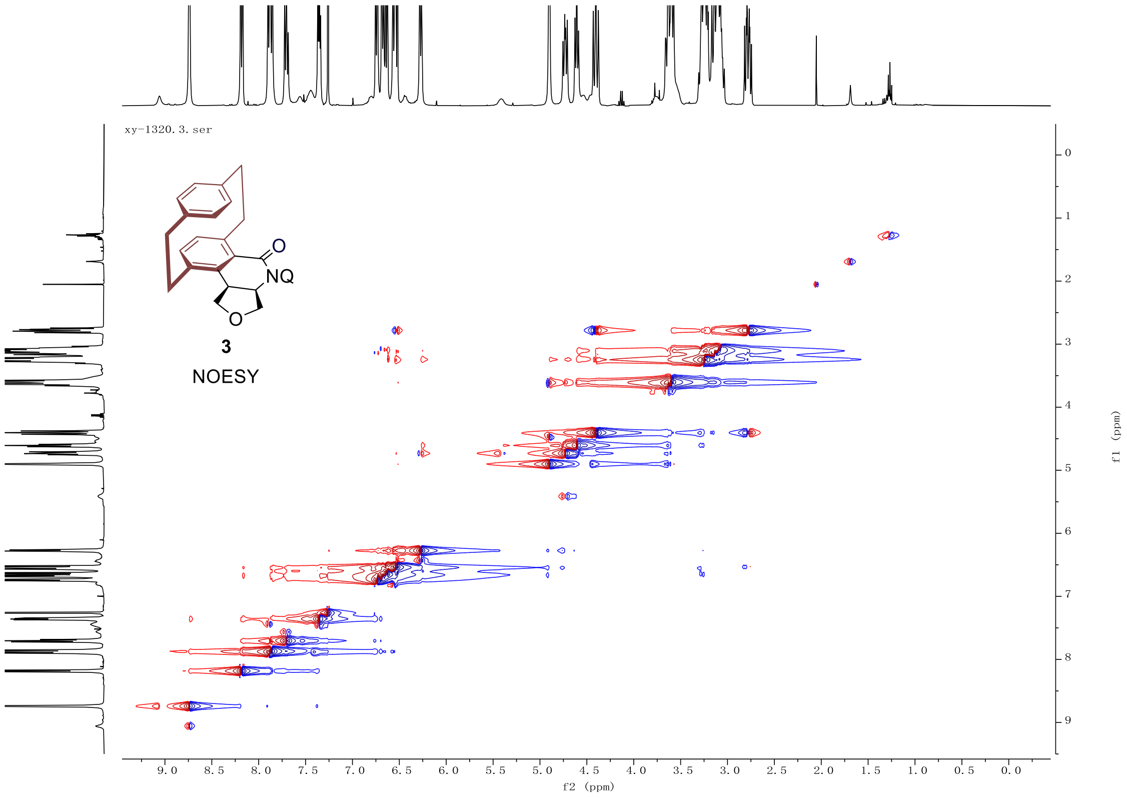
xy-1320. 3. ser
3
NOESY
f2 (ppm)
f1 (ppm)

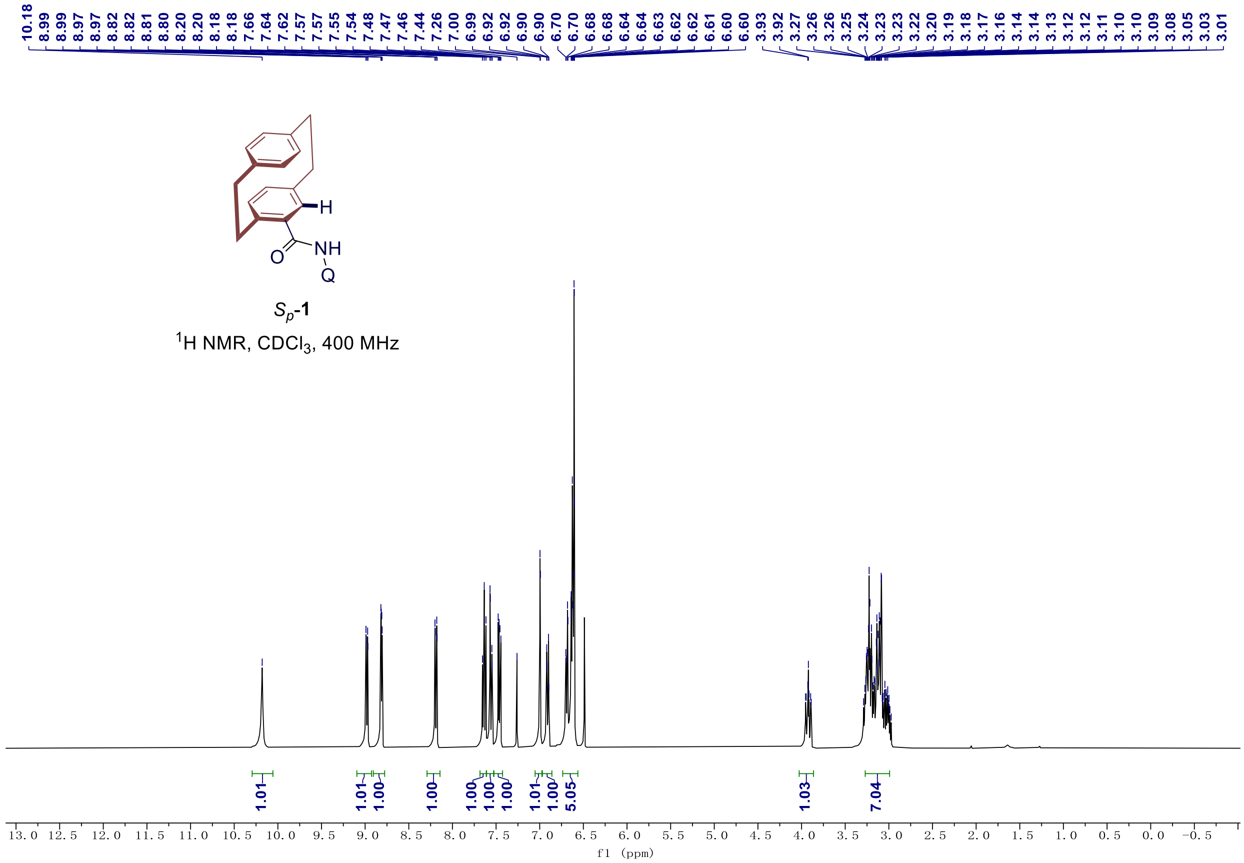
10.18
8.99
8.99
8.97
8.82
8.82
8.81
8.80
8.20
8.20
8.18
8.18
7.66
7.64
7.62
7.57
7.57
7.55
7.54
7.48
7.47
7.46
7.44
7.26
7.00
6.99
6.92
6.92
6.90
6.90
6.70
6.70
6.68
6.64
6.64
6.63
6.62
6.62
6.61
6.60
3.93
3.92
3.27
3.26
3.25
3.24
3.23
3.23
3.22
3.20
3.19
3.18
3.17
3.16
3.14
3.14
3.13
3.12
3.12
3.11
3.10
3.10
3.09
3.08
3.05
3.03
3.01
H
O
NH
Q
S_p-**1**
^{1}H NMR, $CDCl_3$, 400 MHz
1.01
1.01
1.00
1.00
1.00
1.00
1.00
1.01
1.00
5.05
1.03
7.04
f1 (ppm)

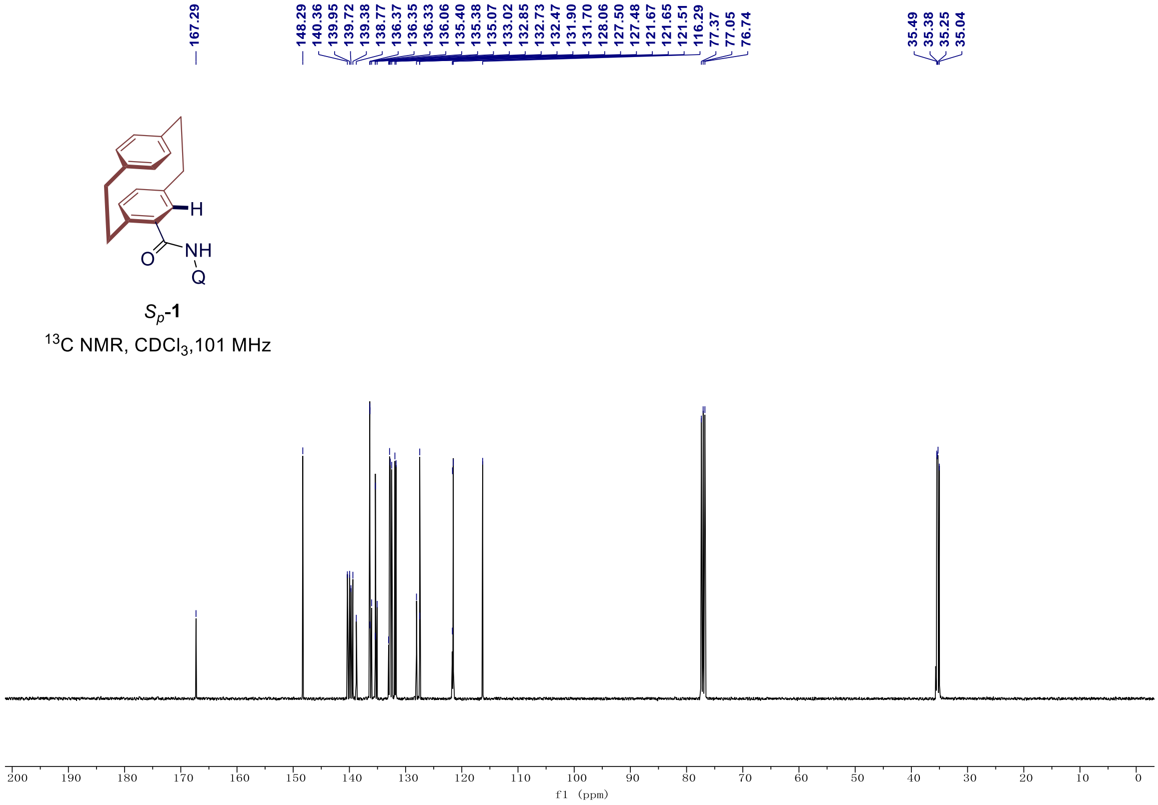
167.29
148.29
140.36
139.95
139.72
139.38
138.77
136.37
136.35
136.33
136.06
135.40
135.38
135.07
133.02
132.85
132.73
132.47
131.90
131.70
128.06
127.50
127.48
121.67
121.65
121.51
116.29
77.37
77.05
76.74
35.49
35.38
35.25
35.04
H
O
NH
Q
S_p-**1**
^{13}C NMR, $CDCl_3$,101 MHz
f1 (ppm)

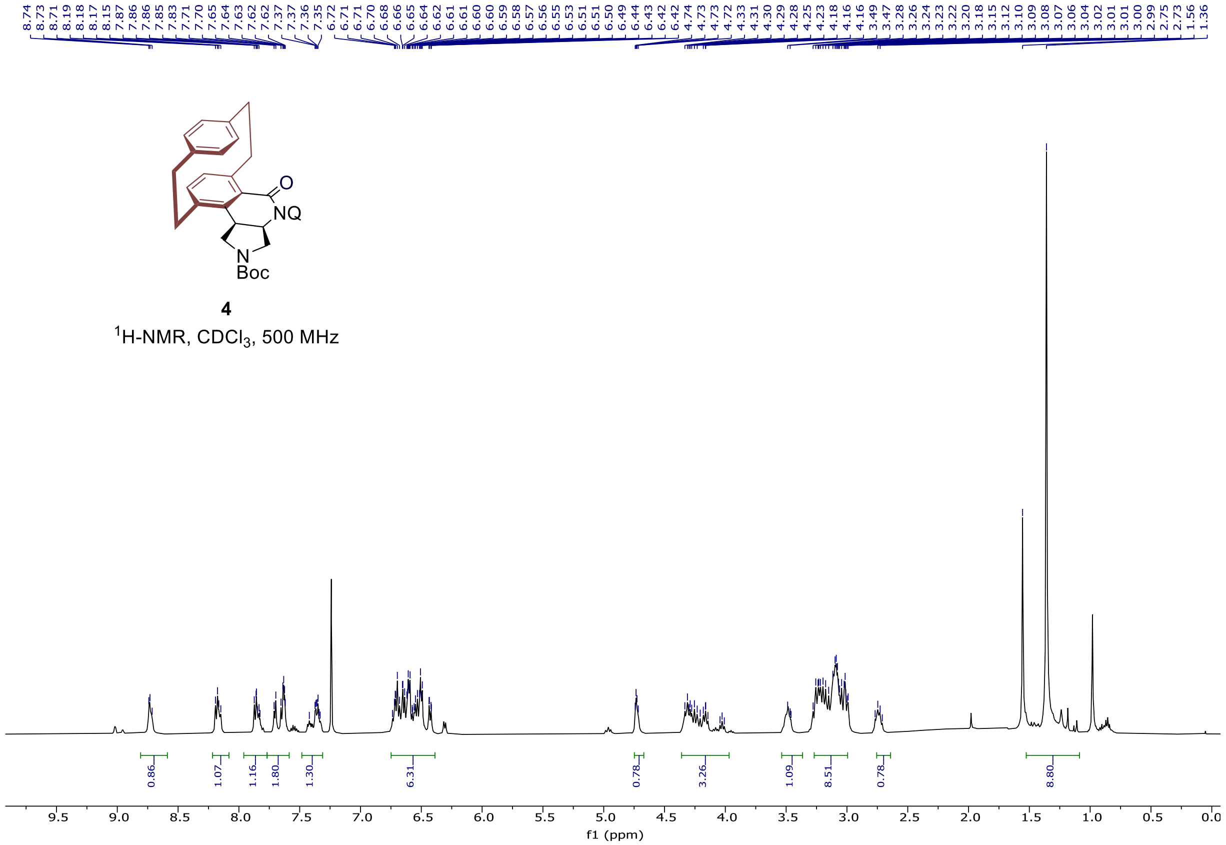

4

^{1}H-NMR, $CDCl_3$, 500 MHz

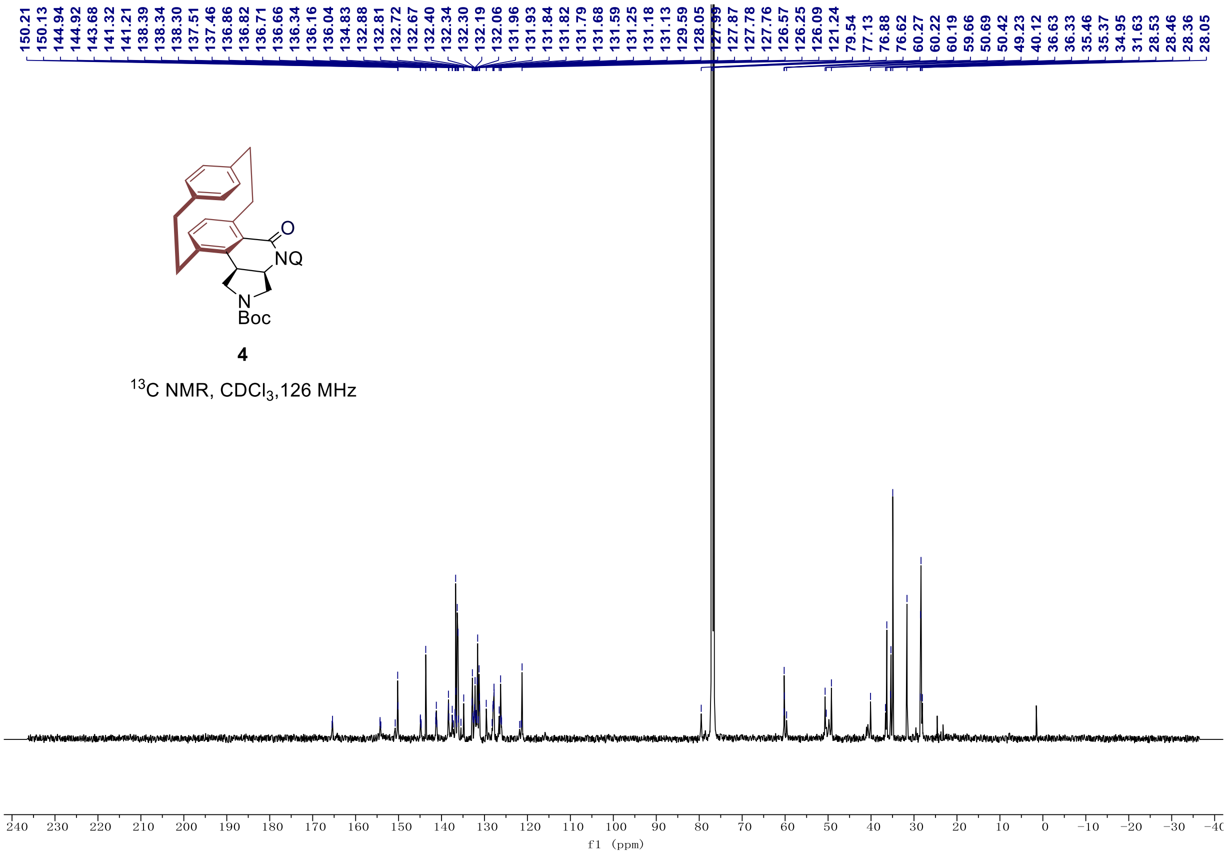

4

^{13}C NMR, $CDCl_3$,126 MHz

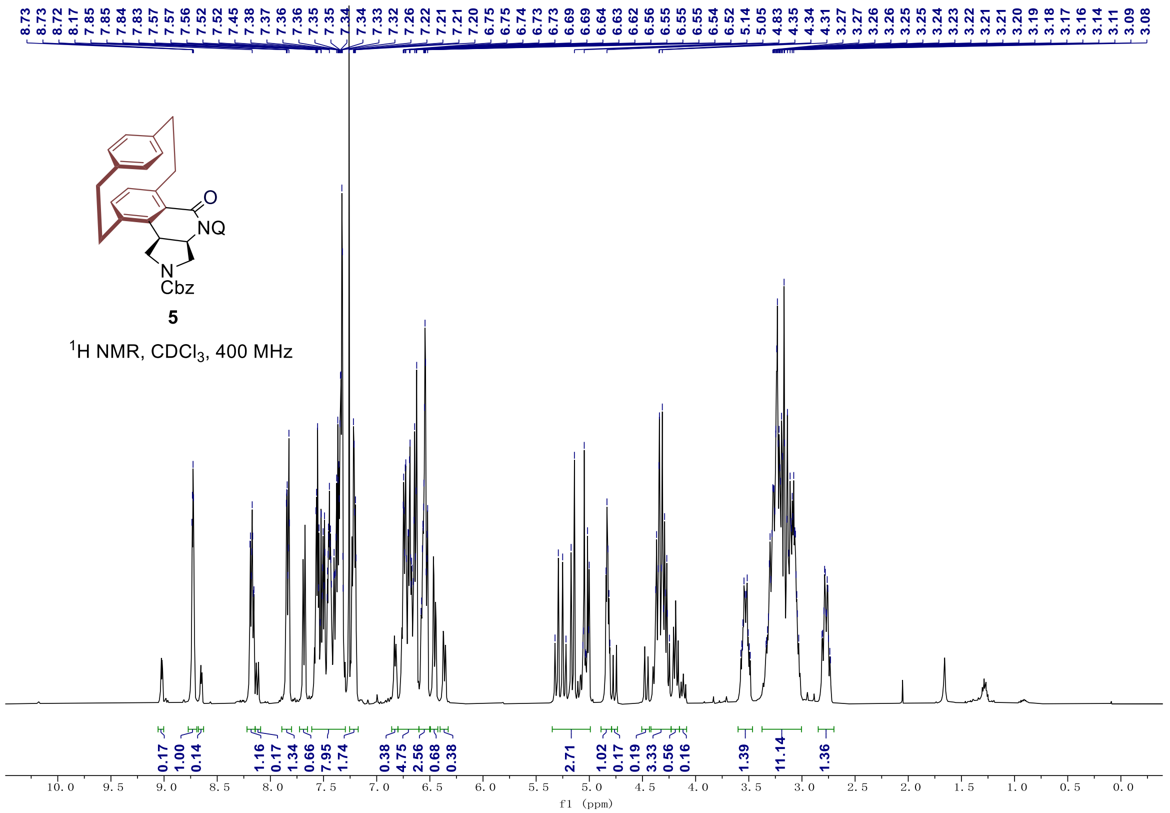
8.73 8.73 8.72 8.17 7.85 7.85 7.84 7.83 7.57 7.57 7.56 7.52 7.52 7.45 7.38 7.37 7.36 7.36 7.35 7.35 7.34 7.34 7.33 7.32 7.26 7.22 7.21 7.21 7.20 6.75 6.75 6.74 6.73 6.73 6.69 6.69 6.64 6.63 6.62 6.56 6.55 6.55 6.54 6.52 5.14 5.05 4.83 4.35 4.34 4.31 3.27 3.27 3.26 3.26 3.25 3.25 3.24 3.23 3.22 3.21 3.21 3.20 3.19 3.18 3.17 3.16 3.14 3.11 3.09 3.08
O
NQ
N
Cbz
5
^{1}H NMR, $CDCl_3$, 400 MHz
0.17 1.00 0.14 1.16 0.17 1.34 0.66 7.95 1.74 0.38 4.75 2.56 0.68 0.38 2.71 1.02 0.17 0.19 3.33 0.56 0.16 1.39 11.14 1.36
10.0 9.5 9.0 8.5 8.0 7.5 7.0 6.5 6.0 5.5 5.0 4.5 4.0 3.5 3.0 2.5 2.0 1.5 1.0 0.5 0.0
f1 (ppm)

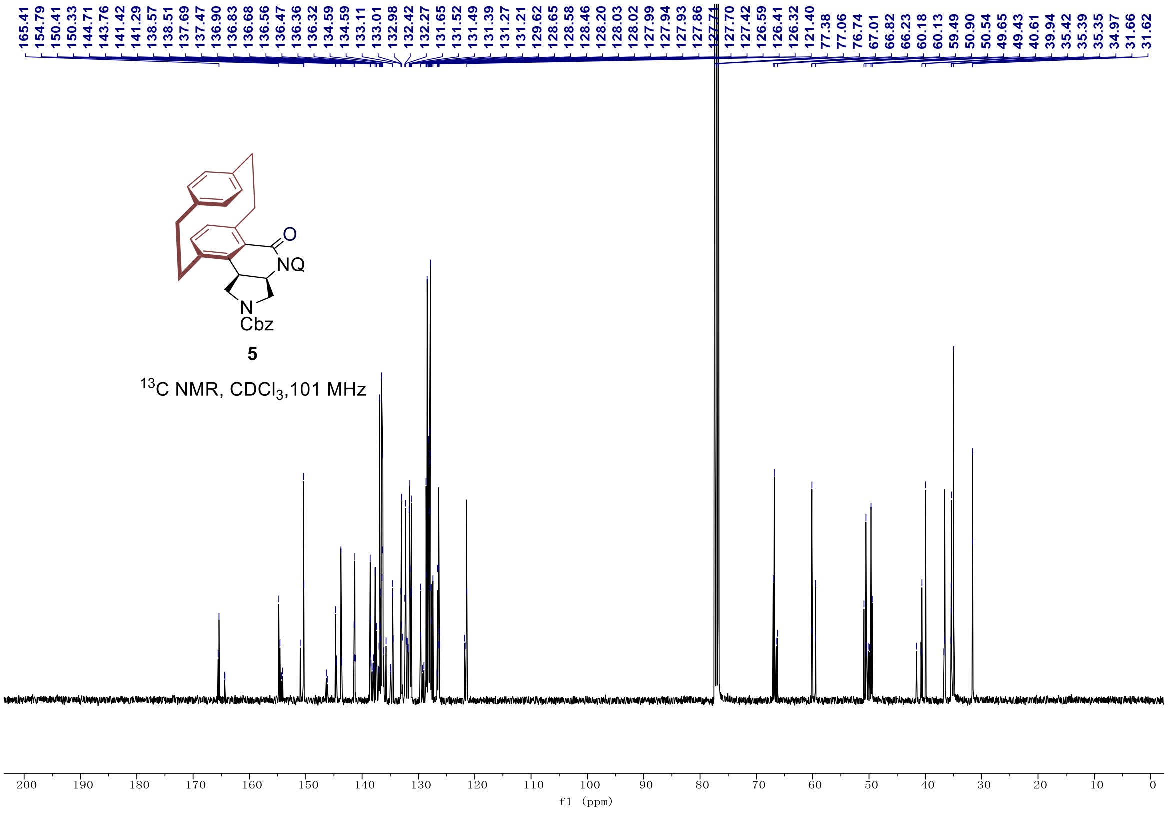
165.41 154.79 150.41 150.33 144.71 143.76 141.42 141.29 138.57 138.51 137.69 137.47 136.90 136.83 136.68 136.56 136.47 136.36 136.32 134.59 134.59 133.11 133.01 132.98 132.42 132.27 131.65 131.52 131.49 131.39 131.27 131.21 129.62 128.65 128.58 128.46 128.20 128.03 128.02 127.99 127.94 127.93 127.86 127.70 127.42 126.59 126.41 126.32 121.40 77.38 77.06 76.74 67.01 66.82 66.23 60.18 60.13 59.49 50.90 50.54 49.65 49.43 40.61 39.94 35.42 35.39 35.35 34.97 31.66 31.62
O
NQ
N
Cbz
5
^{13}C NMR, $CDCl_3$,101 MHz
200 190 180 170 160 150 140 130 120 110 100 90 80 70 60 50 40 30 20 10 0
f1 (ppm)

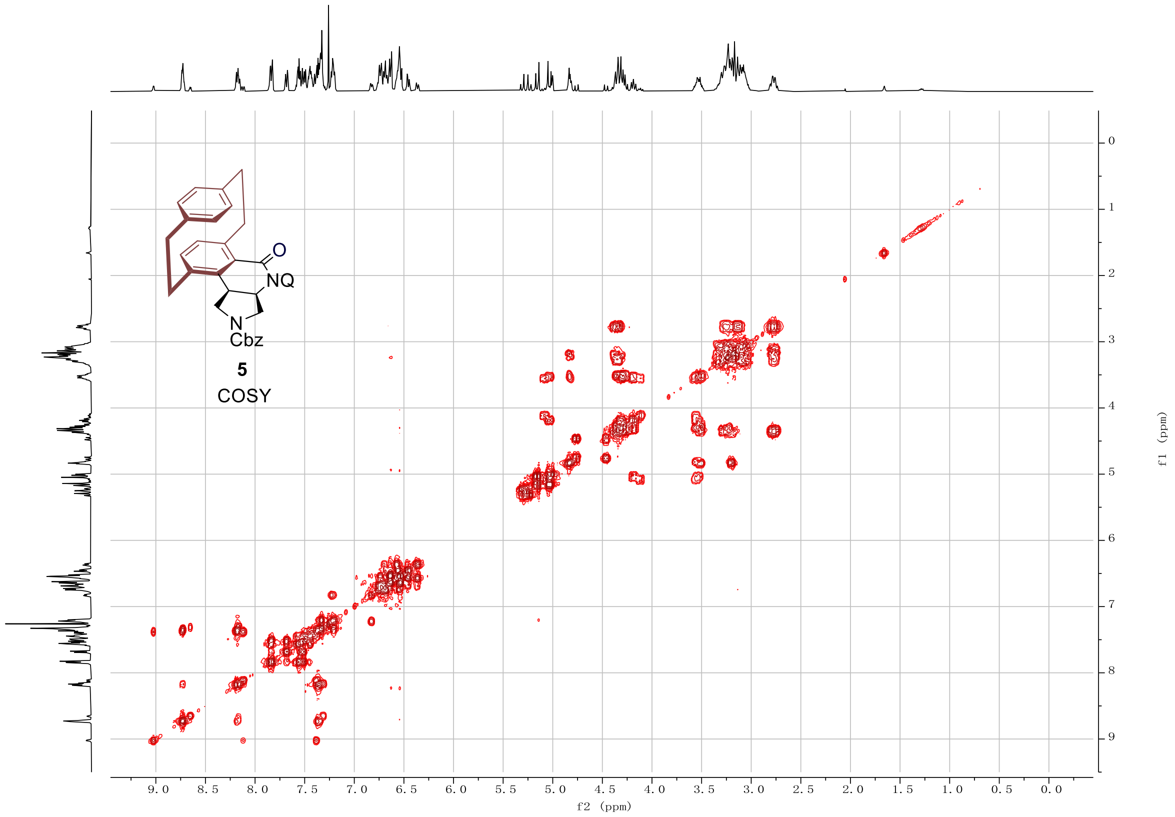
O
NQ
N
Cbz
5
COSY
f2 (ppm)
f1 (ppm)

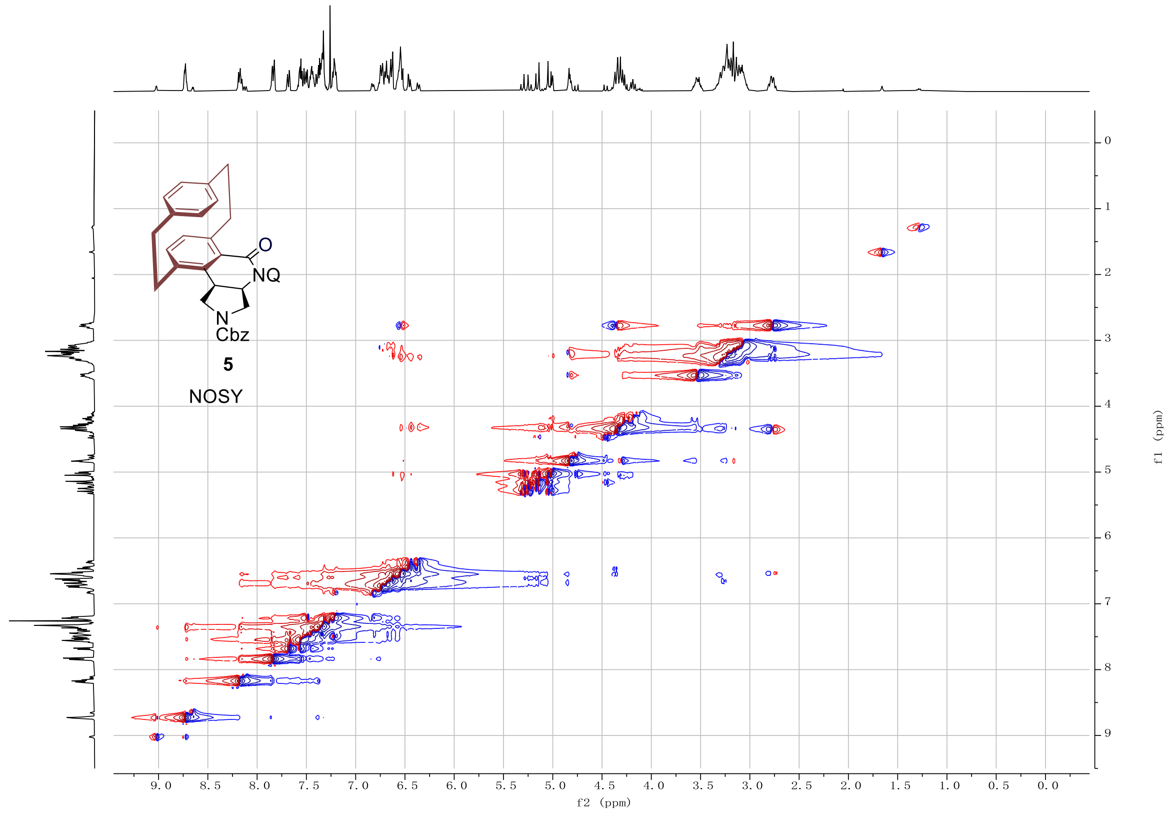
O
NQ
N
Cbz
5
NOSY
f2 (ppm)
f1 (ppm)

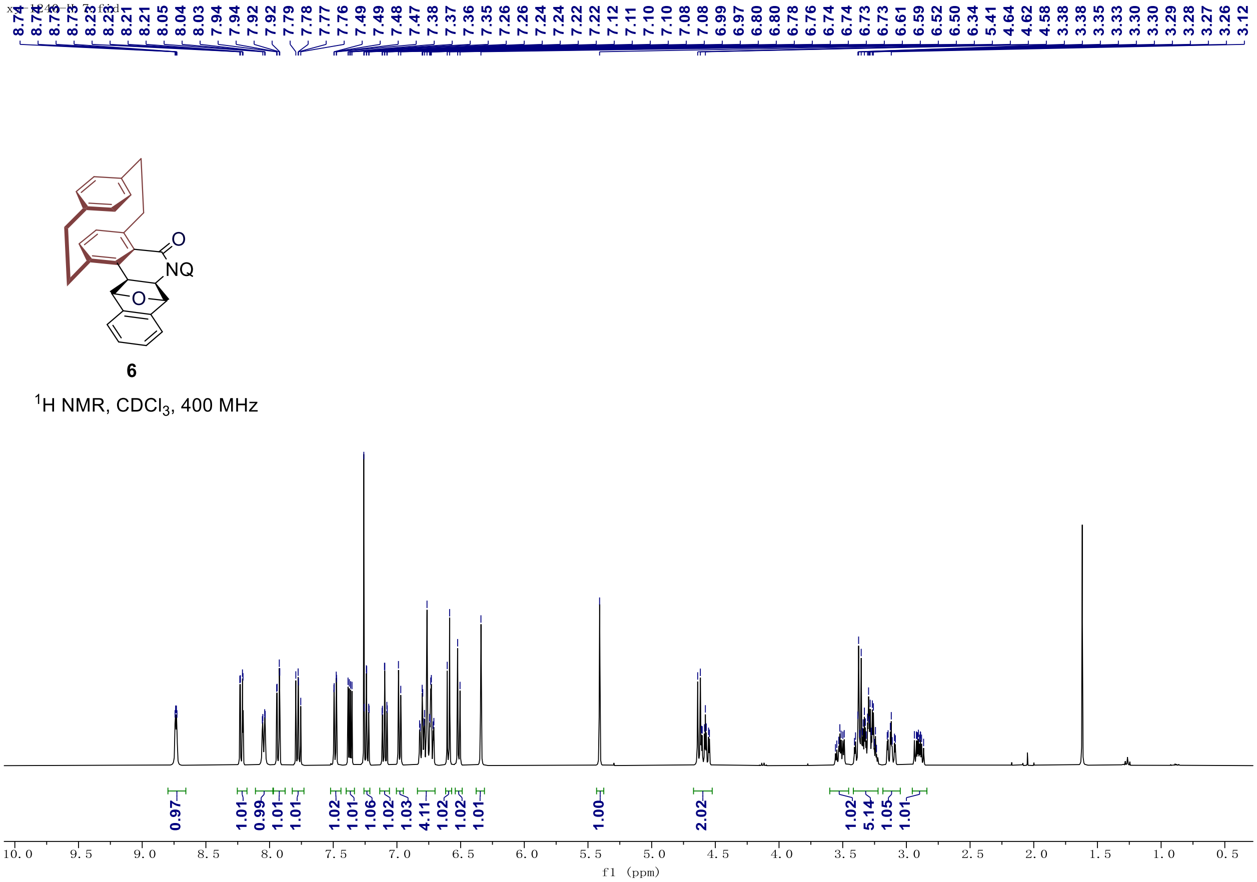

6

^{1}H NMR, $CDCl_3$, 400 MHz

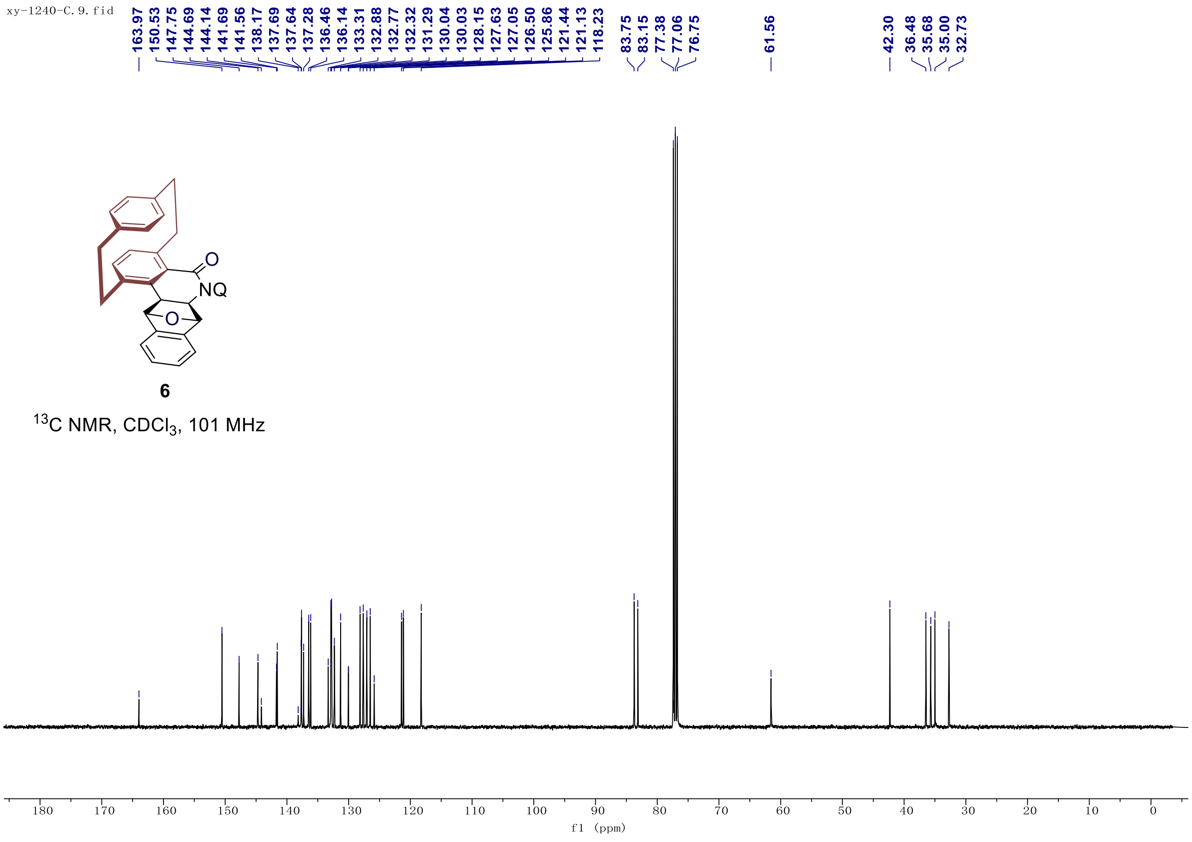

6

^{13}C NMR, $CDCl_3$, 101 MHz

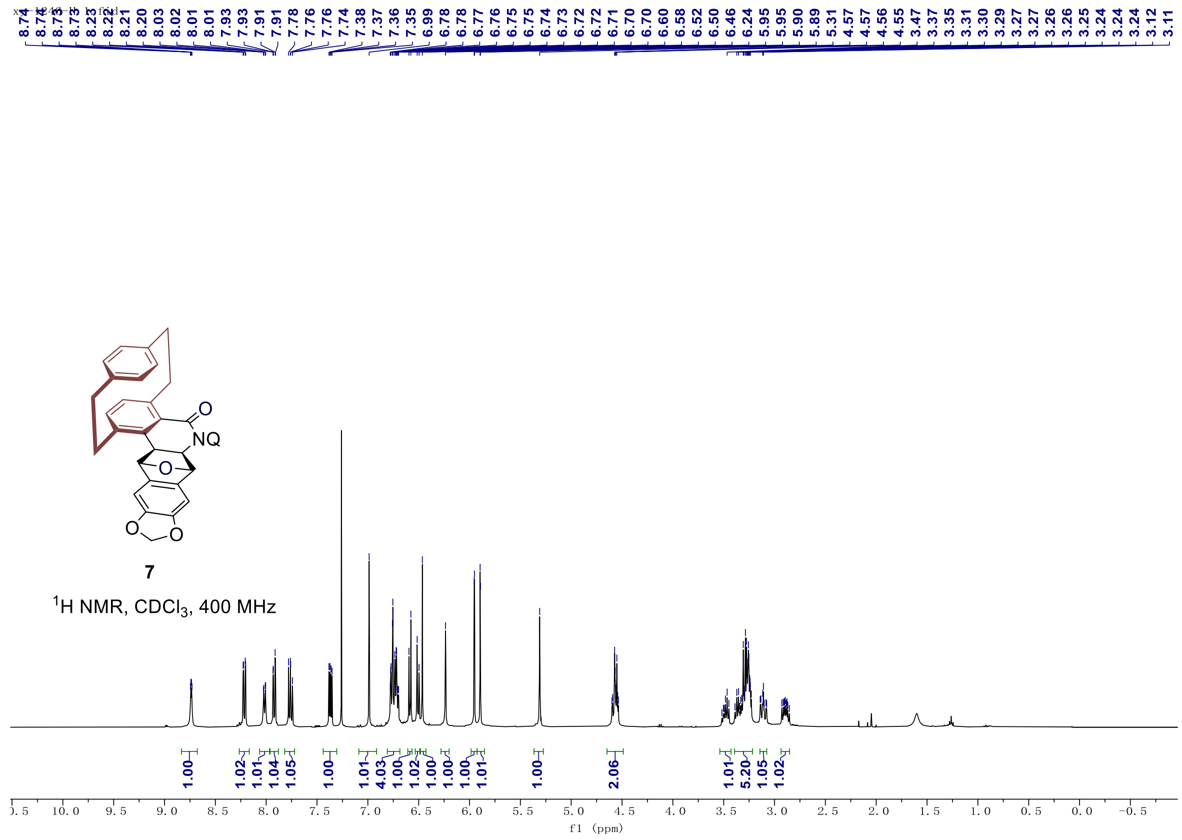
8.74 8.74 8.73 8.73 8.23 8.22 8.21 8.20 8.03 8.02 8.01 8.01 7.93 7.93 7.91 7.91 7.78 7.76 7.76 7.74 7.38 7.37 7.36 7.35 6.99 6.78 6.78 6.77 6.76 6.75 6.75 6.74 6.73 6.72 6.72 6.71 6.70 6.70 6.60 6.58 6.52 6.50 6.46 6.24 5.95 5.95 5.90 5.89 5.31 4.57 4.57 4.56 4.55 3.47 3.37 3.35 3.31 3.30 3.29 3.27 3.26 3.26 3.25 3.24 3.24 3.24 3.12 3.11
O
NQ
O
O
O
7
^{1}H NMR, $CDCl_3$, 400 MHz
1.00 1.02 1.01 1.04 1.05 1.00 1.01 4.03 1.00 1.02 1.00 1.00 1.01 1.00 2.06 1.01 5.20 1.05 1.02
f1 (ppm)

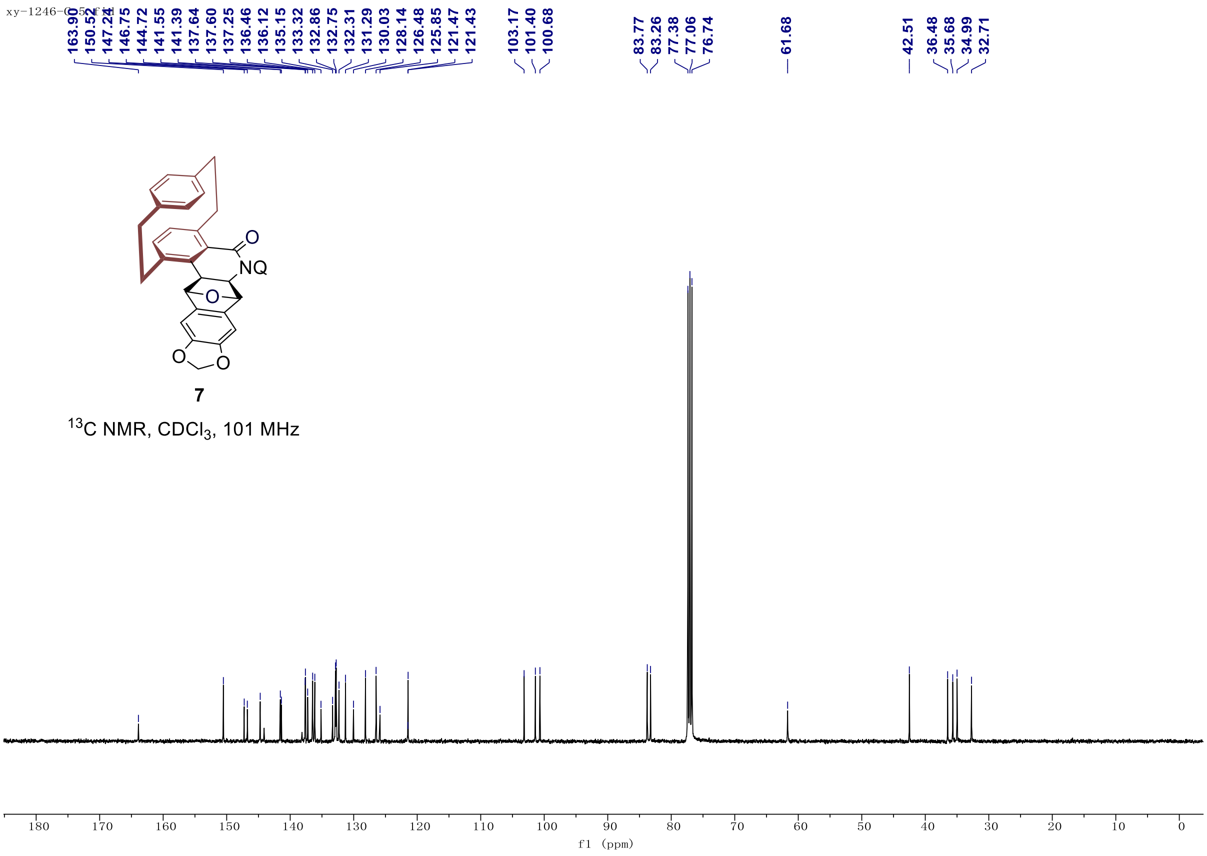
163.90 150.52 147.24 146.75 144.72 141.55 141.39 137.64 137.60 137.25 136.46 136.12 135.15 133.32 132.86 132.75 132.31 131.29 130.03 128.14 126.48 125.85 121.47 121.43 103.17 101.40 100.68 83.77 83.26 77.38 77.06 76.74 61.68 42.51 36.48 35.68 34.99 32.71
O
NQ
O
O
O
7
^{13}C NMR, $CDCl_3$, 101 MHz
f1 (ppm)

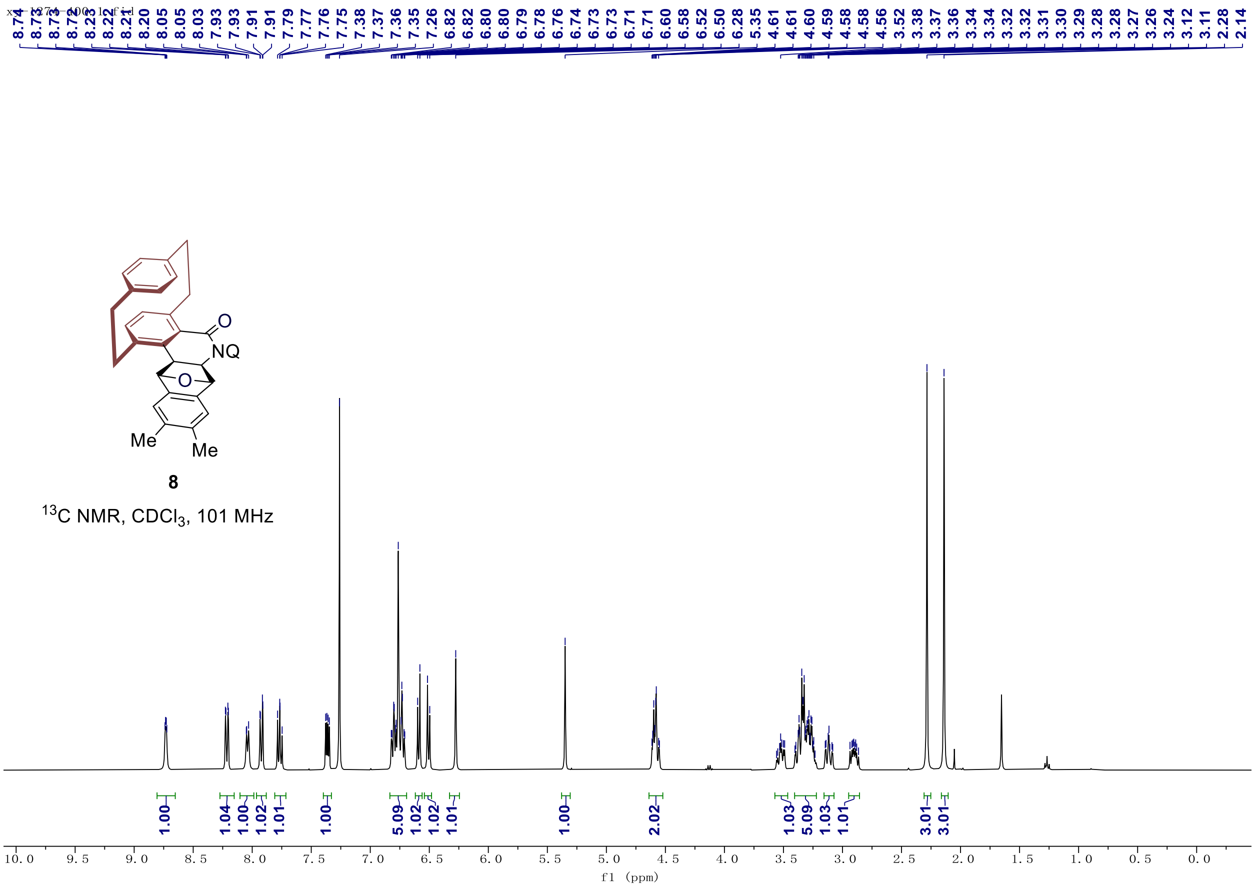
8.74 8.73 8.73 8.72 8.23 8.22 8.21 8.20 8.05 8.05 8.03 7.93 7.93 7.91 7.91 7.79 7.77 7.76 7.75 7.38 7.37 7.36 7.35 7.26 6.82 6.82 6.80 6.80 6.79 6.78 6.76 6.74 6.73 6.73 6.71 6.71 6.60 6.58 6.52 6.50 6.28 5.35 4.61 4.61 4.60 4.59 4.58 4.56 3.52 3.38 3.37 3.36 3.34 3.34 3.32 3.32 3.31 3.30 3.29 3.28 3.28 3.27 3.26 3.24 3.12 3.11 2.28 2.14
O
NQ
O
Me
Me
8
^{13}C NMR, $CDCl_3$, 101 MHz
1.00 1.04 1.00 1.02 1.01 1.00 5.09 1.02 1.02 1.01 1.00 2.02 1.03 5.09 1.03 1.01 3.01 3.01
10.0 9.5 9.0 8.5 8.0 7.5 7.0 6.5 6.0 5.5 5.0 4.5 4.0 3.5 3.0 2.5 2.0 1.5 1.0 0.5 0.0
f1 (ppm)

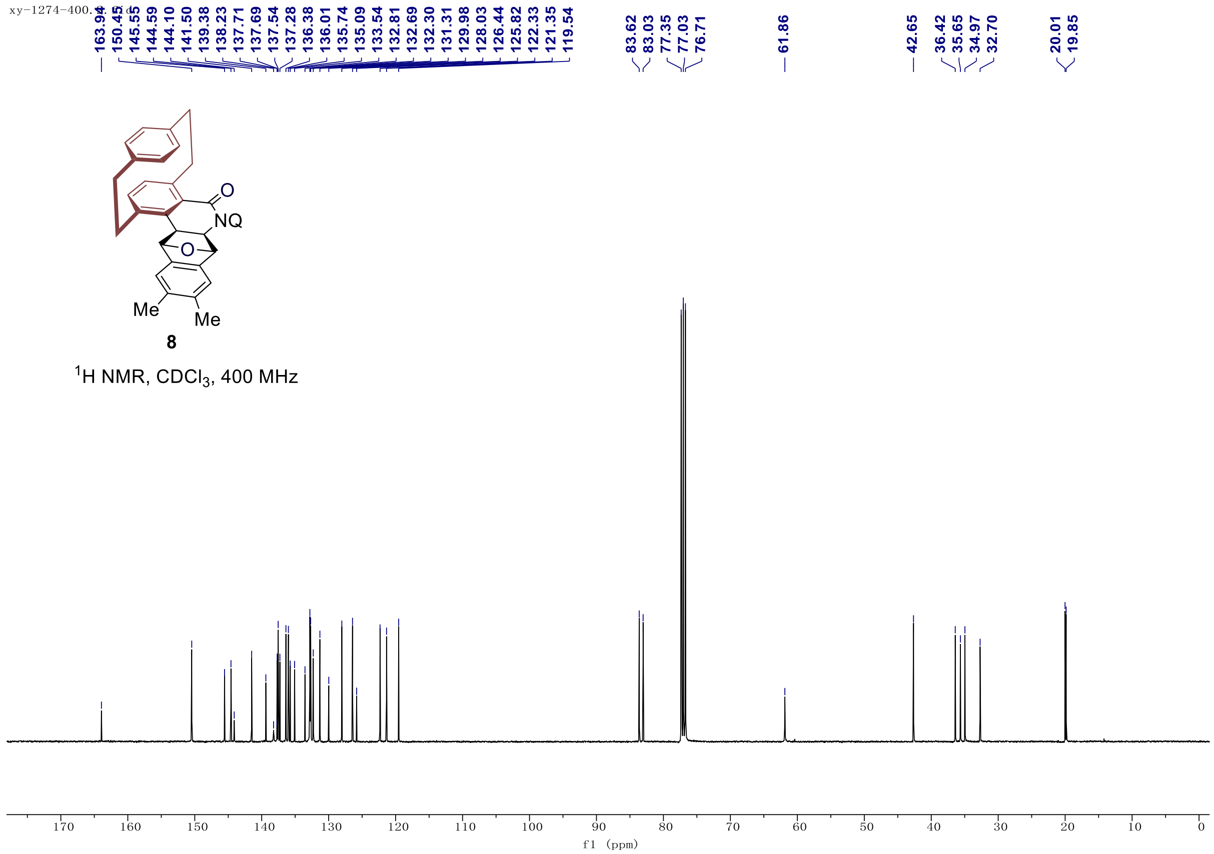
163.94 150.45 145.55 144.59 144.10 141.50 139.38 138.23 137.71 137.69 137.54 137.28 136.38 136.01 135.74 135.09 133.54 132.81 132.69 132.30 131.31 129.98 128.03 126.44 125.82 122.33 121.35 119.54
83.62 83.03 77.35 77.03 76.71
61.86
42.65 36.42 35.65 34.97 32.70
20.01 19.85
O
NQ
O
Me
Me
8
^{1}H NMR, $CDCl_3$, 400 MHz
170 160 150 140 130 120 110 100 90 80 70 60 50 40 30 20 10 0
f1 (ppm)

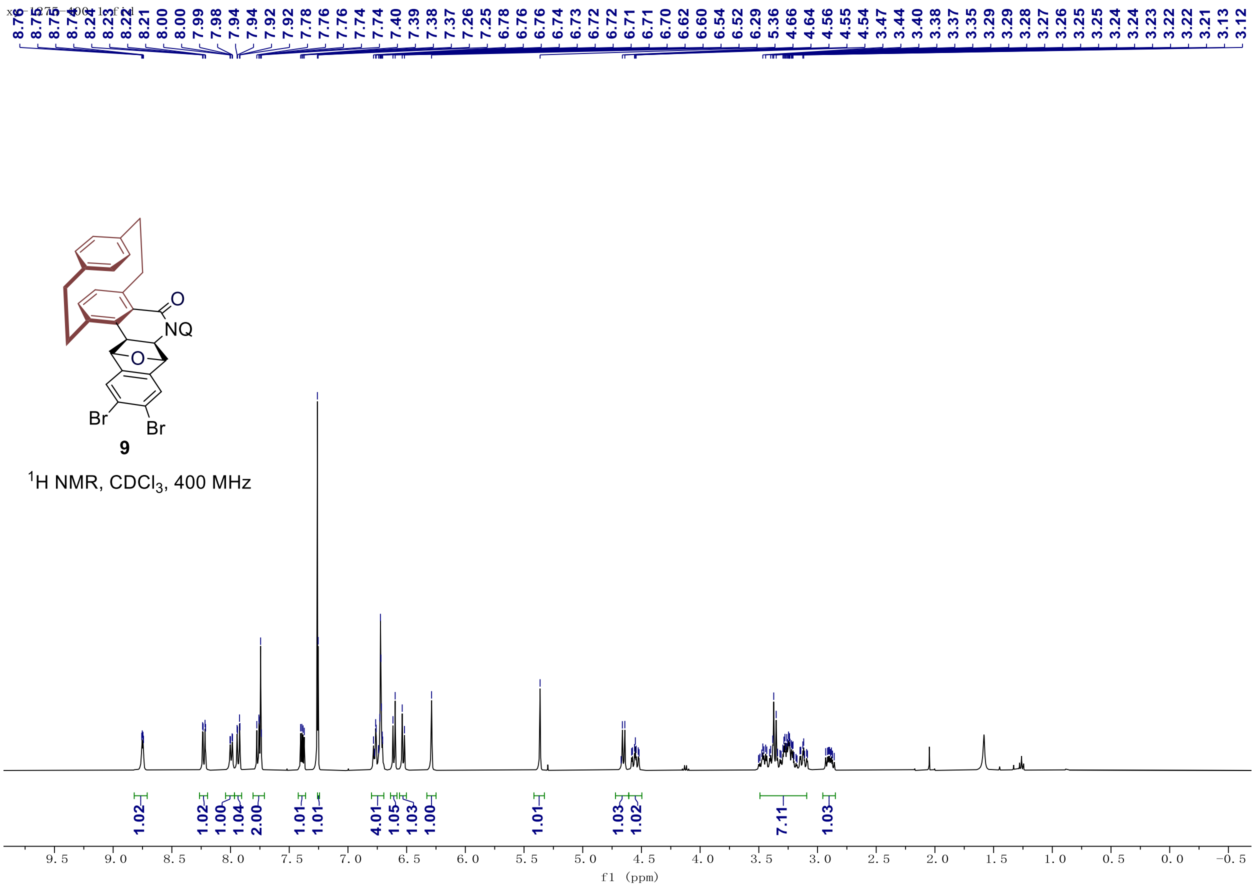
8.76 8.75 8.75 8.74 8.24 8.23 8.22 8.21 8.00 7.99 7.98 7.94 7.94 7.92 7.92 7.78 7.76 7.76 7.74 7.74 7.40 7.39 7.38 7.37 7.26 7.25 6.78 6.76 6.74 6.73 6.72 6.72 6.71 6.71 6.70 6.62 6.60 6.54 6.52 6.29 5.36 4.66 4.64 4.56 4.55 4.54 3.47 3.44 3.40 3.38 3.37 3.35 3.29 3.29 3.28 3.27 3.26 3.25 3.25 3.24 3.24 3.23 3.22 3.22 3.21 3.13 3.12
O
NQ
O
Br
Br
9
^{1}H NMR, CDCl$_3$, 400 MHz
1.02 1.02 1.00 1.04 2.00 1.01 1.01 4.01 1.05 1.03 1.00 1.01 1.03 1.02 7.11 1.03
9.5 9.0 8.5 8.0 7.5 7.0 6.5 6.0 5.5 5.0 4.5 4.0 3.5 3.0 2.5 2.0 1.5 1.0 0.5 0.0 -0.5
f1 (ppm)

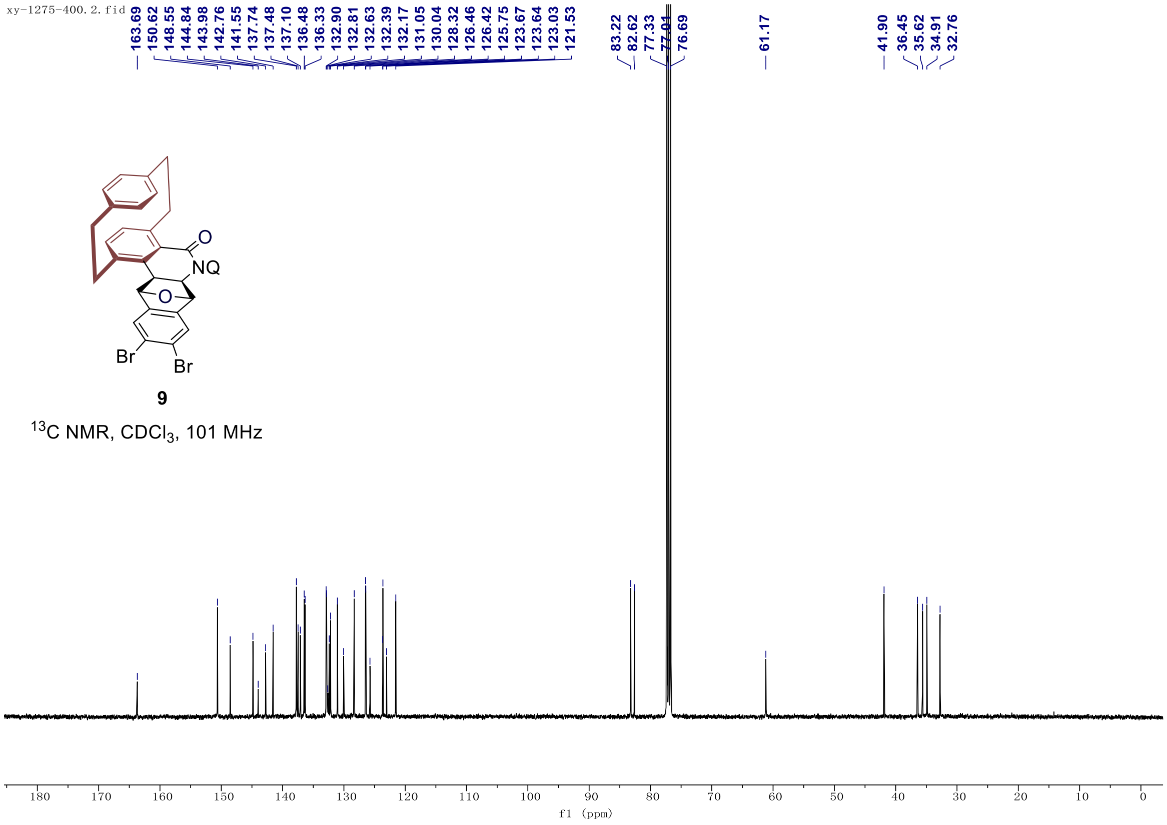
xy-1275-400.2.fid
163.69 150.62 148.55 144.84 143.98 142.76 141.55 137.74 137.48 137.10 136.48 136.33 132.90 132.81 132.63 132.39 132.17 131.05 130.04 128.32 126.46 126.42 125.75 123.67 123.64 123.03 121.53
83.22 82.62 77.33 77.01 76.69
61.17
41.90 36.45 35.62 34.91 32.76
O
NQ
O
Br
Br
9
^{13}C NMR, CDCl$_3$, 101 MHz
180 170 160 150 140 130 120 110 100 90 80 70 60 50 40 30 20 10 0
f1 (ppm)

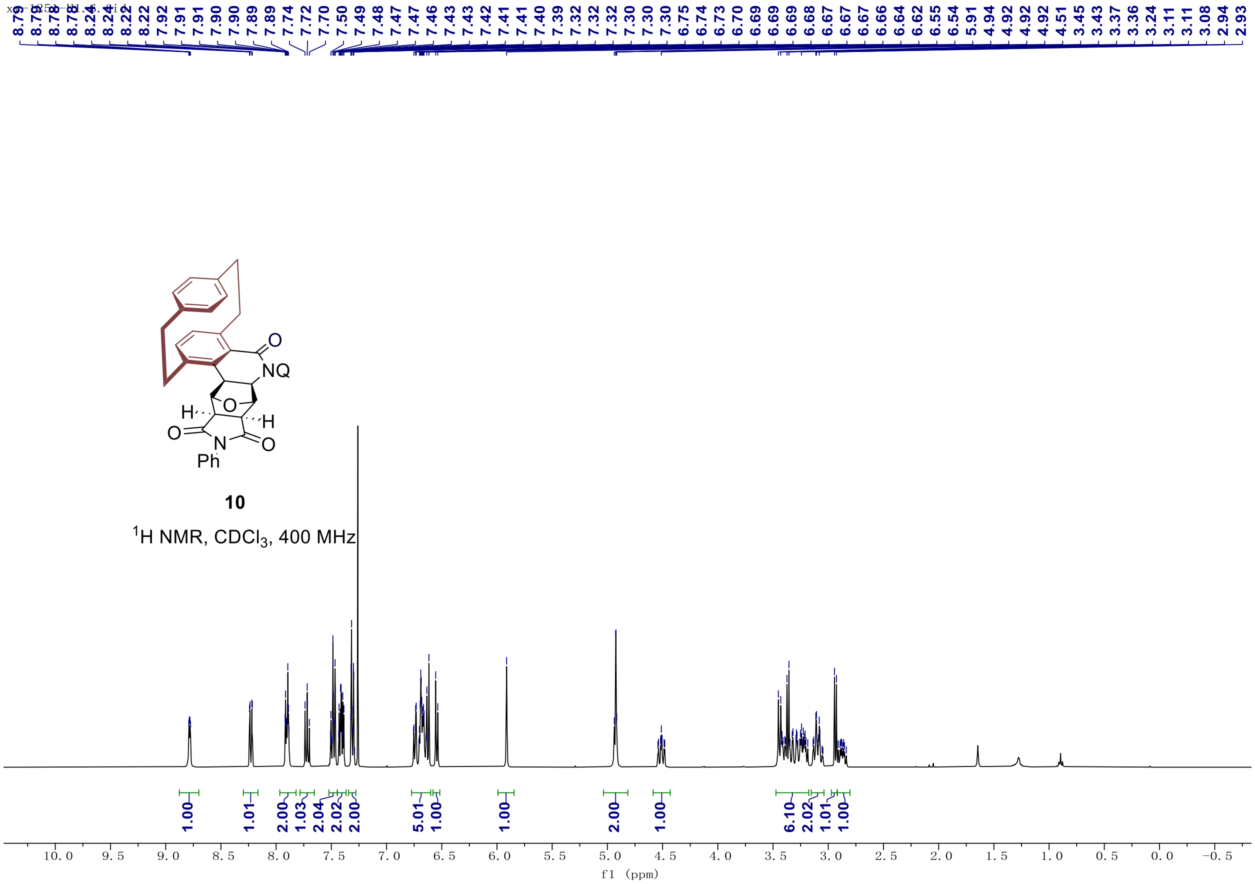
8.79 8.79 8.78 8.24 8.24 8.22 8.22 7.92 7.91 7.91 7.90 7.89 7.74 7.72 7.70 7.50 7.49 7.48 7.47 7.46 7.43 7.43 7.42 7.41 7.41 7.40 7.39 7.32 7.32 7.30 7.30 6.75 6.74 6.73 6.70 6.69 6.69 6.68 6.67 6.67 6.66 6.64 6.62 6.55 6.54 5.91 4.94 4.92 4.92 4.51 3.45 3.43 3.37 3.36 3.24 3.11 3.11 3.08 2.94 2.93
NQ
H
H
O
O
N
O
O
Ph
10
^{1}H NMR, CDCl$_3$, 400 MHz
1.00 1.01 2.00 1.03 2.04 2.02 2.00 5.01 1.00 1.00 2.00 1.00 6.10 2.02 1.01 1.00
f1 (ppm)

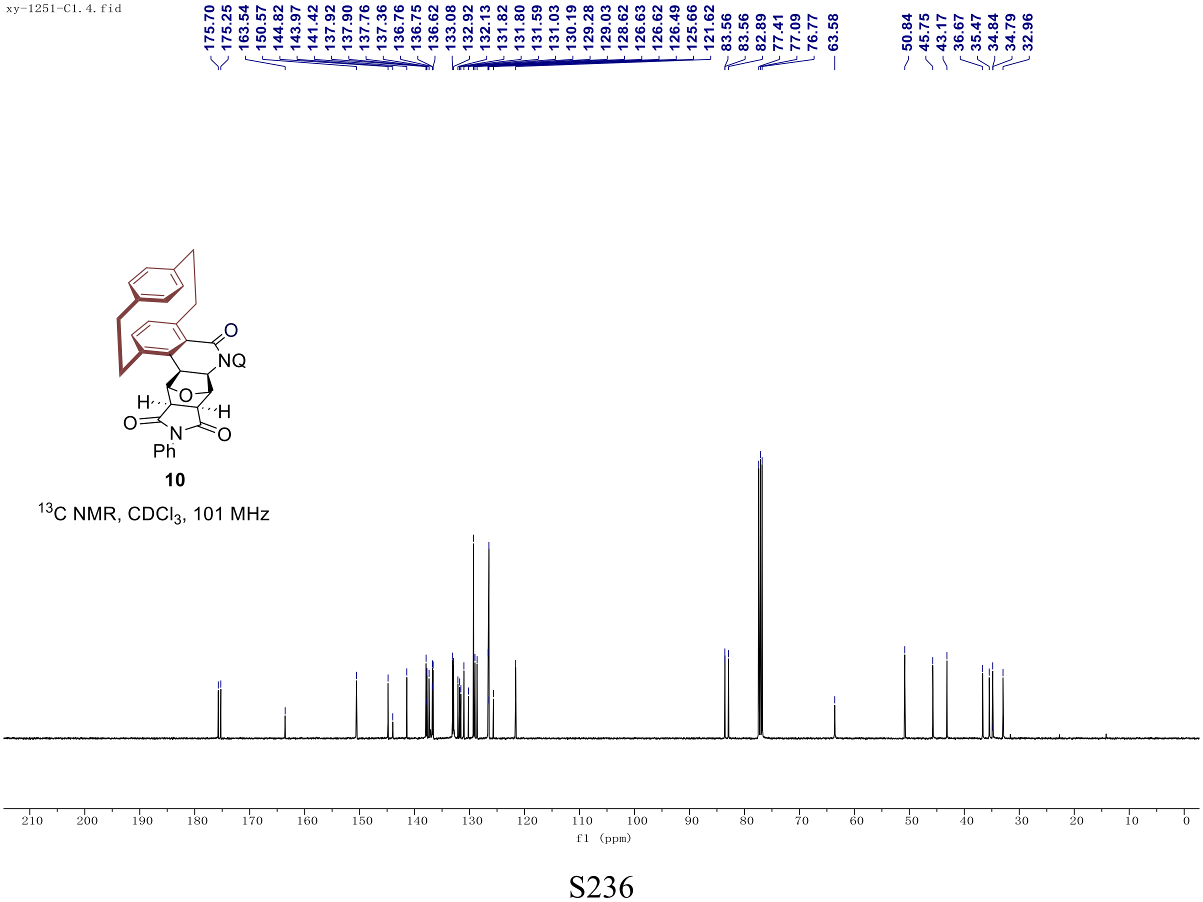
xy-1251-C1.4.fid
175.70 175.25 163.54 150.57 144.82 143.97 141.42 137.92 137.90 137.76 137.36 136.76 136.75 136.62 133.08 132.92 132.13 131.82 131.80 131.59 131.03 130.19 129.28 129.03 128.62 126.63 126.62 126.49 125.66 121.62 83.56 83.56 82.89 77.41 77.09 76.77 63.58 50.84 45.75 43.17 36.67 35.47 34.84 34.79 32.96
NQ
H
H
O
O
N
O
O
Ph
10
^{13}C NMR, CDCl$_3$, 101 MHz
f1 (ppm)

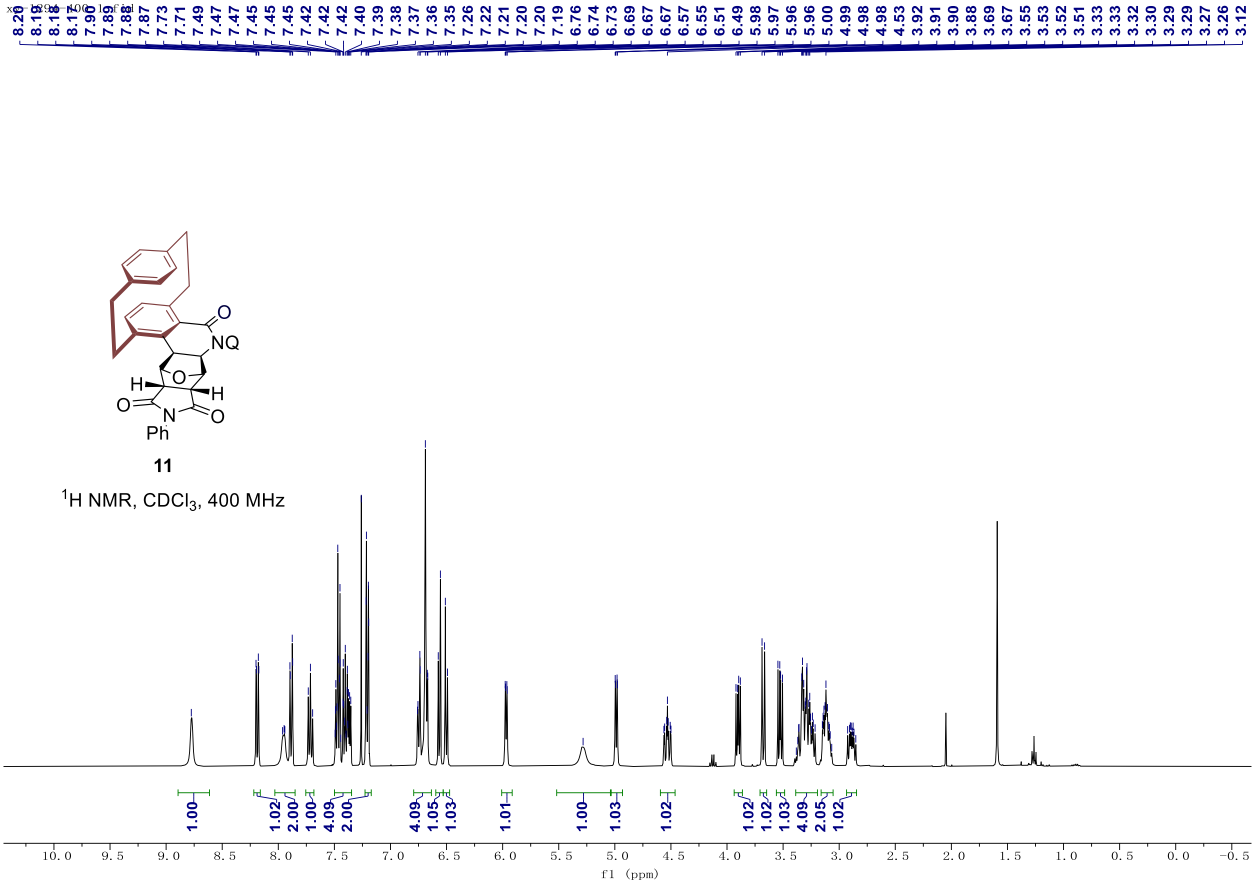
8.20 8.19 8.18 8.18 7.90 7.89 7.88 7.87 7.73 7.71 7.49 7.47 7.47 7.45 7.45 7.45 7.42 7.42 7.42 7.40 7.39 7.38 7.37 7.36 7.35 7.26 7.22 7.21 7.20 7.19 6.76 6.74 6.73 6.69 6.67 6.67 6.57 6.55 6.51 6.49 5.98 5.97 5.96 5.96 5.00 4.99 4.98 4.98 4.53 3.92 3.91 3.90 3.88 3.69 3.67 3.55 3.53 3.52 3.51 3.33 3.33 3.32 3.30 3.29 3.27 3.26 3.12
O
NQ
O
H
H
O
N
O
Ph
11
¹H NMR, CDCl₃, 400 MHz
1.00 1.02 2.00 1.00 4.09 2.00 4.09 1.05 1.03 1.01 1.00 1.03 1.02 1.02 1.02 1.03 4.09 2.05 1.02
10.0 9.5 9.0 8.5 8.0 7.5 7.0 6.5 6.0 5.5 5.0 4.5 4.0 3.5 3.0 2.5 2.0 1.5 1.0 0.5 0.0 -0.5
f1 (ppm)

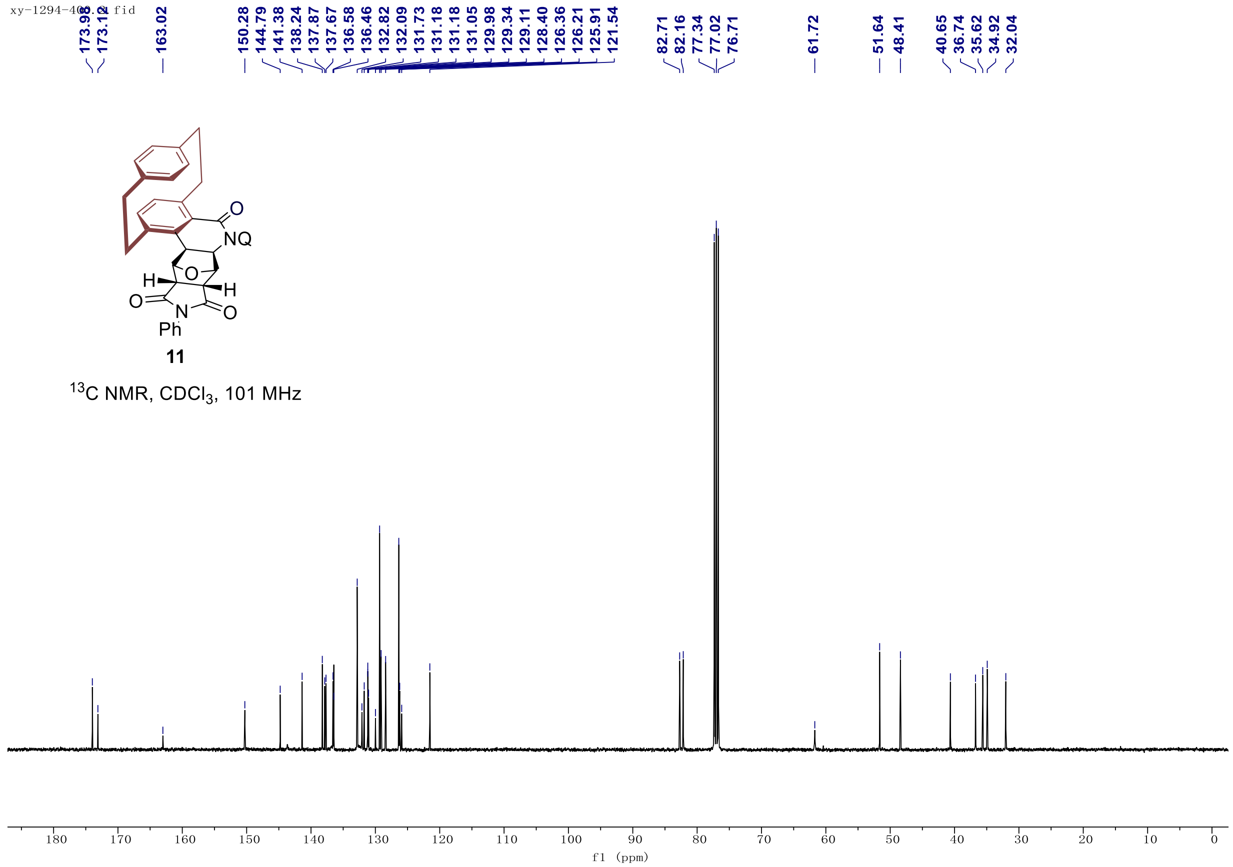
173.12 163.02 150.28 144.79 141.38 138.24 137.87 137.67 136.58 136.46 132.82 132.09 131.73 131.18 131.05 129.98 129.34 129.11 128.40 126.36 126.21 125.91 121.54 82.71 82.16 77.34 77.02 76.71 61.72 51.64 48.41 40.65 36.74 35.62 34.92 32.04
O
NQ
O
H
H
O
N
O
Ph
11
¹³C NMR, CDCl₃, 101 MHz
180 170 160 150 140 130 120 110 100 90 80 70 60 50 40 30 20 10 0
f1 (ppm)

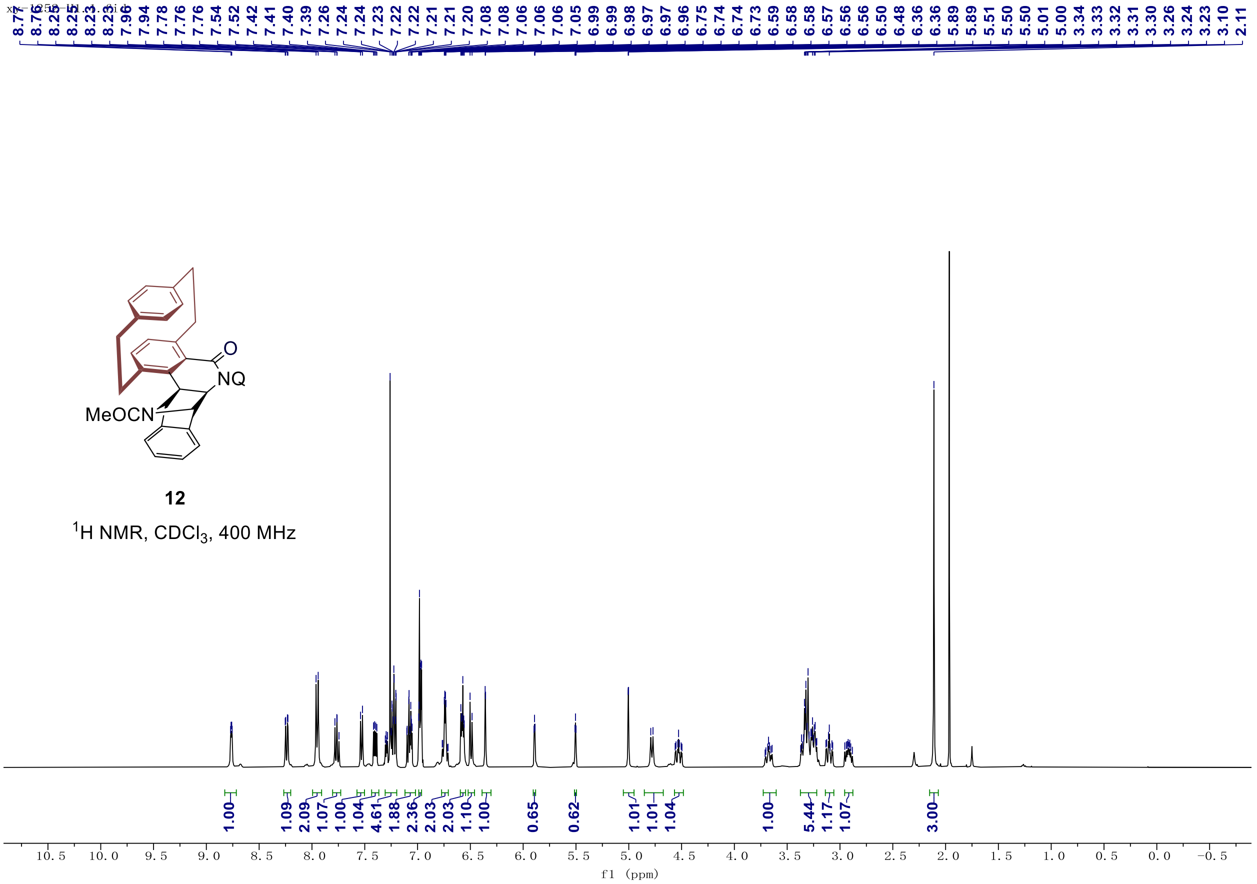

12

^{1}H NMR, $CDCl_3$, 400 MHz

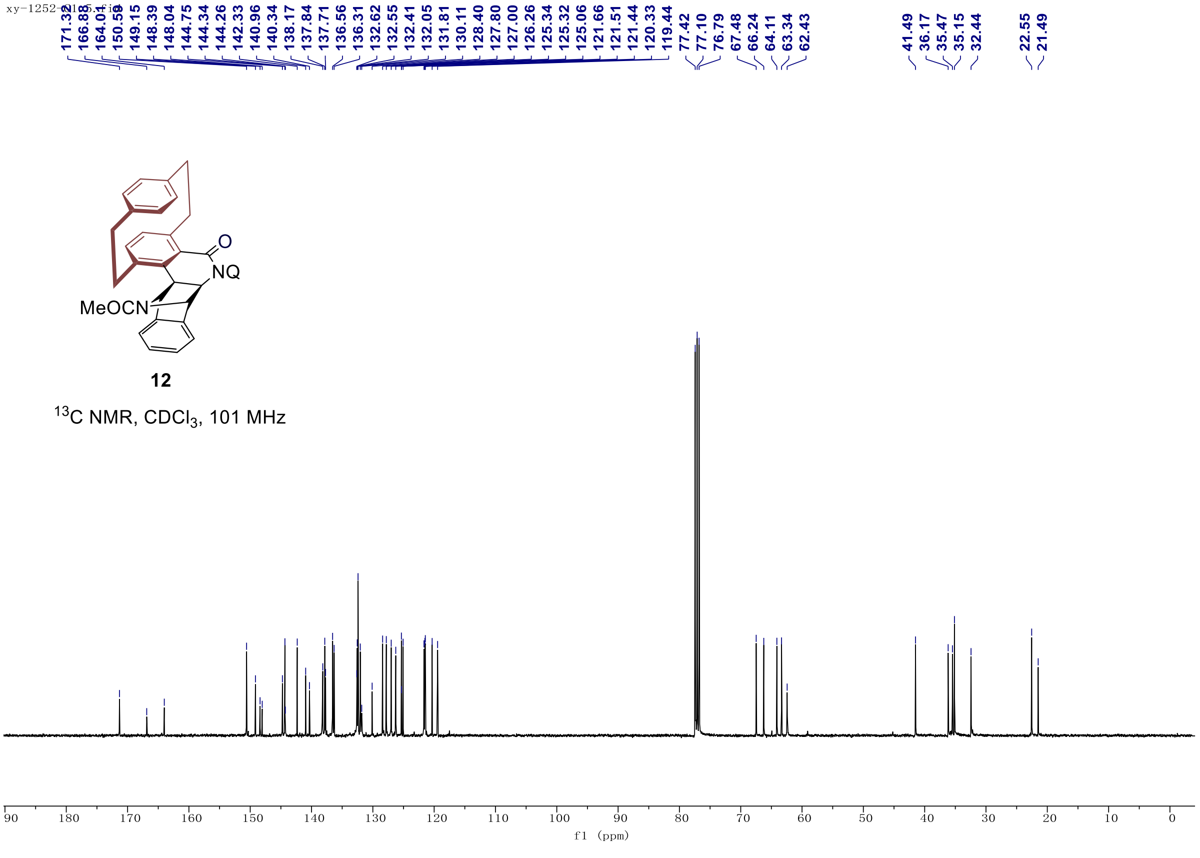

12

^{13}C NMR, $CDCl_3$, 101 MHz

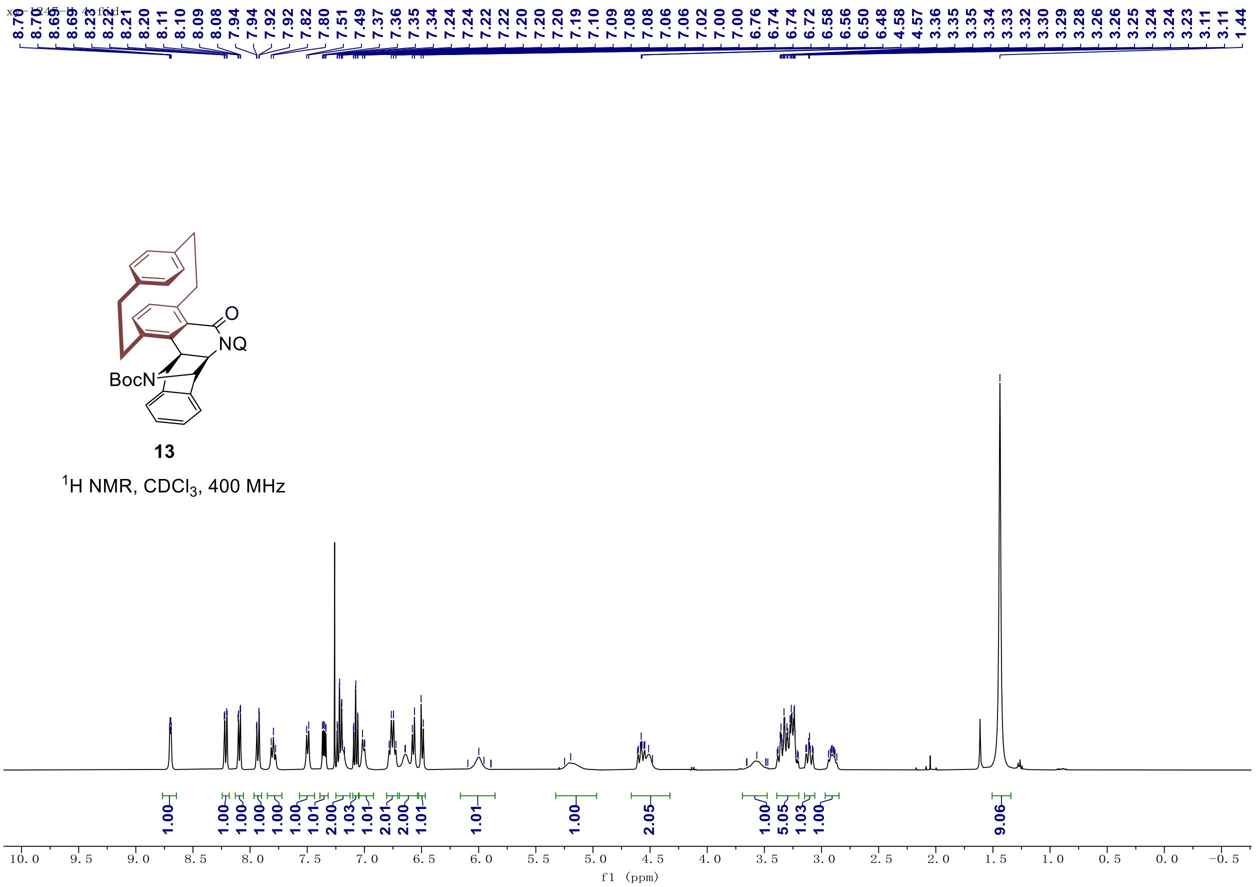

13

^{1}H NMR, $CDCl_3$, 400 MHz

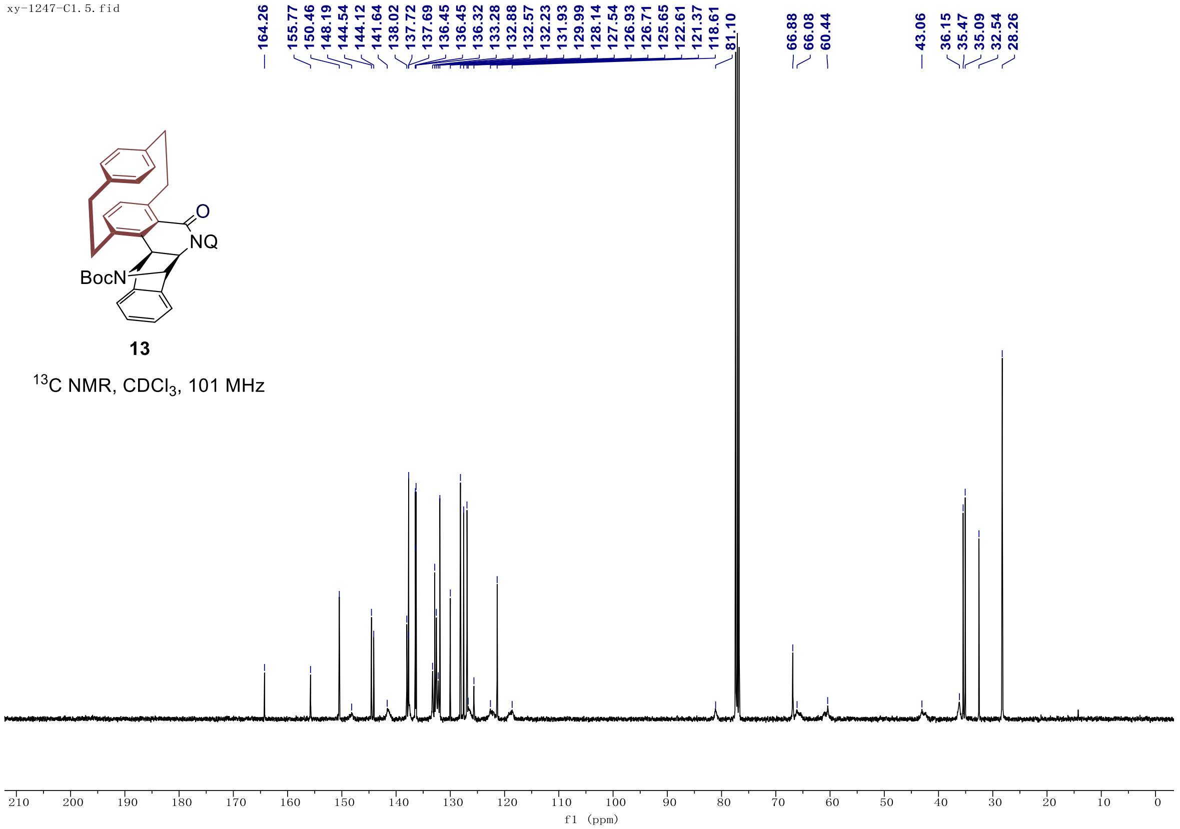

13

^{13}C NMR, $CDCl_3$, 101 MHz

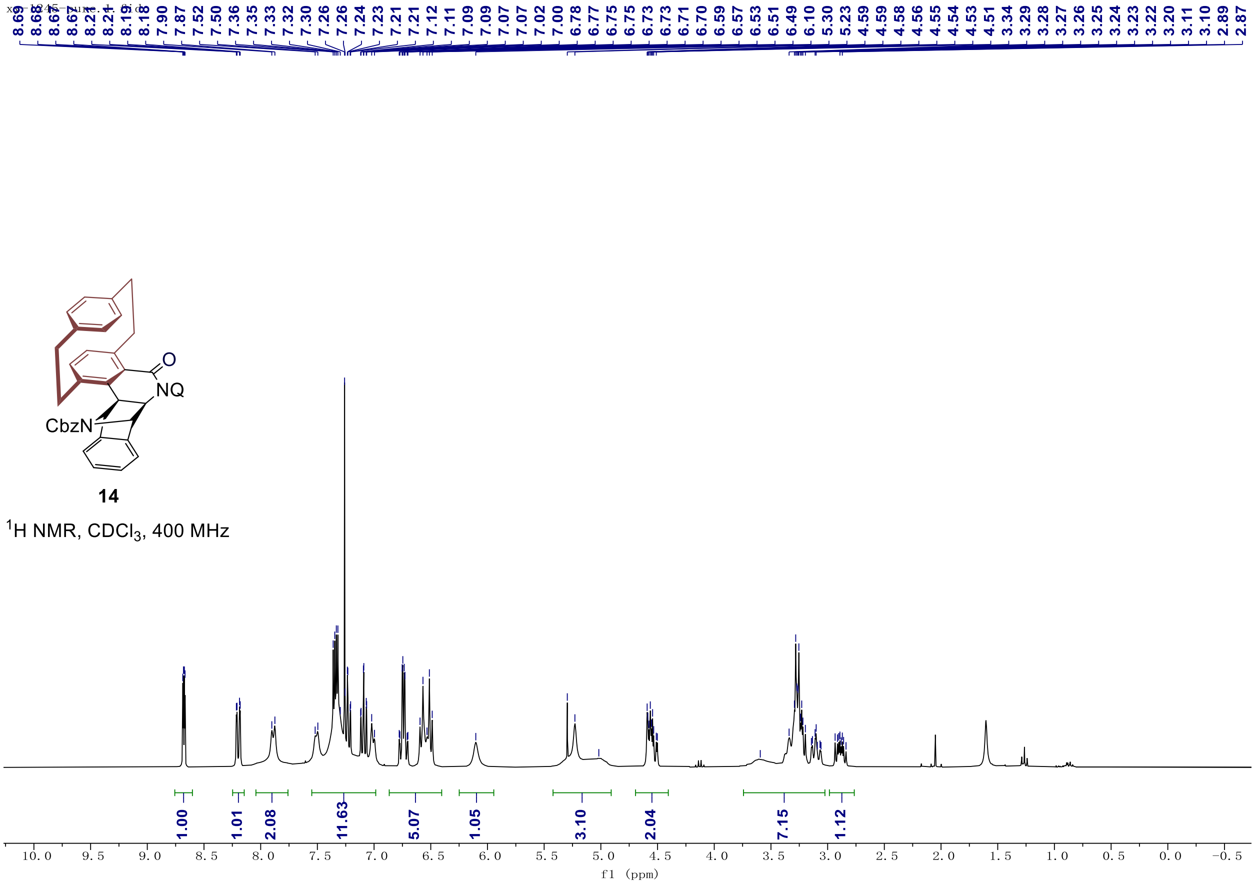

14

^{1}H NMR, $CDCl_3$, 400 MHz

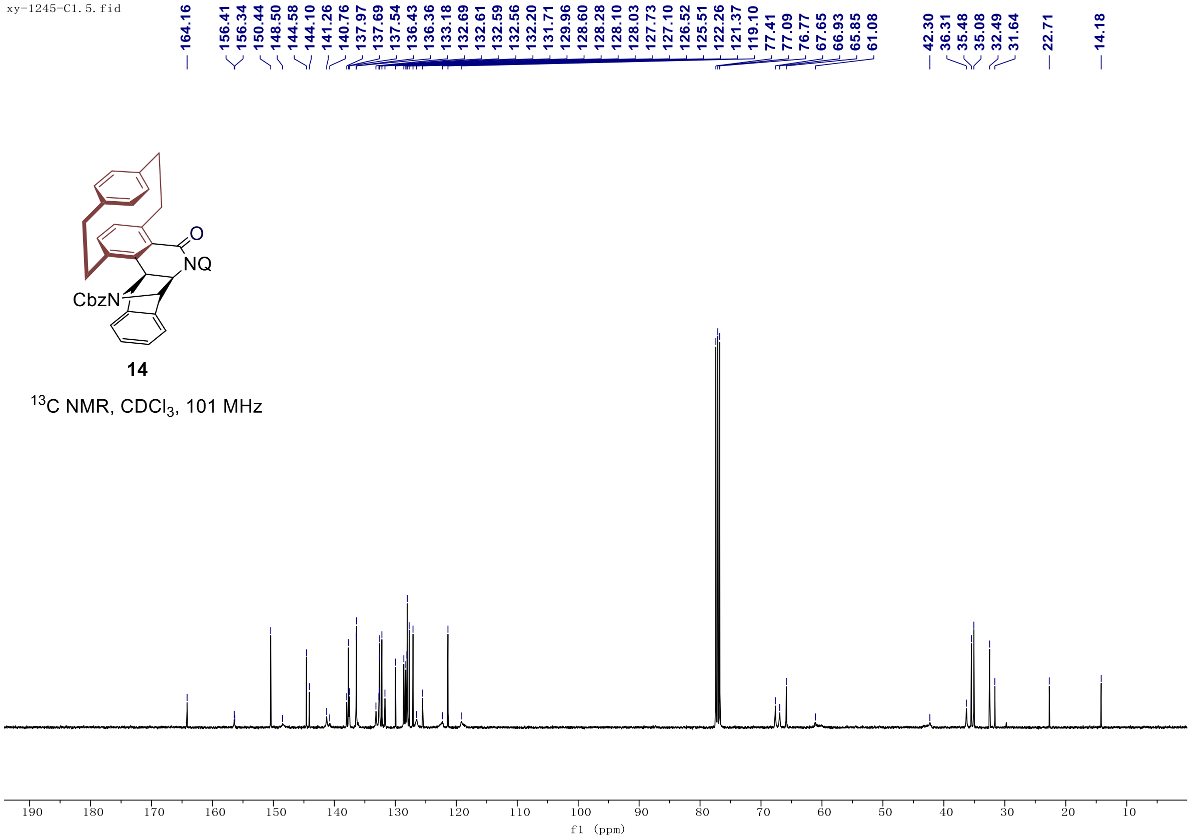

14

^{13}C NMR, $CDCl_3$, 101 MHz

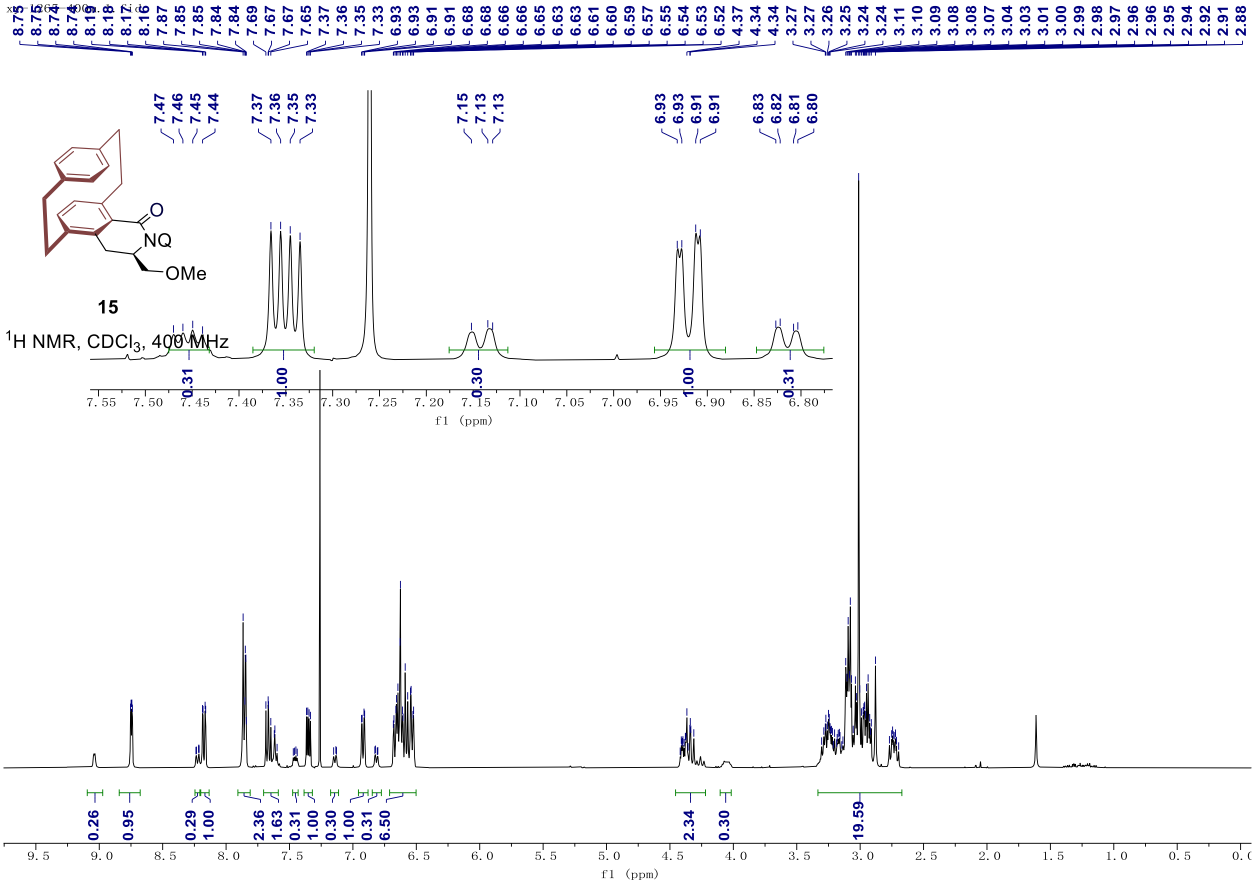
O
NQ
OMe
15
1H NMR, CDCl3, 400 MHz
f1 (ppm)

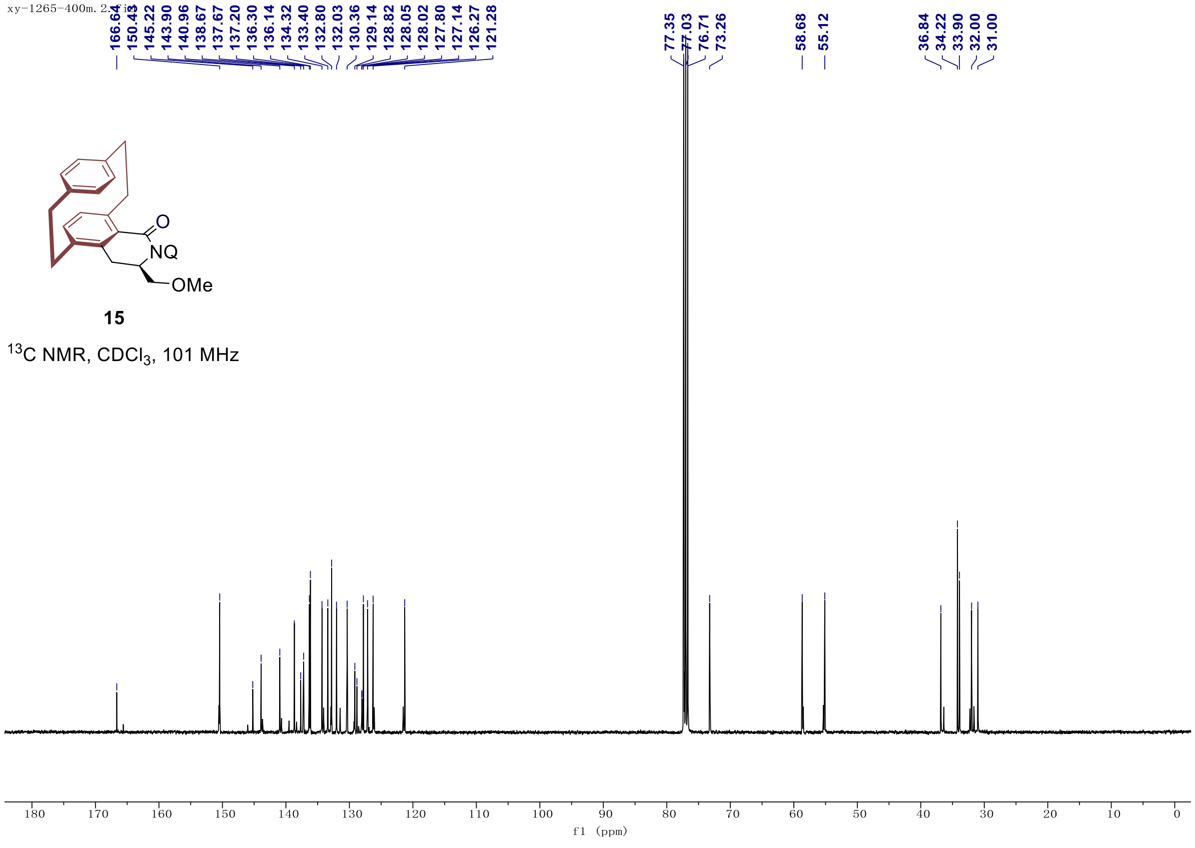
O
NQ
OMe
15
13C NMR, CDCl3, 101 MHz
f1 (ppm)

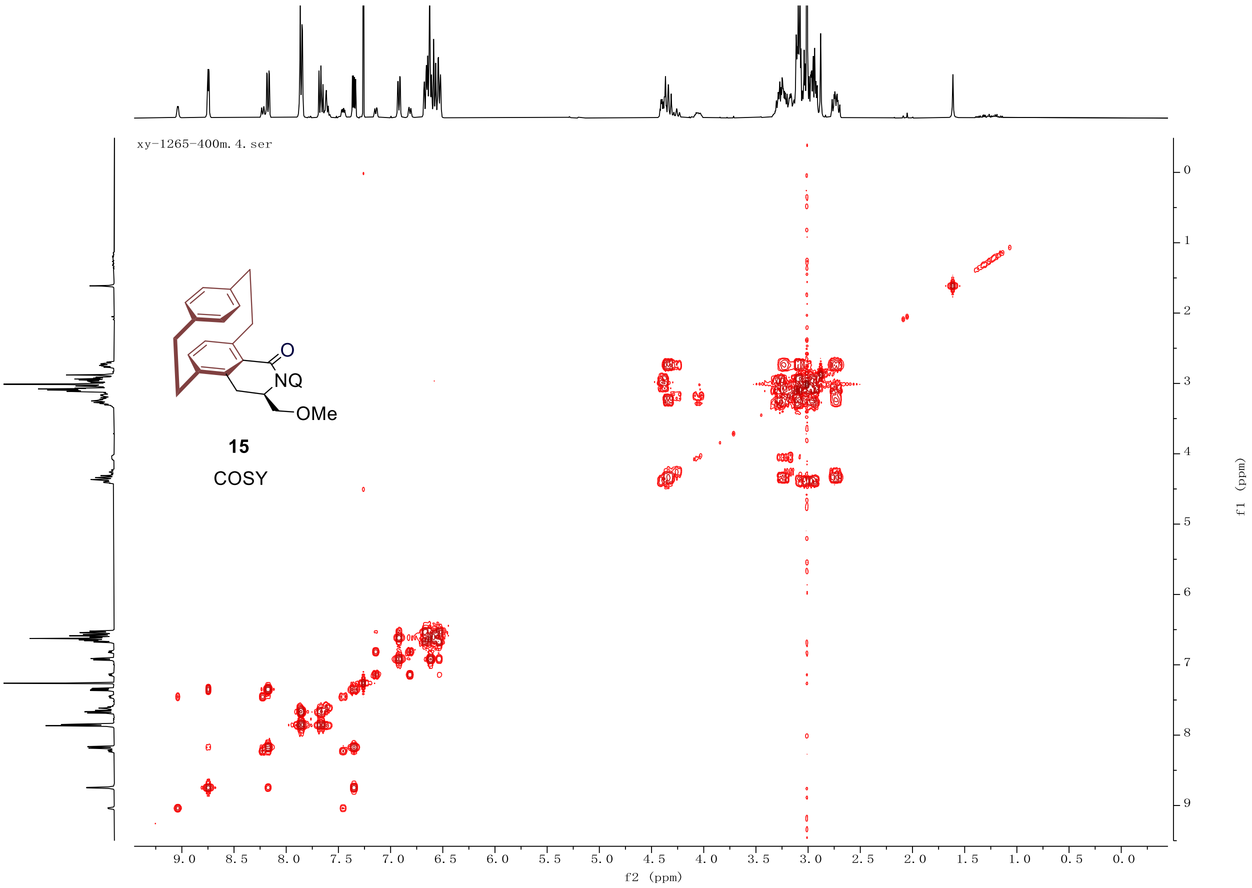

xy-1265-400m.4.ser
O
NQ
OMe
15
COSY
f1 (ppm)
f2 (ppm)

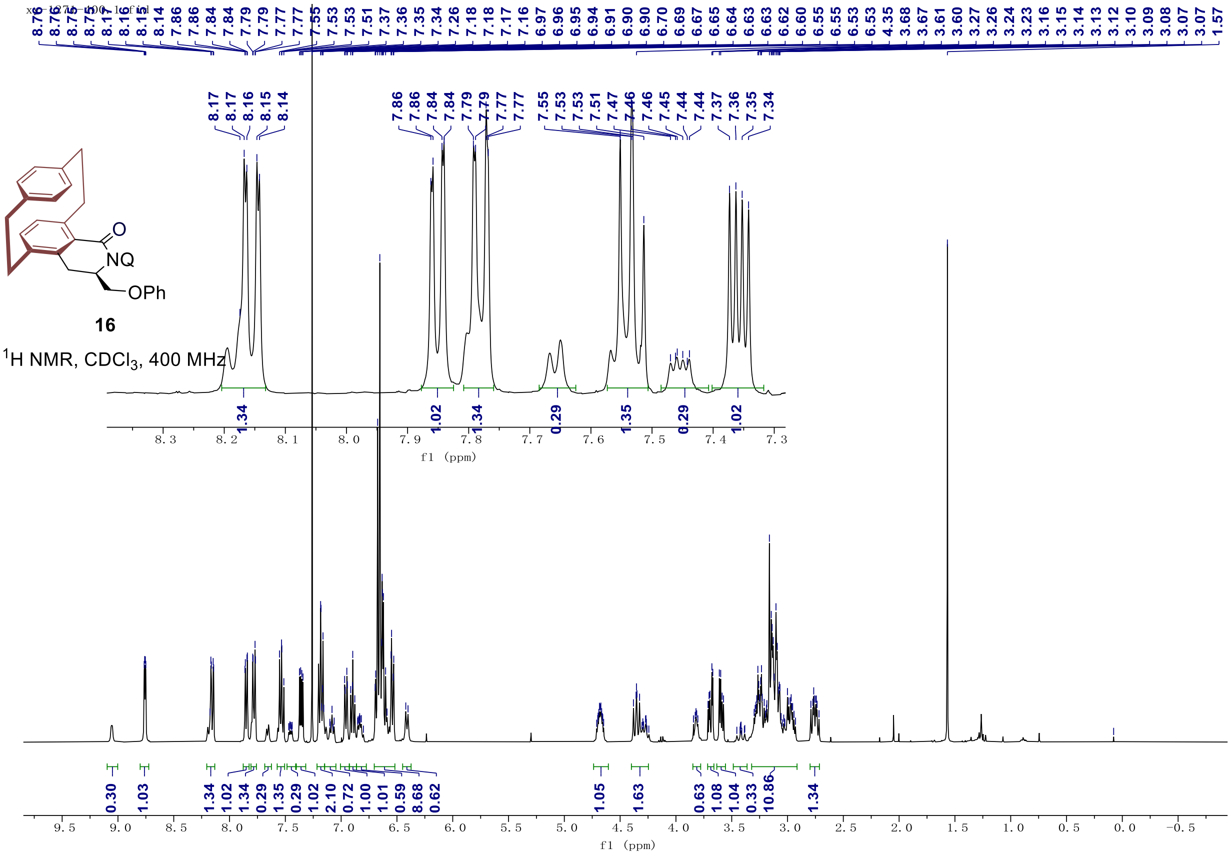
xy-1271-400.1.fid
8.76 8.75 8.75 8.75 8.17 8.16 8.15 8.15 8.14 7.86 7.86 7.84 7.84 7.79 7.79 7.77 7.77 7.55 7.53 7.53 7.51 7.37 7.36 7.35 7.34 7.26 7.18 7.18 7.17 7.16 6.97 6.96 6.95 6.94 6.91 6.90 6.90 6.70 6.69 6.67 6.65 6.64 6.63 6.63 6.62 6.60 6.55 6.55 6.53 6.53 4.35 3.68 3.67 3.61 3.60 3.27 3.26 3.24 3.23 3.16 3.15 3.14 3.13 3.12 3.10 3.09 3.08 3.07 1.57
NQ
OPh
O
16
^{1}H NMR, $CDCl_3$, 400 MHz
f1 (ppm)

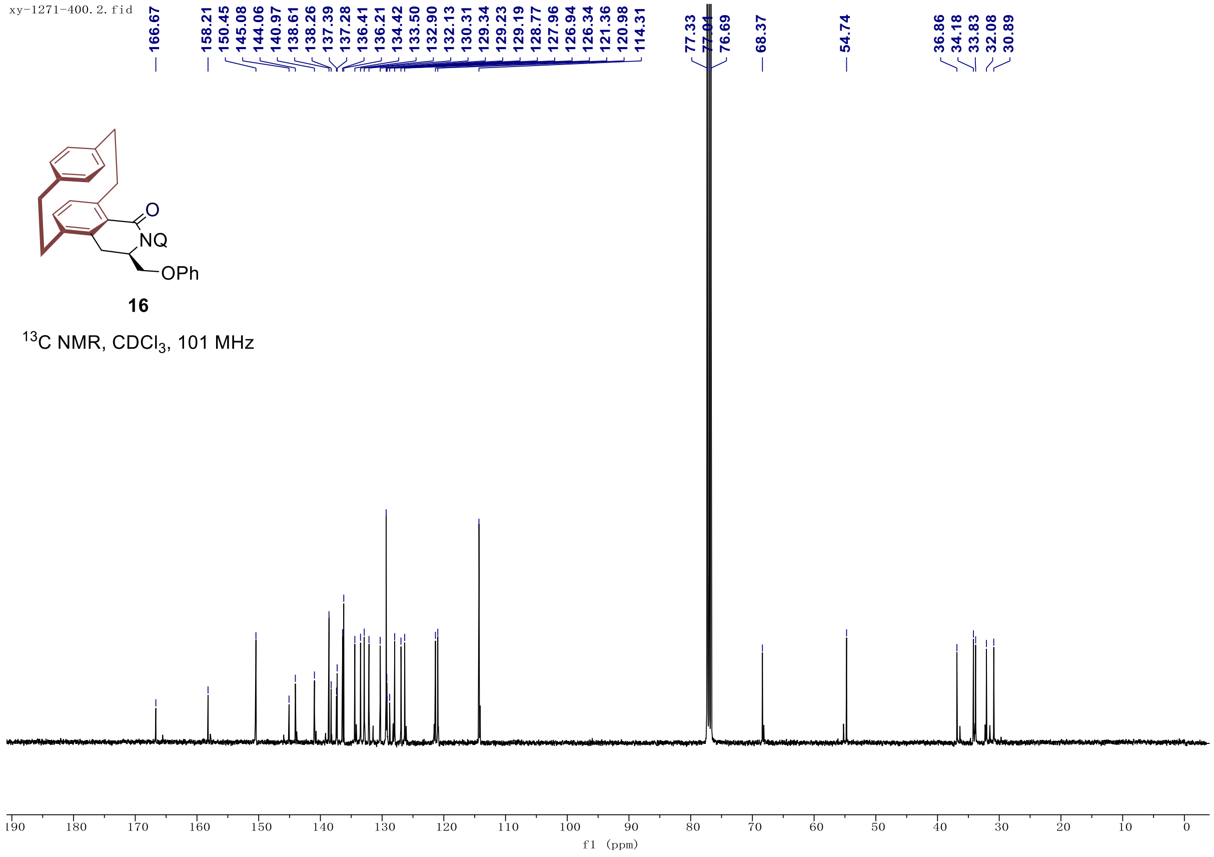
xy-1271-400.2.fid
166.67 158.21 150.45 145.08 144.06 140.97 138.61 138.26 137.39 137.28 136.41 136.21 134.42 133.50 132.90 132.13 130.31 129.34 129.23 129.19 128.77 127.96 126.94 126.34 121.36 120.98 114.31 77.33 77.01 76.69 68.37 54.74 36.86 34.18 33.83 32.08 30.89
NQ
OPh
O
16
^{13}C NMR, $CDCl_3$, 101 MHz
f1 (ppm)

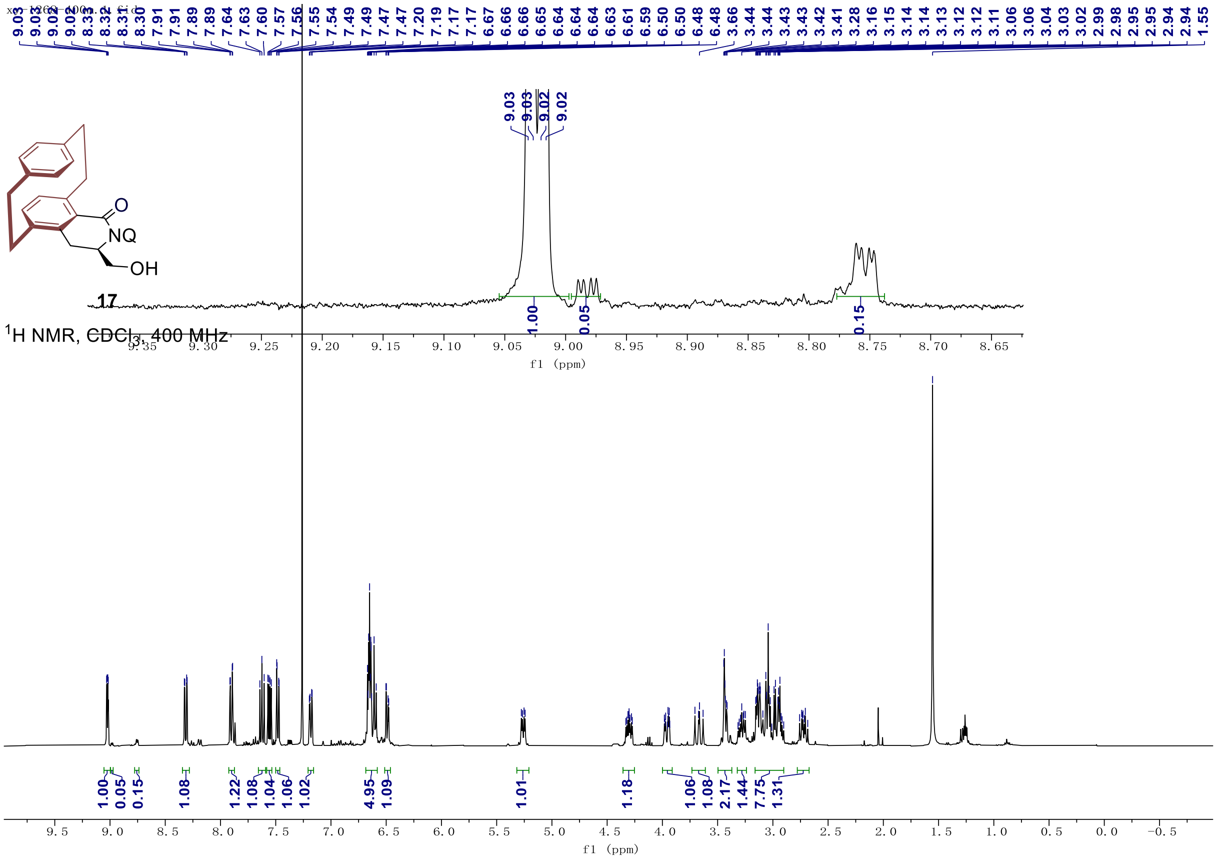

^{1}H NMR, $CDCl_3$, 400 MHz

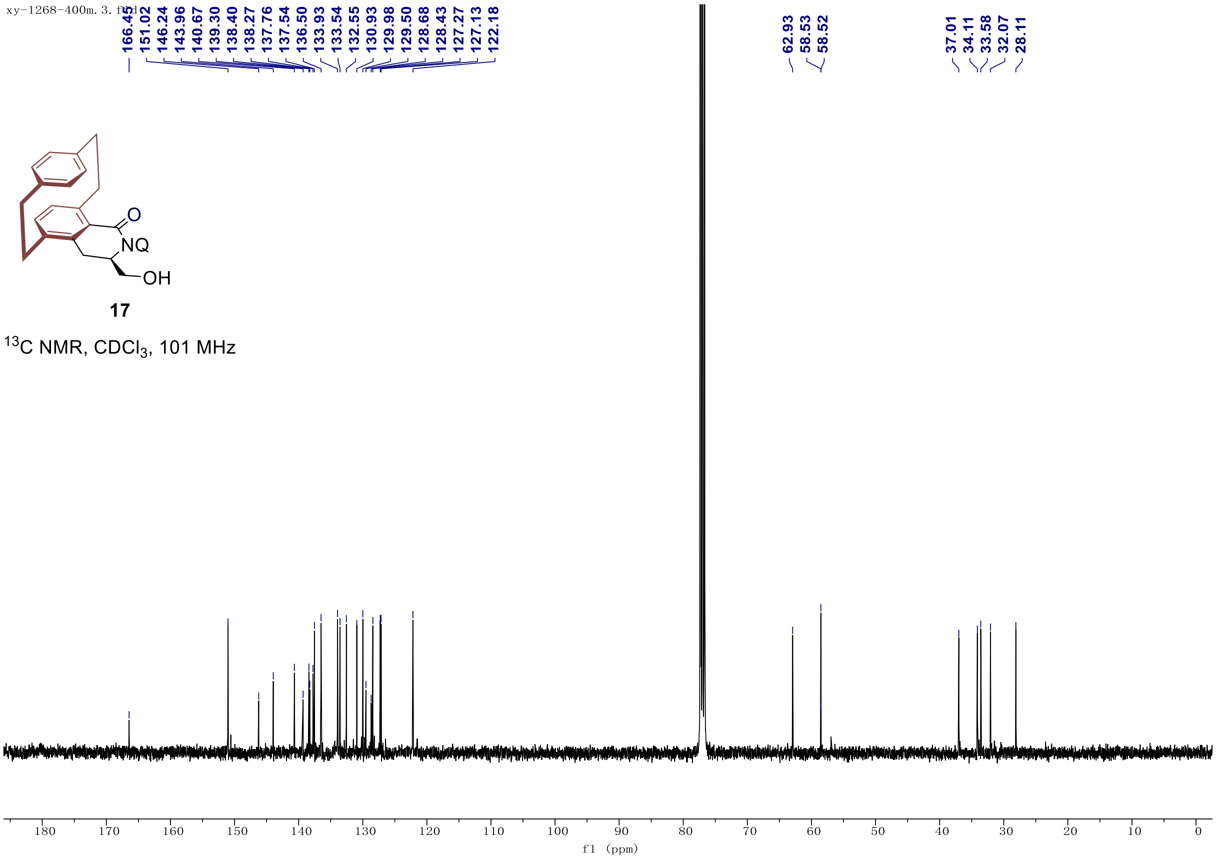

^{13}C NMR, $CDCl_3$, 101 MHz

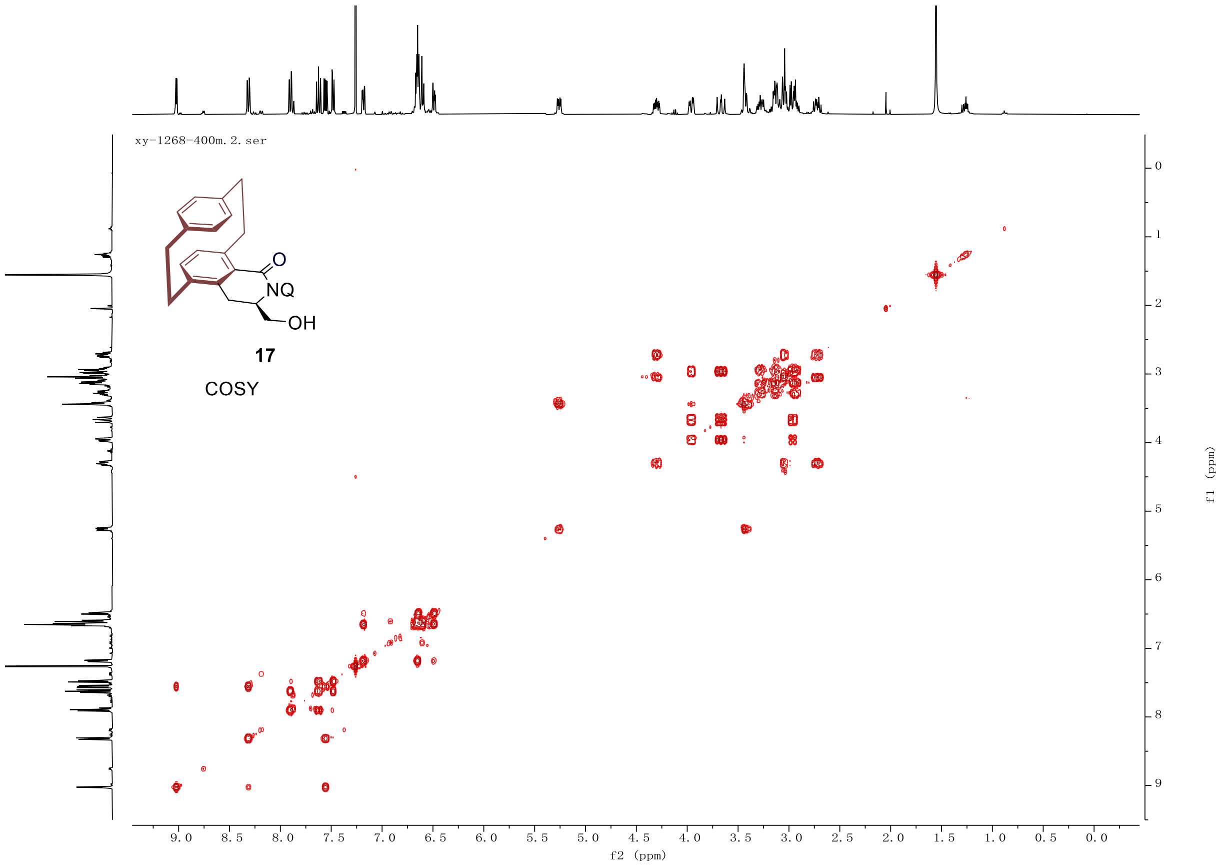
xy-1268-400m.2.ser
17
COSY
f1 (ppm)
f2 (ppm)

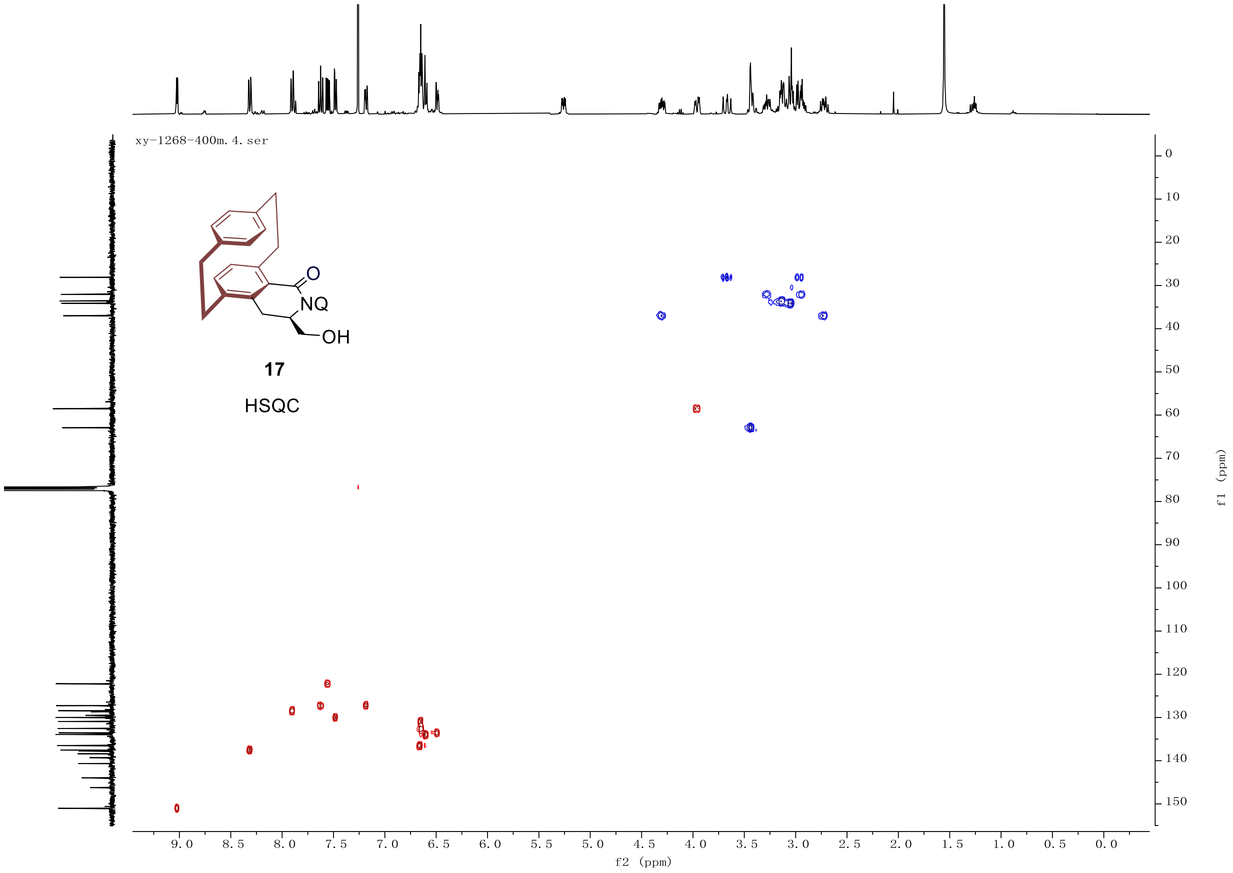
xy-1268-400m.4.ser
17
HSQC
f1 (ppm)
f2 (ppm)

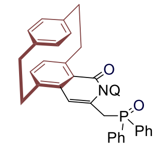

18

^{1}H-NMR, $CDCl_3$, 300 MHz

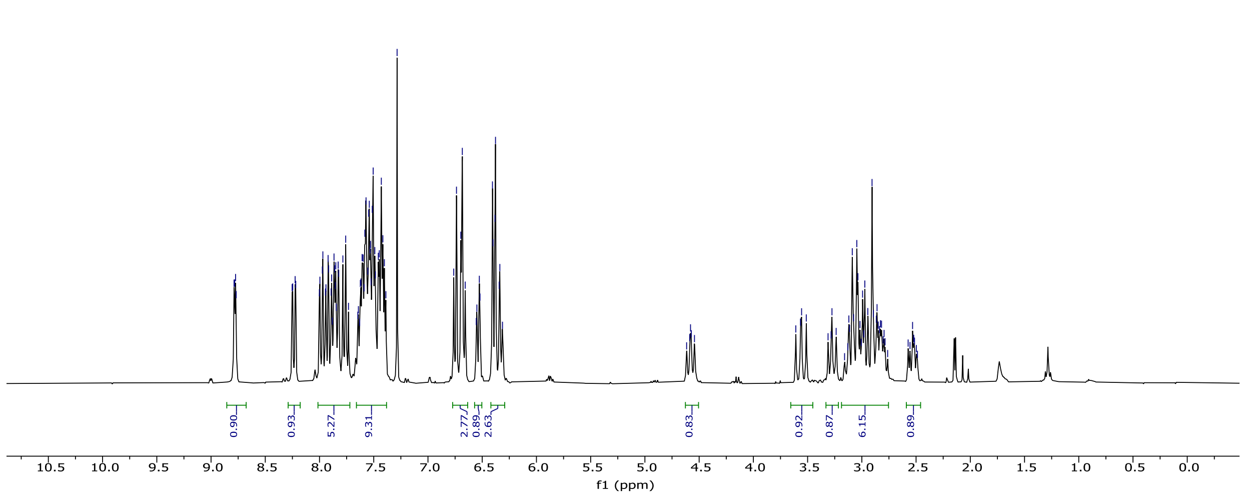

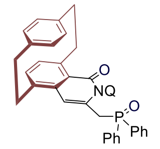

18

^{13}C-NMR, $CDCl_3$, 75 MHz

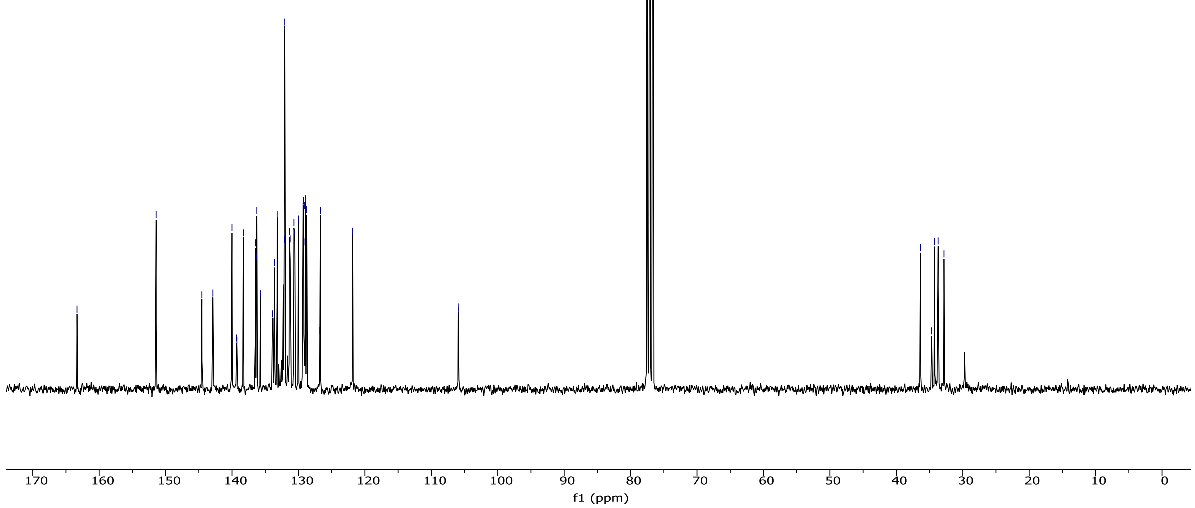

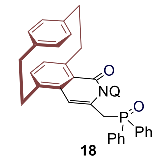

18

^{31}P-NMR, $CDCl_3$, 121 MHz

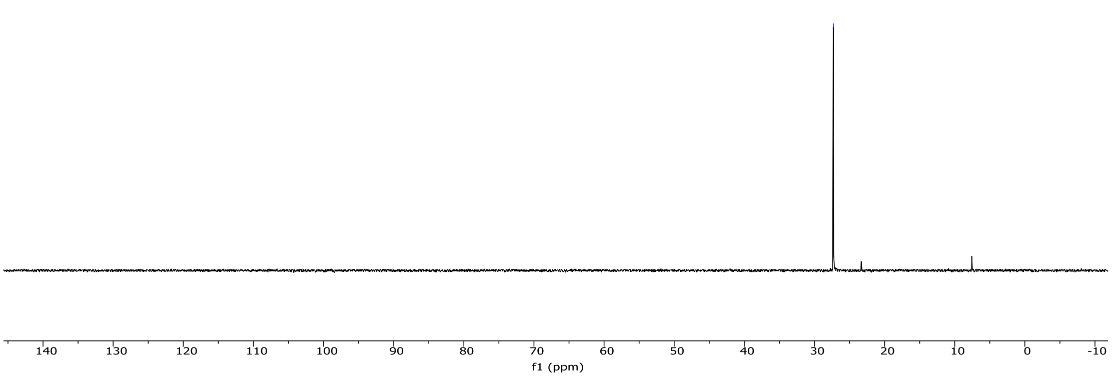

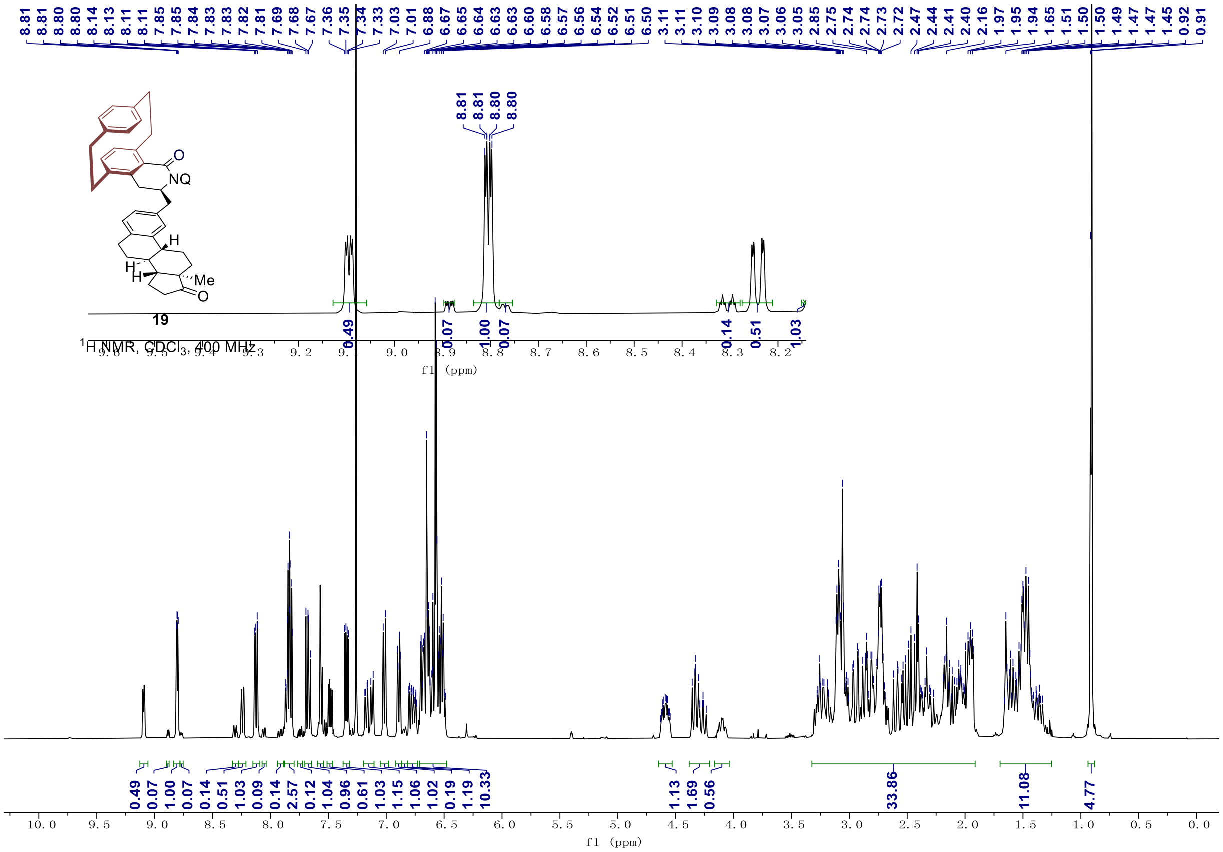

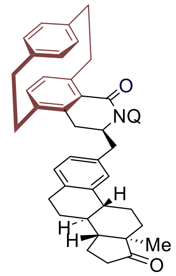

19

^{13}C NMR, $CDCl_3$, 101 MHz

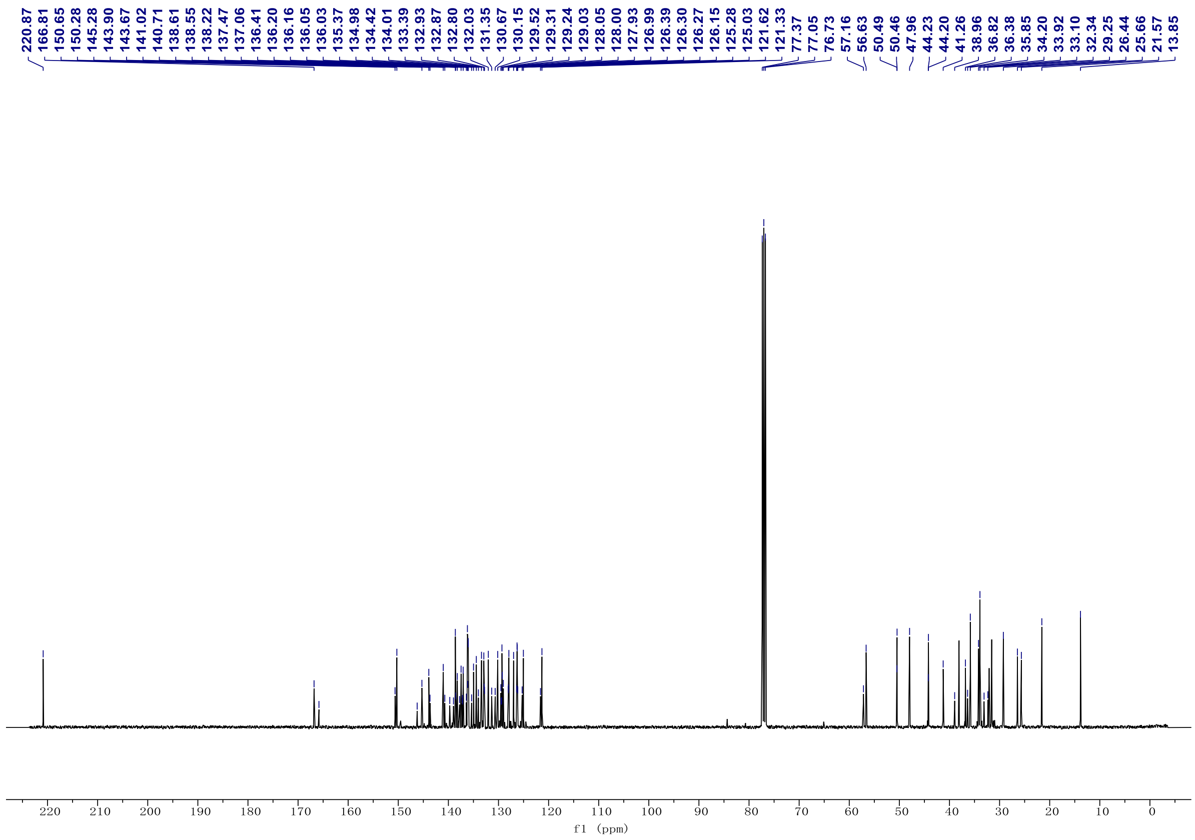
220.87
166.81
150.65
150.28
145.28
143.90
143.67
141.02
140.71
138.61
138.55
138.22
137.47
137.06
136.41
136.20
136.16
136.05
136.03
135.37
134.98
134.42
134.01
133.39
132.93
132.87
132.80
132.03
131.35
130.67
130.15
129.52
129.31
129.24
129.03
128.05
128.00
127.93
126.99
126.39
126.30
126.27
126.15
125.28
125.03
121.62
121.33
77.37
77.05
76.73
57.16
56.63
50.49
50.46
47.96
44.23
44.20
41.26
38.96
36.82
36.38
35.85
34.20
33.92
33.10
32.34
29.25
26.44
25.66
21.57
13.85
f1 (ppm)

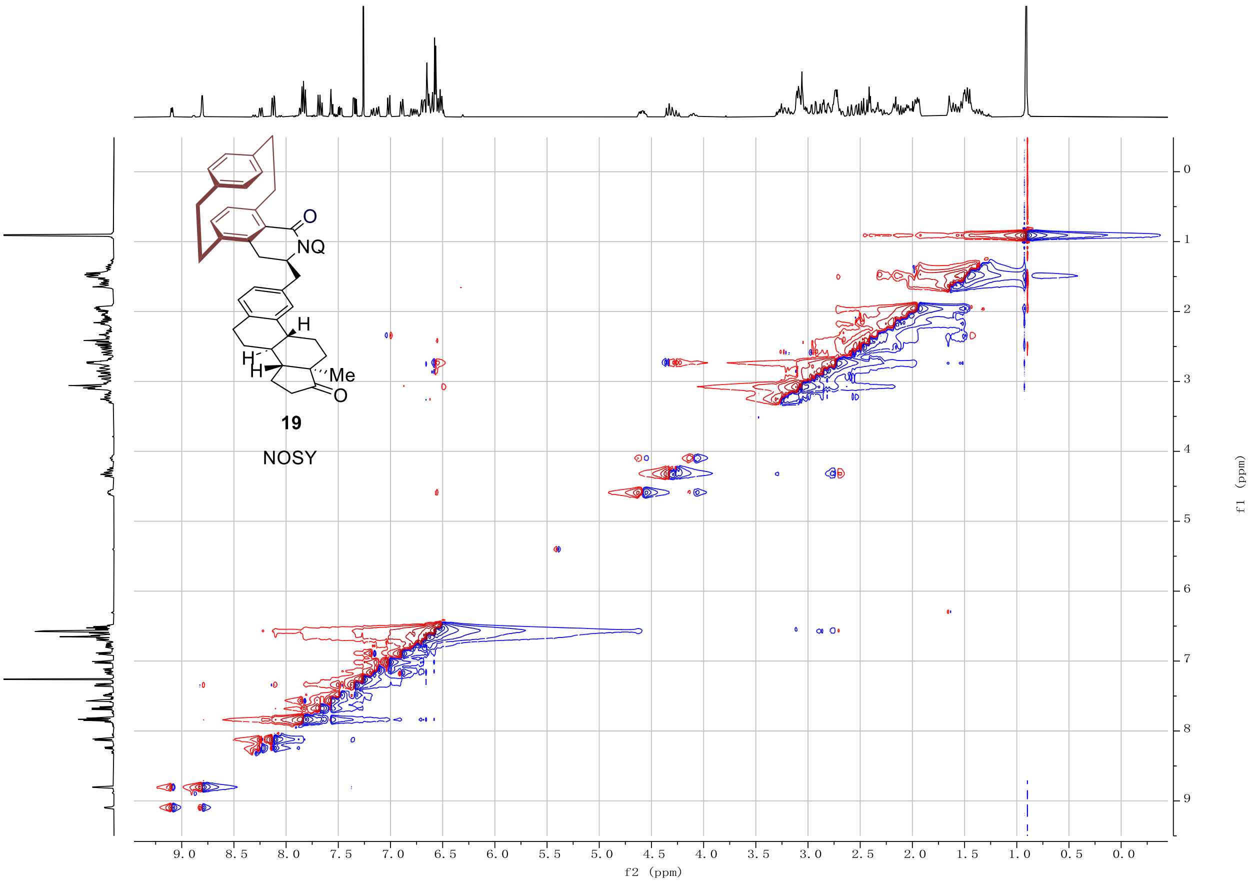
O
NQ
H
H
H
Me
O
19
NOSY
f2 (ppm)
f1 (ppm)

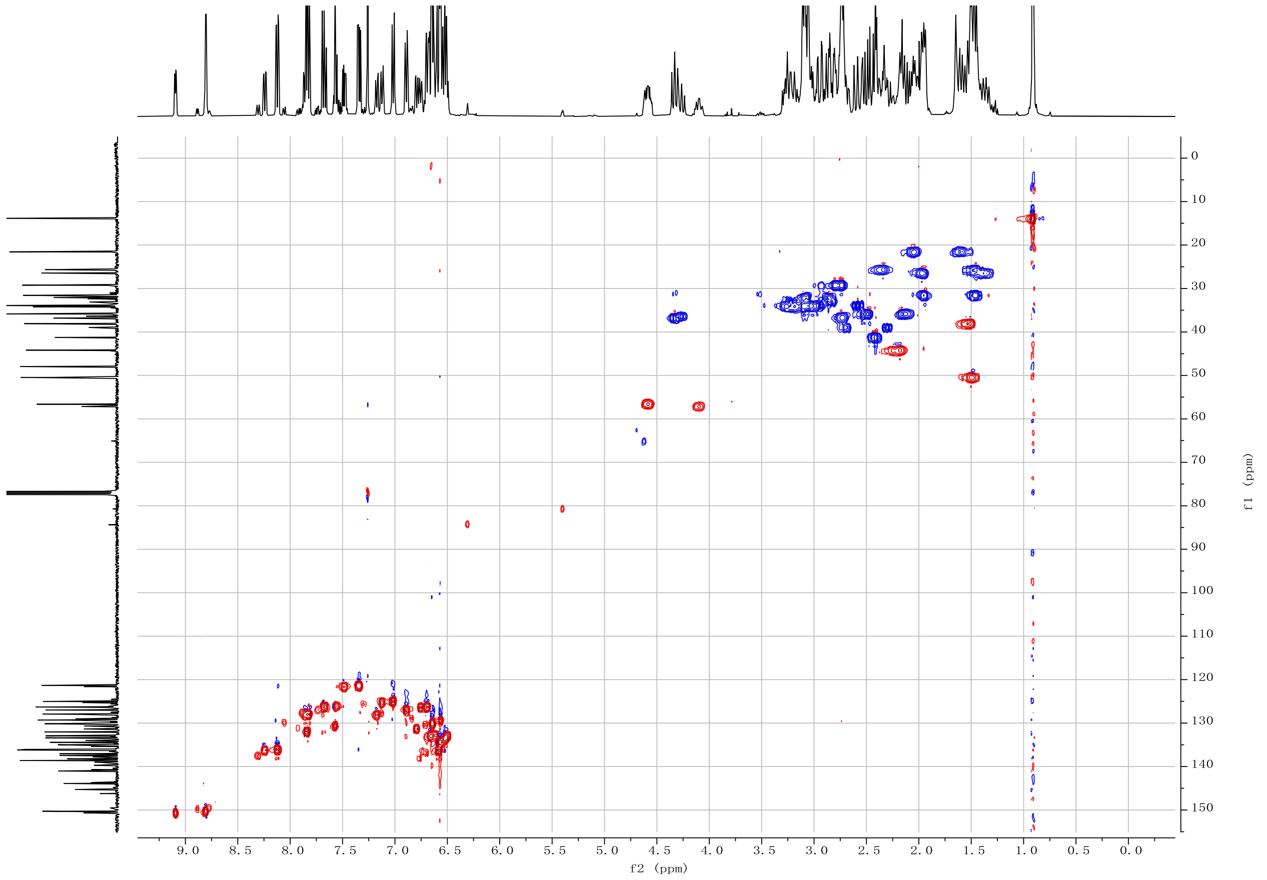
f2 (ppm)
f1 (ppm)

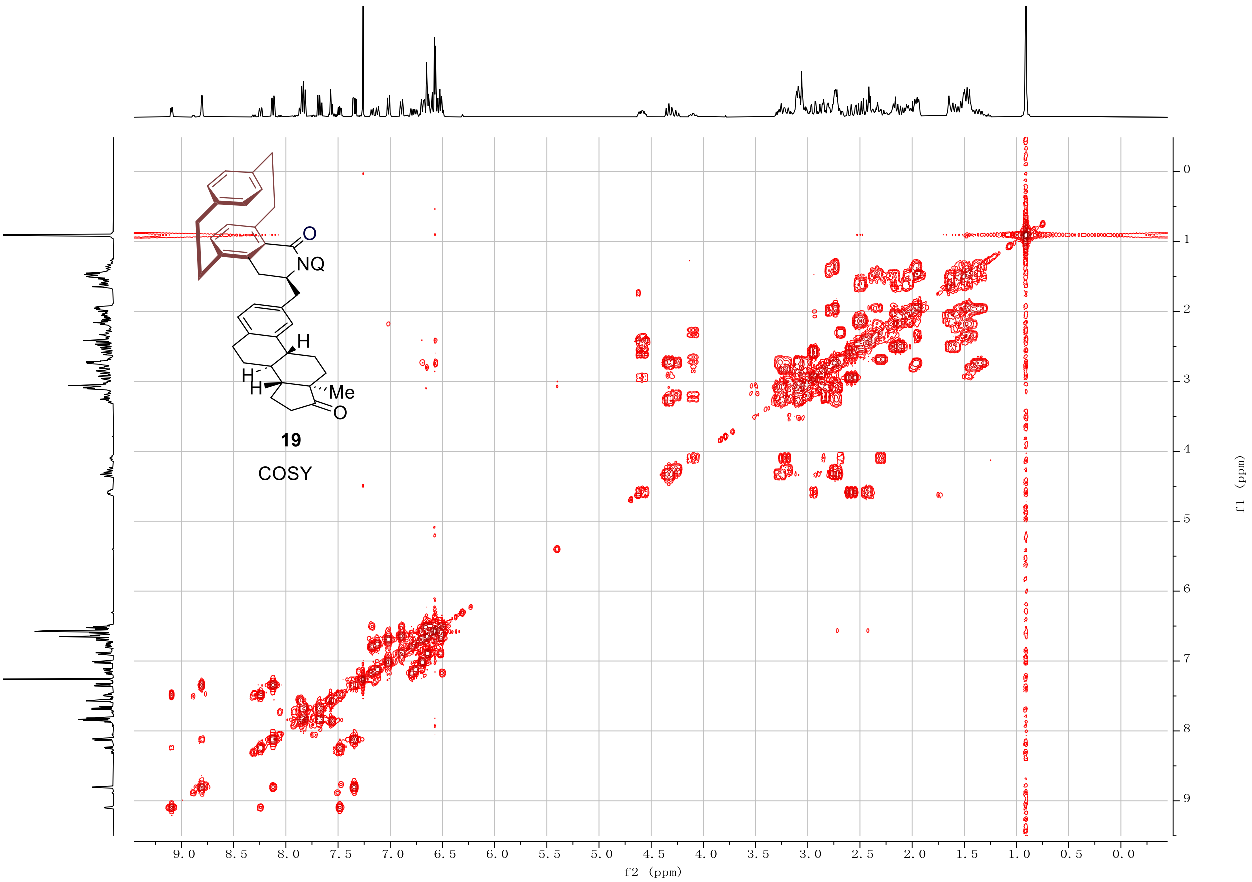

O
NQ
H
H
H
Me
O
19
COSY
f2 (ppm)
f1 (ppm)

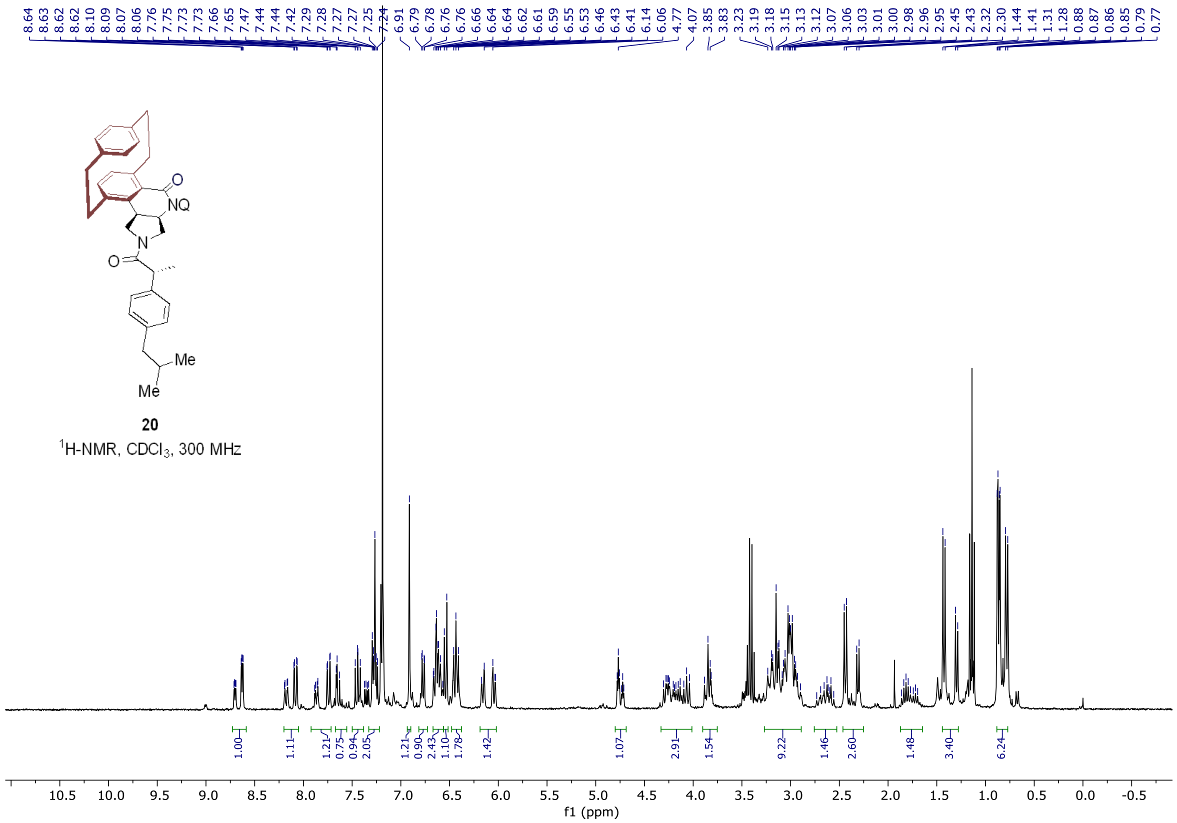
8.64 8.63 8.62 8.10 8.09 8.07 8.06 7.76 7.75 7.73 7.73 7.66 7.65 7.47 7.44 7.44 7.42 7.29 7.28 7.27 7.27 7.25 7.24 6.91 6.79 6.78 6.76 6.76 6.66 6.64 6.64 6.62 6.61 6.59 6.55 6.53 6.46 6.43 6.41 6.14 6.06 4.77 4.07 3.85 3.83 3.23 3.19 3.18 3.15 3.13 3.12 3.07 3.06 3.03 3.01 3.00 2.98 2.96 2.95 2.45 2.43 2.32 2.30 1.44 1.41 1.31 1.28 0.88 0.87 0.86 0.85 0.79 0.77
O
NQ
N
O
Me
Me
20
1H-NMR, CDCl3, 300 MHz
1.00 1.11 1.21 0.75 0.94 2.05 1.21 0.90 2.43 1.10 1.78 1.42 1.07 2.91 1.54 9.22 1.46 2.60 1.48 3.40 6.24
10.5 10.0 9.5 9.0 8.5 8.0 7.5 7.0 6.5 6.0 5.5 5.0 4.5 4.0 3.5 3.0 2.5 2.0 1.5 1.0 0.5 0.0 -0.5
f1 (ppm)

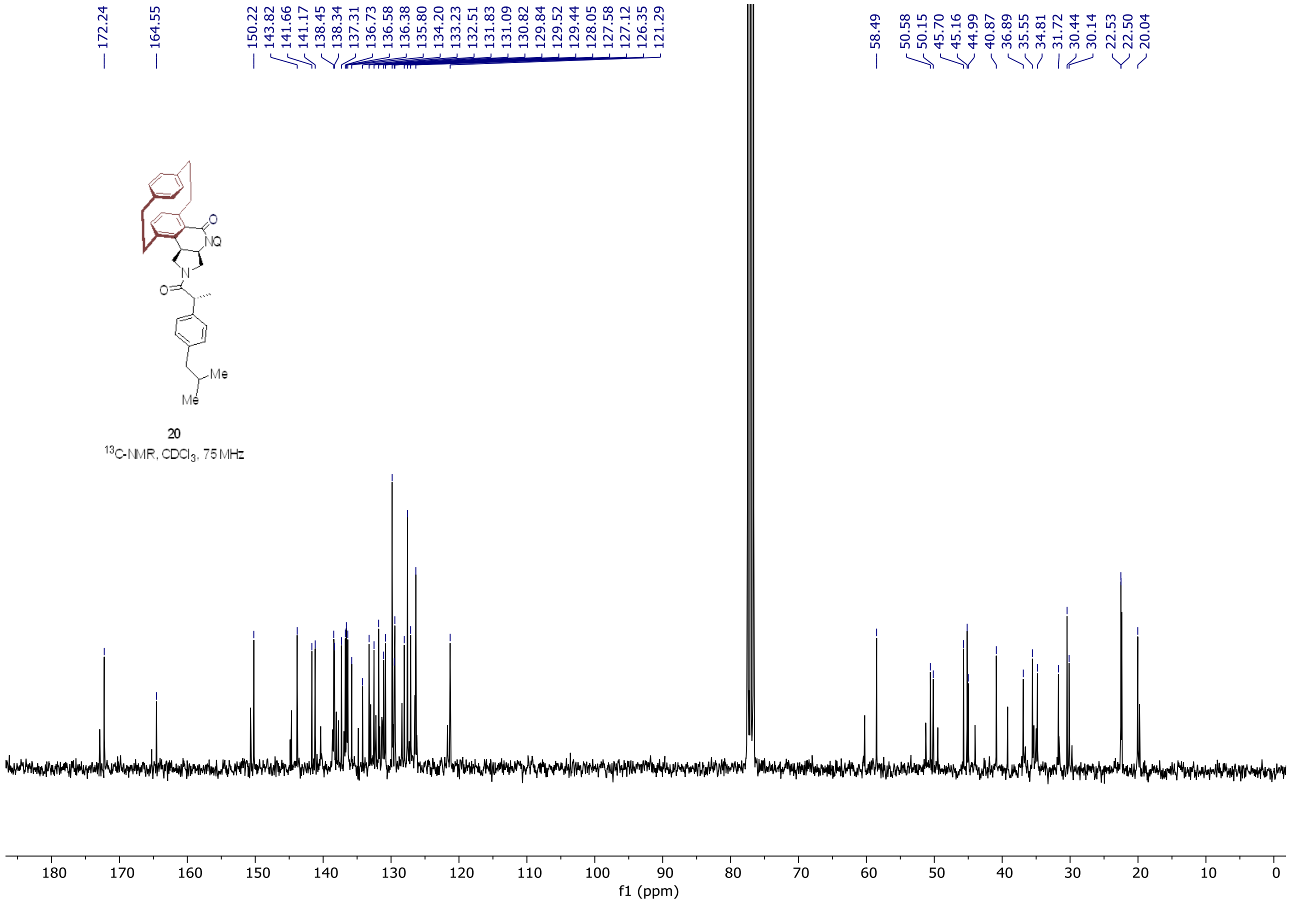
172.24 164.55 150.22 143.82 141.66 141.17 138.45 138.34 137.31 136.73 136.58 136.38 135.80 134.20 133.23 132.51 131.83 131.09 130.82 129.84 129.52 129.44 128.05 127.58 127.12 126.35 121.29 58.49 50.58 50.15 45.70 45.16 44.99 40.87 36.89 35.55 34.81 31.72 30.44 30.14 22.53 22.50 20.04
O
NQ
N
O
Me
Me
20
13C-NMR, CDCl3, 75 MHz
180 170 160 150 140 130 120 110 100 90 80 70 60 50 40 30 20 10 0
f1 (ppm)

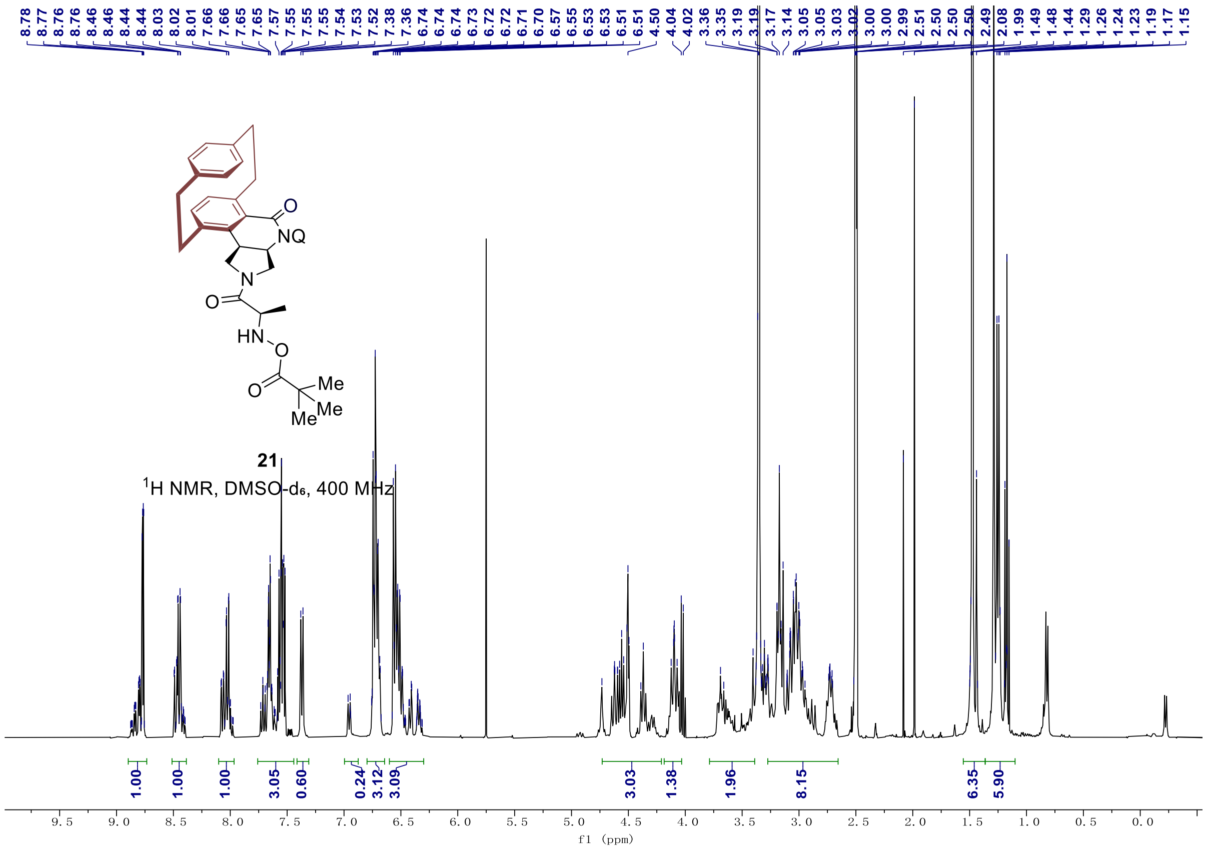
21
^{1}H NMR, DMSO-d6, 400 MHz

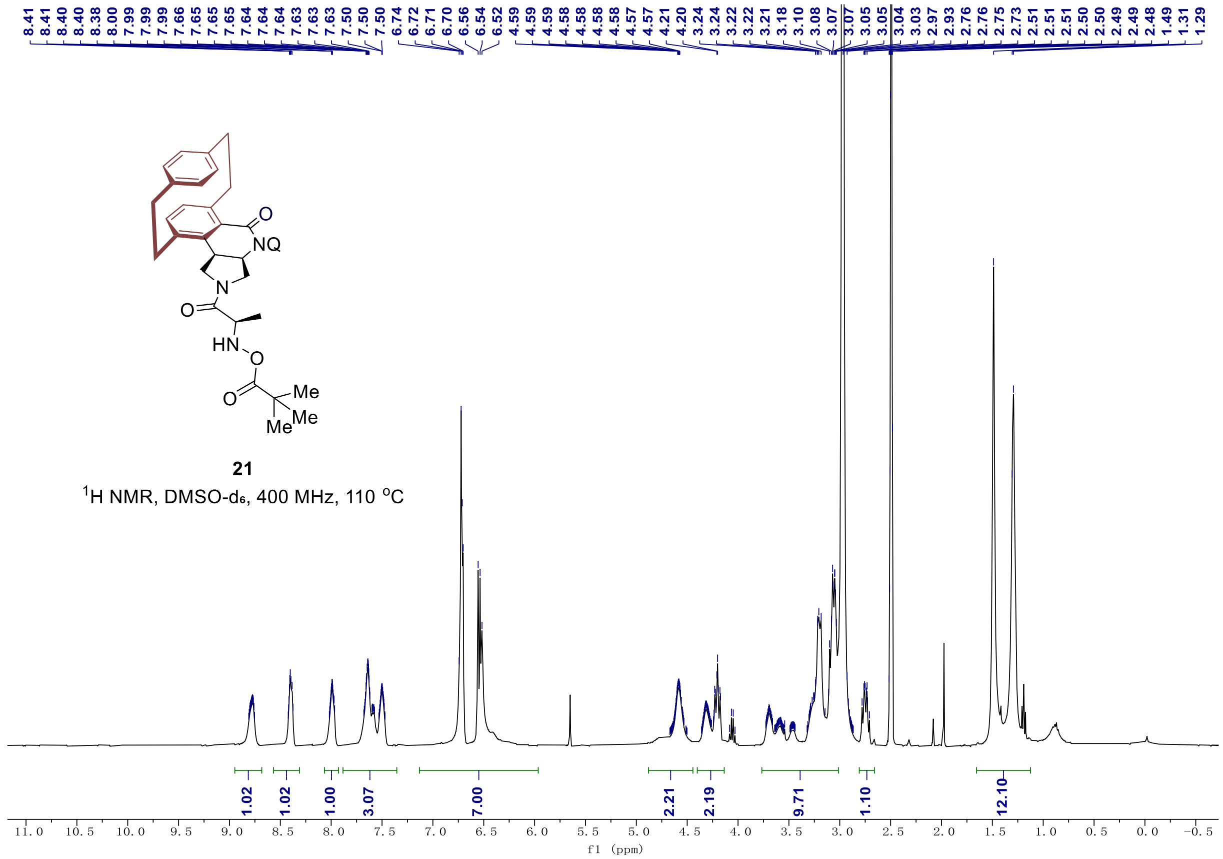
21
^{1}H NMR, DMSO-d6, 400 MHz, 110 °C

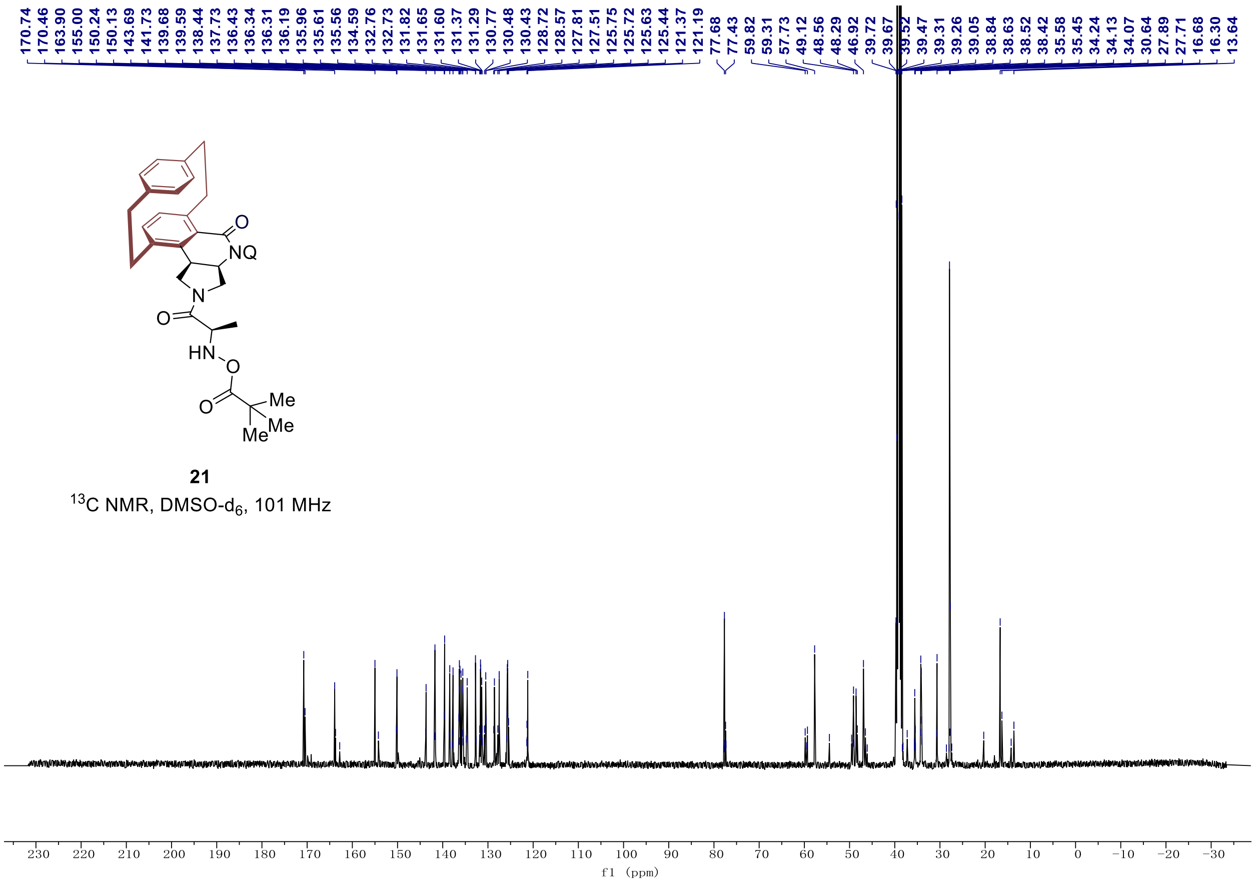
170.74
170.46
163.90
155.00
150.24
150.13
143.69
141.73
139.68
139.59
138.44
137.73
136.43
136.34
136.31
136.19
135.96
135.61
135.56
134.59
132.76
132.73
131.82
131.65
131.60
131.37
131.29
130.77
130.48
130.43
128.72
128.57
127.81
127.51
125.75
125.72
125.63
125.44
121.37
121.19
77.68
77.43
59.82
59.31
57.73
49.12
48.56
48.29
46.92
39.72
39.67
39.52
39.47
39.31
39.26
39.05
38.84
38.63
38.52
38.42
35.58
35.45
34.24
34.13
34.07
30.64
27.89
27.71
16.68
16.30
13.64
O
NQ
N
O
HN
O
O
Me
Me
Me
21
^{13}C NMR, DMSO-d$_6$, 101 MHz
f1 (ppm)

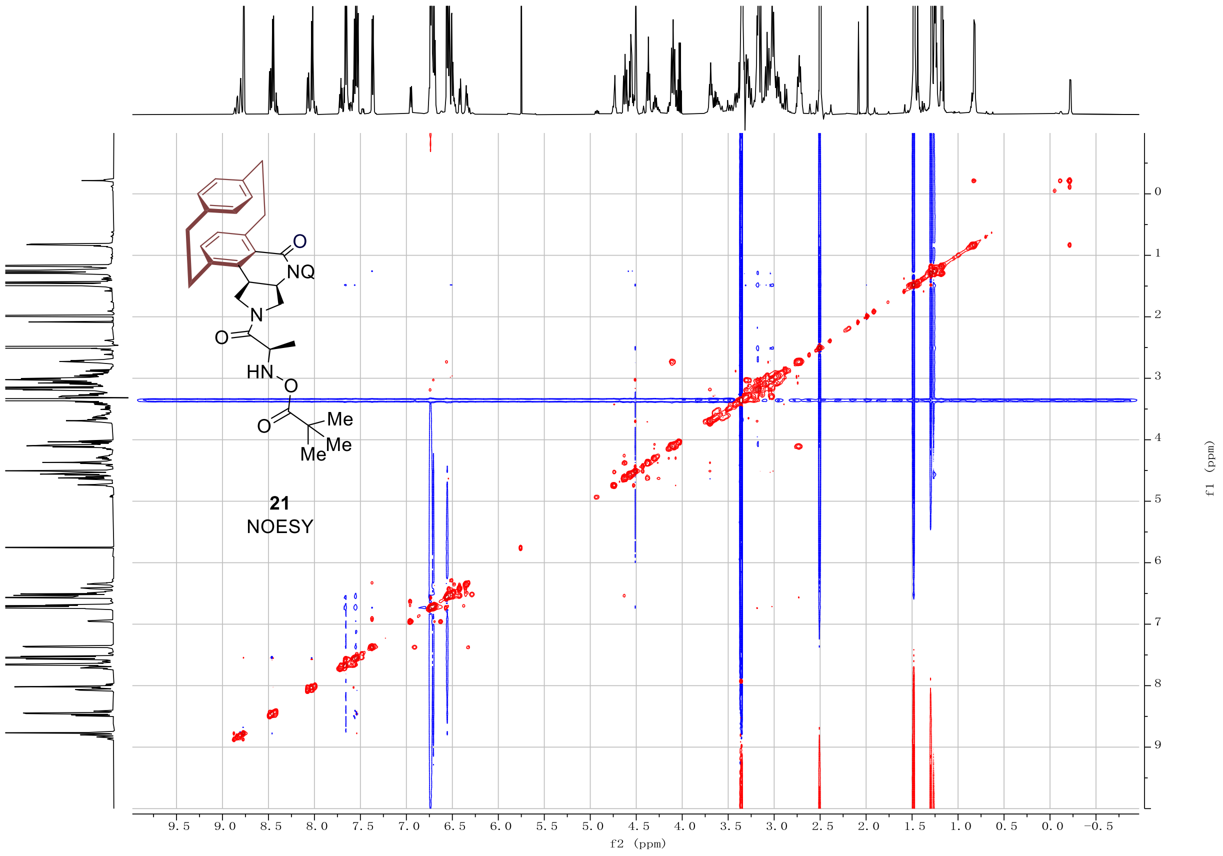
O
NQ
N
O
HN
O
O
Me
Me
Me
21
NOESY
f2 (ppm)
f1 (ppm)

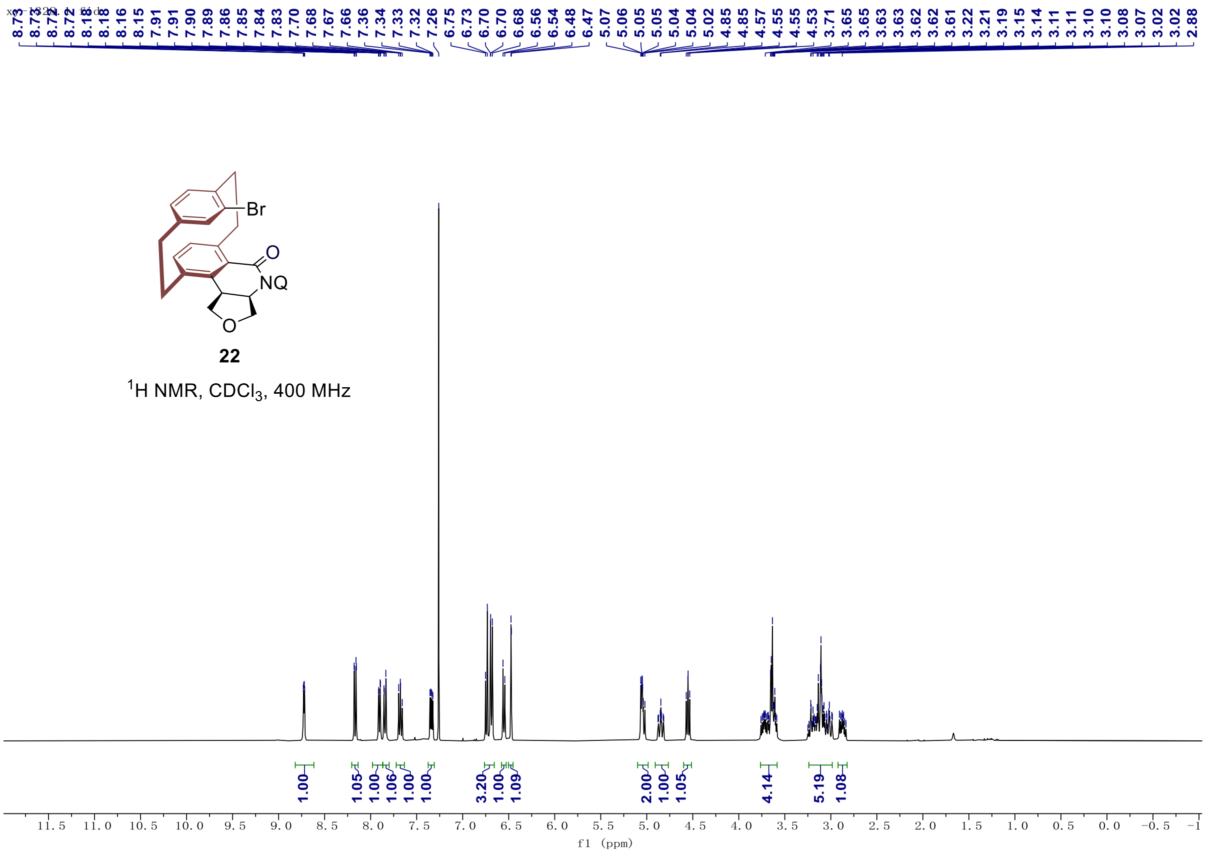

22

^{1}H NMR, $CDCl_3$, 400 MHz

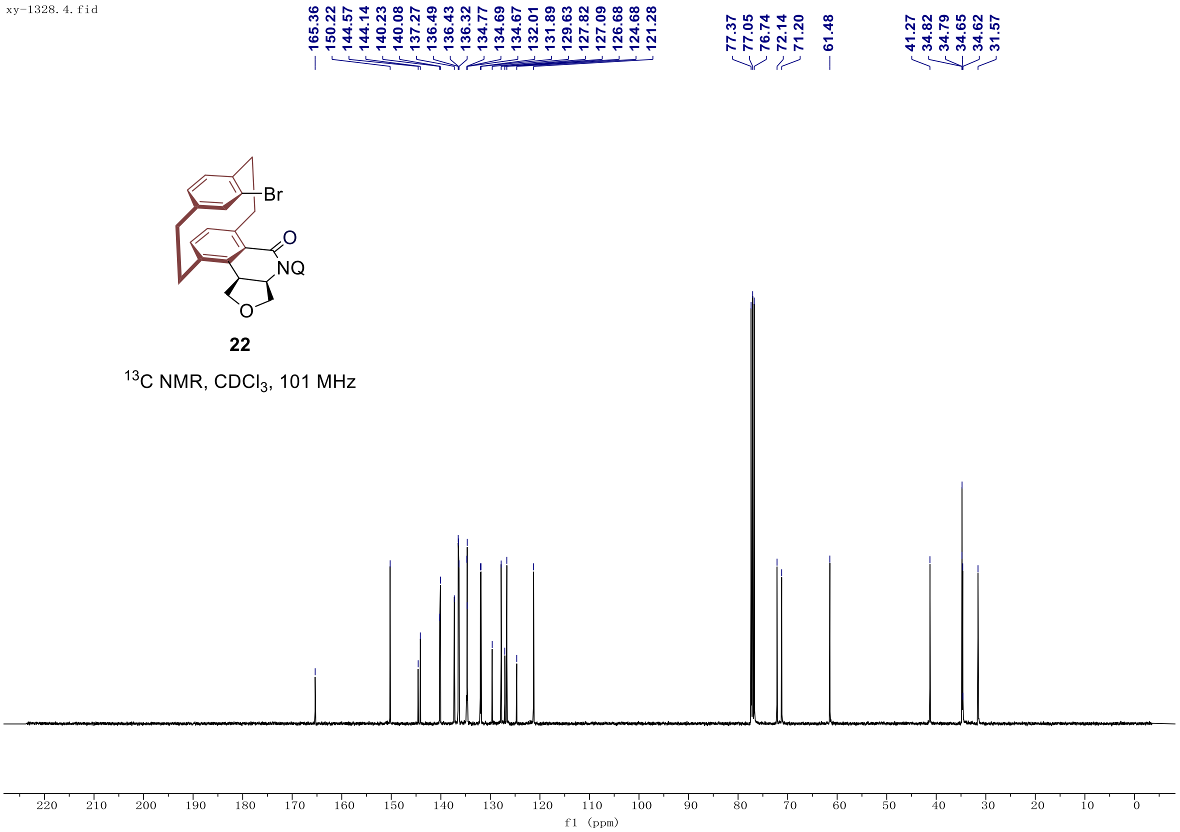

22

^{13}C NMR, $CDCl_3$, 101 MHz

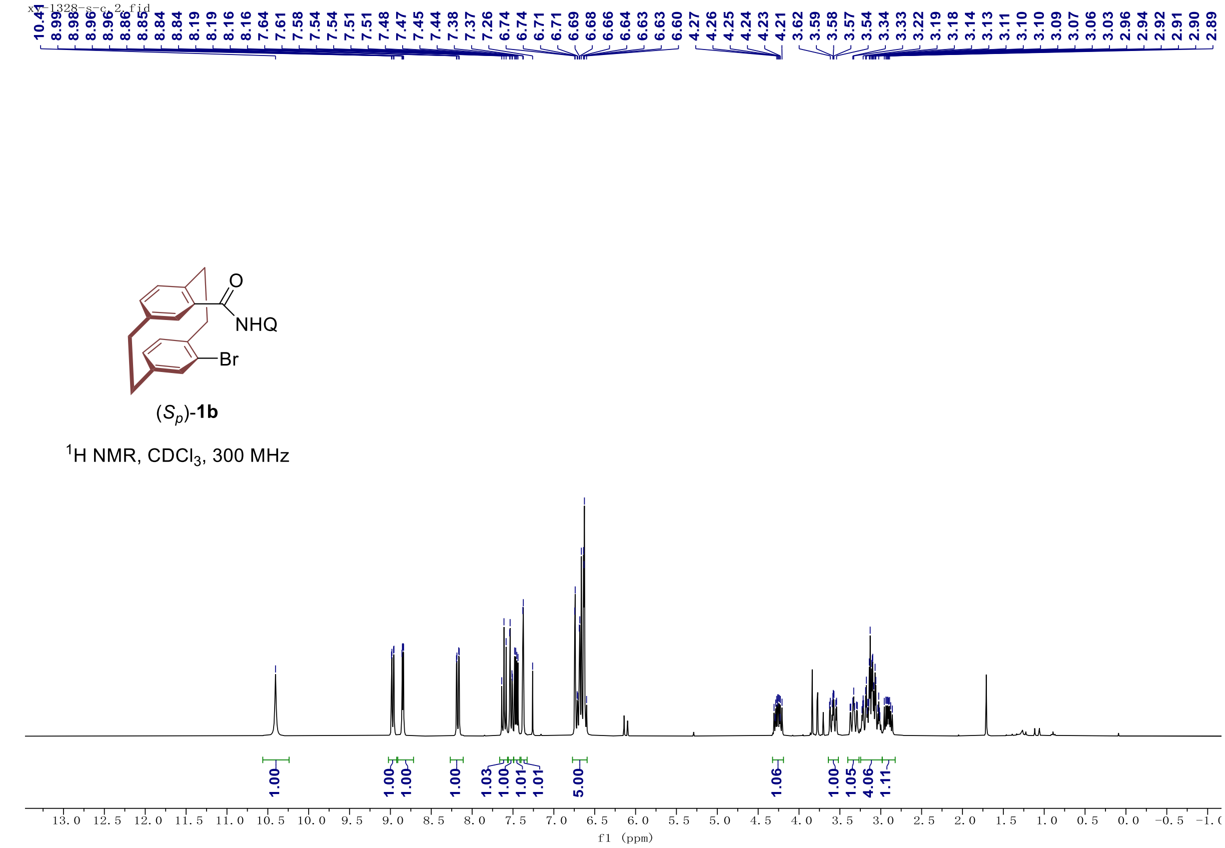
xy-1328-s-c.2.fid
10.41 8.99 8.98 8.96 8.96 8.86 8.85 8.84 8.84 8.19 8.19 8.16 8.16 7.64 7.61 7.58 7.54 7.54 7.51 7.51 7.48 7.47 7.45 7.44 7.38 7.37 7.26 6.74 6.74 6.71 6.71 6.69 6.68 6.66 6.64 6.63 6.60 4.27 4.26 4.25 4.24 4.23 4.21 3.62 3.59 3.58 3.57 3.54 3.34 3.33 3.22 3.19 3.18 3.14 3.13 3.11 3.10 3.09 3.07 3.06 3.03 2.96 2.94 2.92 2.91 2.90 2.89
O
NHQ
Br
(S_p)-**1b**
^{1}H NMR, $CDCl_3$, 300 MHz
1.00 1.00 1.00 1.00 1.03 1.00 1.01 1.01 5.00 1.06 1.00 1.05 4.06 1.11
f1 (ppm)

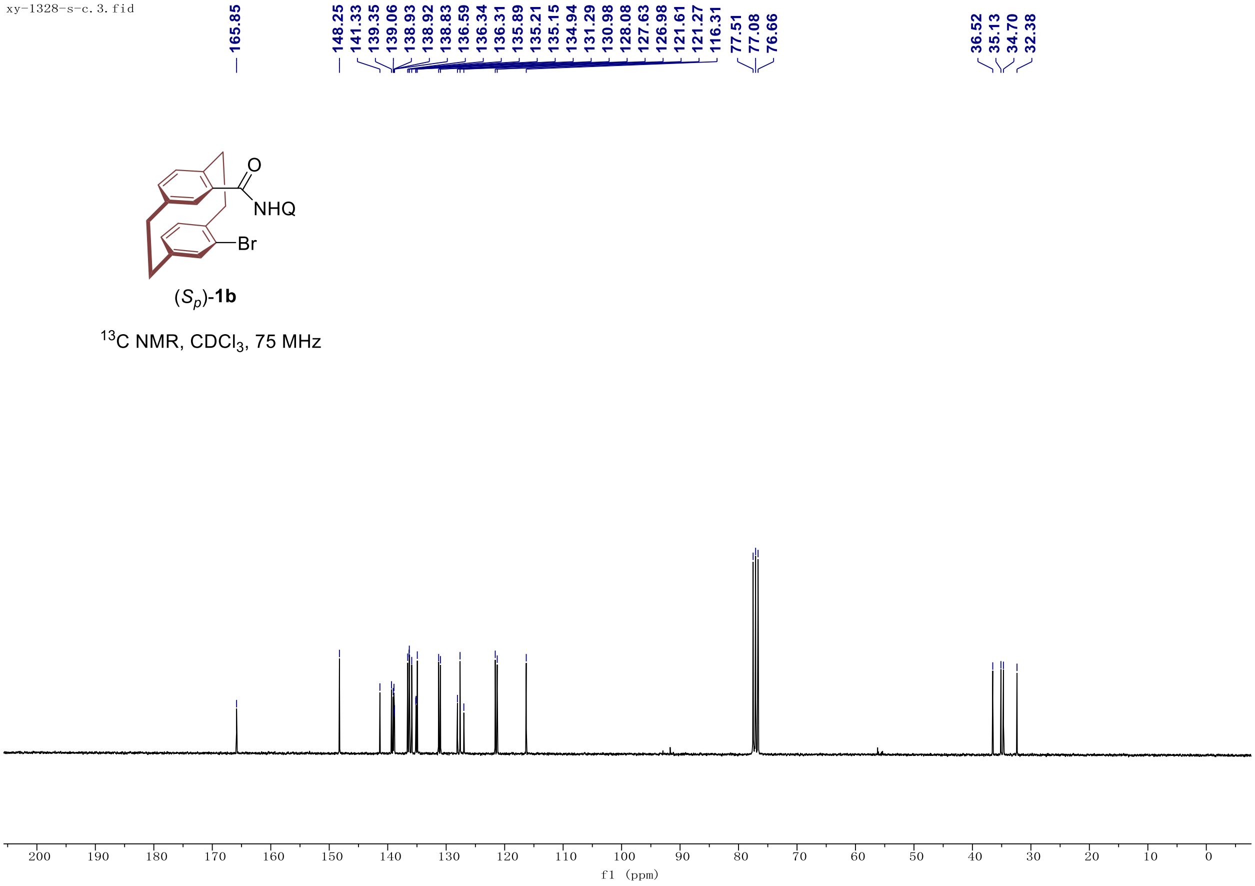
xy-1328-s-c.3.fid
165.85 148.25 141.33 139.35 139.06 138.93 138.83 136.59 136.34 136.31 135.89 135.21 135.15 134.94 131.29 130.98 128.08 127.63 126.98 121.61 121.27 116.31 77.51 77.08 76.66 36.52 35.13 34.70 32.38
O
NHQ
Br
(S_p)-**1b**
^{13}C NMR, $CDCl_3$, 75 MHz
f1 (ppm)

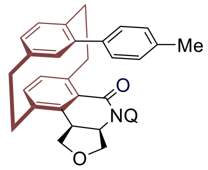

23

^{1}H NMR, $CDCl_3$, 400 MHz

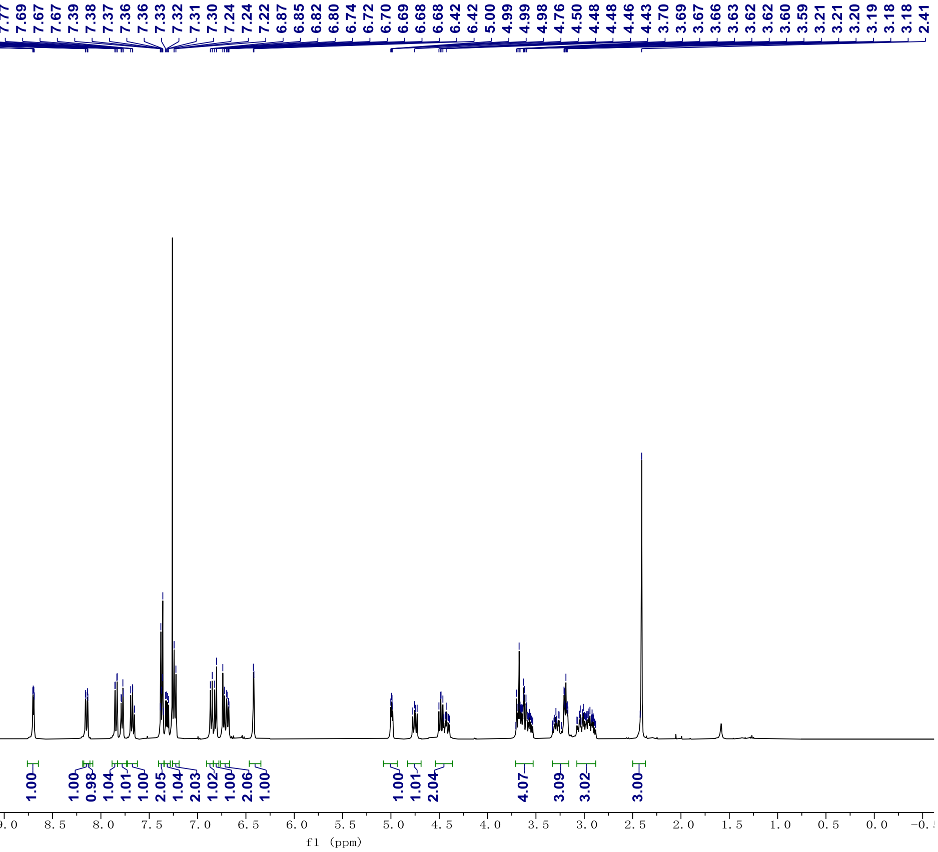

xy-1330.4.fid

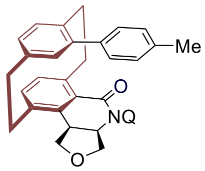

23

^{13}C NMR, $CDCl_3$, 101 MHz

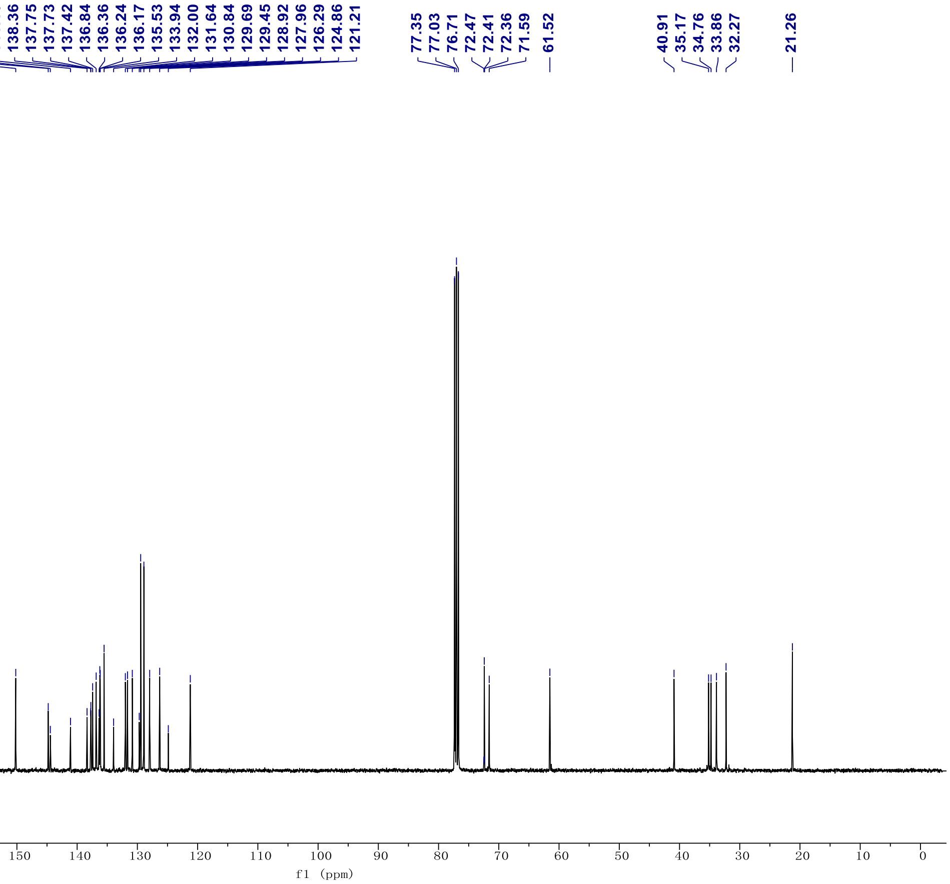

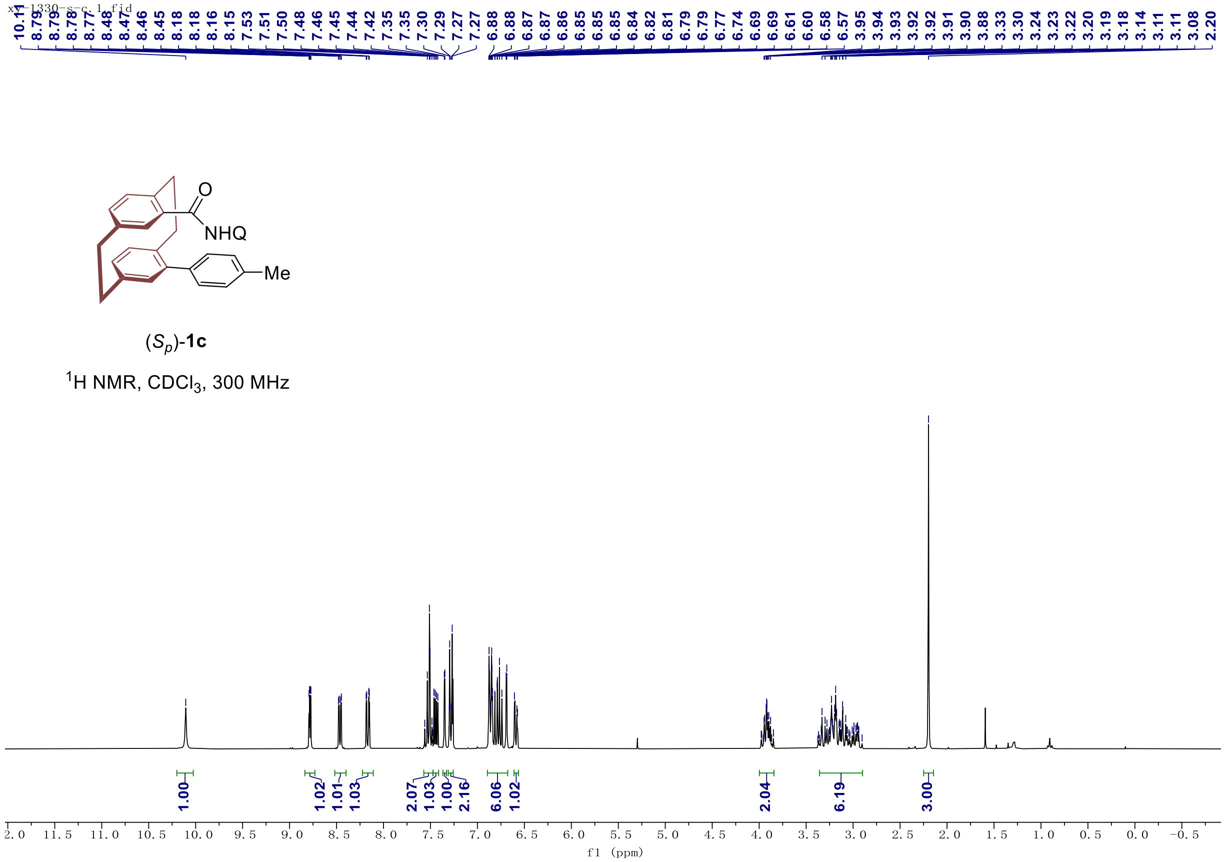
xy-1330-s-c.1.fid
10.11 8.79 8.79 8.78 8.77 8.48 8.47 8.46 8.45 8.18 8.18 8.16 8.15 7.53 7.51 7.50 7.48 7.46 7.45 7.44 7.42 7.35 7.30 7.29 7.27 7.27 6.88 6.88 6.87 6.86 6.85 6.85 6.84 6.82 6.81 6.79 6.77 6.74 6.69 6.69 6.61 6.60 6.58 6.57 3.95 3.94 3.93 3.92 3.92 3.91 3.90 3.88 3.33 3.30 3.24 3.23 3.22 3.20 3.19 3.18 3.14 3.11 3.11 3.08 2.20
O
NHQ
Me
(S_p)-**1c**
^{1}H NMR, $CDCl_3$, 300 MHz
1.00 1.02 1.01 1.03 2.07 1.03 1.00 2.16 6.06 1.02 2.04 6.19 3.00
f1 (ppm)

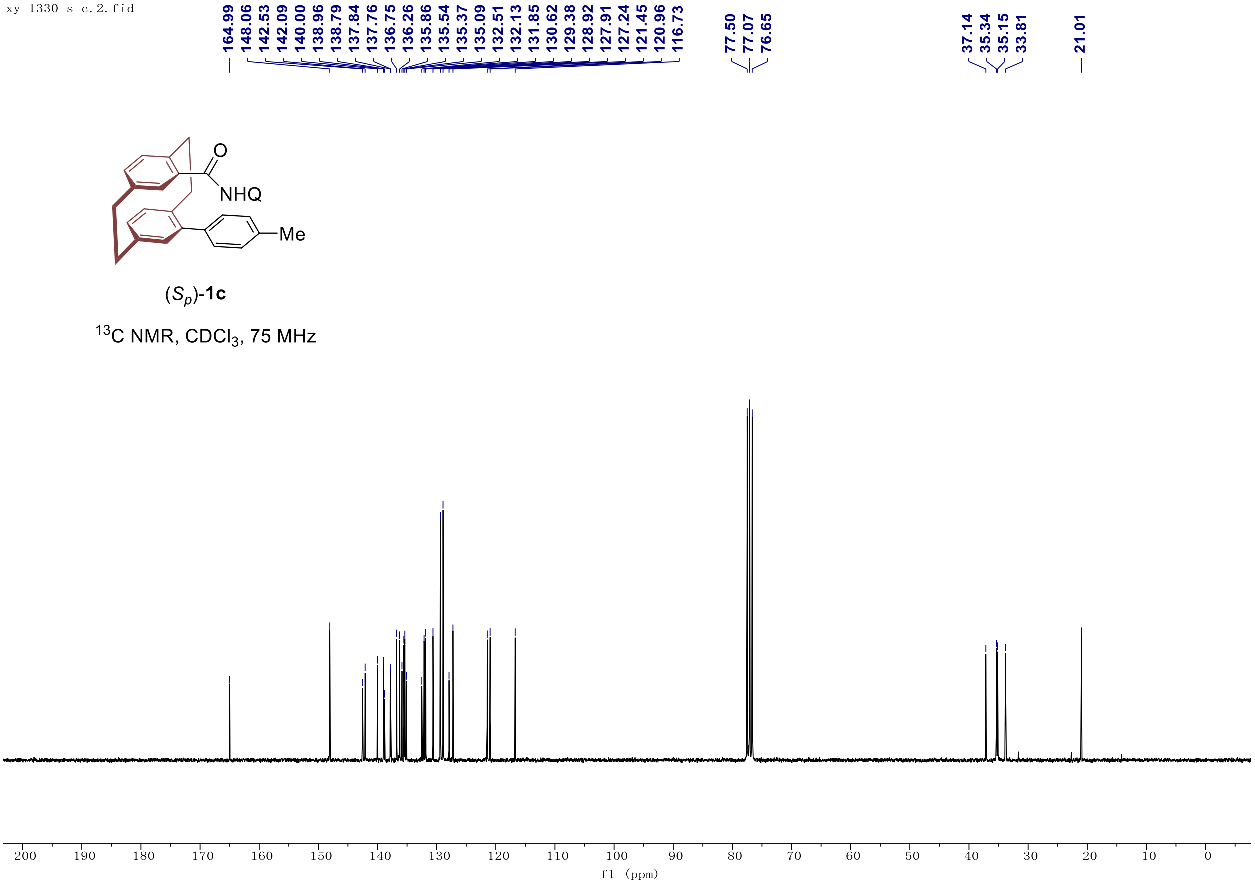
xy-1330-s-c.2.fid
164.99 148.06 142.53 142.09 140.00 138.96 138.79 137.84 137.76 136.75 136.26 135.86 135.54 135.37 135.09 132.51 132.13 131.85 130.62 129.38 128.92 127.91 127.24 121.45 120.96 116.73
77.50 77.07 76.65
37.14 35.34 35.15 33.81
21.01
O
NHQ
Me
(S_p)-**1c**
^{13}C NMR, $CDCl_3$, 75 MHz
f1 (ppm)

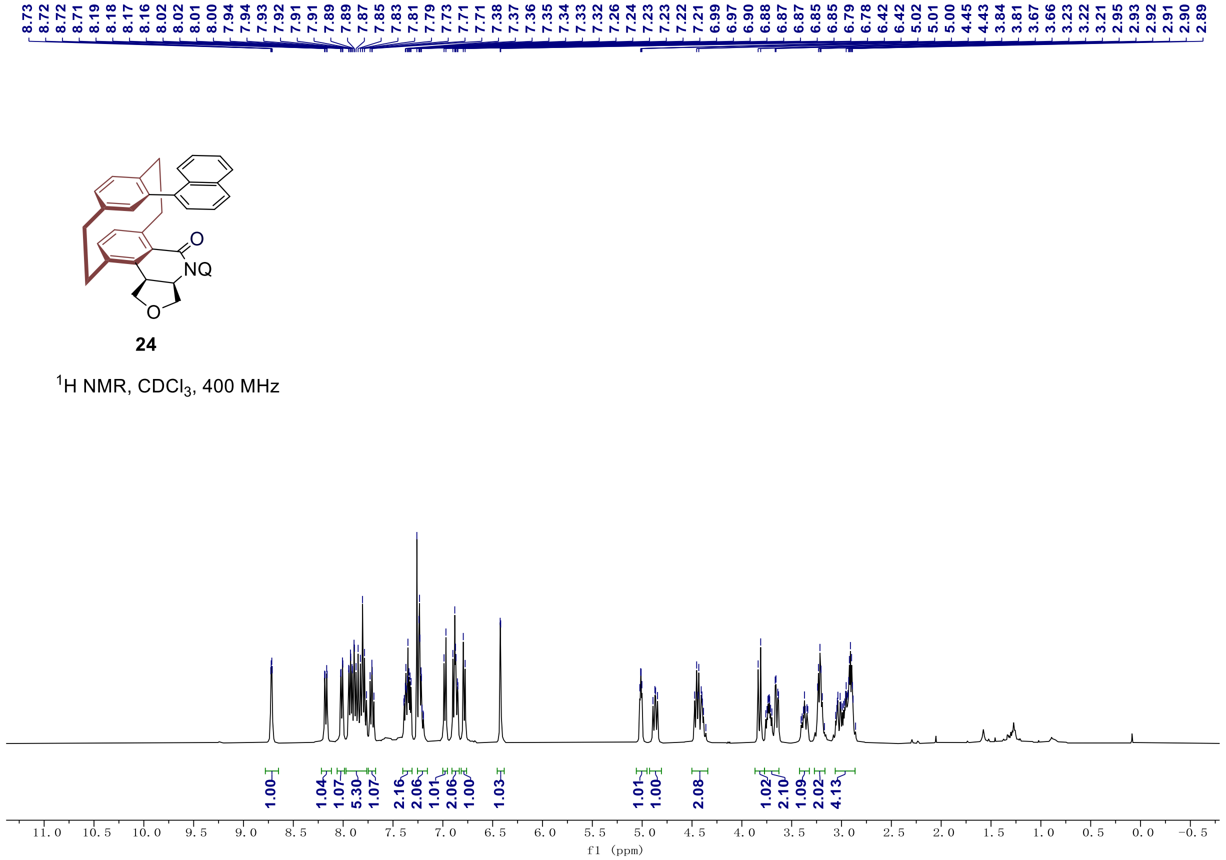

24

^{1}H NMR, $CDCl_3$, 400 MHz

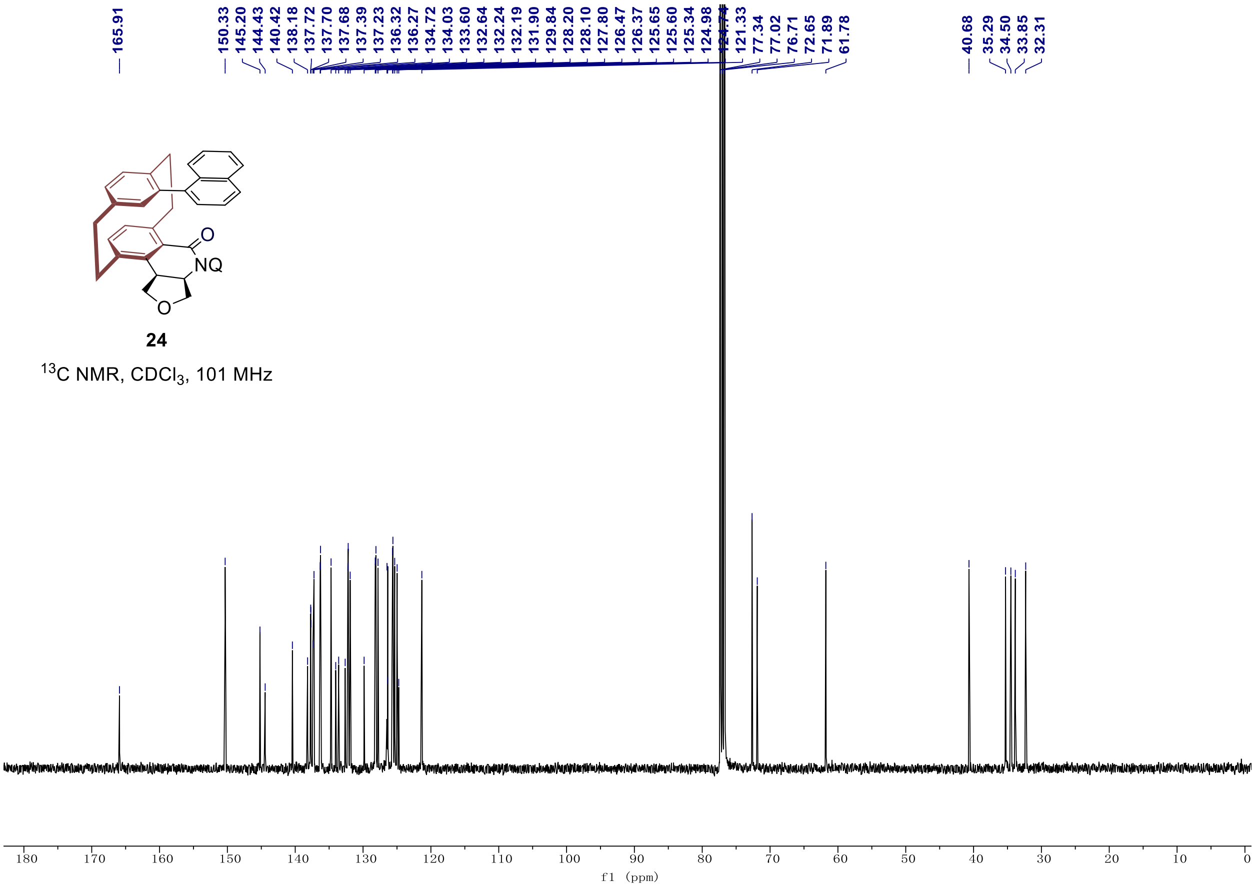

24

^{13}C NMR, $CDCl_3$, 101 MHz

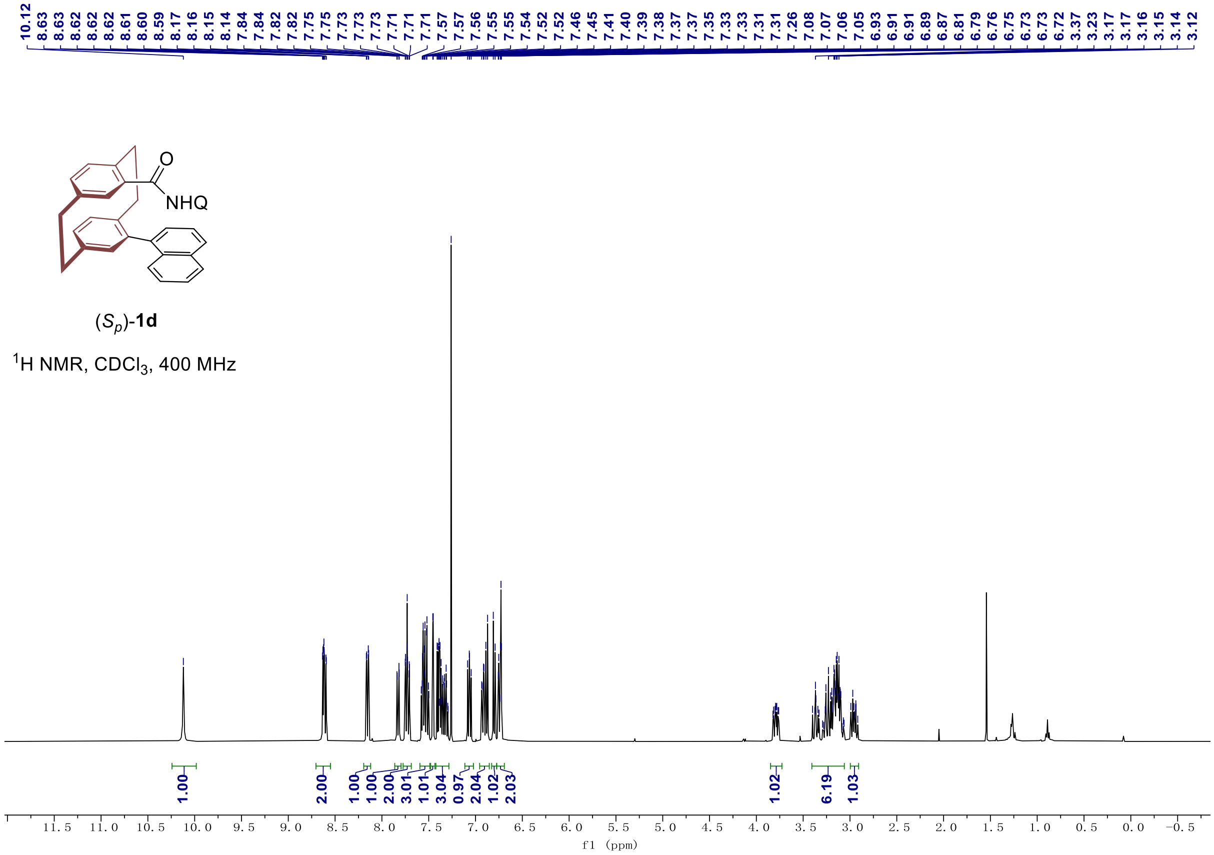

(S_p)-**1d**

^{1}H NMR, $CDCl_3$, 400 MHz

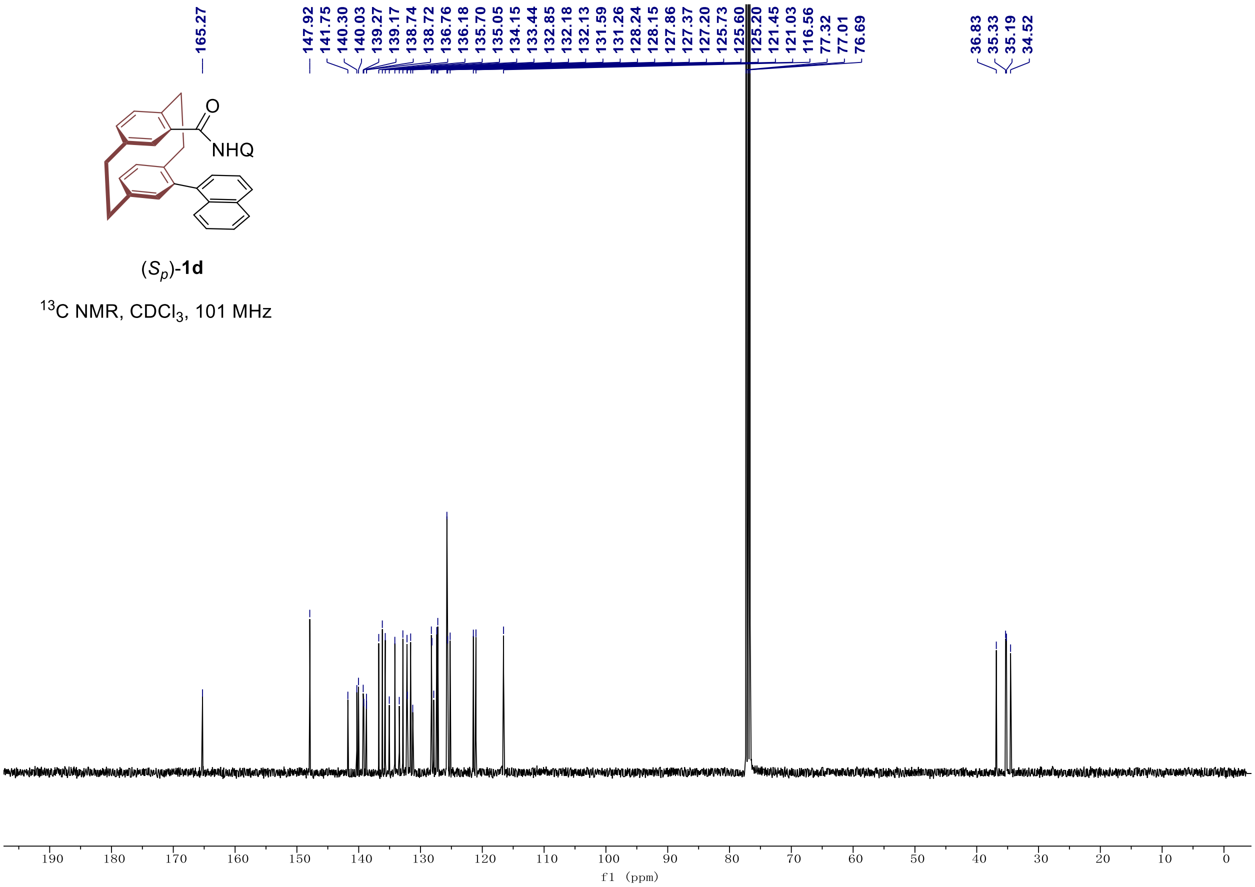

(S_p)-**1d**

^{13}C NMR, $CDCl_3$, 101 MHz

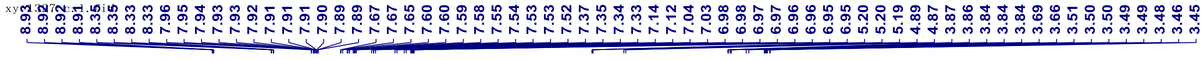

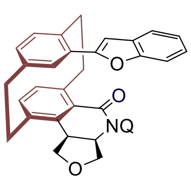

25

^{1}H NMR, $CDCl_3$, 400 MHz

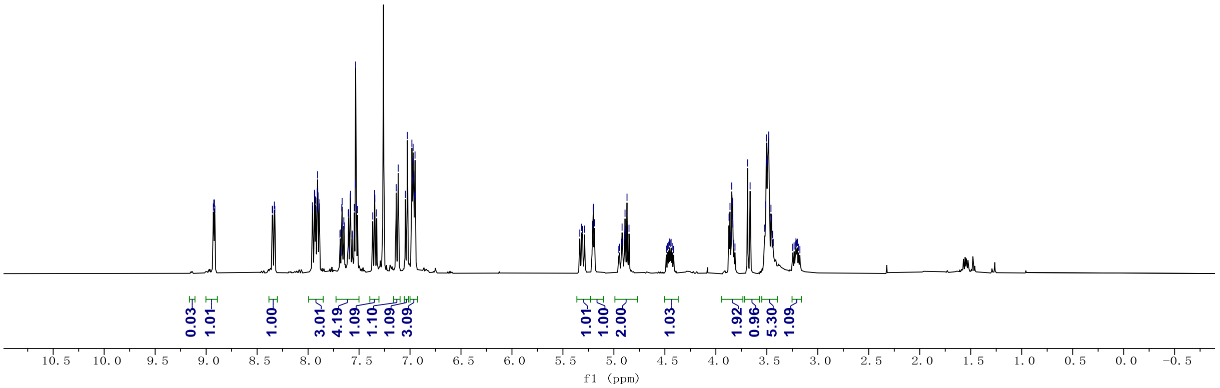

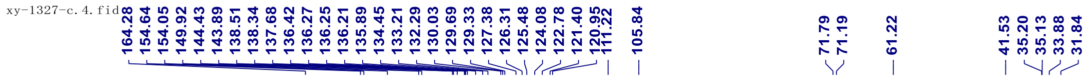

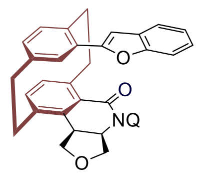

25

^{13}C NMR, $CDCl_3$, 101 MHz

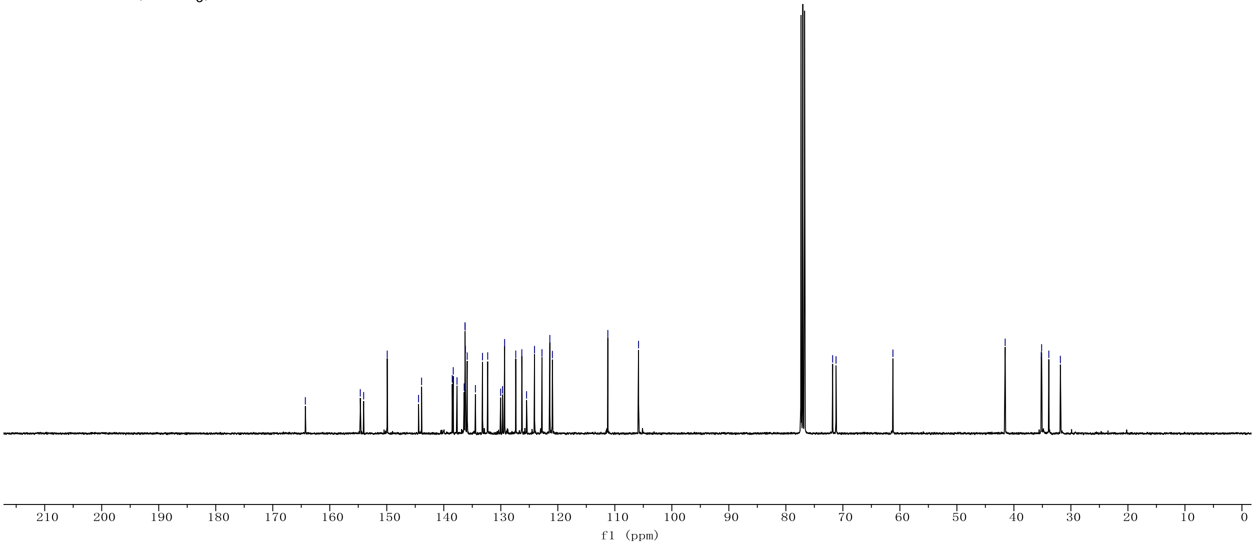

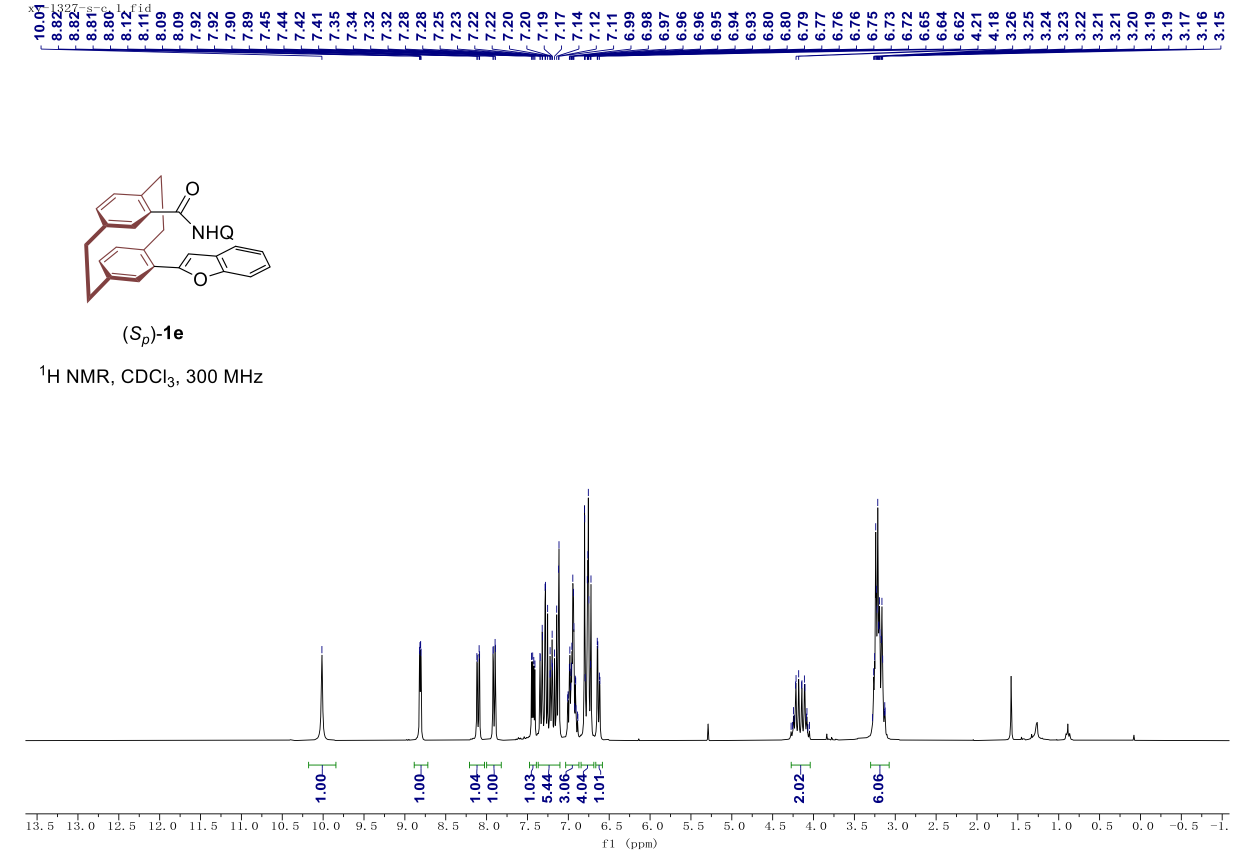

(S_p)-**1e**

^{1}H NMR, $CDCl_3$, 300 MHz

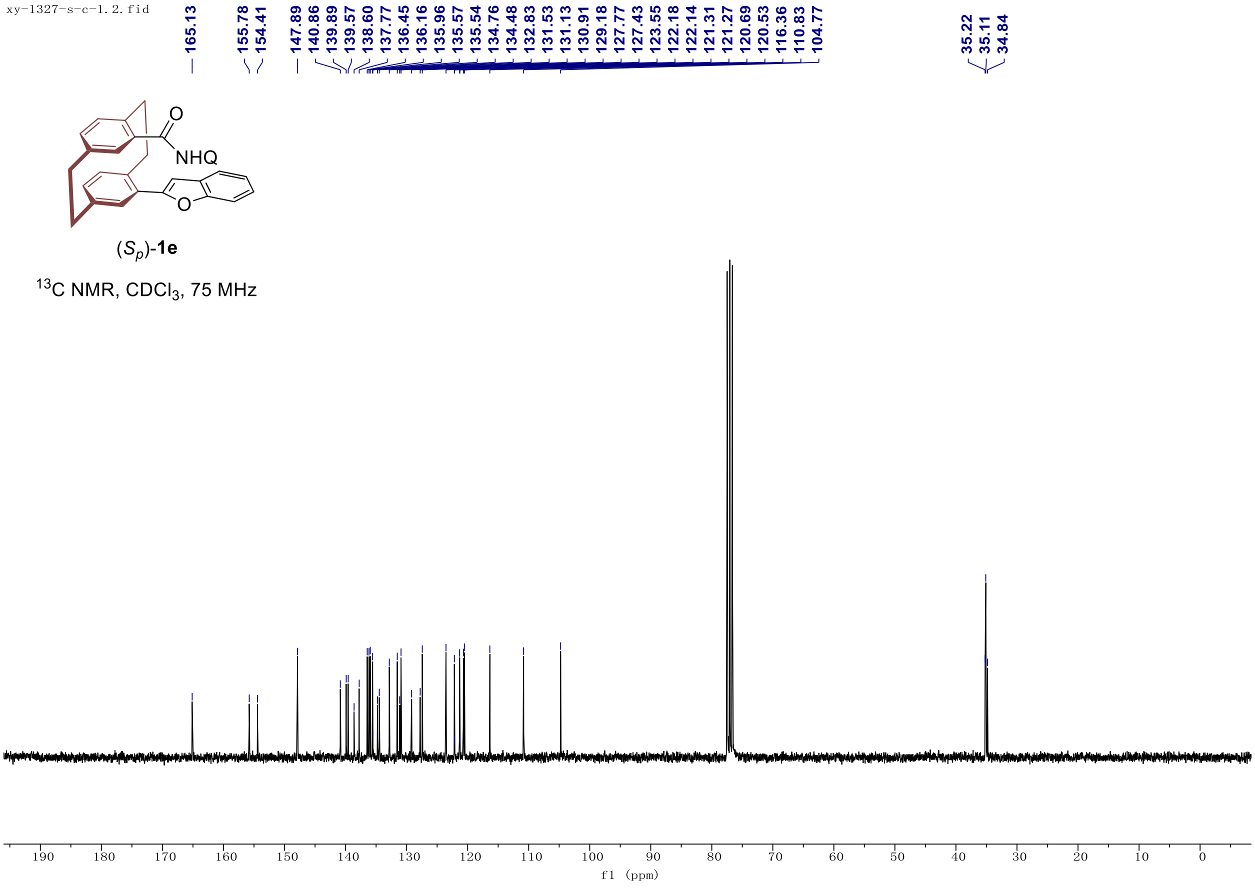

(S_p)-**1e**

^{13}C NMR, $CDCl_3$, 75 MHz

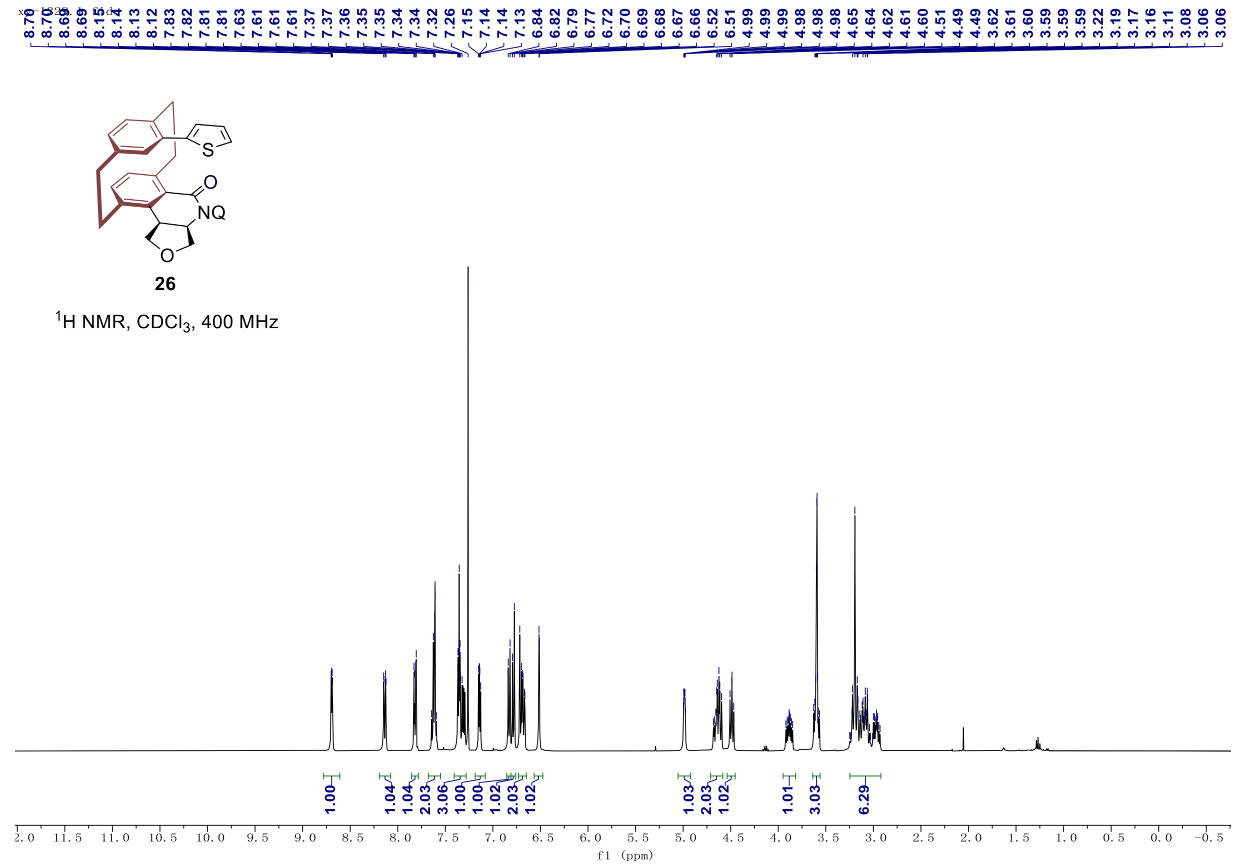
8.70 8.70 8.69 8.69 8.15 8.14 8.13 8.12 7.83 7.82 7.81 7.81 7.63 7.61 7.61 7.61 7.37 7.37 7.36 7.35 7.35 7.34 7.34 7.32 7.26 7.15 7.14 7.14 7.13 6.84 6.82 6.79 6.77 6.72 6.70 6.69 6.68 6.67 6.66 6.52 6.51 4.99 4.99 4.99 4.98 4.98 4.98 4.65 4.64 4.62 4.61 4.60 4.51 4.49 4.49 3.62 3.61 3.60 3.59 3.59 3.59 3.22 3.19 3.17 3.16 3.11 3.08 3.06 3.06
O
NQ
S
O
26
^{1}H NMR, $CDCl_3$, 400 MHz
1.00 1.04 1.04 2.03 3.06 1.00 1.00 1.02 2.03 1.02 1.03 2.03 1.02 1.01 3.03 6.29
f1 (ppm)

xy-1329.4.fid

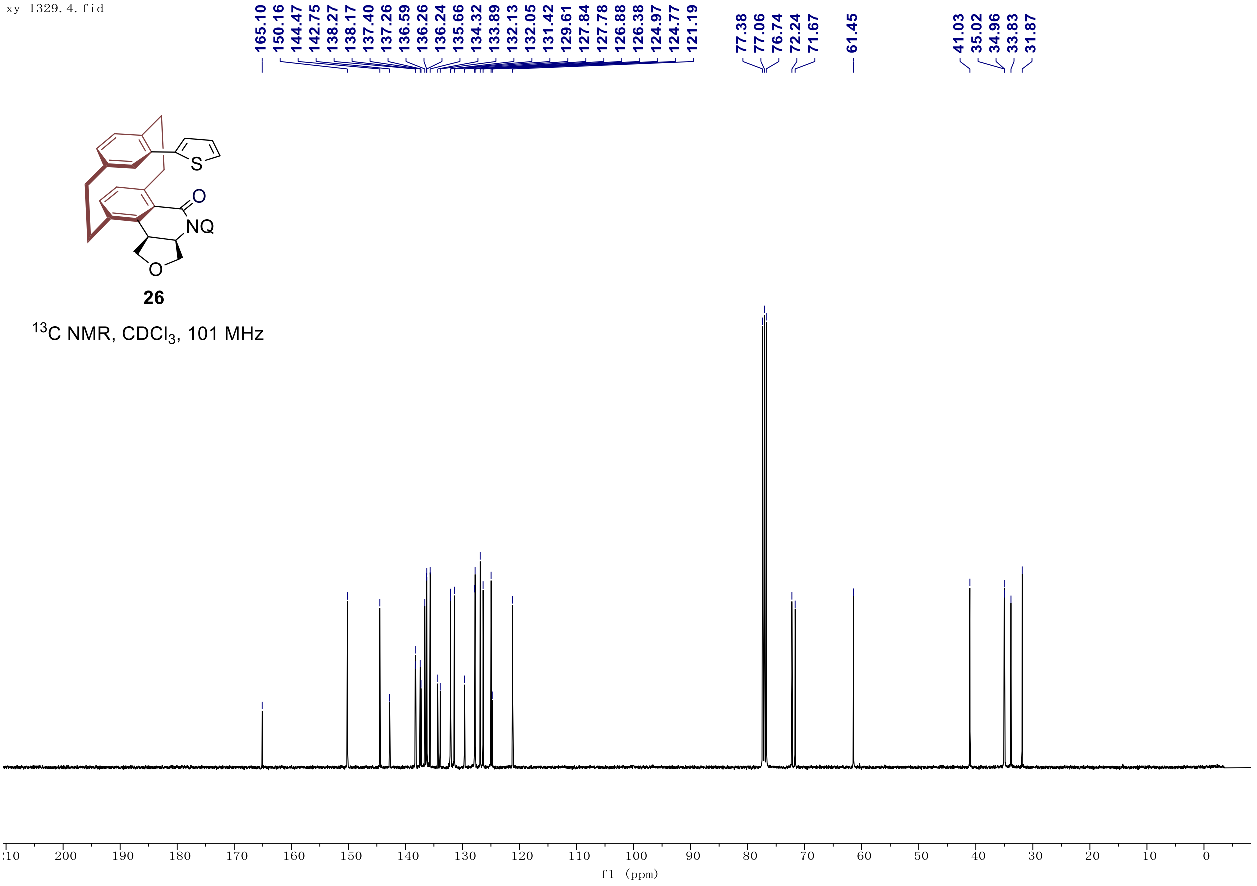
165.10 150.16 144.47 142.75 138.27 138.17 137.40 137.26 136.59 136.26 136.24 135.66 134.32 133.89 132.13 132.05 131.42 129.61 127.84 127.78 126.88 126.38 124.97 124.77 121.19 77.38 77.06 76.74 72.24 71.67 61.45 41.03 35.02 34.96 33.83 31.87
O
NQ
S
O
26
^{13}C NMR, $CDCl_3$, 101 MHz
f1 (ppm)

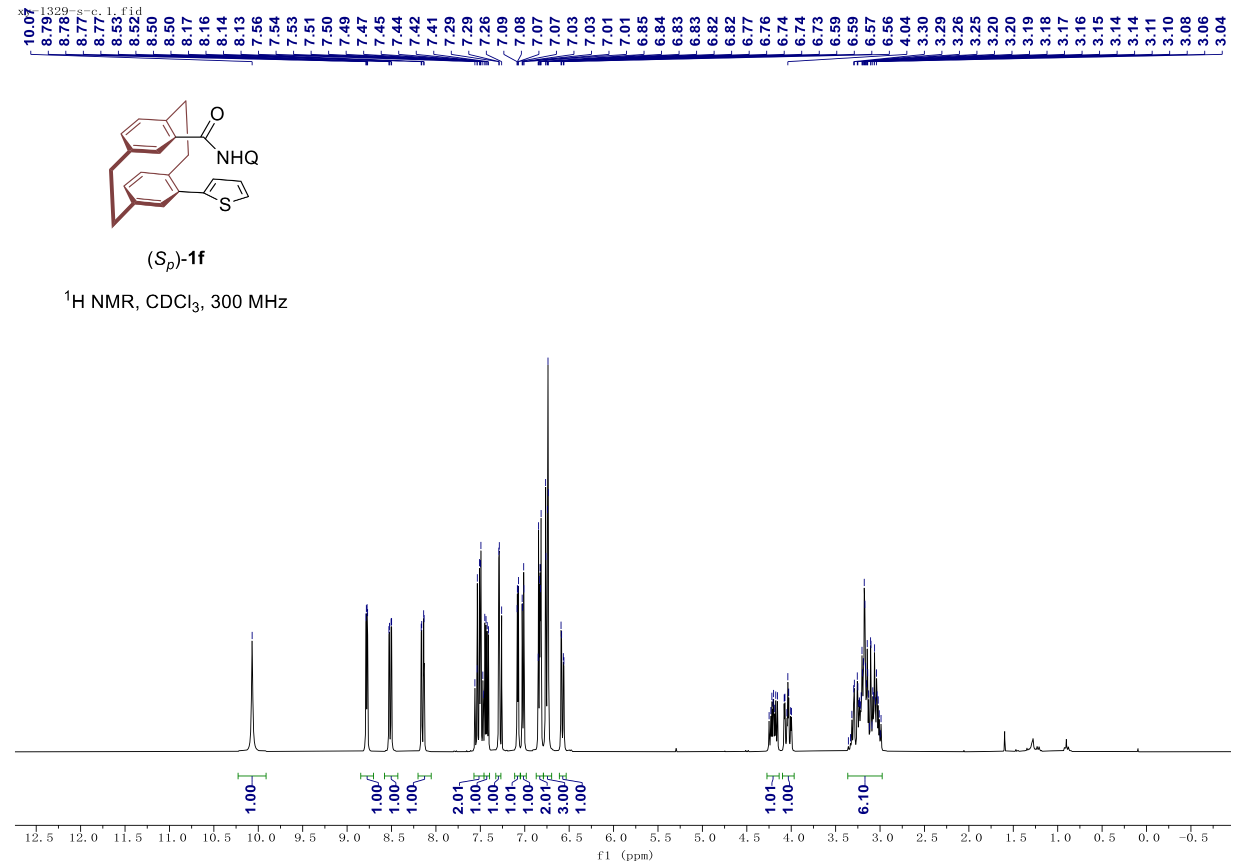
xy-1329-s-c.1.fid
10.07 8.79 8.78 8.77 8.77 8.53 8.52 8.50 8.50 8.17 8.16 8.14 8.13 7.56 7.54 7.53 7.51 7.50 7.49 7.47 7.45 7.44 7.42 7.41 7.29 7.29 7.26 7.09 7.08 7.07 7.07 7.03 7.01 7.01 6.85 6.84 6.83 6.82 6.82 6.77 6.76 6.74 6.74 6.73 6.59 6.57 6.56 4.04 3.30 3.29 3.26 3.25 3.20 3.19 3.18 3.17 3.16 3.15 3.14 3.14 3.11 3.10 3.08 3.06 3.04
O
NHQ
S
(S_p)-**1f**
^{1}H NMR, $CDCl_3$, 300 MHz
1.00 1.00 1.00 1.00 2.01 1.00 1.00 1.01 1.00 2.01 3.00 1.00 1.01 1.00 6.10
12.5 12.0 11.5 11.0 10.5 10.0 9.5 9.0 8.5 8.0 7.5 7.0 6.5 6.0 5.5 5.0 4.5 4.0 3.5 3.0 2.5 2.0 1.5 1.0 0.5 0.0 -0.5
f1 (ppm)

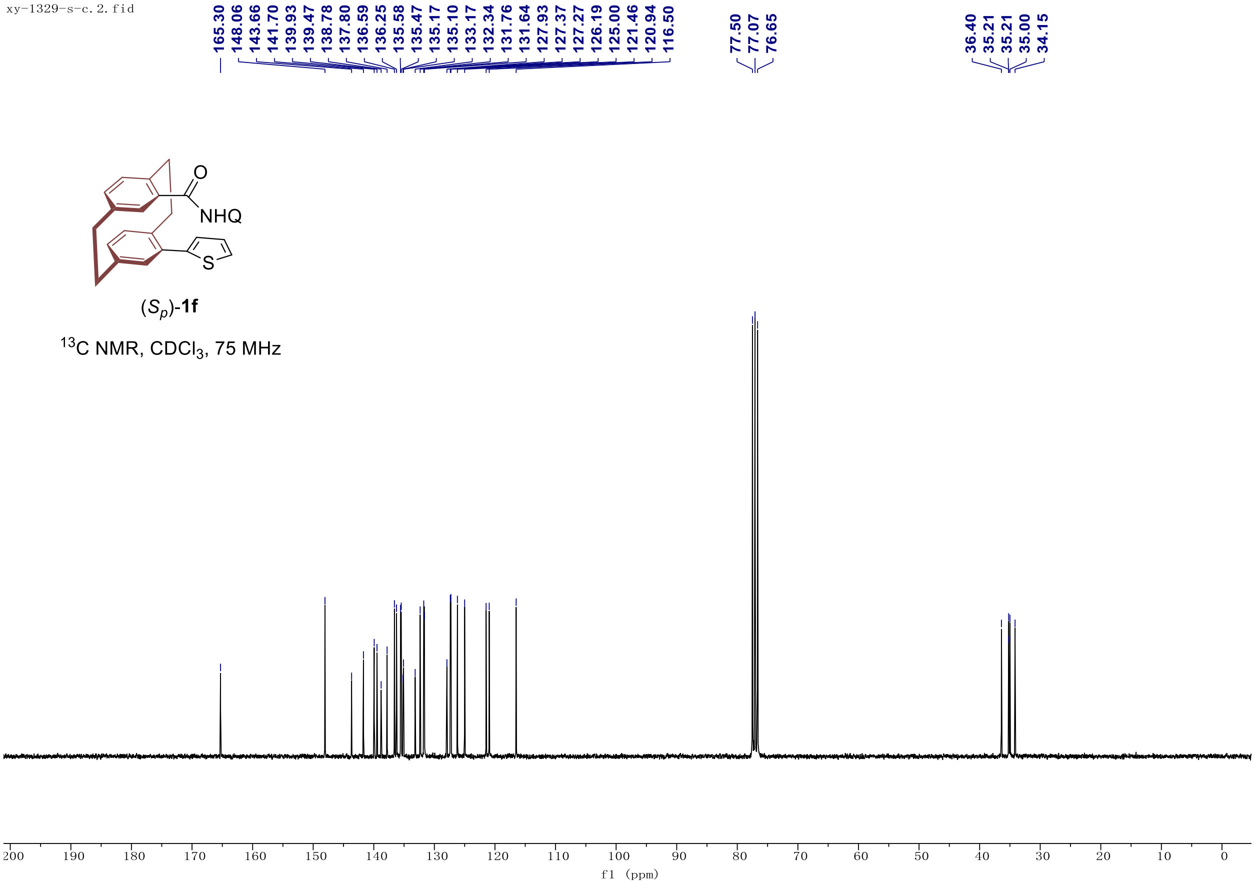
xy-1329-s-c.2.fid
165.30 148.06 143.66 141.70 139.93 139.47 138.78 137.80 136.59 136.25 135.58 135.47 135.17 135.10 133.17 132.34 131.76 131.64 127.93 127.37 127.27 126.19 125.00 121.46 120.94 116.50
77.50 77.07 76.65
36.40 35.21 35.21 35.00 34.15
O
NHQ
S
(S_p)-**1f**
^{13}C NMR, $CDCl_3$, 75 MHz
200 190 180 170 160 150 140 130 120 110 100 90 80 70 60 50 40 30 20 10 0
f1 (ppm)

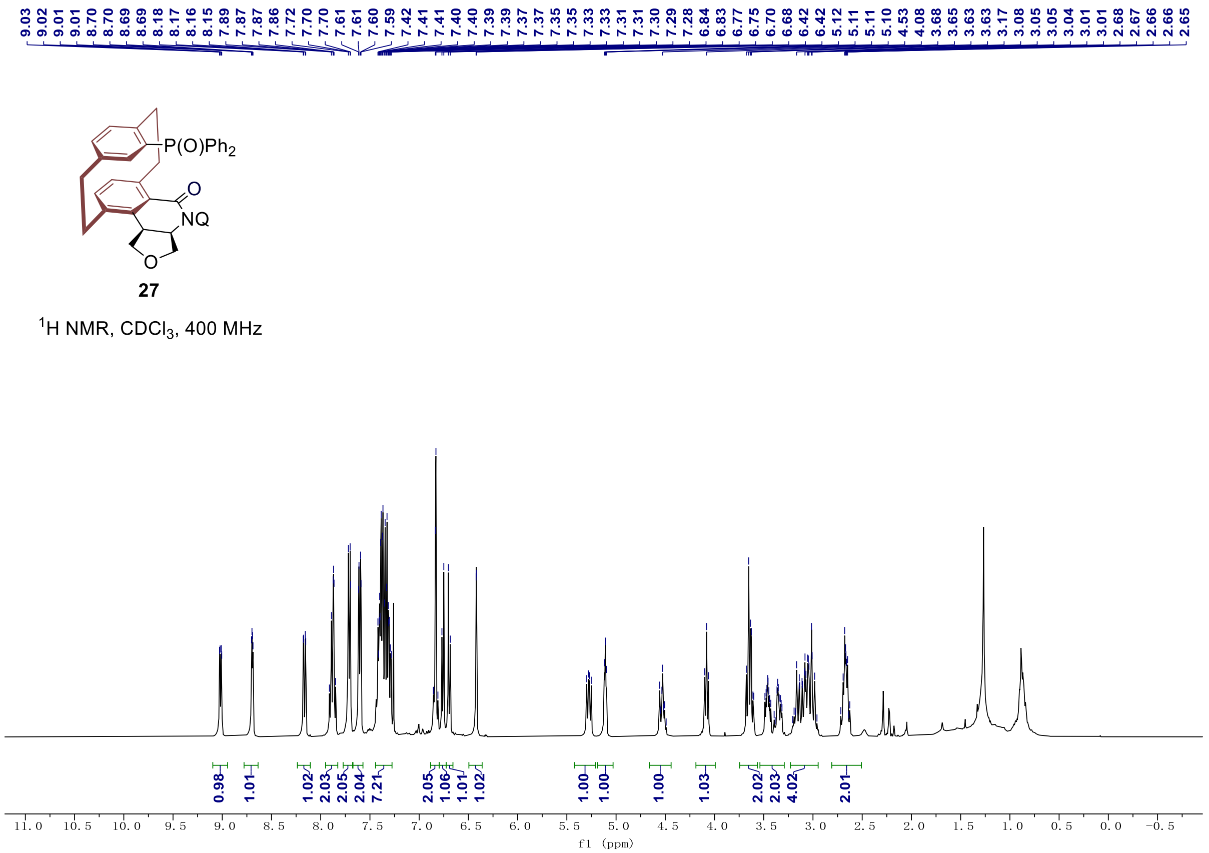

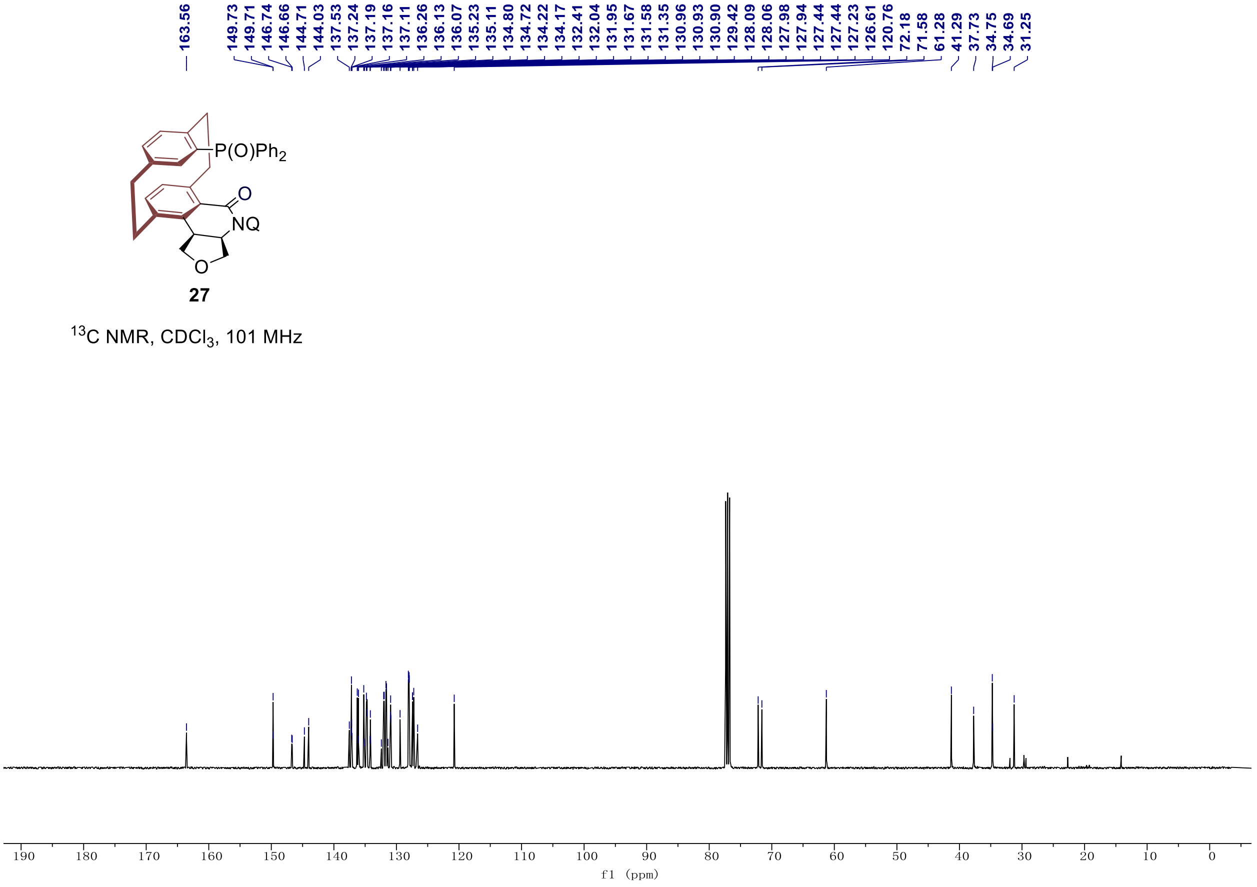

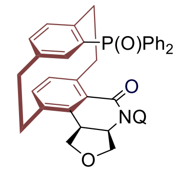

27

^{31}P NMR, $CDCl_3$, 162 MHz

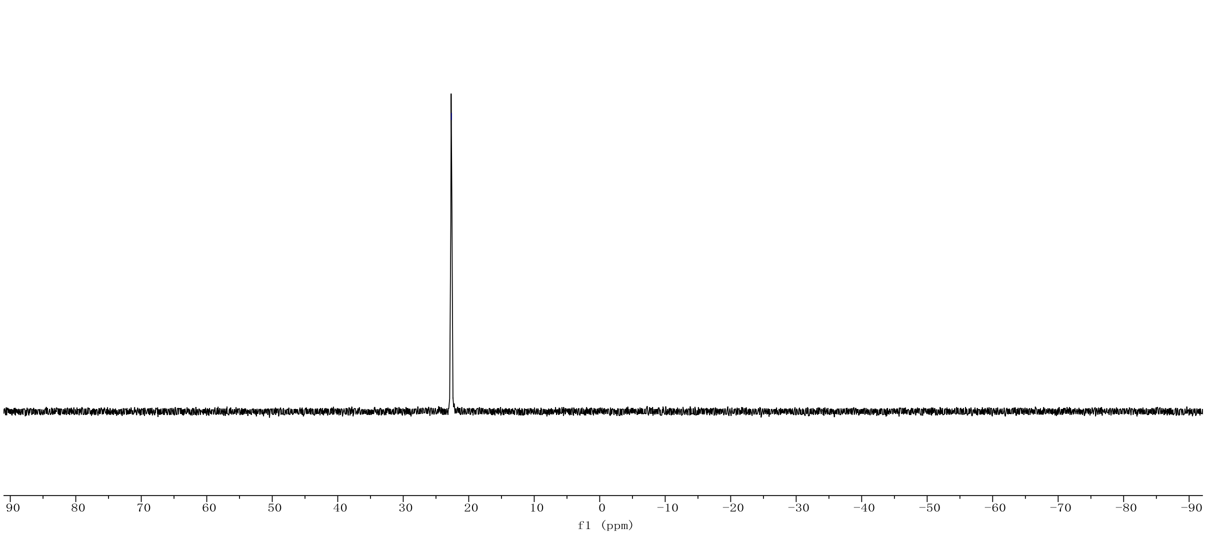

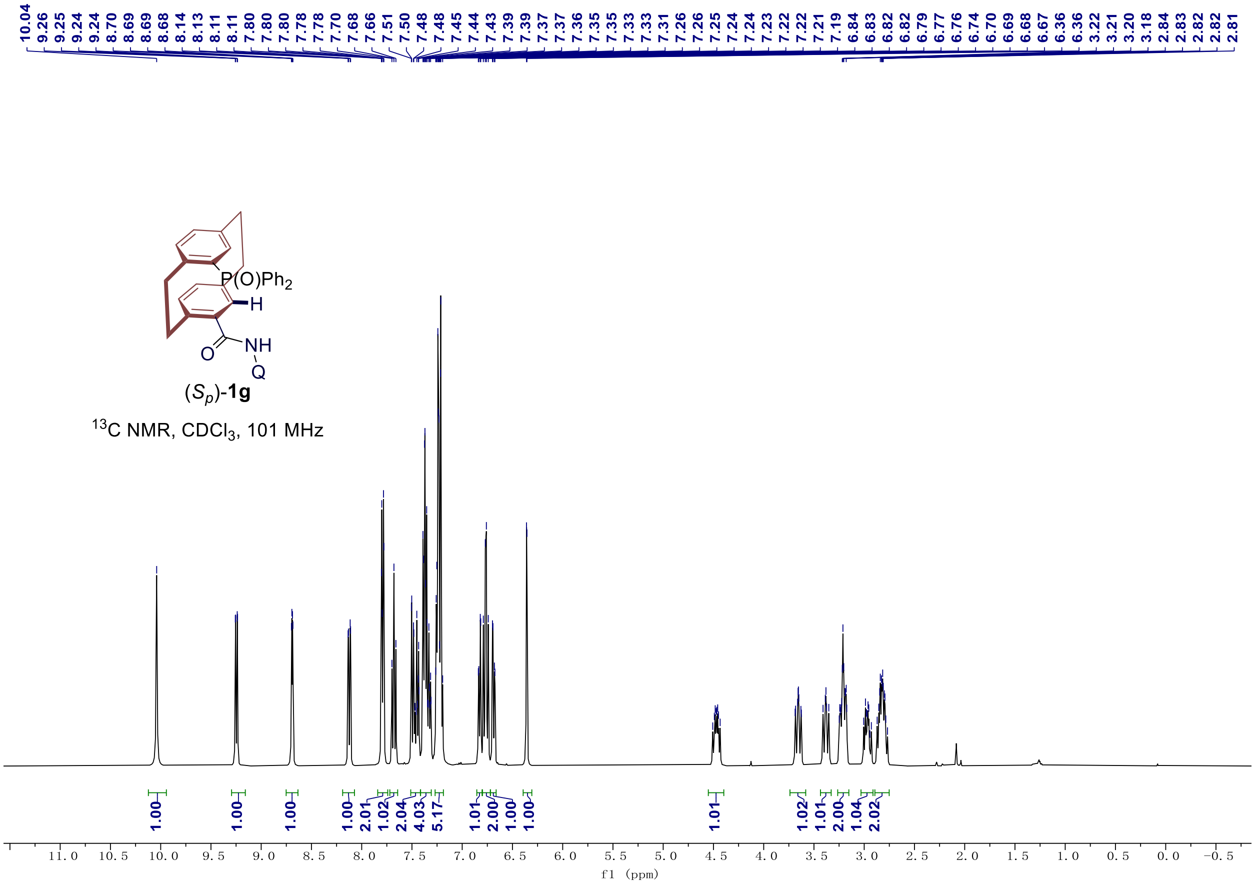
10.04 9.26 9.25 9.24 9.24 8.70 8.69 8.69 8.68 8.14 8.13 8.11 8.11 7.80 7.80 7.80 7.78 7.78 7.70 7.68 7.66 7.51 7.50 7.48 7.48 7.45 7.44 7.43 7.39 7.39 7.37 7.37 7.36 7.35 7.35 7.33 7.33 7.31 7.26 7.26 7.25 7.24 7.24 7.23 7.22 7.22 7.21 7.19 6.84 6.83 6.82 6.82 6.79 6.77 6.76 6.74 6.70 6.69 6.68 6.67 6.36 6.36 3.22 3.21 3.20 3.18 2.84 2.83 2.82 2.82 2.81
P(O)Ph2
H
O
NH
Q
(Sp)-1g
13C NMR, CDCl3, 101 MHz
1.00 1.00 1.00 1.00 2.01 1.02 2.04 4.03 5.17 1.01 2.00 1.00 1.00 1.01 1.02 1.01 2.00 1.04 2.02
11.0 10.5 10.0 9.5 9.0 8.5 8.0 7.5 7.0 6.5 6.0 5.5 5.0 4.5 4.0 3.5 3.0 2.5 2.0 1.5 1.0 0.5 0.0 -0.5
f1 (ppm)

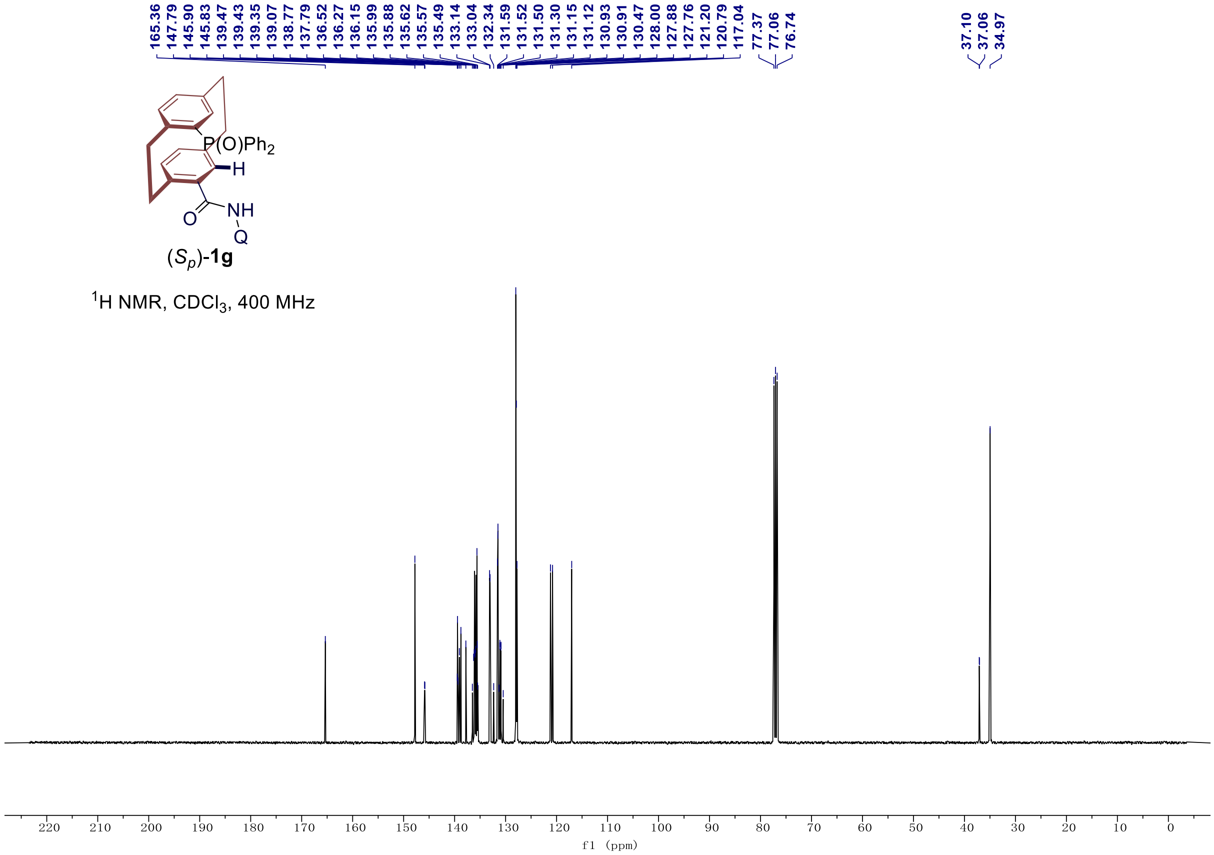
165.36 147.79 145.90 145.83 139.47 139.43 139.35 139.07 138.77 137.79 136.52 136.27 136.15 135.99 135.88 135.62 135.57 135.49 133.14 133.04 132.34 131.59 131.52 131.50 131.30 131.15 131.12 130.93 130.91 130.47 128.00 127.88 127.76 121.20 120.79 117.04 77.37 77.06 76.74 37.10 37.06 34.97
P(O)Ph2
H
O
NH
Q
(Sp)-1g
1H NMR, CDCl3, 400 MHz
220 210 200 190 180 170 160 150 140 130 120 110 100 90 80 70 60 50 40 30 20 10 0
f1 (ppm)

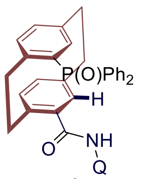

(S_p)-**1g**

^{31}P NMR, $CDCl_3$, 162 MHz

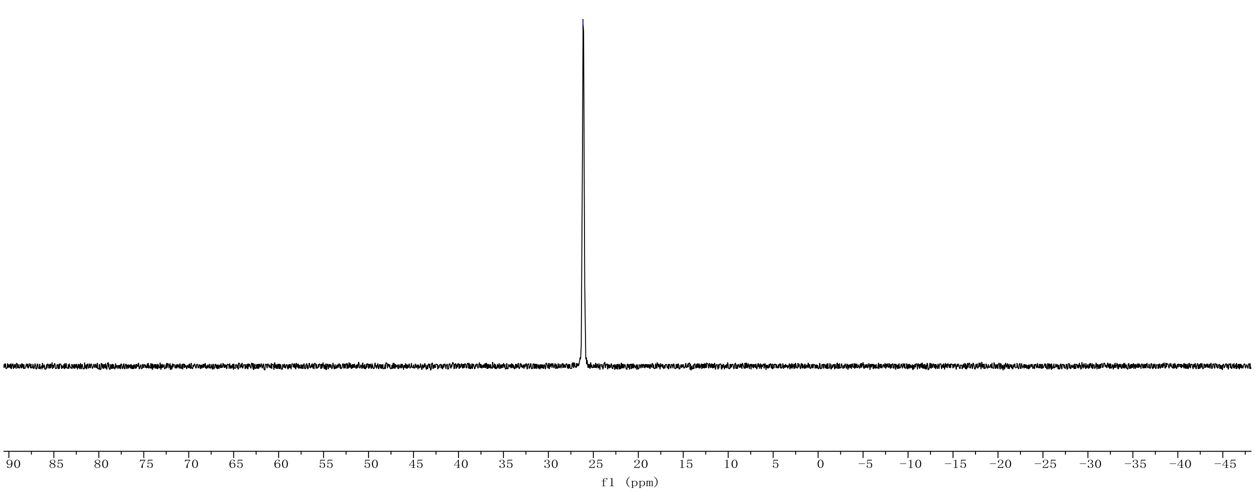

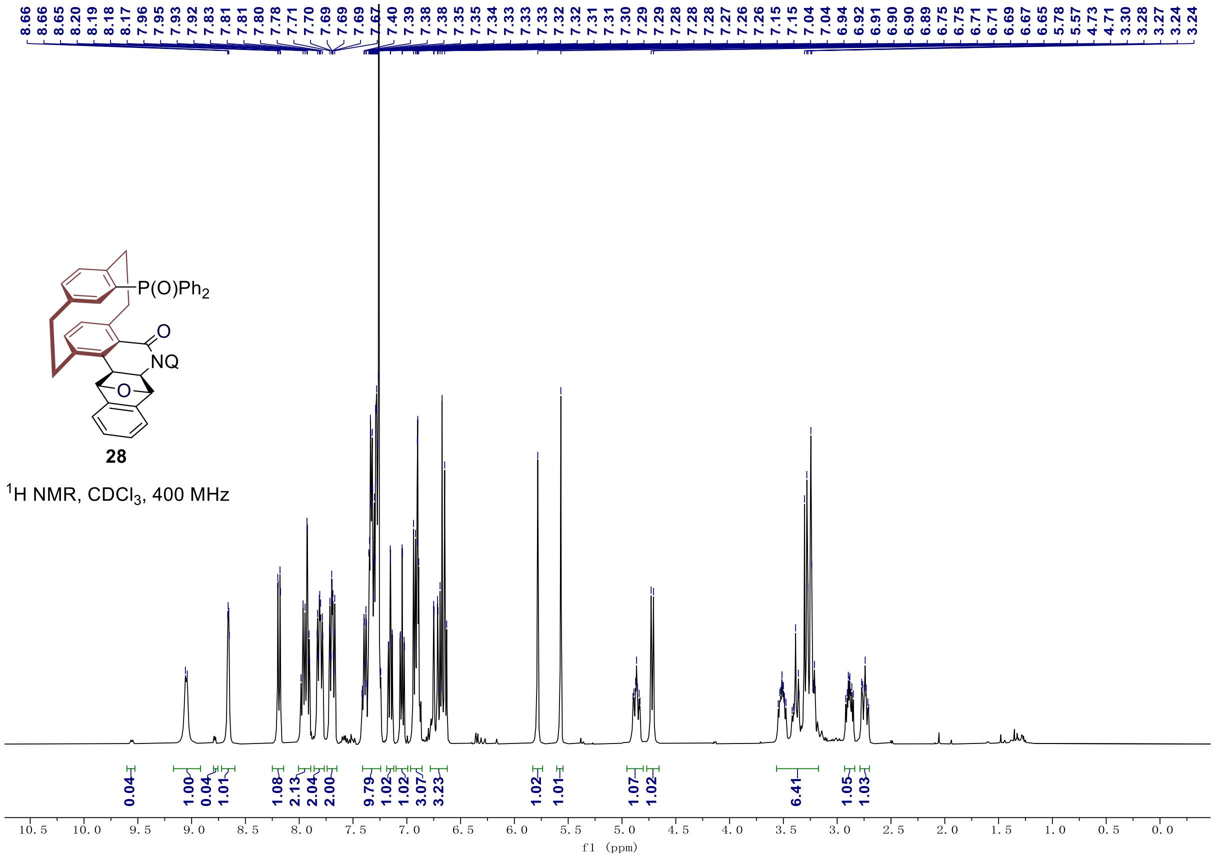
8.66 8.66 8.65 8.20 8.19 8.18 8.17 7.96 7.95 7.93 7.92 7.83 7.81 7.81 7.80 7.78 7.71 7.70 7.69 7.69 7.69 7.67 7.40 7.39 7.38 7.38 7.35 7.35 7.34 7.33 7.33 7.33 7.32 7.32 7.31 7.31 7.30 7.29 7.29 7.28 7.28 7.28 7.27 7.26 7.26 7.15 7.15 7.04 7.04 6.94 6.92 6.91 6.91 6.90 6.90 6.89 6.75 6.75 6.71 6.71 6.69 6.67 6.65 5.78 5.57 4.73 4.71 3.30 3.28 3.27 3.24 3.24
P(O)Ph2
O
NQ
O
28
1H NMR, CDCl3, 400 MHz
0.04 1.00 0.04 1.01 1.08 2.13 2.04 2.00 9.79 1.02 1.02 3.07 3.23 1.02 1.01 1.07 1.02 6.41 1.05 1.03
10.5 10.0 9.5 9.0 8.5 8.0 7.5 7.0 6.5 6.0 5.5 5.0 4.5 4.0 3.5 3.0 2.5 2.0 1.5 1.0 0.5 0.0
f1 (ppm)

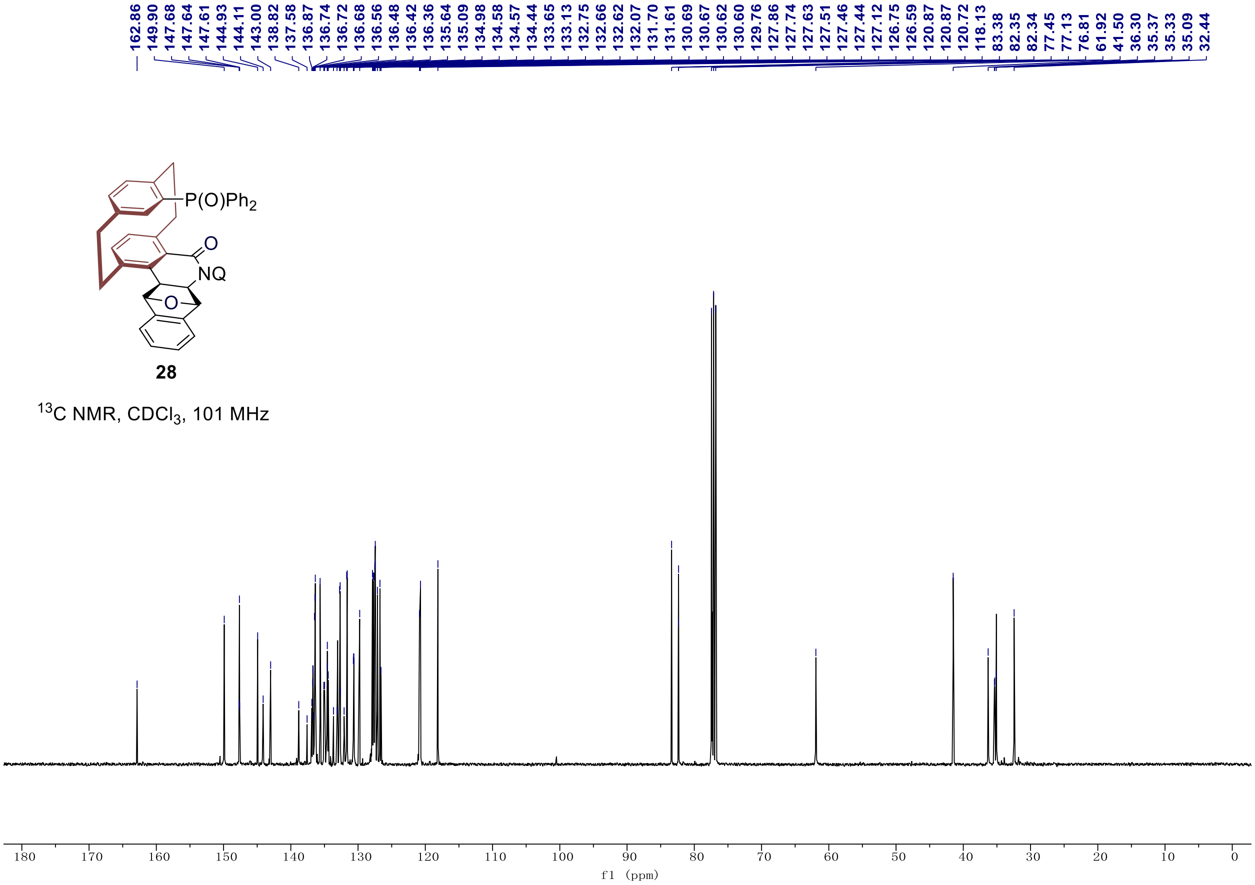
162.86 149.90 147.68 147.64 147.61 144.93 144.11 143.00 138.82 137.58 136.87 136.74 136.72 136.68 136.56 136.48 136.42 136.36 135.64 135.09 134.98 134.58 134.57 134.44 133.65 133.13 132.75 132.66 132.62 132.07 131.70 131.61 130.69 130.67 130.62 130.60 129.76 127.86 127.74 127.63 127.51 127.46 127.44 127.12 126.75 126.59 120.87 120.87 120.72 118.13 83.38 82.35 82.34 77.45 77.13 76.81 61.92 41.50 36.30 35.37 35.33 35.09 32.44
P(O)Ph2
O
NQ
O
28
13C NMR, CDCl3, 101 MHz
180 170 160 150 140 130 120 110 100 90 80 70 60 50 40 30 20 10 0
f1 (ppm)

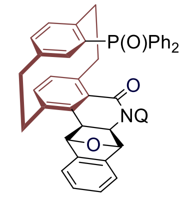

28

^{31}P NMR, $CDCl_3$, 162 MHz

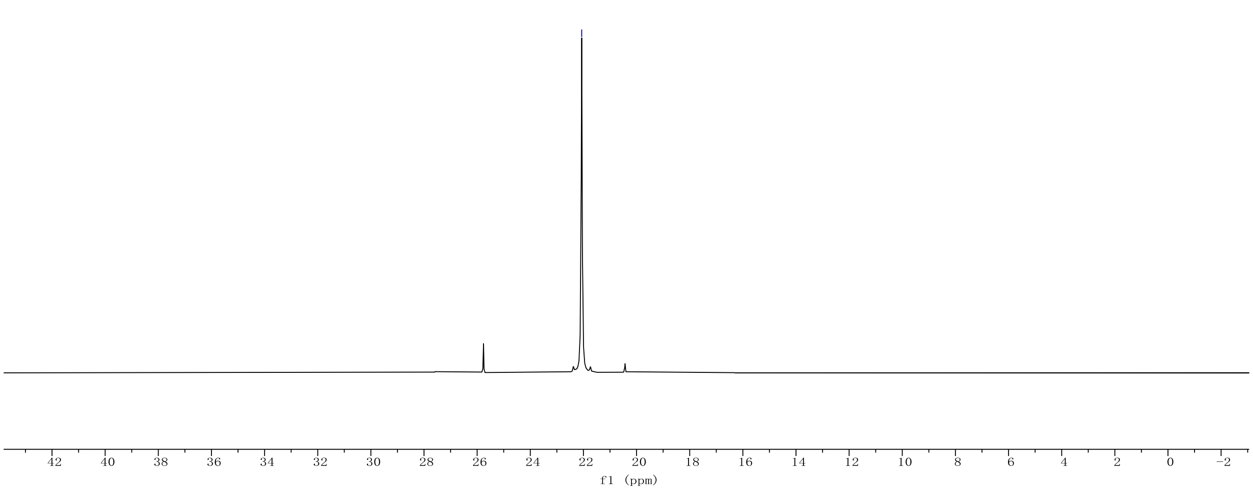

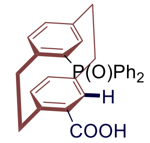

(S_p)-**29**

^{1}H NMR, $CDCl_3$, 400 MHz

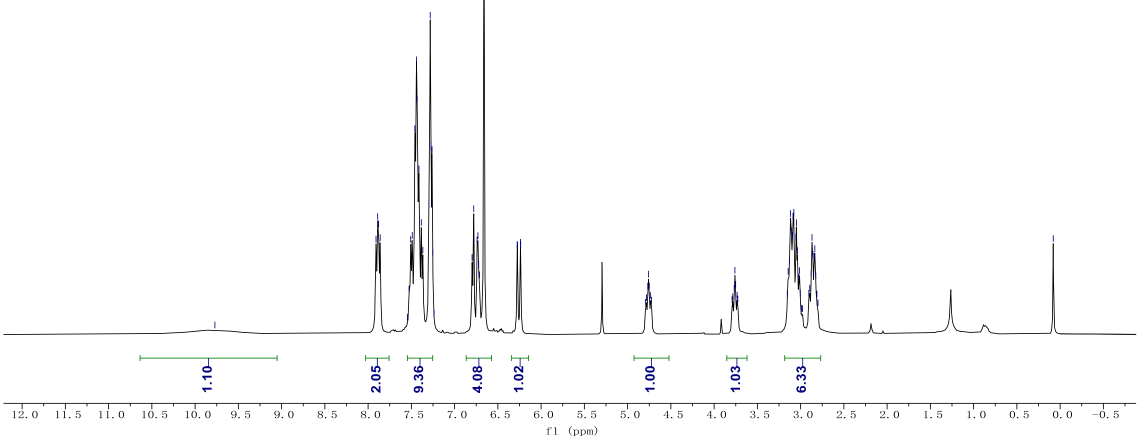

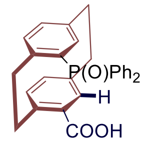

(S_p)-**29**

^{13}C NMR, $CDCl_3$, 101 MHz

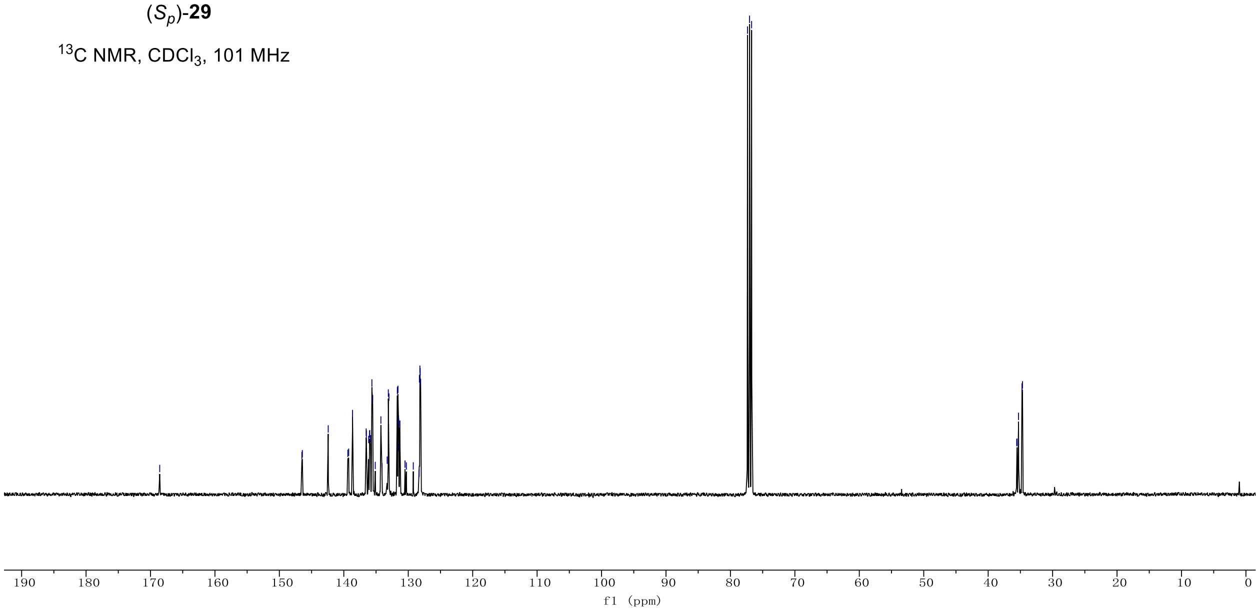

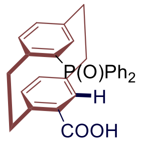

(S_p)-**29**

^{31}P NMR, $CDCl_3$, 162 MHz

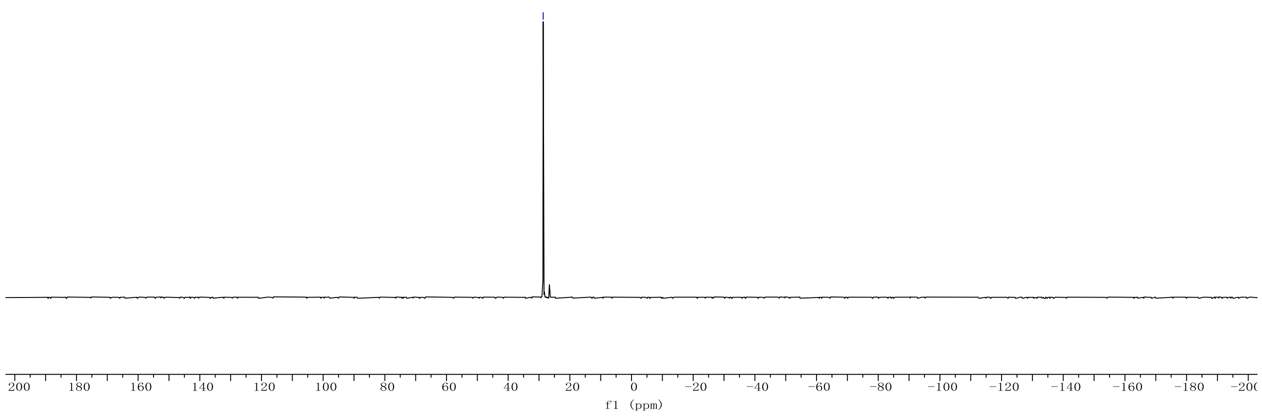

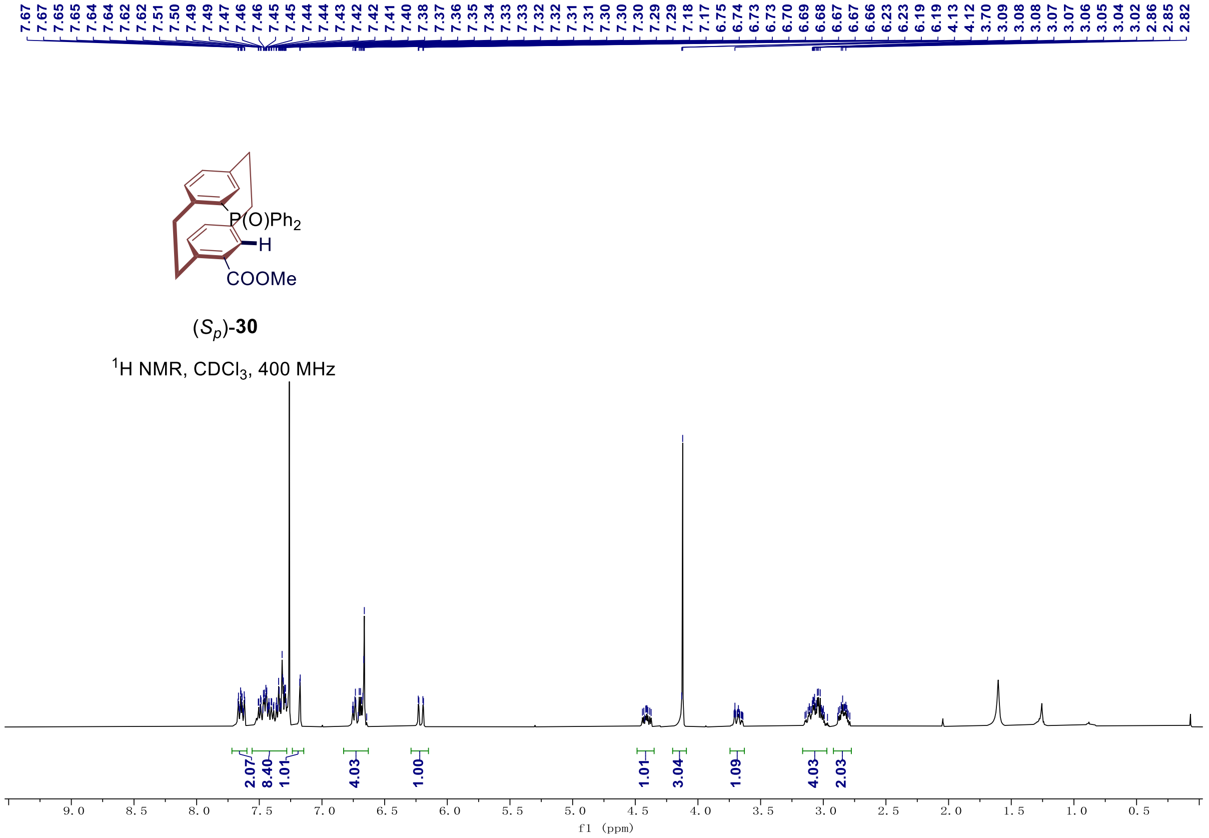
7.67 7.67 7.65 7.64 7.64 7.62 7.62 7.51 7.50 7.49 7.49 7.47 7.46 7.46 7.45 7.45 7.44 7.44 7.43 7.42 7.42 7.41 7.40 7.38 7.37 7.36 7.35 7.34 7.33 7.33 7.32 7.32 7.31 7.31 7.30 7.30 7.30 7.29 7.29 7.18 7.17 6.75 6.74 6.73 6.73 6.70 6.69 6.68 6.67 6.67 6.66 6.23 6.23 6.19 6.19 4.13 4.12 3.70 3.09 3.08 3.08 3.07 3.07 3.06 3.05 3.04 3.02 2.86 2.85 2.82
P(O)Ph2
H
COOMe
(Sp)-30
1H NMR, CDCl3, 400 MHz
2.07 8.40 1.01 4.03 1.00 1.01 3.04 1.09 4.03 2.03
9.0 8.5 8.0 7.5 7.0 6.5 6.0 5.5 5.0 4.5 4.0 3.5 3.0 2.5 2.0 1.5 1.0 0.5
f1 (ppm)

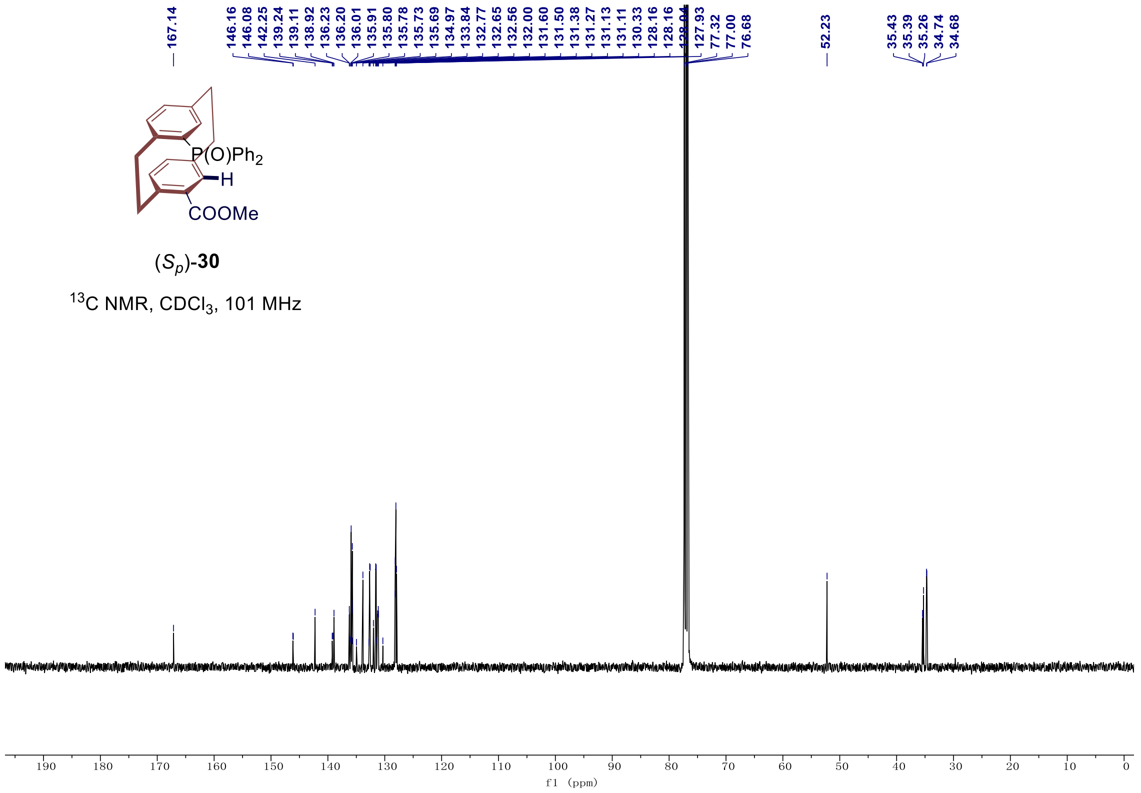
167.14
146.16 146.08 142.25 139.24 139.11 138.92 136.23 136.20 136.01 135.91 135.80 135.78 135.73 135.69 134.97 133.84 132.77 132.65 132.56 132.00 131.60 131.50 131.38 131.27 131.13 131.11 130.33 128.16 127.93 77.32 77.00 76.68
52.23
35.43 35.39 35.26 34.74 34.68
P(O)Ph2
H
COOMe
(Sp)-30
13C NMR, CDCl3, 101 MHz
190 180 170 160 150 140 130 120 110 100 90 80 70 60 50 40 30 20 10 0
f1 (ppm)

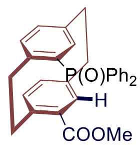

(S_p)-**30**

^{31}P NMR, $CDCl_3$, 162 MHz

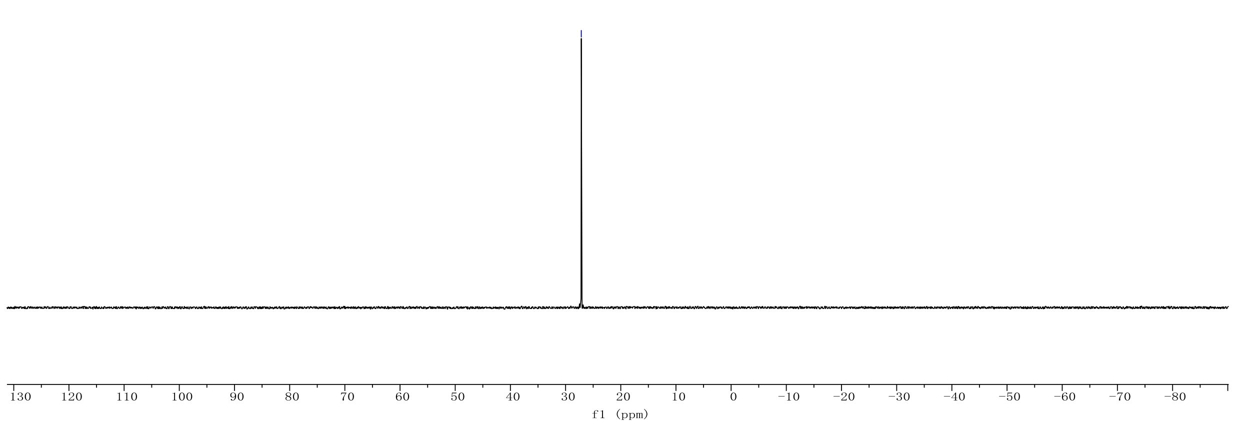

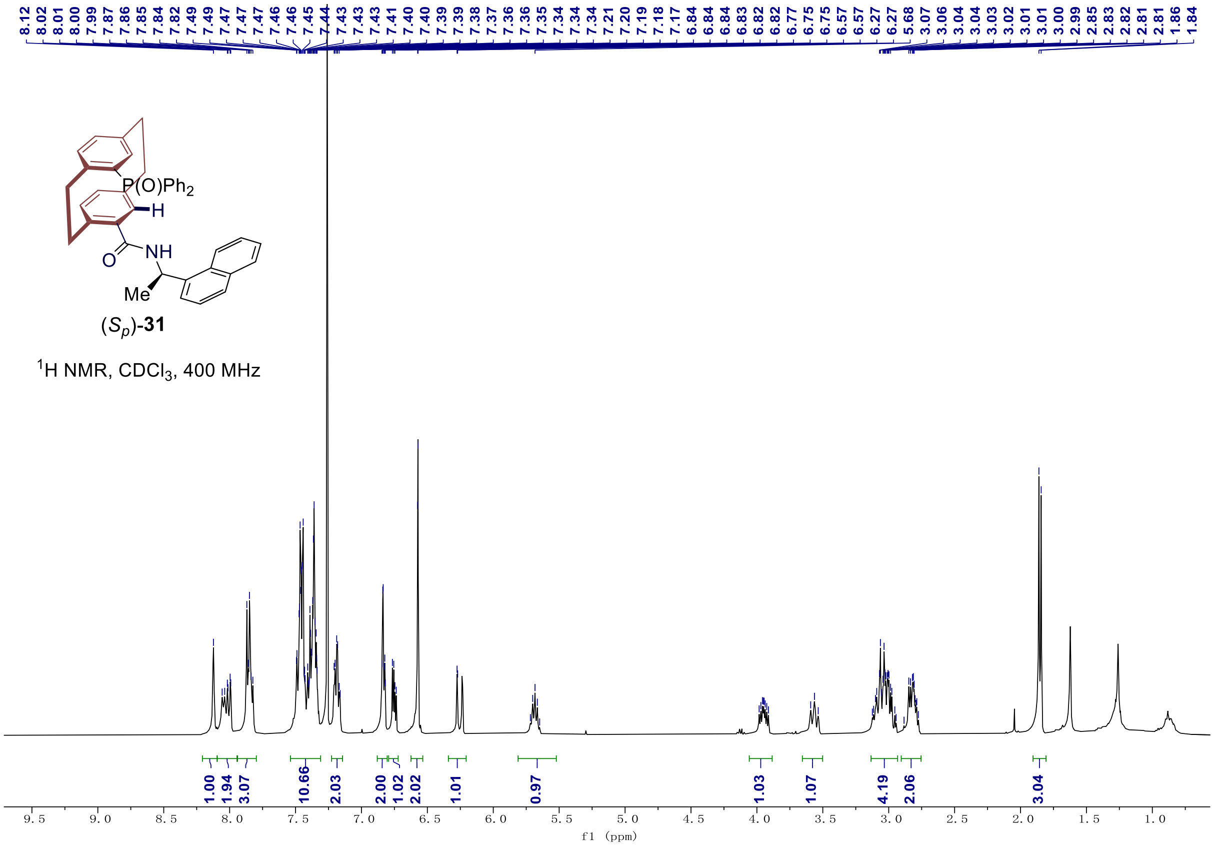

(S_p)-**31**

^{1}H NMR, $CDCl_3$, 400 MHz

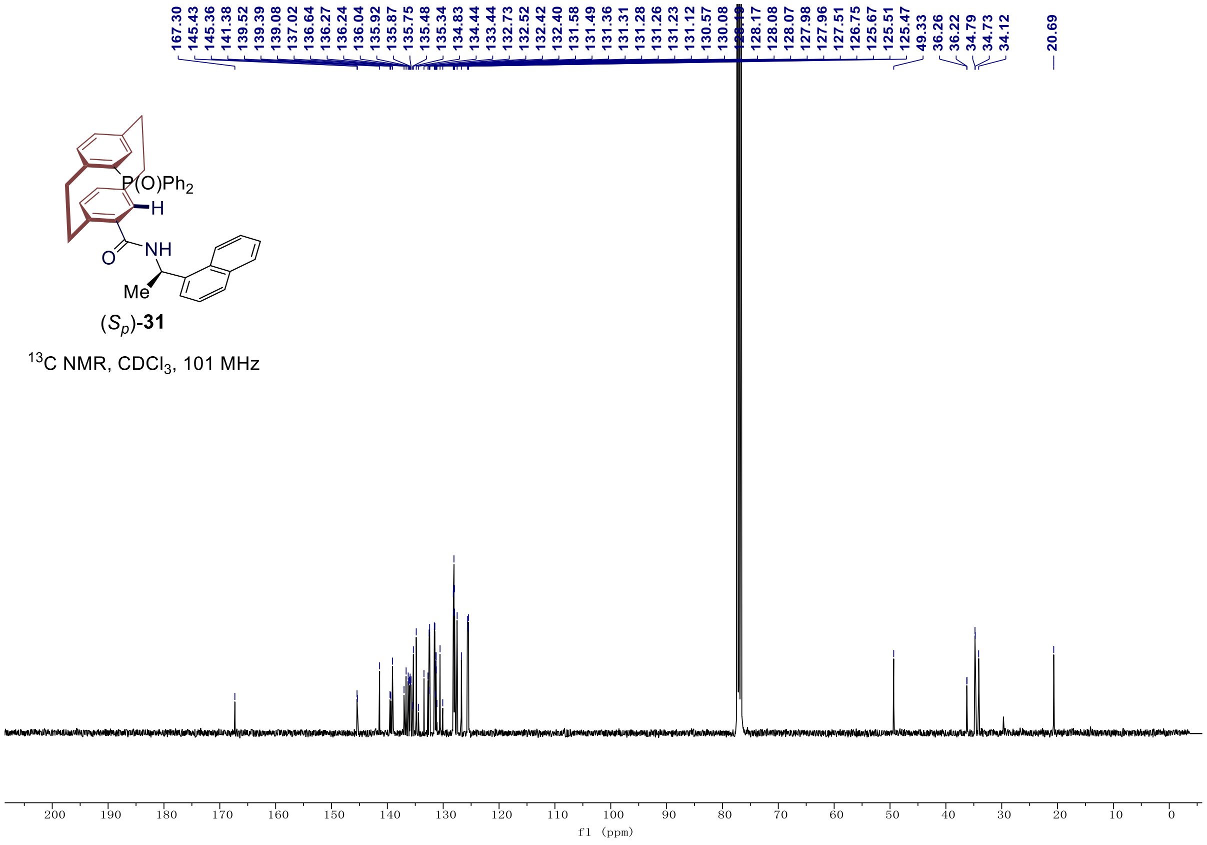

(S_p)-**31**

^{13}C NMR, $CDCl_3$, 101 MHz

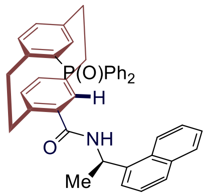

(S_p)-**31**

^{31}P NMR, $CDCl_3$, 162 MHz

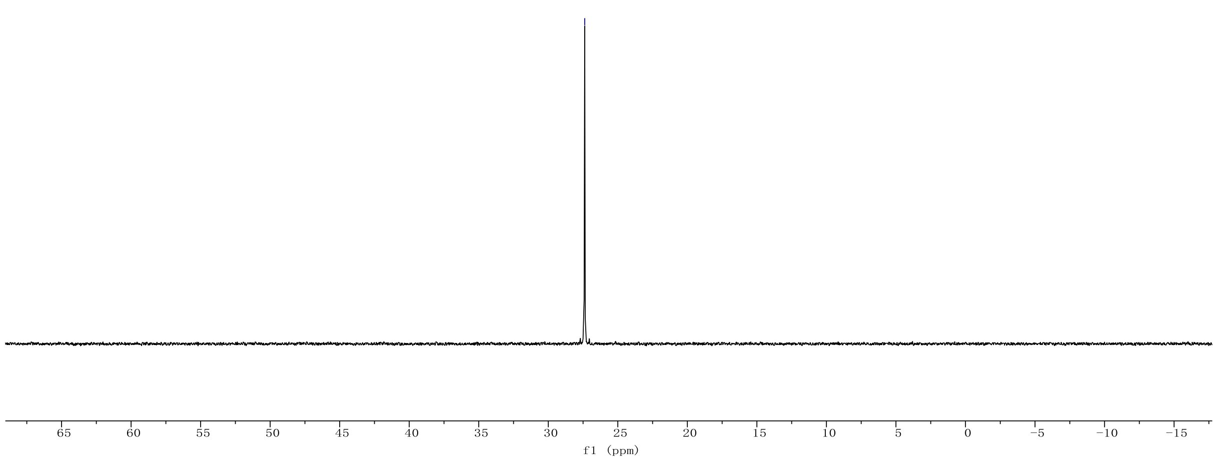

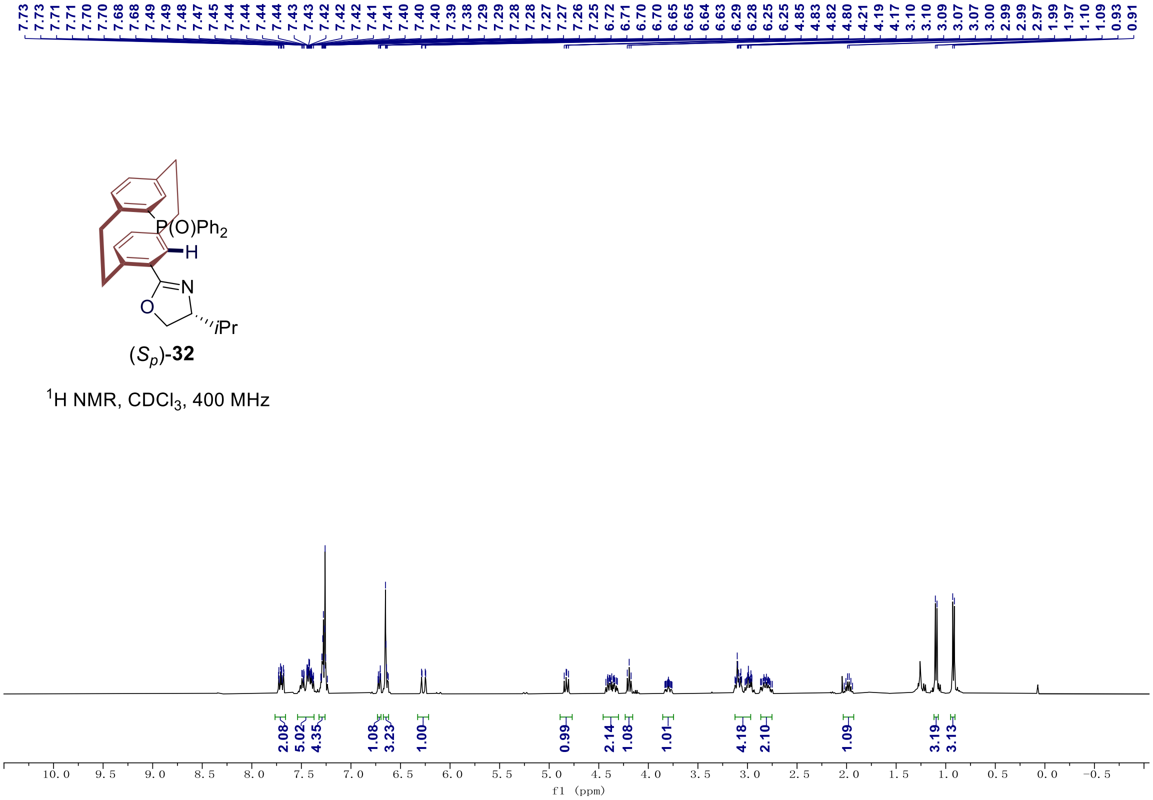
7.73 7.73 7.71 7.71 7.70 7.68 7.49 7.49 7.48 7.47 7.45 7.44 7.44 7.44 7.43 7.43 7.42 7.42 7.41 7.41 7.40 7.40 7.39 7.38 7.29 7.29 7.28 7.28 7.27 7.27 7.26 7.25 6.72 6.71 6.70 6.70 6.65 6.65 6.64 6.63 6.29 6.28 6.25 6.25 4.85 4.83 4.82 4.80 4.21 4.19 4.17 3.10 3.10 3.09 3.07 3.00 2.99 2.99 2.97 1.99 1.97 1.10 1.09 0.93 0.91
P(O)Ph2
H
N
O
iPr
(Sp)-32
1H NMR, CDCl3, 400 MHz
2.08 5.02 4.35 1.08 3.23 1.00 0.99 2.14 1.08 1.01 4.18 2.10 1.09 3.19 3.13
10.0 9.5 9.0 8.5 8.0 7.5 7.0 6.5 6.0 5.5 5.0 4.5 4.0 3.5 3.0 2.5 2.0 1.5 1.0 0.5 0.0 -0.5
f1 (ppm)

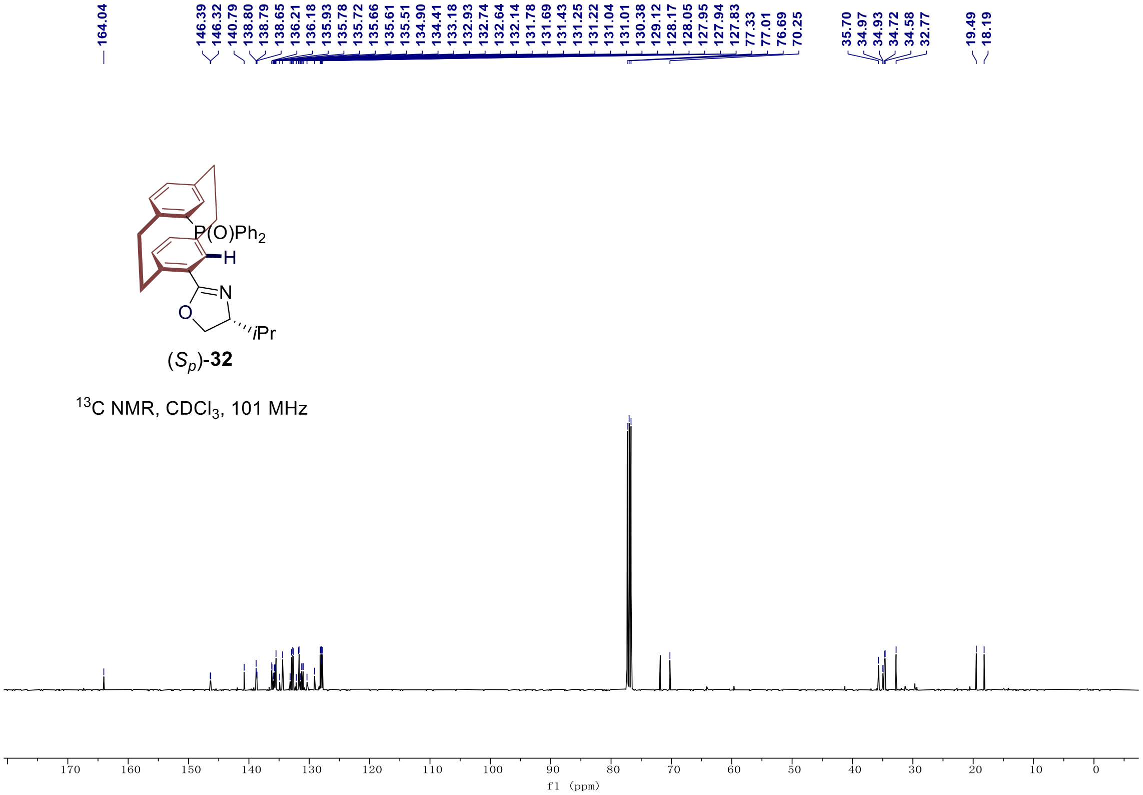
164.04 146.39 146.32 140.79 138.80 138.79 138.65 136.21 136.18 135.93 135.78 135.72 135.66 135.61 135.51 134.90 134.41 133.18 132.93 132.74 132.64 132.14 131.78 131.69 131.43 131.25 131.22 131.04 131.01 130.38 129.12 128.17 128.05 127.95 127.94 127.83 77.33 77.01 76.69 70.25 35.70 34.97 34.93 34.72 34.58 32.77 19.49 18.19
P(O)Ph2
H
N
O
iPr
(Sp)-32
13C NMR, CDCl3, 101 MHz
170 160 150 140 130 120 110 100 90 80 70 60 50 40 30 20 10 0
f1 (ppm)

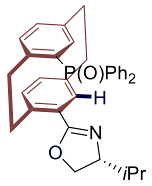

(S_p)-**32**

^{31}P NMR, $CDCl_3$, 162 MHz

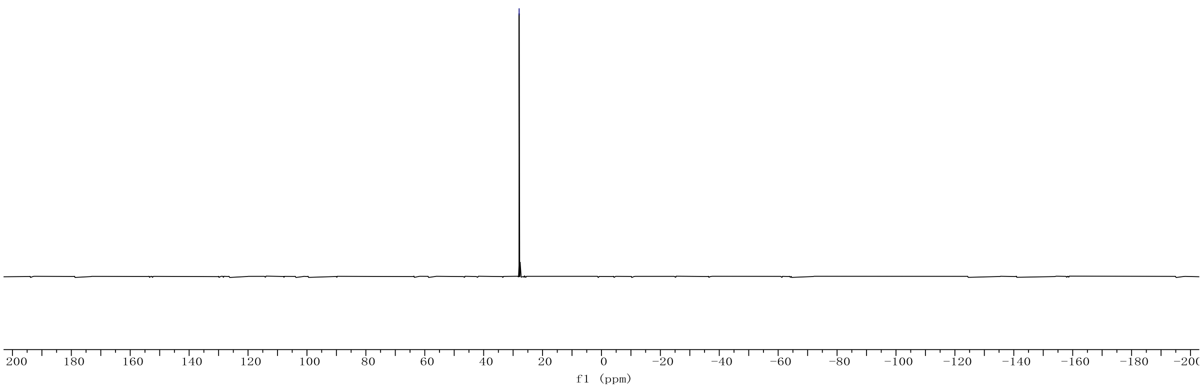

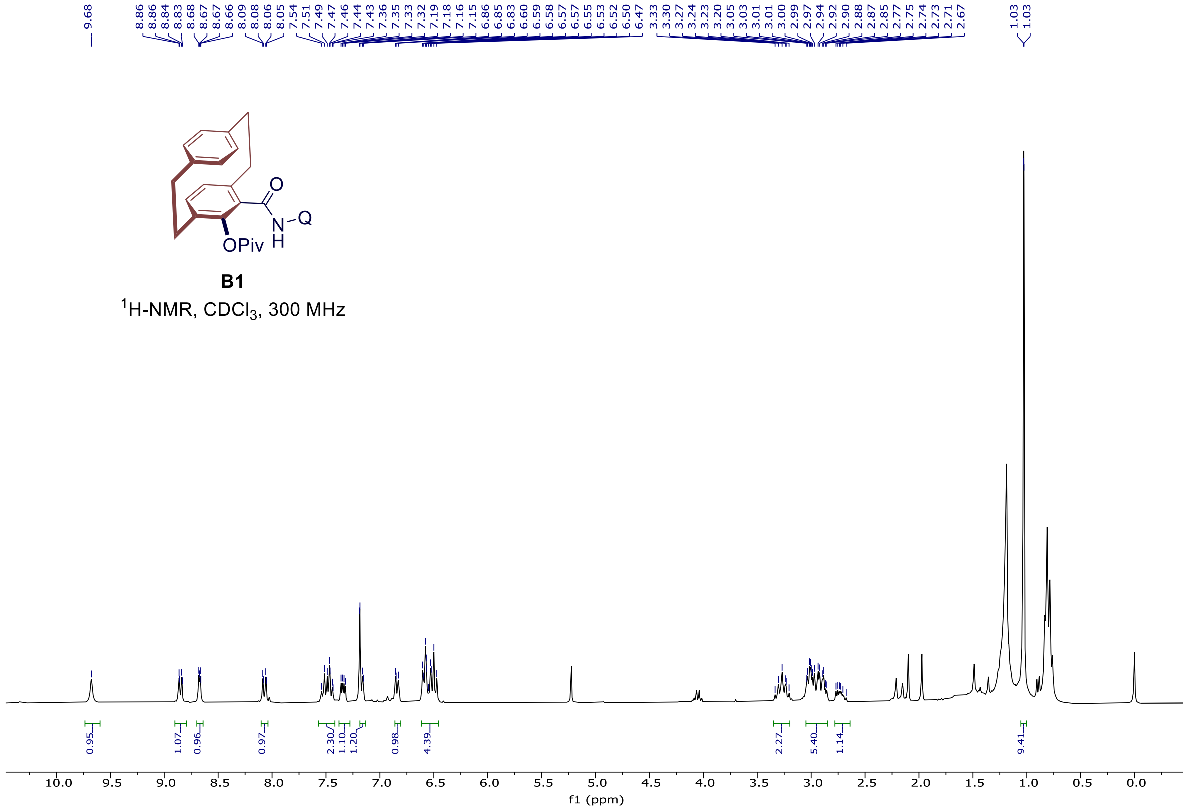
9.68
8.86 8.86 8.84 8.83 8.68 8.67 8.67 8.66 8.09 8.08 8.06 8.05 7.54 7.51 7.49 7.47 7.46 7.44 7.43 7.36 7.35 7.33 7.32 7.19 7.18 7.16 7.15 6.86 6.85 6.83 6.60 6.59 6.58 6.57 6.55 6.53 6.52 6.50 6.47
3.33 3.30 3.27 3.24 3.23 3.20 3.05 3.03 3.01 3.00 2.99 2.97 2.94 2.92 2.90 2.88 2.87 2.85 2.77 2.75 2.74 2.73 2.71 2.67
1.03 1.03
O
N–Q
H
OPiv
B1
1H-NMR, CDCl3, 300 MHz
0.95 1.07 0.96 0.97 2.30 1.10 1.20 0.98 4.39 2.27 5.40 1.14 9.41
f1 (ppm)

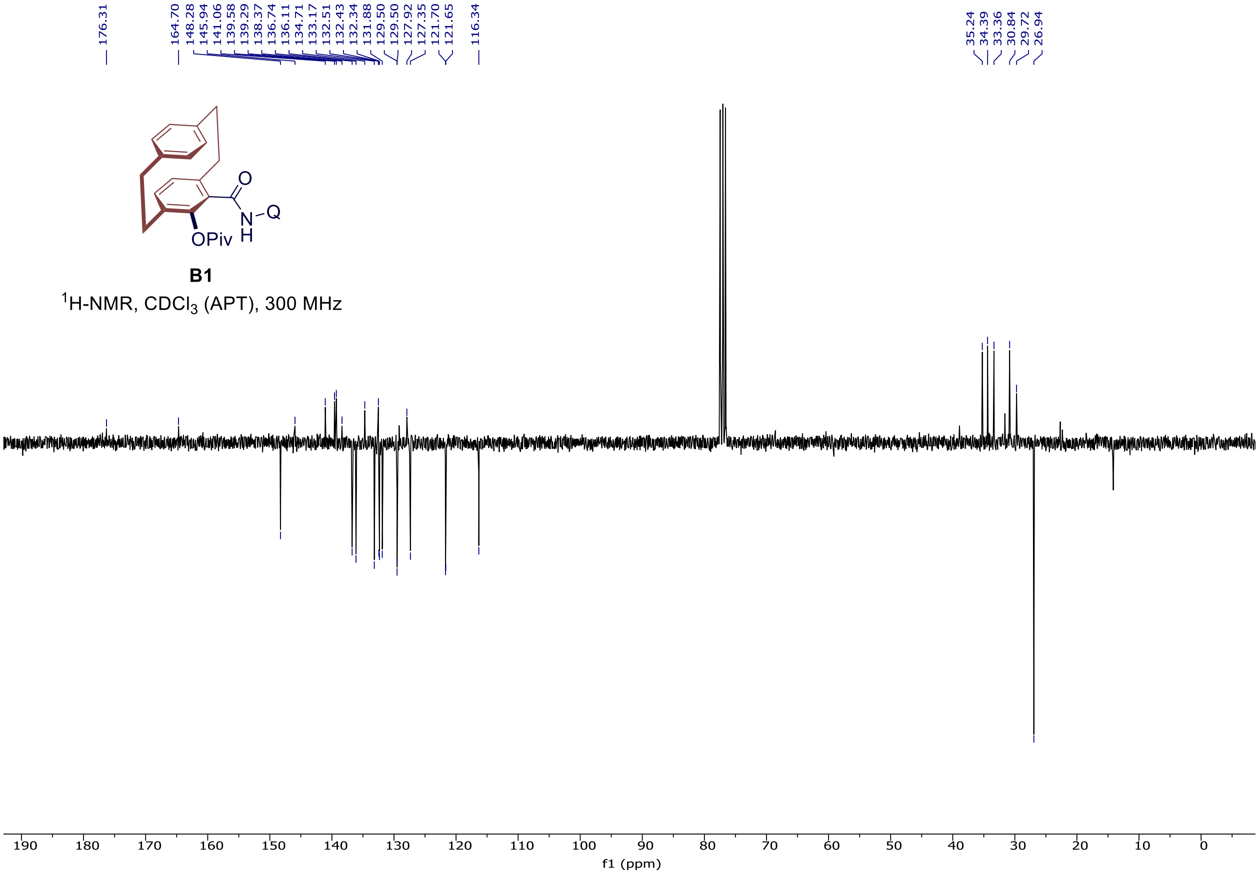
176.31
164.70 148.28 145.94 141.06 139.58 139.29 138.37 136.74 136.11 134.71 133.17 132.51 132.43 132.34 131.88 129.50 127.92 127.35 121.70 121.65 116.34
35.24 34.39 33.36 30.84 29.72 26.94
O
N–Q
H
OPiv
B1
1H-NMR, CDCl3 (APT), 300 MHz
f1 (ppm)

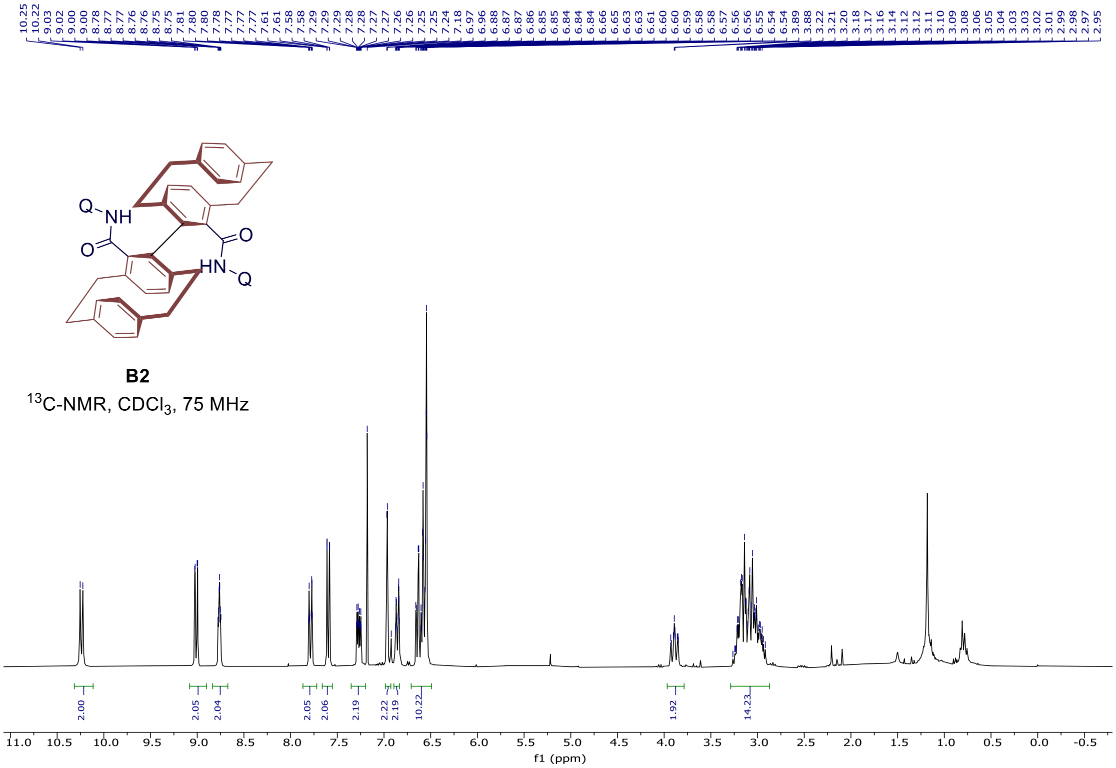
10.25 10.22 9.03 9.02 9.00 9.00 8.78 8.77 8.77 8.76 8.76 8.75 8.75 7.81 7.80 7.80 7.78 7.77 7.77 7.77 7.61 7.61 7.58 7.58 7.29 7.29 7.29 7.28 7.27 7.27 7.26 7.26 7.25 7.25 7.24 7.18 6.97 6.96 6.88 6.87 6.87 6.86 6.85 6.85 6.84 6.84 6.84 6.66 6.65 6.63 6.63 6.61 6.60 6.59 6.58 6.58 6.57 6.56 6.56 6.55 6.54 3.89 3.88 3.22 3.21 3.20 3.18 3.17 3.16 3.14 3.12 3.12 3.11 3.10 3.09 3.08 3.06 3.05 3.04 3.03 3.03 3.02 3.01 2.99 2.98 2.97 2.95
Q
NH
O
O
HN
Q
B2
^{13}C-NMR, $CDCl_3$, 75 MHz
2.00 2.05 2.04 2.05 2.06 2.19 2.22 2.19 10.22 1.92 14.23
f1 (ppm)

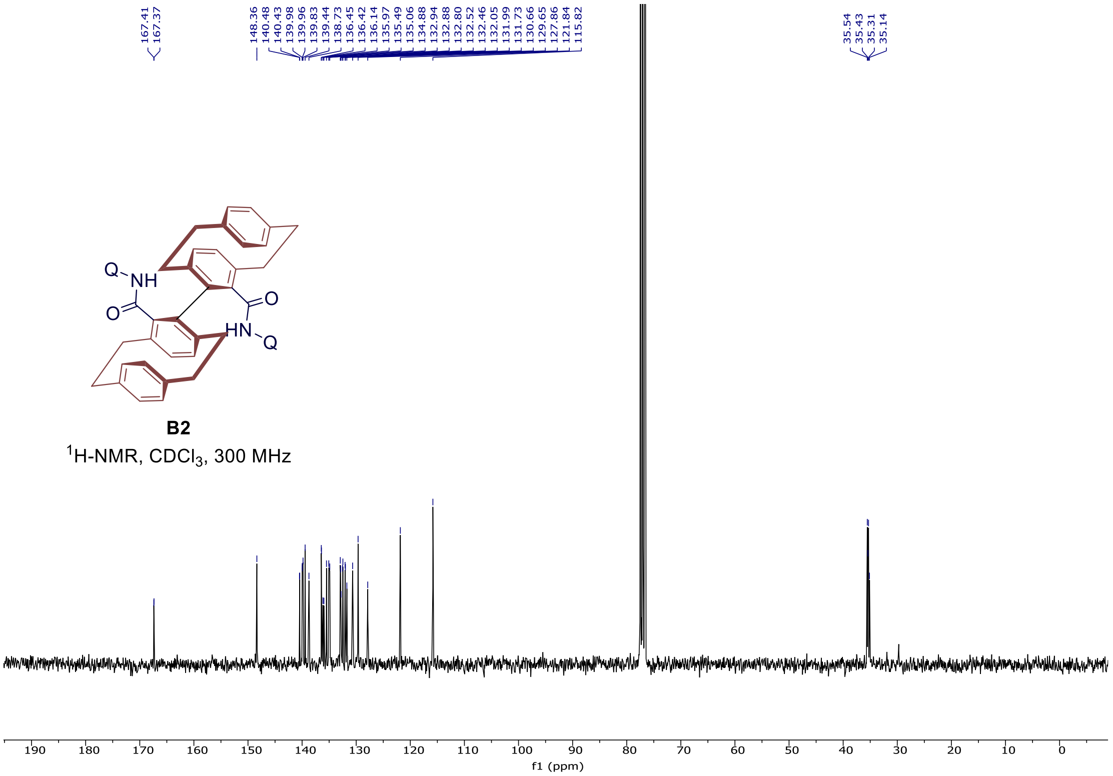
167.41 167.37
148.36 140.48 140.43 139.98 139.96 139.83 139.44 138.73 136.45 136.42 136.14 135.97 135.49 135.06 134.88 132.94 132.88 132.80 132.52 132.46 132.05 131.99 131.73 130.66 129.65 127.86 121.84 115.82
35.54 35.43 35.31 35.14
Q
NH
O
O
HN
Q
B2
^{1}H-NMR, $CDCl_3$, 300 MHz
f1 (ppm)
